# Supplementary material for: Gazing through time and beyond the health sector: Insights from a system dynamics model of cardiovascular disease in Australia
Source: PLoS One. 2021 Sep 30;16(9):e0257760. doi: 10.1371/journal.pone.0257760 (PMC8483334; doi:10.1371/journal.pone.0257760)
Supplement: S1 Appendices — (DOCX) [file pone.0257760.s001.docx]

# **S1 Appendices**

**Appendix 1. Key numeric input values related to health outcomes**

A 1.1. Core model structure

The list below summarises the key parameter values and their sources. For default values and assumptions of strategies, see **Appendix 1.2**.

| **Variable** | **Value** | | **Description/Additional notes** | **Source** |
| --- | --- | --- | --- | --- |
| Age-weighted risk multiplier | Graphical function, increasing over time | | This was applied to probabilities of having any CVD to account for rise in CVD incidence over model horizon from change in age structure.  It was calculated by applying current age-specific rate to population age structure, and the value increases from 1 in 2011 (baseline) to 1.63 in 2051. | (1) |
| In people who have not had previous CVD, current annual probability of having ACS and stroke | Male | 1% | Model calibration with hospitalisation and death data | (2) (1) |
|  | Female | 1% |  |  |
| Among people who have their first ACS or stroke, the proportion who die before reaching hospitals | 12.5% | | Model calibration with hospitalisation and death data | (2) (1) |
| Among people who are admitted to ED due to their first ACS or stroke, the proportion who die before being hospitalised | 3% | | Approximately a total of 5% of people who are admitted for ACS or stroke die in hospitals | (3) |
| Among people who are hospitalised for their first ACS or stroke, the proportion who die in or immediately after hospitals | 2% | | Approximately a total of 5% of people who are admitted for ACS or stroke die in hospitals | (3) |
| Among people who are hospitalised and discharged for their first ACS or stroke, the proportion who die from CVD in the first year | 0.5% | | Model calibration with hospitalisation and death data; note that people who have a recurrent attack and subsequently die are counted in a separate part of the model | (2) (1) |
| Among people who are hospitalised and discharged for their first ACS or stroke, the proportion who die from other causes in the first year | 5% | | Model calibration with hospitalisation and death data | (2) (1) |
| Among people who are hospitalised and discharged for ACS or stroke, the proportion who have another ACS or stroke in the subsequent year | 10% | | Model calibration with hospitalisation and death data | (2) (1) |
| In people who survived a year after an ACS or stroke, annual probability of having another ACS or stroke | Male | 2.5% | Annual average probability - probability of recurrent ACS or stroke in the first year is as high as 10% but wanes over time | (4, 5) |
|  | Female | 2.4% |  |  |
| Among people who have their subsequent ACS or stroke, the proportion who die before reaching hospitals | 25% | | Mortality rates approximately double in those who have recurrent ACS or stroke | (6) |
| Among people who are admitted to ED due to their subsequent ACS or stroke, the proportion who die before being hospitalised | 6% | | Mortality rates approximately double in those who have recurrent ACS or stroke | (6) |
| Among people who are hospitalised for their subsequent ACS or stroke, the proportion who die in or immediately after hospitals | 4% | | Mortality rates approximately double in those who have recurrent ACS or stroke | (6) |
| Among people who are hospitalised and discharged for their subsequent ACS or stroke, the proportion who die from CVD in the first year | 1% | | Model calibration with hospitalisation and death data; note that people who have a recurrent attack and subsequently die are counted in a separate part of the model; mortality rates approximately double in those who have recurrent ACS or stroke | (6) |
| Among people who have not had previous CVD, current annual probability of hospitalisation due to CVD other than ACS and stroke | Male | 1% | Model calibration with hospitalisation and death data | (2) (1) |
|  | Female | 1.2% |  |  |
| Among people who have their first ‘other CVD’, the proportion who die in and immediately after hospitalisation | 0.75% | | Model calibration with hospitalisation and death data | (2) (1) |
| After the first hospitalisation due to ‘other CVD’, annual probability of having another hospitalisation due to ‘other CVD’ | 10% | | Model calibration with hospitalisation and death data | (2) (1) |
| Among people living with chronic CV conditions after being hospitalised for *other CVD*, annual probability of having an ACS or stroke | 1.1% | | Model calibration with hospitalisation and death data | (2) (1) |
| Annual probability of death in people living with chronic CVD in the community | Male | 0.1% | Model calibration with hospitalisation and death data | (2) (1) |
|  | Female | 0.2% |  |  |
| Annual death rates for CVD before having any hospitalisation episodes | 1/1,000 | | ABS and model calibration | (2) |
| The average number of separation episodes recorded per CVD-related hospitalisation | 1.36 | | Used for calibration; unpublished estimate informed by the modelling consortium | Unpublished |

ACS = acute coronary syndrome, CVD = cardiovascular disease, ED = emergency department

A1.2. Current parameter values related to strategies and uncertainty assumptions

Current values were determined via evidence review or expert consensus. Uncertainty related to impacts of strategies were conducted with uncertainty ranges and distribution as specified below.

| **Variable** | **Current value** | **Uncertainty range^1^** | **Uncertainty distribution^2^** | **Source/rationale** |
| --- | --- | --- | --- | --- |
| **Salt reduction in processed food** | | | | |
| Roll-out duration | 4 years | (2, 6) | Uniform | (7) |
| Decrease in sodium content in processed food due to the strategy | 10% | (5, 15) | Uniform | (7) (8) |
| Current average salt intake | 9.6g | NA | NA | (9) (10) |
| Relative risk reduction in CVD with a 1000mg lower sodium intake | 17% | (13.6, 20.4) | Triangular | (11) |
| **Improving physical activity** | | | | |
| Reduction in physically inactive adults due to social marketing campaign | 3% | (1.5, 4.5) | Uniform | (12) |
| Increase in physically active population due to infrastructure | 3% | (1.5, 4.5) | Uniform | (13) |
| Lifespan of infrastructures | 40 years | NA | NA | Expert consensus |
| Annual net increase in infrastructures | 0.75% | NA | NA | Expert consensus |
| Relative risk reduction of CVD associated with being active | 17% | (11, 23) | Triangular | (14) |
| **Reducing smoking prevalence** | | | | |
| Time to implement standardized pack size | 2 years | (1, 3) | Uniform | Implementation assumption |
| Duration of social marketing campaign | 8 years | (4, 12) | Uniform | Implementation assumption |
| Increase in minimum price of cigarette | 10% | (5, 15) | Uniform | Implementation assumption |
| Relative risk of smokers compared to non-smokers | 1.6 | (1.5, 1.7) | Triangular | (15) |
| Time taken for CVD risk to normalize after a smoker quits | 8 years | (4, 12) | Triangular | (16) |
| **National public access defibrillator program** | | | | |
| Proportion of acute CVD deaths from cardiac arrest | 30% | (24, 36) | Triangular | Model calibration |
| Proportion of cardiac arrests that are preventable | 60% | (45, 75) | Uniform | (17, 18) |
| Decrease in deaths due to public access defibrillator | 50% | (25, 75) | Uniform | (19, 20) |
| Time for implementation | 5 years | (2.5, 7.5) | Uniform | Implementation assumption |
| **Reducing pre-hospital delay** | | | | |
| Current duration between symptom onset and arrival at ED | 5.2 hours | NA | NA | De Gruyter, Saunders (21) |
| Target pre-hospital delay | 2 hours | (1, 3) | Uniform | De Gruyter, Saunders (21) |
| Relative risk reduction in deaths at 30 days | 20% | (16, 24) | Triangular | De Gruyter, Saunders (21) |
| Time to reach target level | 5 years | (2.5, 7.5) | Uniform | Implementation assumption |
| **Prescription of preventive medications** | | | | |
| Time to reach target | 5 years | (2.5, 7.5) | Uniform | Implementation assumption |
| Increase in proportion of eligible persons currently prescribed BP lowering medications for primary prevention | 5% | (2.5, 7.5) | Uniform | (22) |
| Increase in proportion of eligible persons currently prescribed BP lowering medications for secondary prevention | 5% | (2.5, 7.5) | Uniform | (22) |
| Increase in proportion of eligible persons currently prescribed lipid lowering medications for primary prevention | 10% | (5, 15) | Uniform | (22) |
| Increase in proportion of eligible persons currently prescribed lipid lowering medications for secondary prevention | 10% | (5, 15) | Uniform | (22) |
| Relative reduction in CVD risk per 10mmHg reduction in systolic BP | 20% | (17, 23) | Triangular | (23) |
| Relative reduction in CVD risk from lipid-lowering medications | 22% | (17.6, 26.4) | Triangular | (24) |
| **Adherence to preventive medications** | | | | |
| Time to reach target | 5 years | (2.5, 7.5) | Uniform | (22) |
| Increase in adherence to BP lowering medications for primary prevention | 20% | (10, 30) | Uniform | (22) |
| Increase in adherence to BP lowering medications for secondary prevention | 20% | (10,30) | Uniform | (22) |
| Increase in adherence to lipid lowering medications for primary prevention | 20% | (10, 30) | Uniform | (22) |
| Increase in adherence to lipid lowering medications for secondary prevention | 20% | (10, 30) | Uniform | (22) |
| Relative reduction in CVD risk from BP-lowering medications | 20% | (16, 24) | Triangular | (25) |
| Relative reduction in CVD risk from lipid-lowering medications | 20% | (16, 24) | Triangular | (26) |
| **Cardiac rehabilitation program** | | | | |
| Time to reach target | 5 years | (2.5, 7.5) | Uniform | Implementation assumption |
| ACS patients eligible for cardiac rehab | 50% | (40, 60) | Triangular | (27, 28) |
| Increase in referral rate to cardiac rehab program | 30% | (24, 36) | Uniform | (27, 28) |
| Increase in attendance rate to cardiac rehab program | 15% | (12, 18) | Uniform | (27, 28) |
| Increase in completion rate to cardiac rehab program | 15% | (12, 18) | Uniform | (27, 28) |
| Relative risk reduction in recurrent MI | 20% | (8, 30) | Triangular | (29) |

1. Uncertainty ranges were determined by the evidence base: the 95% confidence intervals were used when available; or a relative difference of 50% or 20% depending on the strength of evidence, i.e. wider range when uncertainty level is high
2. Uniform distribution was used for parameters related to implementation of strategies and other parameters with high uncertainty (i.e. quality of evidence did not support an estimate with high confidence). Triangular distribution was mostly used for parameters related to underlying assumptions in potential impacts of strategies, with the modes being the most likely values

ACS = acute coronary syndrome, BP = blood pressure, CI = confidence intervals, ED = emergency department

**Sources:**

1. Australian Institute of Health and Welfare. Cardiovascular disease snapshot. Canberra: AIHW; 2018. Contract No.: Cat. no: CVD 83.

2. Australian Bureau of Statistics. Changing Patterns of Mortality in Australia, 1968-2017 2018.

3. Australian Institute of Health and Welfare. Monitoring acute coronary syndrome using national hospital data: an information paper on trends and issues. Canberra: AIHW; 2011.

4. Tangri N, Ferguson TW, Whitlock RH, Rigatto C, Jassal DS, Kass M, et al. Long term health outcomes in patients with a history of myocardial infarction: A population based cohort study. PLOS ONE. 2017;12(7):e0180010.

5. Gunnoo T, Hasan N, Khan MS, Slark J, Bentley P, Sharma P. Quantifying the risk of heart disease following acute ischaemic stroke: a meta-analysis of over 50,000 participants. BMJ open. 2016;6(1):e009535.

6. Radovanovic D, Maurer L, Bertel O, Witassek F, Urban P, Stauffer JC, et al. Treatment and outcomes of patients with recurrent myocardial infarction: A prospective observational cohort study. Journal of cardiology. 2016;68(6):498-503.

7. Prgram HFPR. Evidence Informing the Approach, Draft Targets and Modelling Outcomes. Healthy Food Partnership 2018.

8. He FJ, Brinsden HC, MacGregor GA. Salt reduction in the United Kingdom: a successful experiment in public health. Journal of human hypertension. 2014;28(6):345-52.

9. Land MA, Neal BC, Johnson C, Nowson CA, Margerison C, Petersen KS. Salt consumption by Australian adults: a systematic review and meta-analysis. The Medical journal of Australia. 2018;208(2):75-81.

10. Santos JA, Webster J, Land M-A, Flood V, Chalmers J, Woodward M, et al. Dietary salt intake in the Australian population. Public Health Nutrition. 2017;20(11):1887-94.

11. Cook NR, Appel LJ, Whelton PK. Lower levels of sodium intake and reduced cardiovascular risk. Circulation. 2014;129(9):981-9.

12. Leavy JE, Bull FC, Rosenberg M, Bauman A. Physical activity mass media campaigns and their evaluation: a systematic review of the literature 2003–2010. Health Education Research. 2011;26(6):1060-85.

13. Billie Giles-Corti PH, Sarah Foster, Mohammad Javad Koohsari, Jacinta Francis. Low density development: Impacts on physical activity and associated health outcomes. 2014.

14. Wahid A, Manek N, Nichols M, Kelly P, Foster C, Webster P, et al. Quantifying the Association Between Physical Activity and Cardiovascular Disease and Diabetes: A Systematic Review and Meta‐Analysis. Journal of the American Heart Association. 2016;5(9):n/a-n/a.

15. Banks E, Joshy G, Korda RJ, Stavreski B, Soga K, Egger S, et al. Tobacco smoking and risk of 36 cardiovascular disease subtypes: fatal and non-fatal outcomes in a large prospective Australian study. BMC Medicine. 2019;17(1):128.

16. Kawachi I, Colditz GA, Stampfer J, Willett WC, Manson JE, Rosner B, et al. Smoking Cessation and Time Course of Decreased Risks of Coronary Heart Disease in Middle-Aged Women. Archives of Internal Medicine. 1994;154(2):169-75.

17. Berdowski J, Blom MT, Bardai A, Tan HL, Tijssen JG, Koster RW. Impact of onsite or dispatched automated external defibrillator use on survival after out-of-hospital cardiac arrest. Circulation. 2011;124(20):2225-32.

18. Weisfeldt ML, Sitlani CM, Ornato JP, Rea T, Aufderheide TP, Davis D, et al. Survival after application of automatic external defibrillators before arrival of the emergency medical system: evaluation in the resuscitation outcomes consortium population of 21 million. Journal of the American College of Cardiology. 2010;55(16):1713-20.

19. van Alem AP, Vrenken RH, de Vos R, Tijssen JG, Koster RW. Use of automated external defibrillator by first responders in out of hospital cardiac arrest: prospective controlled trial. Bmj. 2003;327(7427):1312.

20. Kitamura T, Iwami T, Kawamura T, Nagao K, Tanaka H, Hiraide A. Nationwide public-access defibrillation in Japan. The New England journal of medicine. 2010;362(11):994-1004.

21. De Gruyter E, Saunders C, Stavreski B, Jennings G. Impact of Reducing Pre-Hospital Delay in Response to Heart Attack Symptoms in Australia. Heart, lung & circulation. 2019;28(8):1154-60.

22. Banks E, Crouch SR, Korda RJ, Stavreski B, Page K, Thurber K, et al. Absolute risk of cardiovascular disease events, and blood pressure- and lipid-lowering therapy in Australia. The Medical journal of Australia. 2016;204(8):320.

23. Ettehad D, Emdin CA, Kiran A, Anderson SG, Callender T, Emberson J, et al. Blood pressure lowering for prevention of cardiovascular disease and death: a systematic review and meta-analysis. Lancet (London, England). 2016;387(10022):957-67.

24. Ference BA, Ginsberg HN, Graham I, Ray KK, Packard CJ, Bruckert E, et al. Low-density lipoproteins cause atherosclerotic cardiovascular disease. 1. Evidence from genetic, epidemiologic, and clinical studies. A consensus statement from the European Atherosclerosis Society Consensus Panel. Eur Heart J. 2017;38(32):2459-72.

25. Ho CLB, Breslin M, Doust J, Reid CM, Nelson MR. Effectiveness of blood pressure-lowering drug treatment by levels of absolute risk: post hoc analysis of the Australian National Blood Pressure Study. BMJ Open. 2018;8(3):e017723.

26. Collins R, Reith C, Emberson J, Armitage J, Baigent C, Blackwell L, et al. Interpretation of the evidence for the efficacy and safety of statin therapy. Lancet (London, England). 2016;388(10059):2532-61.

27. Jackson AC, Higgins RO, Murphy BM, Rogerson M, Le Grande MR. Cardiac Rehabilitation in Australia: A Brief Survey of Program Characteristics. Heart, Lung and Circulation. 2018;27(12):1415-20.

28. Scott IA, Lindsay KA, Harden HE. Utilisation of outpatient cardiac rehabilitation in Queensland. Medical Journal of Australia. 2003;179(7):341-5.

29. Abell B, Glasziou P, Hoffmann T. The Contribution of Individual Exercise Training Components to Clinical Outcomes in Randomised Controlled Trials of Cardiac Rehabilitation: A Systematic Review and Meta-regression. Sports medicine - open. 2017;3(1):19.

**A2. Key numeric inputs related to economic outcomes**

A 2.1. Unit cost in the core model structure and cost consequences

Below are unit costs applied to each person or each episode. Each unit cost is stratified by payers, i.e. Commonwealth government, state governments, and consumers (‘out-of-pocket’ for health sector perspective and ‘households’ for societal perspective). The cost is based on the year 2017, and annual discount rate of 5% was applied.

| **Item** | **Payer** | **Cost** | **Additional Notes/Description** | **Source** |
| --- | --- | --- | --- | --- |
| Funeral | Out-of-pocket | $ 5,262 | Weighted cost by funeral type  Updated using consumer price inflation index | 1, 2 |
| Ambulance | Commonwealth | $1,081 | Weighted cost by State and population  Updated using health sector inflation rate  All individuals assumed to arrive by ambulance | 3 |
|  | States | $0.00 |  |  |
|  | Out-of-pocket | $270 |  |  |
| Emergency Department  ACS and Stroke - Male | Commonwealth | $0.00 | Applied when admitted, treated, and died in ED  AIHW reports cost of overall separation  IHPA reports proportion incurred in ED  Updated using hospital sector inflation rate | 4, 5, 6 |
|  | States | $1,155 |  |  |
|  | Out-of-pocket | $0.00 |  |  |
| Emergency Department  ACS and Stroke - Female | Commonwealth | $0.00 | Applied when admitted, treated and died in ED  AIHW reports cost of overall separation  IHPA reports proportion incurred in ED  Updated using hospital sector inflation rate | 4, 5, 6 |
|  | States | $1,043 |  |  |
|  | Out-of-pocket | $0.00 |  |  |
| Emergency Department  Other CVD – Male | Commonwealth | $0.00 | Applied when admitted, treated and died in ED  AIHW reports cost of overall separation  IHPA reports proportion incurred in ED  Updated using hospital sector inflation rate | 4, 5, 6 |
|  | States | $875 |  |  |
|  | Out-of-pocket | $0.00 |  |  |
| Emergency Department  Other CVD – Female | Commonwealth | $0.00 | Applied when admitted, treated and died in ED  AIHW reports cost of overall separation  IHPA reports proportion incurred in ED  Updated using hospital sector inflation rate | 4, 5, 6 |
|  | States | $ 746 |  |  |
|  | Out-of-pocket | $0.00 |  |  |
| Separation  ACS and Stroke - Male | Commonwealth | $0.00 | Applied for a completed separation  Death during a separation given 50% cost  Updated using hospital sector inflation rate | 4, 6 |
|  | States | $13,684 |  |  |
|  | Out-of-pocket | $0.00 |  |  |
| Separation  ACS and Stroke - Female | Commonwealth | $0.00 | Applied for a completed separation  Death during a separation given 50% cost  Updated using hospital sector inflation rate | 4, 6 |
|  | States | $12,359 |  |  |
|  | Out-of-pocket | $0.00 |  |  |
| Separation  Other CVD – Male | Commonwealth | $0.00 | Applied for a completed separation  Death during a separation given 50% cost  Updated using hospital sector inflation rate | 4, 6 |
|  | States | $10,369 |  |  |
|  | Out-of-pocket | $0.00 |  |  |
| Separation  Other CVD – Female | Commonwealth | $0.00 | Applied for a completed separation  Death during a separation given 50% cost  Updated using hospital sector inflation rate | 4, 6 |
|  | States | $8,841 |  |  |
|  | Out-of-pocket | $0.00 |  |  |
| Hospital outpatient  ACS and Stroke - Male | Commonwealth | $0.00 | Total costs, divided by number living with ACS and Stroke  Updated using hospital sector inflation rate | 4, 6, 7 |
|  | States | $587 |  |  |
|  | Out-of-pocket | $0.00 |  |  |
| Hospital outpatient  ACS and Stroke - Female | Commonwealth | $0.00 | Total costs, divided by number living with ACS and Stroke  Updated using hospital sector inflation rate | 4, 6, 7 |
|  | States | $530 |  |  |
|  | Out-of-pocket | $0.00 |  |  |
| Hospital outpatient  Other CVD - Male | Commonwealth | $0.00 | Total costs, divided by number living with ACS and Stroke  Updated using hospital sector inflation rate | 4, 6, 7 |
|  | States | $604 |  |  |
|  | Out-of-pocket | $0.00 |  |  |
| Hospital outpatient  Other CVD - Female | Commonwealth | $0.00 | Total costs, divided by number living with ACS and Stroke  Updated using MBS/PBS inflation rates | 4, 6, 7 |
|  | States | $515 |  |  |
|  | Out-of-pocket | $0.00 |  |  |
| Primary care management  ACS and Stroke - Male | Commonwealth | $452 | Total costs, divided by number living with ACS and Stroke  Updated using MBS/PBS inflation rates | 4, 6 |
|  | States | $0.00 |  |  |
|  | Out-of-pocket | $113 |  |  |
| Primary care management  ACS and Stroke – Female | Commonwealth | $343 | Total costs, divided by number living with ACS and Stroke  Updated using MBS/PBS inflation rates | 4, 6 |
|  | States | $0.00 |  |  |
|  | Out-of-pocket | $86 |  |  |
| Primary care management  Other CVD - Male | Commonwealth | $1,382 | Total costs, divided by number living with ACS and Stroke  Updated using MBS/PBS inflation rates | 4, 6 |
|  | States | $0.00 |  |  |
|  | Out-of-pocket | $346 |  |  |
| Primary care management  Other CVD - Female | Commonwealth | $1,506 | Total costs, divided by number living with Heart and Stroke  Updated using MBS/PBS inflation rates | 4, 6 |
|  | States | $0.00 |  |  |
|  | Out-of-pocket | $376 |  |  |
| Informal carers  Heart and Stroke | Households | $6,628 | Hours of informal care multiplied by the average wage rate  Weighted by carer employment rate (31%). | 8, 9, 10, 11, 12 |
|  |  |  |  |  |
| Informal carers  Other CVD | Households | $4,380 | Hours of informal care multiplied by average wage rate  Weighted by carer employment rate (31%). | 8, 9, 10, 11, 12 |
|  |  |  |  |  |
| Stroke aids | Households | $978 | Applied to 50% of patients, consistent with 50% of patients  Needing a carer | 10, 11 |
|  |  |  |  |  |
| Productivity loss  Death | Economy | $11,727 | Friction cost approach where loss is limited to first 3 months.  Cost is the average wage, weighted by employment rate, minus unemployment rate | 12, 13 |
|  |  |  |  |  |
| Productivity loss  Absenteeism  ACS and Stroke | Economy | $6,059 | Frictional cost approach where loss is limited to the first 3 months, included is return to work rates with the period.  Loss is average wage, weighted by employment rate, minus unemployment rate. | 12, 13, |
|  |  |  |  |  |
|  |  |  |  |  |
| Productivity loss  Absenteeism  Other CVD | Economy | $4,004 | Frictional cost approach where loss is limited to the first 3 months, included is return to work rates with the period.  Loss is average wage, weighted by employment rate, minus unemployment rate. | 11, 12, 13, 14 |
|  |  |  |  |  |
| Alive and CVD-Free  Average health cost | Commonwealth | $,1543 | Total recurrent health care expenditure, minus CVD, divided by  age-weighted population | 6 |
|  | States | $1,859 |  |  |
|  | Out-of-pocket | $867 |  |  |
| Alive and CVD-Free  Productivity benefit | Economy | $51,400 | Average wage, weighted by employment rate minus unemployment rate. | 12, 13 |
|  |  |  |  |  |

ACS = acute coronary syndrome, CVD = cardiovascular disease, ED = emergency department , IHPA = Independent Hospital Pricing Authority, MBS = Medicare Benefits Schedule (medical service subsidised by the Australian government), PBS = Pharmaceutical Benefits Scheme

A2.2. Quality Adjusted Life Years (QALYs): Health utility when CVD free and CVD utility decrements

| **Item** | **Value** |  | **Additional Notes/Description** | **Source** |
| --- | --- | --- | --- | --- |
| Free of CVD  Male | 0.758 |  | Population norms, average health utility, age adjusted | 15 |
|  |  |  |  |  |
| Free of CVD  Female | 0.736 |  | Population norms, average health utility, age adjusted | 15 |
|  |  |  |  |  |
| Living with Heart and Stroke  1st year - Male | 0.684 |  | Population norms minus utility decrement from event  Weighted Heart and Stroke decrement by event rates | 16 |
|  |  |  |  |  |
| Living with Heart and Stroke  Subsequent years - Male | 0.712 |  | Population norms minus utility decrement from event  Weighted Heart and Stroke decrement by event rates | 16 |
|  |  |  |  |  |
| Living with Heart and Stroke  1st year - Female | 0.662 |  | Population norms minus utility decrement from event  Weighted Heart and Stroke decrement by event rates | 16 |
| Living with Heart and Stroke  Subsequent years - Female | 0.690 |  | Population norms minus utility decrement from event  Weighted other CVD decrement (Heart Failure, peripheral arterial) | 16 |
| Living with other CVD  1st year - Male | 0.711 |  | Population norms minus utility decrement from event  Weighted other CVD decrement (Heart Failure, peripheral arterial) | 16 |
| Living with other CVD  Subsequent years - Male | 0.719 |  | Population norms minus utility decrement from event  Weighted other CVD decrement (Heart Failure, peripheral arterial) | 16 |
| Living with other CVD  1st year - Female | 0.689 |  | Population norms minus utility decrement from event  Weighted other CVD decrement (Heart Failure, peripheral arterial) | 16 |
| Living with other CVD  Subsequent years – Female | 0.697 |  | Population norms minus utility decrement from event  Weighted other CVD decrement (Heart Failure, peripheral arterial) | 16 |

CVD = cardiovascular disease

A 2.3. Unit costs relevant to strategies

The unit costs below are used for estimating cost consequences on health care from implementing strategies

| **Item** | **Funder** | **Cost** | **Additional Notes/Description** | **Source** |
| --- | --- | --- | --- | --- |
| Low dose diuretic | Commonwealth | $51 | Annual cost of drug in BP lowering  Converted to 2017 using PBS inflation rate | 6, 17 |
|  | States | $0.00 |  |  |
|  | Out-of-pocket | $18 |  |  |
| Calcium channel blocker | Commonwealth | $161 | Annual cost of drug in BP lowering  Converted to 2017 using PBS inflation rate | 6, 17 |
|  | States | $0.00 |  |  |
|  | Out-of-pocket | $53 |  |  |
| ACE inhibitor/therapy | Commonwealth | $128 | Annual cost of drug in BP lowering  Converted to 2017 using PBS inflation rate | 6, 17 |
|  | States | $0.00 |  |  |
|  | Out-of-pocket | $80 |  |  |
| Beta-blocker therapy | Commonwealth | $166 | Annual cost of drug in BP lowering  Converted to 2017 using PBS inflation rate | 6, 17 |
|  | States | $0.00 |  |  |
|  | Out-of-pocket | $46 |  |  |
| Statins | Commonwealth  States  Out-of-pocket | $499  $0.00  $175 | Annual cost of drug in LDL lowering  Converted to 2017 using PBS inflation rate | 6, 17 |
| Rehabilitation | Commonwealth | $0.00 | Applied once to each separation  Converted to 2017 using PBS inflation rate | 6, 17 |
|  | States | $722 |  |  |
|  | Out-of-pocket | $181 |  |  |

BP = blood pressure, LDL = low-density lipoproteins, PBS = pharmaceutical benefits scheme

A2.4. Alternative assumptions built in for future sensitivity analysis

| **Item** | **Funder** | **Cost** | **Additional Notes/Description** | **Source** |
| --- | --- | --- | --- | --- |
| Productivity loss  Death | Economy | $51,400 | Human capital approach. Applied every year of working life (40-65)  Average wage, weighted by employment rate, minus unemployment rate | 12, 13 |
|  |  |  |  |  |
| Productivity loss  Absenteeism  Heart and Stroke | Economy | $14,392 | Human capital approach. Applied every year of working life (40-65)  Average wage, weighted by employment rate, minus unemployment rate | 12, 13 |
|  |  |  |  |  |
|  |  |  |  |  |
| Productivity loss  Absenteeism  Other CVD | Economy | $9,511 | Human capital approach. Applied every year of working life (40-65)  Average wage, weighted by employment rate, minus unemployment rate | 12, 13 |
|  |  |  |  |  |
|  |  |  |  |  |
| Informal carers – value of time  ACS and Stroke | Society | $21,468 | All carers hours multiplied by average formal carer wage rate.  Not a cost, but value of time. Used to highlight value of unpaid care. | 12, 13 |
|  |  |  |  |  |
| Informal carers – value of time  ACS and Stroke | Society | $14,187 | All carers hours multiplied by average formal carer wage rate.  Not a cost, but value of time. Used to highlight value of unpaid care. | 8, 9, 10, 11, 19 |
| Value of Statistical Life | Society | $182,000 | Applied to a CVD death and each year of life lost.  Used by certain non-health sectors (e.g. transport) and endorsed  by Prime Minister and Cabinet. The dollar value is not universally accepted in academia or industry and therefore reserved for use in a sensitivity analysis. | 20 |
|  |  |  |  |  |
|  |  |  |  |  |

ACS = acute coronary syndrome, CVD = cardiovascular disease

**Sources**

1. Bureau of Transport Economics, Road Crash Costs in Australia, Report 102, 2000
2. Reserve Bank of Australia, Measures of Consumer Price Inflation, April 2019
3. Report on Government Services: Chapter 11 Ambulance Services, Productivity Commission,
4. Health-care expenditure on cardiovascular diseases 2008–09, Australian Institute of Health and Welfare (AIHW), 2012
5. Hospital Independent Pricing Authority, National Hospital Cost Data Collection, Round 19, 2014-15.
6. Health Care Expenditure in Australia 2016-17, Australian Institute of Health and Welfare (AIHW)
7. Health system costs of cardiovascular diseases and diabetes in Australia 1993-94, Australian Institute of Health and Welfare (AIHW), 1999
8. J L Y Liu, N Maniadakis, A Gray, M Rayner, The economic burden of coronary heart disease in the UK, Heart 2002;88:597–603
9. Survey of Disability, Ageing, and Caring (SDAC), Australia Bureau of Statistics (ABS), 2015
10. National Stroke Audit – rehabilitation services report-2012. Melbourne, Victoria: National Stroke Foundation, 2012.
11. The Economic Impact of Stroke, Deloitte Access Economics, 2013
12. Earnings and Work Hours, Australian Bureau of Statistics (ABS), November 2017
13. Labour Force Australia, Australia Bureau of Statistics, November 2017
14. National Heart Foundation of Australia, Heart Attack Survivors Survey, Melbourne, 2014
15. Norman R et al, Australian health-related quality of life population norms derived from the SF-6D, Aust NZ J Public Health. 2013; 37:17-23
16. Lawson et al, A Cardiovascular Disease Policy Model: part 2 – preparing for economic evaluation
17. Cobiac et al. Improving the cost-effectiveness of cardiovascular disease prevention in Australia: a modelling study, BMC Public Health 2012, 12:39
18. De Gruyter et al, Economic and Social Impact of Increasing Uptake of Cardiac Rehabilitation Services: a cost benefit analysis, Heart, Lung and Circulation 2016, 25 175:183
19. The Economic Impact of Informal Care in Australia 2015, Carers Australia, Deloitte Access Economics
20. Best Practice Regulation Guidance Note Value of Statistical Life, Department of Prime Minister and Cabinet, Australian Government, 2014.

**Appendix 2. Key assumptions**

The following assumptions are relevant to the core component of the model. Please see Appendix 3 for default values and assumptions of strategies.

| **Assumptions** | **Source/rationale** |
| --- | --- |
| Cardiovascular disease is separated into acute CVD (i.e. ACS and stroke) and ‘other CVD’. People with acute or ‘other CVD’ have different probability for hospitalisation and death. | This compartmentalisation allows for strategies to have differential impacts on acute and chronic CVD; it is also comparable to administrative data which the model is calibrated against |
| The incidence of CVD over time is driven by projection with population change and age-specific incidence rates. The baseline incidence also includes a projection of CVD risk associated with a declining smoking prevalence. | As the risk for CVD changes with age, the incidence of CVD at the population level is likely to change with population structure in the future |
| Future trend of smoking prevalence is projected with current legislated policies and their impacts on smoking cessation. Strategy of further driving down smoking prevalence includes stricter legislations on pricing as well as social marketing campaigns. | Smoking prevalence is expected to continue downwards, which would contribute to a lower CVD incidence |
| Absolute CVD risk is incorporated into the model through selection of eligible individuals for preventive medications. Individuals are considered eligible for preventive medications if they have: a high absolute CVD risk, or high blood pressure or lipid levels, or previous CVD, according to the National Health Survey 2011. | This approach is in line with recommendations from clinical guidelines and data available in Australia.  *National Vascular Disease Prevention Alliance. Guidelines for the management of absolute cardiovascular disease risk. 2012*  *Banks, E., et al. (2016). "Absolute risk of cardiovascular disease events, and blood pressure- and lipid-lowering therapy in Australia." Med J Aust 204(8): 320.* |
| Implementation of strategies (i.e. specific interventions) are not costed, instead cost consequences from various strategies can be used to inform investment and set goals for strategies.  Note that the cost consequences occurring in the health sector are captured as flow-on use of health service but those beyond the health sector (e.g. change in tax from tobacco industry, construction of roads and parks, and household spending on food) are out of the scope of the current model. | Evidence is insufficient to support specific interventions that would achieve the assumed/modelled impacts of the strategies. Therefore, the economic analysis, and the model itself, has been constructed to inform high-level strategic planning. For example, the cost saved from 10% reduction of salt intake as a result of lower health service use can inform the level of investment in a program that delivers the 10% reduction. |
| Economic impacts under the health system perspective include costs associated with health service provision, including medication, primary care management, ambulance, emergency department, hospitalisation, and rehabilitation. Under the societal perspective, costs associated with carers’ time and productivity loss/gains are also added. | Both perspectives are used in the model for various audiences to consider the economic impact within and beyond the health sector. |
| Under the societal perspective, frictional cost approach is used to estimated productivity loss; informal carer time is valued at hours used multiplied by average wage and weighted by carer employment rate (31%); monetised health gains are valued at $50,000 per QALY gained. | The approaches used have been chosen to produce a relatively conservative estimate of economic impacts. Sensitivity analyses can be run with alternative approaches, that is, human capital approach to estimate productivity loss, carer valued by multiplying time and formal carer wage rate, and applying value of statistical life of $182,000 to CVD deaths and each year of life lost. |

ACS = acute coronary syndrome, CVD = cardiovascular disease

**Appendix 3. Model Equations**

The model file can be provided on request.

Top-Level Model:

Accum_HS_Deaths(t) = Accum_HS_Deaths(t - dt) + (inc_deaths) * dt {NON-NEGATIVE}

INIT Accum_HS_Deaths = 0

INFLOWS:

inc_deaths = All_HS_Deaths_pa {UNIFLOW}

Acute_Subsequent_pre_hospital[Gender](t) = Acute_Subsequent_pre_hospital[Gender](t - dt) + ("2nd_Plus_attack"[Gender] + First_year_1_attacks[Gender] + attack_subsequent_first_year[Gender] - in_to_ED_2plus[Gender] - Subsequent_Attack_pre_ED_Deaths[Gender]) * dt {CONVEYOR}

INIT Acute_Subsequent_pre_hospital[Gender] = 0

TRANSIT TIME = DT

CONTINUOUS

ACCEPT MULTIPLE BATCHES

INFLOWS:

"2nd_Plus_attack"[Gender] = People_with_Chronic_HS*Prob_subsequent_attacks*Combined_Secondary_RR_HS {UNIFLOW}

INFLOW PRIORITY: 3

OUTFLOW PRIORITY: 1

First_year_1_attacks[Gender] = LEAKAGE OUTFLOW

LEAKAGE FRACTION = Prob_attack_in_first_year_%/100*Combined_Secondary_RR_HS

LINEAR LEAKAGE

LEAK ZONE = 0% to 100%

INFLOW PRIORITY: 1

OUTFLOW PRIORITY: 2

attack_subsequent_first_year[Gender] = LEAKAGE OUTFLOW

LEAKAGE FRACTION = Prob_attack_in_first_year_%/100*prior_attack_mpr*Combined_Secondary_RR_HS

LINEAR LEAKAGE

LEAK ZONE = 0% to 100%

INFLOW PRIORITY: 2

OUTFLOW PRIORITY: 3

OUTFLOWS:

in_to_ED_2plus[Gender] = CONVEYOR OUTFLOW

Subsequent_Attack_pre_ED_Deaths[Gender] = LEAKAGE OUTFLOW

LEAKAGE FRACTION = Subsequent_attack_fatality_mpr*adj_pre_hospital_fatality_rate_%/100

LINEAR LEAKAGE

LEAK ZONE = 0% to 100%

all_cvd_admits_cumulative(t) = all_cvd_admits_cumulative(t - dt) + (Flow_34) * dt {NON-NEGATIVE}

INIT all_cvd_admits_cumulative = 0

INFLOWS:

Flow_34 = All_CVD_admits {UNIFLOW}

All_HS_Deaths_1(t) = All_HS_Deaths_1(t - dt) + (Flow_18) * dt {NON-NEGATIVE}

INIT All_HS_Deaths_1 = 0

INFLOWS:

Flow_18 = HS_pa {UNIFLOW}

Cumulative_HS_admits_custom_time_horizons[comp_period](t) = Cumulative_HS_admits_custom_time_horizons[comp_period](t - dt) + (Flow_33[comp_period]) * dt {NON-NEGATIVE}

INIT Cumulative_HS_admits_custom_time_horizons[comp_period] = 0

INFLOWS:

Flow_33[comp_period] = IF TIME >= Comparison_start_time AND TIME <(Comparison_start_time+custom_time_horizon) THEN Total_HS_Admits ELSE 0 {UNIFLOW}

Cumulative_HS_admits_from_2011(t) = Cumulative_HS_admits_from_2011(t - dt) + (Flow_25) * dt {NON-NEGATIVE}

INIT Cumulative_HS_admits_from_2011 = 0

INFLOWS:

Flow_25 = Total_HS_Admits {UNIFLOW}

Cumulative_HS_deaths_from_2011(t) = Cumulative_HS_deaths_from_2011(t - dt) + (Flow_21) * dt {NON-NEGATIVE}

INIT Cumulative_HS_deaths_from_2011 = 0

INFLOWS:

Flow_21 = All_HS_Deaths_pa {UNIFLOW}

Cumulative_HS_deaths_from_2019[comp_period](t) = Cumulative_HS_deaths_from_2019[comp_period](t - dt) + (Flow_22[comp_period]) * dt {NON-NEGATIVE}

INIT Cumulative_HS_deaths_from_2019[comp_period] = 0

INFLOWS:

Flow_22[comp_period] = IF TIME >= Comparison_start_time AND TIME <(Comparison_start_time+custom_time_horizon) THEN All_HS_Deaths_pa ELSE 0 {UNIFLOW}

Cumulative_other_circulatory_deaths_from_2019[comp_period](t) = Cumulative_other_circulatory_deaths_from_2019[comp_period](t - dt) + (Flow_24[comp_period]) * dt {NON-NEGATIVE}

INIT Cumulative_other_circulatory_deaths_from_2019[comp_period] = 0

INFLOWS:

Flow_24[comp_period] = IF TIME >= Comparison_start_time AND TIME <(Comparison_start_time+custom_time_horizon) THEN Other_circulatory_deaths_total ELSE 0 {UNIFLOW}

Cumulative_other_cvd_admits_from_2011(t) = Cumulative_other_cvd_admits_from_2011(t - dt) + (Flow_27) * dt {NON-NEGATIVE}

INIT Cumulative_other_cvd_admits_from_2011 = 0

INFLOWS:

Flow_27 = Total_other_CVD_admits {UNIFLOW}

Cumulative_other_cvd_admits_from_2019[comp_period](t) = Cumulative_other_cvd_admits_from_2019[comp_period](t - dt) + (Flow_28[comp_period]) * dt {NON-NEGATIVE}

INIT Cumulative_other_cvd_admits_from_2019[comp_period] = 0

INFLOWS:

Flow_28[comp_period] = IF TIME >= Comparison_start_time AND TIME <(Comparison_start_time+custom_time_horizon) THEN Total_other_CVD_admits ELSE 0 {UNIFLOW}

Cumulative_other_CVD_deaths_from_2011(t) = Cumulative_other_CVD_deaths_from_2011(t - dt) + (Flow_23) * dt {NON-NEGATIVE}

INIT Cumulative_other_CVD_deaths_from_2011 = 0

INFLOWS:

Flow_23 = Other_circulatory_deaths_total {UNIFLOW}

Cumulative_total_attacks_from_2019[comp_period](t) = Cumulative_total_attacks_from_2019[comp_period](t - dt) + (Flow_29[comp_period]) * dt {NON-NEGATIVE}

INIT Cumulative_total_attacks_from_2019[comp_period] = 0

INFLOWS:

Flow_29[comp_period] = IF TIME >= Comparison_start_time AND TIME <(Comparison_start_time+custom_time_horizon) THEN Total_attacks ELSE 0 {UNIFLOW}

ED_Dept[Gender](t) = ED_Dept[Gender](t - dt) + (into_ED[Gender] - admit_1[Gender] - First_Attack_ED_deaths[Gender]) * dt {CONVEYOR}

INIT ED_Dept[Gender] = 0

TRANSIT TIME = DT

CONTINUOUS

ACCEPT MULTIPLE BATCHES

INFLOWS:

into_ED[Gender] = CONVEYOR OUTFLOW

OUTFLOWS:

admit_1[Gender] = CONVEYOR OUTFLOW

First_Attack_ED_deaths[Gender] = LEAKAGE OUTFLOW

LEAKAGE FRACTION = ED_deaths_%/100*Reduce_Pre_hospital_delay.Death_%_reduction_mpr*Age_Specific_Mpr.Hospital_Death_Rate_MPR

LINEAR LEAKAGE

LEAK ZONE = 0% to 100%

ED_Dept_2[Gender](t) = ED_Dept_2[Gender](t - dt) + (in_to_ED_2plus[Gender] - admit_3[Gender] - Subsequent_attack_ED_2_deaths[Gender]) * dt {CONVEYOR}

INIT ED_Dept_2[Gender] = 0

TRANSIT TIME = DT

CONTINUOUS

ACCEPT MULTIPLE BATCHES

INFLOWS:

in_to_ED_2plus[Gender] = CONVEYOR OUTFLOW

OUTFLOWS:

admit_3[Gender] = CONVEYOR OUTFLOW

Subsequent_attack_ED_2_deaths[Gender] = LEAKAGE OUTFLOW

LEAKAGE FRACTION = ED_deaths_%/100*Subsequent_attack_fatality_mpr*Reduce_Pre_hospital_delay.Death_%_reduction_mpr*Age_Specific_Mpr.Hospital_Death_Rate_MPR

LINEAR LEAKAGE

LEAK ZONE = 0% to 100%

First_Year[Gender](t) = First_Year[Gender](t - dt) + (survive_first_attack[Gender] - survive_first_year[Gender] - First_year_1_attacks[Gender] - other_deaths_first_year[Gender] - other_CVD_deaths_first_year[Gender]) * dt {CONVEYOR}

INIT First_Year[Gender] = 0

TRANSIT TIME = 1

CONTINUOUS

ACCEPT MULTIPLE BATCHES

INFLOWS:

survive_first_attack[Gender] = CONVEYOR OUTFLOW

OUTFLOWS:

survive_first_year[Gender] = CONVEYOR OUTFLOW

OUTFLOW PRIORITY: 1

First_year_1_attacks[Gender] = LEAKAGE OUTFLOW

LEAKAGE FRACTION = Prob_attack_in_first_year_%/100*Combined_Secondary_RR_HS

LINEAR LEAKAGE

LEAK ZONE = 0% to 100%

INFLOW PRIORITY: 1

OUTFLOW PRIORITY: 2

other_deaths_first_year[Gender] = LEAKAGE OUTFLOW

LEAKAGE FRACTION = Die_other_first_year_%/100

LINEAR LEAKAGE

LEAK ZONE = 0% to 100%

OUTFLOW PRIORITY: 3

other_CVD_deaths_first_year[Gender] = LEAKAGE OUTFLOW

LEAKAGE FRACTION = first_year_other_CVD_death_%/100*Reduce_Pre_hospital_delay.Death_%_reduction_mpr

LINEAR LEAKAGE

LEAK ZONE = 0% to 100%

OUTFLOW PRIORITY: 4

"H\\S_5year"[Gender](t) = "H\\S_5year"[Gender](t - dt) + (Flow_12[Gender] - Flow_13[Gender]) * dt {CONVEYOR}

INIT "H\\S_5year"[Gender] = 0

TRANSIT TIME = 5

CONTINUOUS

ACCEPT MULTIPLE BATCHES

INFLOWS:

Flow_12[Gender] = HS_deaths {UNIFLOW}

OUTFLOWS:

Flow_13[Gender] = CONVEYOR OUTFLOW

Hospital[Gender](t) = Hospital[Gender](t - dt) + (admit_1[Gender] - survive_first_attack[Gender] - First_attack_and_other_CVD_hosp_deaths[Gender]) * dt {CONVEYOR}

INIT Hospital[Gender] = 0

TRANSIT TIME = 30/365

CONTINUOUS

ACCEPT MULTIPLE BATCHES

INFLOWS:

admit_1[Gender] = CONVEYOR OUTFLOW

OUTFLOWS:

survive_first_attack[Gender] = CONVEYOR OUTFLOW

First_attack_and_other_CVD_hosp_deaths[Gender] = LEAKAGE OUTFLOW

LEAKAGE FRACTION = Hospital_Fatality_rate_%/100*Reduce_Pre_hospital_delay.Death_%_reduction_mpr*Age_Specific_Mpr.Hospital_Death_Rate_MPR

LINEAR LEAKAGE

LEAK ZONE = 0% to 100%

"HS_Seps_2016-17"(t) = "HS_Seps_2016-17"(t - dt) + (Flow_31) * dt {NON-NEGATIVE}

INIT "HS_Seps_2016-17" = 0

INFLOWS:

Flow_31 = IF TIME >=2016.5 AND TIME < 2017.5 THEN total_HS_Separations ELSE 0 {UNIFLOW}

no_CVD[M](t) = no_CVD[M](t - dt) + (turn_40[M] + migrants_40p[M] - die_no_CVD[M] - die_at_home_other_CVD[M] - First_Attack_no_prior_cvd[M] - admit_2[M]) * dt {NON-NEGATIVE}

INIT no_CVD[M] = 4983391-People_with_Chronic_HS[M]-People_with_Chronic_non_HS_CVD[M]

no_CVD[F](t) = no_CVD[F](t - dt) + (turn_40[F] + migrants_40p[F] - die_no_CVD[F] - die_at_home_other_CVD[F] - First_Attack_no_prior_cvd[F] - admit_2[F]) * dt {NON-NEGATIVE}

INIT no_CVD[F] = 5289657-People_with_Chronic_HS[F]-People_with_Chronic_non_HS_CVD[F]

Non_Circ_Deaths[Gender](t) = Non_Circ_Deaths[Gender](t - dt) + (Flow_16[Gender] - Flow_17[Gender]) * dt {CONVEYOR}

INIT Non_Circ_Deaths[Gender] = 0

TRANSIT TIME = 5

CONTINUOUS

ACCEPT MULTIPLE BATCHES

INFLOWS:

Flow_16[Gender] = non_cvd_deaths {UNIFLOW}

OUTFLOWS:

Flow_17[Gender] = CONVEYOR OUTFLOW

other_Circ_5_year[Gender](t) = other_Circ_5_year[Gender](t - dt) + (Flow_30[Gender] - Flow_15[Gender]) * dt {CONVEYOR}

INIT other_Circ_5_year[Gender] = 0

TRANSIT TIME = 5

CONTINUOUS

ACCEPT MULTIPLE BATCHES

INFLOWS:

Flow_30[Gender] = Other_Circ_diseases_deaths {UNIFLOW}

OUTFLOWS:

Flow_15[Gender] = CONVEYOR OUTFLOW

Other_CVD_firstYears[Gender](t) = Other_CVD_firstYears[Gender](t - dt) + (Flow_19[Gender] - Flow_20[Gender]) * dt {CONVEYOR}

INIT Other_CVD_firstYears[Gender] = 0

TRANSIT TIME = 1

CONTINUOUS

ACCEPT MULTIPLE BATCHES

INFLOWS:

Flow_19[Gender] = live_discharge_other_CVD {UNIFLOW}

OUTFLOWS:

Flow_20[Gender] = CONVEYOR OUTFLOW

Other_CVD_hospital[Gender](t) = Other_CVD_hospital[Gender](t - dt) + (admit_2[Gender] + subsequent_CVD_admits[Gender] - live_discharge_other_CVD[Gender] - other_CVD_hospital_deaths[Gender]) * dt {CONVEYOR}

INIT Other_CVD_hospital[Gender] = 0

TRANSIT TIME = 30/365

CONTINUOUS

ACCEPT MULTIPLE BATCHES

INFLOWS:

admit_2[Gender] = no_CVD*All_Other_CVD_first_hospitalisations_%_ap/100*Age_Specific_Mpr.age_weighted_risk_mpr*Combined_Effect_Primary {UNIFLOW}

INFLOW PRIORITY: 1

OUTFLOW PRIORITY: 4

subsequent_CVD_admits[Gender] = People_with_Chronic_non_HS_CVD*prob_annual_hospital_other_pa/100*Combined_Secondary_RR_non_HS {UNIFLOW}

INFLOW PRIORITY: 2

OUTFLOW PRIORITY: 2

OUTFLOWS:

live_discharge_other_CVD[Gender] = CONVEYOR OUTFLOW

other_CVD_hospital_deaths[Gender] = LEAKAGE OUTFLOW

LEAKAGE FRACTION = Other_hospital_CVD_death_rate_%/100

LINEAR LEAKAGE

LEAK ZONE = 0% to 100%

"other_CVD_Seps_2016-_17"(t) = "other_CVD_Seps_2016-_17"(t - dt) + (Flow_32) * dt {NON-NEGATIVE}

INIT "other_CVD_Seps_2016-_17" = 0

INFLOWS:

Flow_32 = IF TIME >=2016.5 AND TIME < 2017.5 THEN CVD_seps ELSE 0 {UNIFLOW}

Other_HS_relatedhospital[Gender](t) = Other_HS_relatedhospital[Gender](t - dt) + (other_HS_hospitalisations[Gender] - live_other_Hs_separations[Gender]) * dt {CONVEYOR}

INIT Other_HS_relatedhospital[Gender] = 0

TRANSIT TIME = DT

CONTINUOUS

ACCEPT MULTIPLE BATCHES

INFLOWS:

other_HS_hospitalisations[Gender] = People_with_Chronic_HS*Other_heart_hospitalisations_%_pa/100 {UNIFLOW}

OUTFLOW PRIORITY: 4

OUTFLOWS:

live_other_Hs_separations[Gender] = CONVEYOR OUTFLOW

People_with_Chronic_HS[Gender](t) = People_with_Chronic_HS[Gender](t - dt) + (survive_first_year[Gender] + survive_subsequent_first_year[Gender] + live_other_Hs_separations[Gender] - "2nd_Plus_attack"[Gender] - die_with_CVD_HS[Gender] - die_with_CVD_from_other_circulatory[Gender] - other_HS_hospitalisations[Gender]) * dt {NON-NEGATIVE}

INIT People_with_Chronic_HS[Gender] = "Initial_HS_CVD_M\\F"

INFLOWS:

survive_first_year[Gender] = CONVEYOR OUTFLOW

OUTFLOW PRIORITY: 1

survive_subsequent_first_year[Gender] = CONVEYOR OUTFLOW

OUTFLOW PRIORITY: 1

live_other_Hs_separations[Gender] = CONVEYOR OUTFLOW

OUTFLOWS:

"2nd_Plus_attack"[Gender] = People_with_Chronic_HS*Prob_subsequent_attacks*Combined_Secondary_RR_HS {UNIFLOW}

INFLOW PRIORITY: 3

OUTFLOW PRIORITY: 1

die_with_CVD_HS[Gender] = People_with_Chronic_HS*CDR_with_CVD/1000 {UNIFLOW}

OUTFLOW PRIORITY: 2

die_with_CVD_from_other_circulatory[Gender] = People_with_Chronic_HS*Die_other_CVD_with_prior_HS_CDR/1000*Combined_Secondary_RR_HS {UNIFLOW}

OUTFLOW PRIORITY: 3

other_HS_hospitalisations[Gender] = People_with_Chronic_HS*Other_heart_hospitalisations_%_pa/100 {UNIFLOW}

OUTFLOW PRIORITY: 4

People_with_Chronic_non_HS_CVD[Gender](t) = People_with_Chronic_non_HS_CVD[Gender](t - dt) + (live_discharge_other_CVD[Gender] - First_attack_with_Prior_CVD[Gender] - subsequent_CVD_admits[Gender] - die_with_CVD_no_HS[Gender] - die_of_CVD_non_hospitalised[Gender]) * dt {NON-NEGATIVE}

INIT People_with_Chronic_non_HS_CVD[Gender] = "Initial_non_HS_CVD_M\\F"

INFLOWS:

live_discharge_other_CVD[Gender] = CONVEYOR OUTFLOW

OUTFLOWS:

First_attack_with_Prior_CVD[Gender] = age_adj_attack_prob*People_with_Chronic_non_HS_CVD*CVD_mpr*Combined_Effect_Primary {UNIFLOW}

INFLOW PRIORITY: 2

OUTFLOW PRIORITY: 1

subsequent_CVD_admits[Gender] = People_with_Chronic_non_HS_CVD*prob_annual_hospital_other_pa/100*Combined_Secondary_RR_non_HS {UNIFLOW}

INFLOW PRIORITY: 2

OUTFLOW PRIORITY: 2

die_with_CVD_no_HS[Gender] = People_with_Chronic_non_HS_CVD*CDR_with_CVD/1000 {UNIFLOW}

OUTFLOW PRIORITY: 3

die_of_CVD_non_hospitalised[Gender] = People_with_Chronic_non_HS_CVD*Die_other_CVD_non_hospitalised_CDR/100 {UNIFLOW}

OUTFLOW PRIORITY: 4

Pop_40_Plus[Gender](t) = Pop_40_Plus[Gender](t - dt) + (t40[Gender] + net_migration[Gender] - deaths_40p[Gender]) * dt {NON-NEGATIVE}

INIT Pop_40_Plus[Gender] = INIT(Aus.Pop_40_Plus)

INFLOWS:

t40[Gender] = Aus.Turning_40 {UNIFLOW}

net_migration[Gender] = Aus."Migrants_40P_M\\F" {UNIFLOW}

OUTFLOWS:

deaths_40p[Gender] = Aus.Deaths_40 {UNIFLOW}

Pre_hospital[Gender](t) = Pre_hospital[Gender](t - dt) + (First_Attack_no_prior_cvd[Gender] + First_attack_with_Prior_CVD[Gender] - into_ED[Gender] - die_on_way_to_hospital[Gender]) * dt {CONVEYOR}

INIT Pre_hospital[Gender] = 0

TRANSIT TIME = DT

CONTINUOUS

ACCEPT MULTIPLE BATCHES

INFLOWS:

First_Attack_no_prior_cvd[Gender] = no_CVD*age_adj_attack_prob*Combined_Effect_Primary {UNIFLOW}

INFLOW PRIORITY: 1

OUTFLOW PRIORITY: 3

First_attack_with_Prior_CVD[Gender] = age_adj_attack_prob*People_with_Chronic_non_HS_CVD*CVD_mpr*Combined_Effect_Primary {UNIFLOW}

INFLOW PRIORITY: 2

OUTFLOW PRIORITY: 1

OUTFLOWS:

into_ED[Gender] = CONVEYOR OUTFLOW

die_on_way_to_hospital[Gender] = LEAKAGE OUTFLOW

LEAKAGE FRACTION = adj_pre_hospital_fatality_rate_%/100

LINEAR LEAKAGE

LEAK ZONE = 0% to 100%

Subsequent_First_Year[Gender](t) = Subsequent_First_Year[Gender](t - dt) + (initial_survive_subsequent_attack[Gender] - survive_subsequent_first_year[Gender] - other_deaths_subsequent_first_year[Gender] - attack_subsequent_first_year[Gender] - die_other_CVD_in_subsequent_first_year[Gender]) * dt {CONVEYOR}

INIT Subsequent_First_Year[Gender] = 0

TRANSIT TIME = 1

CONTINUOUS

ACCEPT MULTIPLE BATCHES

INFLOWS:

initial_survive_subsequent_attack[Gender] = CONVEYOR OUTFLOW

OUTFLOWS:

survive_subsequent_first_year[Gender] = CONVEYOR OUTFLOW

OUTFLOW PRIORITY: 1

other_deaths_subsequent_first_year[Gender] = LEAKAGE OUTFLOW

LEAKAGE FRACTION = first_year_other_CVD_death_%/100*Subsequent_attack_fatality_mpr

LINEAR LEAKAGE

LEAK ZONE = 0% to 100%

OUTFLOW PRIORITY: 2

attack_subsequent_first_year[Gender] = LEAKAGE OUTFLOW

LEAKAGE FRACTION = Prob_attack_in_first_year_%/100*prior_attack_mpr*Combined_Secondary_RR_HS

LINEAR LEAKAGE

LEAK ZONE = 0% to 100%

INFLOW PRIORITY: 2

OUTFLOW PRIORITY: 3

die_other_CVD_in_subsequent_first_year[Gender] = LEAKAGE OUTFLOW

LEAKAGE FRACTION = first_year_other_CVD_death_%/100*Subsequent_attack_fatality_mpr

LINEAR LEAKAGE

LEAK ZONE = 0% to 100%

OUTFLOW PRIORITY: 4

Subsequent_Hosp[Gender](t) = Subsequent_Hosp[Gender](t - dt) + (admit_3[Gender] - initial_survive_subsequent_attack[Gender] - Susequent_Attack_Hospital_deaths[Gender]) * dt {CONVEYOR}

INIT Subsequent_Hosp[Gender] = 0

TRANSIT TIME = 30/365

CONTINUOUS

ACCEPT MULTIPLE BATCHES

INFLOWS:

admit_3[Gender] = CONVEYOR OUTFLOW

OUTFLOWS:

initial_survive_subsequent_attack[Gender] = CONVEYOR OUTFLOW

Susequent_Attack_Hospital_deaths[Gender] = LEAKAGE OUTFLOW

LEAKAGE FRACTION = Hospital_Fatality_rate_%/100*Subsequent_attack_fatality_mpr*Reduce_Pre_hospital_delay.Death_%_reduction_mpr*Age_Specific_Mpr.Hospital_Death_Rate_MPR

LINEAR LEAKAGE

LEAK ZONE = 0% to 100%

"%_cvd_deaths_of_all_deaths" = All_CVD_deaths/All_Deaths_40P_am

"%_other_CVD_deaths" = Other_circulatory_deaths_total/All_CVD_deaths*100

"%_pre_hosp_deaths/total_deaths" = "Pre-hospital_deaths_total"/All_CVD_deaths*100

"%_prevalence_all_CVD_40+" = (Prevalence_of_CVD_40P/"pop_40+")*100

"%_prevalence_CVD" = Prevalence_of_CVD_40P/"pop_40+"*100

"%_prevalence_HS_40+" = (People_with_previous_HS/"pop_40+")*100

"%_prevalence_non_HS_CVD_40+" = (People_with_prevalent_non_HS_CVD/"pop_40+")*100

Adj_fatality_rate[Gender] = base_pre_hosp_Fatality_Rate_%

adj_pre_hospital_fatality_rate_%[Gender] = base_pre_hosp_Fatality_Rate_%*Out_of_Hospital_Cardiac_Arrest.Combined_Impact_on_Pre_Ambulance_Deaths

age_adj_attack_prob[Gender] = Base_probability_of_first_attack/100*Generic_Intervention.HS_attack_Modifier*Age_Specific_Mpr.age_weighted_risk_mpr

AIHW_CVD_deaths = GRAPH(TIME)

Points: (2012.000, 44038.0), (2013.000, 43615.0), (2014.000, 45069.0), (2015.000, 45605.0), (2016.000, 43963.0), (2017.000, 43477.0)

AIHW_CVD_seps = GRAPH(TIME)

Points: (2012.500, 518264.0), (2013.500, 480548.0), (2014.500, 490092.0), (2015.500, 556638.0), (2016.500, 576515.0)

AIHW_HS_seps = GRAPH(TIME)

Points: (2012.500, 97391.0), (2013.500, 98147.0), (2014.500, 99359.0), (2015.500, 127336.0), (2016.500, 131487.0)

All_Circ[Gender] = "H\\S_5year"+other_Circ_5_year

All_circ_Deaths_pa = SUM(All_Circ_Deaths_pa_MF)

All_Circ_Deaths_pa_MF[Gender] = HS_deaths+Other_Circ_diseases_deaths

All_CVD_admits = Total_HS_Admits + Total_other_CVD_admits {SUMMING CONVERTER}

All_CVD_deaths = SUM(All_CVD_deaths_by_attack_model[*]) {SUMMING CONVERTER}

all_cvd_deaths_5_years = SUM("H\\S_5year"[*]) + SUM(other_Circ_5_year[*]) {SUMMING CONVERTER}

All_CVD_deaths_by_attack_model[Gender] = Other_Circ_diseases_deaths+HS_deaths

All_Deaths_40P_am = SUM(All_Deaths_40P_mf)

All_Deaths_40P_mf[Gender] = All_CVD_deaths_by_attack_model+non_cvd_deaths

All_Deaths_last_5_years = SUM("All_Deaths_last_5_years_M\\F"[*])

"All_Deaths_last_5_years_M\\F"[Gender] = Non_Circ_Deaths+All_Circ

All_HS_Deaths_pa = SUM(HS_deaths)

All_HS_First_Years[Gender] = Subsequent_First_Year+First_Year

"All_Off_\\On_individual_selector_Sec"[Primary_care] = 1

"All_Off_\\On_individual_selector_Sec"[BP] = 1

"All_Off_\\On_individual_selector_Sec"[Lipids] = 1

"All_Off_\\On_individual_selector_Sec"[Salt] = "Food_Reformulation_-_Salt_Reduction".Salt_RR_Mpr

"All_Off_\\On_individual_selector_Sec"[Exercise] = Physical_Activity.Impact_of_Exercise_on_CVD_risk

"All_Off_\\On_individual_selector_Sec"[Smoking] = Smoking.Smoking_RR

"All_Off_\\On_individual_selector_Sec"[Rehab] = 1

"All_Off\\On_individual_selector_Primary"[Primary_care] = 1

"All_Off\\On_individual_selector_Primary"[BP] = 1

"All_Off\\On_individual_selector_Primary"[Lipids] = 1

"All_Off\\On_individual_selector_Primary"[Salt] = 1

"All_Off\\On_individual_selector_Primary"[Exercise] = 1

"All_Off\\On_individual_selector_Primary"[Smoking] = Smoking.Smoking_RR

"All_Off\\On_individual_selector_Primary"[Rehab] = 1

All_Other_CVD_first_hospitalisations_%_ap[M] = 1

All_Other_CVD_first_hospitalisations_%_ap[F] = 1.2

All_persons_40P = SUM(Aus.Persons_in_stock_cohort[*, A44:A104])

all_post_first_HS_cvd_deaths = SUM(post_first_HS_CVD_deaths)

"All_Selected_Post_Attack_Off\\On" = 1

"All_Selected_Pre_Attack_Off\\On" = 1

Aus_Pop[Age, Gender] = Aus.Persons_in_stock_cohort[Gender,Age]

"Ave_CVD_Prev_by_attack_model_40+" = Prevalence_of_CVD_40P/SUM(Pop_40_Plus)*100

Ave_CVD_Prev_GBD = CVD_Burden_of_Disease.People_with_CVD/SUM(Pop_40_Plus)*100

Ave_HS_NHS_HS_Prevalence_40P = HS_people_40P/All_persons_40P*100

"Ave_HS_prevalence_National_Health_Survey_40-74" = "HS_people_40-74"/SUM("Persons_40-74")*100

ave_Los_post_CVD = SUM(Prevalence_of_HS_MF)/all_post_first_HS_cvd_deaths

Ave_Primary_BP_risk_reduction = HyperTension.total_no_prior_RR_pts/No_Prior_CVD

Ave_Primary_LDL_risk_reduction = Lipids.total_no_prior_RR_pts/No_Prior_CVD

Ave_sec_BP_risk_reduction = HyperTension.Total_prior_RRpts/Prevalence_of_CVD_40P

Ave_sec_LDL_risk_reduction_1 = Lipids.Total_prior_RRpts/Prevalence_of_CVD_40P

base_pre_hosp_Fatality_Rate_%[Gender] = 12.5

Base_probability_of_first_attack[M] = 1

Base_probability_of_first_attack[F] = 0.96

BAU_HS_Prevalence_GBD_40p = Heart_Disease_Types_1.CBV_&_IHD/All_persons_40P*100

CDR_all_other[M] = GRAPH(CDR_input)

Points: (2011.50, 10.79), (2051.50, 10.90)

CDR_all_other[F] = GRAPH(CDR_input)

Points: (2011.50, 9.12), (2051.50, 9.40)

CDR_input = TIME

CDR_other_CVD[Gender] = 1

CDR_with_CVD[Gender] = CDR_all_other*1.1

change_in_primary_ldl_pts_pp = Ave_Primary_LDL_risk_reduction-INIT(Ave_Primary_LDL_risk_reduction)

change_in_primary_risk_pts_per_person = Ave_Primary_BP_risk_reduction-INIT(Ave_Primary_BP_risk_reduction)

change_in_sec_risk_pts_per_person = Ave_sec_BP_risk_reduction-INIT(Ave_sec_BP_risk_reduction)

change_in_sec_risk_pts_per_person_ldl = Ave_sec_LDL_risk_reduction_1-INIT(Ave_sec_LDL_risk_reduction_1)

Combined_Effect_Primary = PROD(Primary_Reduction_by_Intervention_a)

Combined_HS_prev[Age_cvd] = Heart_CVD+Stroke_CVD

Combined_Prev_75P = Heart_75P+Stroke_75P

Combined_Secondary_RR_HS = PROD(Secondary_Reduction_by_Intervention_a)

Combined_Secondary_RR_non_HS = PROD(Secondary_RR_Non_HS)

comp_indicator = IF My_runcount<2 OR TIME<STOPTIME THEN 0 ELSE 1

Comparison_start_time = 2020

Converter_36 = Prevalence_of_CVD_40P+No_Prior_CVD

cumulative_CVD_attacks[comp_period] = Cumulative_total_attacks_from_2019

cumulative_HS_admits_2019[comp_period] = Cumulative_HS_admits_custom_time_horizons

"cumulative_HS_deaths_2019+"[comp_period] = Cumulative_HS_deaths_from_2019

cumulative_other_cvd_admits_2019[comp_period] = Cumulative_other_cvd_admits_from_2019

cumulative_other_cvd_deaths_2019[comp_period] = Cumulative_other_circulatory_deaths_from_2019

custom_time_horizon[Total_study_period] = 20

custom_time_horizon[One_year] = 1

custom_time_horizon[Three_Years] = 3

custom_time_horizon[Ten_Years] = 10

CVD_mpr = 1.1

CVD_seps = Total_other_CVD_admits*Separations_per_hospital_stay

"death_<30_day_outcomes%"[Gender] = deaths_first_30_days/"total_Attacks_M\\F"*100

death_rate_per_K[Gender] = deaths_40p/Pop_40_Plus*1000

Deaths_40P_%[Gender] = Aus.Deaths_40/Aus.All_Deaths*100

deaths_first_30_days[Gender] = Initial_pre_discharge_deaths+subsequent_pre_disharge_deaths

Deaths_prior_to_reaching_ED[Gender] = die_on_way_to_hospital+Subsequent_Attack_pre_ED_Deaths

Die_other_CVD_non_hospitalised_CDR[M] = 0.1

Die_other_CVD_non_hospitalised_CDR[F] = 0.2

Die_other_CVD_with_prior_HS_CDR = .5

Die_other_first_year_% = 5

Difference_from_cumulative_cvd_attacks_base_case[Total_study_period, Base_Case] = IF TIME < STOPTIME THEN NAN ELSE IF reset = 1 OR My_runcount <1 THEN NAN ELSE cumulative_CVD_attacks[Total_study_period]-result_by_run_cumulative_cvd_attacks[Total_study_period,1]

Difference_from_cumulative_cvd_attacks_base_case[Total_study_period, Previous_Run] = IF TIME < STOPTIME THEN NAN ELSE IF reset = 1 OR My_runcount <1 THEN NAN ELSE cumulative_CVD_attacks[Total_study_period]-result_by_run_cumulative_cvd_attacks[Total_study_period,prevrun]

Difference_from_cumulative_cvd_attacks_base_case[One_year, Base_Case] = IF TIME < STOPTIME THEN NAN ELSE IF reset = 1 OR My_runcount <1 THEN NAN ELSE cumulative_CVD_attacks[One_year]-result_by_run_cumulative_cvd_attacks[One_year,1]

Difference_from_cumulative_cvd_attacks_base_case[One_year, Previous_Run] = IF TIME < STOPTIME THEN NAN ELSE IF reset = 1 OR My_runcount <1 THEN NAN ELSE cumulative_CVD_attacks[One_year]-result_by_run_cumulative_cvd_attacks[One_year,prevrun]

Difference_from_cumulative_cvd_attacks_base_case[Three_Years, Base_Case] = IF TIME < STOPTIME THEN NAN ELSE IF reset = 1 OR My_runcount <1 THEN NAN ELSE cumulative_CVD_attacks[Three_Years]-result_by_run_cumulative_cvd_attacks[Three_Years,1]

Difference_from_cumulative_cvd_attacks_base_case[Three_Years, Previous_Run] = IF TIME < STOPTIME THEN NAN ELSE IF reset = 1 OR My_runcount <1 THEN NAN ELSE cumulative_CVD_attacks[Three_Years]-result_by_run_cumulative_cvd_attacks[Three_Years,prevrun]

Difference_from_cumulative_cvd_attacks_base_case[Ten_Years, Base_Case] = IF TIME < STOPTIME THEN NAN ELSE IF reset = 1 OR My_runcount <1 THEN NAN ELSE cumulative_CVD_attacks[Ten_Years]-result_by_run_cumulative_cvd_attacks[Ten_Years,1]

Difference_from_cumulative_cvd_attacks_base_case[Ten_Years, Previous_Run] = IF TIME < STOPTIME THEN NAN ELSE IF reset = 1 OR My_runcount <1 THEN NAN ELSE cumulative_CVD_attacks[Ten_Years]-result_by_run_cumulative_cvd_attacks[Ten_Years,prevrun]

Difference_from_HS_admits_cumulative_base_case[Total_study_period, Base_Case] = IF TIME < STOPTIME THEN NAN ELSE IF reset = 1 OR My_runcount <1 THEN NAN ELSE cumulative_HS_admits_2019[Total_study_period]-result_by_run_HS_admits_cumulative[Total_study_period,1]

Difference_from_HS_admits_cumulative_base_case[Total_study_period, Previous_Run] = IF TIME < STOPTIME THEN NAN ELSE IF reset = 1 OR My_runcount <1 THEN NAN ELSE cumulative_HS_admits_2019[Total_study_period]-result_by_run_HS_admits_cumulative[Total_study_period,prevrun]

Difference_from_HS_admits_cumulative_base_case[One_year, Base_Case] = IF TIME < STOPTIME THEN NAN ELSE IF reset = 1 OR My_runcount <1 THEN NAN ELSE cumulative_HS_admits_2019[One_year]-result_by_run_HS_admits_cumulative[One_year,1]

Difference_from_HS_admits_cumulative_base_case[One_year, Previous_Run] = IF TIME < STOPTIME THEN NAN ELSE IF reset = 1 OR My_runcount <1 THEN NAN ELSE cumulative_HS_admits_2019[One_year]-result_by_run_HS_admits_cumulative[One_year,prevrun]

Difference_from_HS_admits_cumulative_base_case[Three_Years, Base_Case] = IF TIME < STOPTIME THEN NAN ELSE IF reset = 1 OR My_runcount <1 THEN NAN ELSE cumulative_HS_admits_2019[Three_Years]-result_by_run_HS_admits_cumulative[Three_Years,1]

Difference_from_HS_admits_cumulative_base_case[Three_Years, Previous_Run] = IF TIME < STOPTIME THEN NAN ELSE IF reset = 1 OR My_runcount <1 THEN NAN ELSE cumulative_HS_admits_2019[Three_Years]-result_by_run_HS_admits_cumulative[Three_Years,prevrun]

Difference_from_HS_admits_cumulative_base_case[Ten_Years, Base_Case] = IF TIME < STOPTIME THEN NAN ELSE IF reset = 1 OR My_runcount <1 THEN NAN ELSE cumulative_HS_admits_2019[Ten_Years]-result_by_run_HS_admits_cumulative[Ten_Years,1]

Difference_from_HS_admits_cumulative_base_case[Ten_Years, Previous_Run] = IF TIME < STOPTIME THEN NAN ELSE IF reset = 1 OR My_runcount <1 THEN NAN ELSE cumulative_HS_admits_2019[Ten_Years]-result_by_run_HS_admits_cumulative[Ten_Years,prevrun]

Difference_from_HS_deaths_cumulative_base_case[Total_study_period, Base_Case] = IF TIME < STOPTIME THEN NAN ELSE IF reset = 1 OR My_runcount <1 THEN NAN ELSE "cumulative_HS_deaths_2019+"[Total_study_period]-result_by_run_HS_deaths_cumulative[Total_study_period,1]

Difference_from_HS_deaths_cumulative_base_case[Total_study_period, Previous_Run] = IF TIME < STOPTIME THEN NAN ELSE IF reset = 1 OR My_runcount <1 THEN NAN ELSE "cumulative_HS_deaths_2019+"[Total_study_period]-result_by_run_HS_deaths_cumulative[Total_study_period,prevrun]

Difference_from_HS_deaths_cumulative_base_case[One_year, Base_Case] = IF TIME < STOPTIME THEN NAN ELSE IF reset = 1 OR My_runcount <1 THEN NAN ELSE "cumulative_HS_deaths_2019+"[One_year]-result_by_run_HS_deaths_cumulative[One_year,1]

Difference_from_HS_deaths_cumulative_base_case[One_year, Previous_Run] = IF TIME < STOPTIME THEN NAN ELSE IF reset = 1 OR My_runcount <1 THEN NAN ELSE "cumulative_HS_deaths_2019+"[One_year]-result_by_run_HS_deaths_cumulative[One_year,prevrun]

Difference_from_HS_deaths_cumulative_base_case[Three_Years, Base_Case] = IF TIME < STOPTIME THEN NAN ELSE IF reset = 1 OR My_runcount <1 THEN NAN ELSE "cumulative_HS_deaths_2019+"[Three_Years]-result_by_run_HS_deaths_cumulative[Three_Years,1]

Difference_from_HS_deaths_cumulative_base_case[Three_Years, Previous_Run] = IF TIME < STOPTIME THEN NAN ELSE IF reset = 1 OR My_runcount <1 THEN NAN ELSE "cumulative_HS_deaths_2019+"[Three_Years]-result_by_run_HS_deaths_cumulative[Three_Years,prevrun]

Difference_from_HS_deaths_cumulative_base_case[Ten_Years, Base_Case] = IF TIME < STOPTIME THEN NAN ELSE IF reset = 1 OR My_runcount <1 THEN NAN ELSE "cumulative_HS_deaths_2019+"[Ten_Years]-result_by_run_HS_deaths_cumulative[Ten_Years,1]

Difference_from_HS_deaths_cumulative_base_case[Ten_Years, Previous_Run] = IF TIME < STOPTIME THEN NAN ELSE IF reset = 1 OR My_runcount <1 THEN NAN ELSE "cumulative_HS_deaths_2019+"[Ten_Years]-result_by_run_HS_deaths_cumulative[Ten_Years,prevrun]

Difference_from_HS_deaths_pa_base_case = IF TIME < STOPTIME THEN NAN ELSE IF reset= 1 OR My_runcount <1 THEN NAN ELSE All_HS_Deaths_pa-result_by_run_HS_deaths_pa[1]

Difference_from_other_CVD_admits_cumulative_base_case[Total_study_period, Base_Case] = IF TIME < STOPTIME THEN NAN ELSE IF reset = 1 OR My_runcount <1 THEN NAN ELSE cumulative_other_cvd_admits_2019[Total_study_period]-result_by_run_other_CVD_admits_cumulative[Total_study_period,1]

Difference_from_other_CVD_admits_cumulative_base_case[Total_study_period, Previous_Run] = IF TIME < STOPTIME THEN NAN ELSE IF reset = 1 OR My_runcount <1 THEN NAN ELSE cumulative_other_cvd_admits_2019[Total_study_period]-result_by_run_other_CVD_admits_cumulative[Total_study_period,prevrun]

Difference_from_other_CVD_admits_cumulative_base_case[One_year, Base_Case] = IF TIME < STOPTIME THEN NAN ELSE IF reset = 1 OR My_runcount <1 THEN NAN ELSE cumulative_other_cvd_admits_2019[One_year]-result_by_run_other_CVD_admits_cumulative[One_year,1]

Difference_from_other_CVD_admits_cumulative_base_case[One_year, Previous_Run] = IF TIME < STOPTIME THEN NAN ELSE IF reset = 1 OR My_runcount <1 THEN NAN ELSE cumulative_other_cvd_admits_2019[One_year]-result_by_run_other_CVD_admits_cumulative[One_year,prevrun]

Difference_from_other_CVD_admits_cumulative_base_case[Three_Years, Base_Case] = IF TIME < STOPTIME THEN NAN ELSE IF reset = 1 OR My_runcount <1 THEN NAN ELSE cumulative_other_cvd_admits_2019[Three_Years]-result_by_run_other_CVD_admits_cumulative[Three_Years,1]

Difference_from_other_CVD_admits_cumulative_base_case[Three_Years, Previous_Run] = IF TIME < STOPTIME THEN NAN ELSE IF reset = 1 OR My_runcount <1 THEN NAN ELSE cumulative_other_cvd_admits_2019[Three_Years]-result_by_run_other_CVD_admits_cumulative[Three_Years,prevrun]

Difference_from_other_CVD_admits_cumulative_base_case[Ten_Years, Base_Case] = IF TIME < STOPTIME THEN NAN ELSE IF reset = 1 OR My_runcount <1 THEN NAN ELSE cumulative_other_cvd_admits_2019[Ten_Years]-result_by_run_other_CVD_admits_cumulative[Ten_Years,1]

Difference_from_other_CVD_admits_cumulative_base_case[Ten_Years, Previous_Run] = IF TIME < STOPTIME THEN NAN ELSE IF reset = 1 OR My_runcount <1 THEN NAN ELSE cumulative_other_cvd_admits_2019[Ten_Years]-result_by_run_other_CVD_admits_cumulative[Ten_Years,prevrun]

Difference_from_other_cvd_deaths_cumulative_base_case[Total_study_period, Base_Case] = IF TIME < STOPTIME THEN NAN ELSE IF reset = 1 OR My_runcount <1 THEN NAN ELSE cumulative_other_cvd_deaths_2019[Total_study_period]-result_by_run_other_cvd_deaths_cumulative[Total_study_period,1]

Difference_from_other_cvd_deaths_cumulative_base_case[Total_study_period, Previous_Run] = IF TIME < STOPTIME THEN NAN ELSE IF reset = 1 OR My_runcount <1 THEN NAN ELSE cumulative_other_cvd_deaths_2019[Total_study_period]-result_by_run_other_cvd_deaths_cumulative[Total_study_period,prevrun]

Difference_from_other_cvd_deaths_cumulative_base_case[One_year, Base_Case] = IF TIME < STOPTIME THEN NAN ELSE IF reset = 1 OR My_runcount <1 THEN NAN ELSE cumulative_other_cvd_deaths_2019[One_year]-result_by_run_other_cvd_deaths_cumulative[One_year,1]

Difference_from_other_cvd_deaths_cumulative_base_case[One_year, Previous_Run] = IF TIME < STOPTIME THEN NAN ELSE IF reset = 1 OR My_runcount <1 THEN NAN ELSE cumulative_other_cvd_deaths_2019[One_year]-result_by_run_other_cvd_deaths_cumulative[One_year,prevrun]

Difference_from_other_cvd_deaths_cumulative_base_case[Three_Years, Base_Case] = IF TIME < STOPTIME THEN NAN ELSE IF reset = 1 OR My_runcount <1 THEN NAN ELSE cumulative_other_cvd_deaths_2019[Three_Years]-result_by_run_other_cvd_deaths_cumulative[Three_Years,1]

Difference_from_other_cvd_deaths_cumulative_base_case[Three_Years, Previous_Run] = IF TIME < STOPTIME THEN NAN ELSE IF reset = 1 OR My_runcount <1 THEN NAN ELSE cumulative_other_cvd_deaths_2019[Three_Years]-result_by_run_other_cvd_deaths_cumulative[Three_Years,prevrun]

Difference_from_other_cvd_deaths_cumulative_base_case[Ten_Years, Base_Case] = IF TIME < STOPTIME THEN NAN ELSE IF reset = 1 OR My_runcount <1 THEN NAN ELSE cumulative_other_cvd_deaths_2019[Ten_Years]-result_by_run_other_cvd_deaths_cumulative[Ten_Years,1]

Difference_from_other_cvd_deaths_cumulative_base_case[Ten_Years, Previous_Run] = IF TIME < STOPTIME THEN NAN ELSE IF reset = 1 OR My_runcount <1 THEN NAN ELSE cumulative_other_cvd_deaths_2019[Ten_Years]-result_by_run_other_cvd_deaths_cumulative[Ten_Years,prevrun]

ED_deaths[Gender] = First_Attack_ED_deaths+Subsequent_attack_ED_2_deaths

ED_deaths_% = 3

ED_deaths_%_admitted = 25

EDdead_admits[Gender] = ED_deaths*ED_deaths_%_admitted/100

Effective_%_script_fillers = Lipids.effective_takers_lipids/SUM(Pop_40_Plus)*100

endtime = TIME

"Females_40_-74"[ae44] = Aus.Persons_in_stock_cohort[F,A44]

"Females_40_-74"[ae49] = Aus.Persons_in_stock_cohort[F,A49]

"Females_40_-74"[ae54] = Aus.Persons_in_stock_cohort[F,A54]

"Females_40_-74"[ae59] = Aus.Persons_in_stock_cohort[F,A59]

"Females_40_-74"[ae64] = Aus.Persons_in_stock_cohort[F,A64]

"Females_40_-74"[ae69] = Aus.Persons_in_stock_cohort[F,A69]

"Females_40_-74"[ae74] = Aus.Persons_in_stock_cohort[F,A74]

first_attack_deaths_%[Gender] = Initial_pre_discharge_deaths/First_Attack_no_prior_cvd*100

First_attacks[Gender] = First_attack_with_Prior_CVD+First_Attack_no_prior_cvd

first_year_other_CVD_death_% = .5

Heart_75P = 15.5

Heart_CVD[ae44] = .3

Heart_CVD[ae49] = 1.2

Heart_CVD[ae54] = 1.2

Heart_CVD[ae59] = 4.1

Heart_CVD[ae64] = 4.1

Heart_CVD[ae69] = 7.6

Heart_CVD[ae74] = 7.6

Hospital_Deaths[Gender] = First_attack_and_other_CVD_hosp_deaths+Susequent_Attack_Hospital_deaths

Hospital_Fatality_rate_% = 2

HS_75P = People_75P*Combined_Prev_75P/100

HS_deaths[Gender] = ED_deaths+Hospital_Deaths+Deaths_prior_to_reaching_ED

HS_pa = SUM(HS_deaths)

"HS_people_40-74" = SUM(HS_people_by_age)

HS_people_40P = HS_75P+"HS_people_40-74"

HS_people_by_age[Age_cvd] = "Persons_40-74"*Combined_HS_prev/100

Init_CVD = INIT(CVD_Burden_of_Disease.People_with_CVD)-INIT(Heart_Disease_Types_1.CBV_&_IHD)

"Initial_HS_CVD_M\\F"[M] = INIT(Heart_Disease_Types_1.CBV_&_IHD)*M%_CVD/100

"Initial_HS_CVD_M\\F"[F] = INIT(Heart_Disease_Types_1.CBV_&_IHD)*(1-M%_CVD/100)

"Initial_non_HS_CVD_M\\F"[M] = Init_CVD*M%_CVD/100

"Initial_non_HS_CVD_M\\F"[F] = Init_CVD*(100-M%_CVD)/100

Initial_pre_discharge_deaths[Gender] = First_Attack_ED_deaths+die_on_way_to_hospital+First_attack_and_other_CVD_hosp_deaths

Intervention_Impact[Primary_care] = 1

Intervention_Impact[BP] = Primary_BP_risk_mpr

Intervention_Impact[Lipids] = Primary_LDL_risk_Mpr

Intervention_Impact[Salt] = "Food_Reformulation_-_Salt_Reduction".Salt_RR_Mpr

Intervention_Impact[Exercise] = Physical_Activity.Impact_of_Exercise_on_CVD_risk

Intervention_Impact[Smoking] = Smoking.Smoking_RR

Intervention_Impact[Rehab] = 1

"Intervention_Off\\On"[Primary_care] = 1

"Intervention_Off\\On"[BP] = IF GPs."BP_adherence_Off\\On" = 1 OR GPs."BP_guideline_compliance_off/on" = 1 THEN 1 ELSE 0

"Intervention_Off\\On"[Lipids] = IF GPs."Lipid_guideline_compliance_Off\\On" = 1 OR GPs."Lipid_adherence_off/on" = 1 THEN 1 ELSE 0

"Intervention_Off\\On"[Salt] = "Food_Reformulation_-_Salt_Reduction"."Food_Reform_Off\\on"

"Intervention_Off\\On"[Exercise] = 1

"Intervention_Off\\On"[Smoking] = 1

"Intervention_Off\\On"[Rehab] = Rehab."Rehab_Increase_Off\\On"

Intervention_secondary_Impact_1[Primary_care] = 1

Intervention_secondary_Impact_1[BP] = Secondary_BP_risk_Mpr

Intervention_secondary_Impact_1[Lipids] = Secondary_LDL_risk_Mpr

Intervention_secondary_Impact_1[Salt] = "Food_Reformulation_-_Salt_Reduction".Salt_RR_Mpr

Intervention_secondary_Impact_1[Exercise] = Physical_Activity.Impact_of_Exercise_on_CVD_risk

Intervention_secondary_Impact_1[Smoking] = Smoking.Smoking_RR

Intervention_secondary_Impact_1[Rehab] = Rehab.Rehab_effect_on_subsequent_attack_rates

Intrervention_applies_to_Non_HS[Primary_care] = 1

Intrervention_applies_to_Non_HS[BP] = 1

Intrervention_applies_to_Non_HS[Lipids] = 0

Intrervention_applies_to_Non_HS[Salt] = 1

Intrervention_applies_to_Non_HS[Exercise] = 1

Intrervention_applies_to_Non_HS[Smoking] = 1

Intrervention_applies_to_Non_HS[Rehab] = 0

Live_HS_Separations[Gender] = survive_first_attack+initial_survive_subsequent_attack

Living_with_CVD_HS[Hosp1, M] = Hospital[M]

Living_with_CVD_HS[Hosp1, F] = Hospital[F]

Living_with_CVD_HS[First_year_1, M] = First_Year[M]

Living_with_CVD_HS[First_year_1, F] = First_Year[F]

Living_with_CVD_HS[Lving_with, M] = People_with_Chronic_HS[M]

Living_with_CVD_HS[Lving_with, F] = People_with_Chronic_HS[F]

Living_with_CVD_HS[sub_pre_hosp, M] = Acute_Subsequent_pre_hospital[M]+ED_Dept_2[M]

Living_with_CVD_HS[sub_pre_hosp, F] = Acute_Subsequent_pre_hospital[F]+ED_Dept_2[F]

Living_with_CVD_HS[hosp2, M] = Subsequent_Hosp[M]

Living_with_CVD_HS[hosp2, F] = Subsequent_Hosp[F]

Living_with_CVD_HS[First_year_2, M] = Subsequent_First_Year[M]

Living_with_CVD_HS[First_year_2, F] = Subsequent_First_Year[F]

Living_with_non_HS_CVD[Gender] = Other_CVD_hospital+People_with_Chronic_non_HS_CVD

M%_CVD = 53.5

Males_40_74[ae44] = Aus.Persons_in_stock_cohort[M,A44]

Males_40_74[ae49] = Aus.Persons_in_stock_cohort[M,A49]

Males_40_74[ae54] = Aus.Persons_in_stock_cohort[M,A54]

Males_40_74[ae59] = Aus.Persons_in_stock_cohort[M,A59]

Males_40_74[ae64] = Aus.Persons_in_stock_cohort[M,A64]

Males_40_74[ae69] = Aus.Persons_in_stock_cohort[M,A69]

Males_40_74[ae74] = Aus.Persons_in_stock_cohort[M,A74]

My_runcount = IF reset <> 0 THEN 0 ELSE ENDVAL(SELF,-1) + 1

No_Prior_CVD = SUM(no_CVD)

non_cvd_deaths[Gender] = die_no_CVD+die_with_CVD_HS+Non_cvd_deaths_first_year+die_with_CVD_no_HS

Non_cvd_deaths_first_year[Gender] = other_deaths_first_year+other_deaths_subsequent_first_year

Non_CVD_prevalence = No_Prior_CVD/SUM(Pop_40_Plus)

Other_Circ_diseases_deaths[Gender] = die_at_home_other_CVD+die_with_CVD_from_other_circulatory+other_CVD_deaths_first_year+die_other_CVD_in_subsequent_first_year+other_CVD_hospital_deaths+die_of_CVD_non_hospitalised

Other_circulatory_deaths_total = SUM(Other_Circ_diseases_deaths[*]) {SUMMING CONVERTER}

Other_CVD_admits[Gender] = admit_2+subsequent_CVD_admits

Other_heart_hospitalisations_%_pa = 4.5

Other_hospital_CVD_death_rate_% = .75

People_75P = SUM(Persons_75P_by_sex)

People_with_prevalent_non_HS_CVD = SUM(Living_with_non_HS_CVD[*]) {SUMMING CONVERTER}

People_with_previous_HS = SUM(Prevalence_of_HS_MF[*]) {SUMMING CONVERTER}

"Person_40-74_by_sex"[M] = SUM(Males_40_74)

"Person_40-74_by_sex"[F] = SUM("Females_40_-74")

"Persons_40-74"[Age_cvd] = Males_40_74+"Females_40_-74"

Persons_75P_by_sex[Gender] = SUM(Aus.Persons_in_stock_cohort[Gender, A79:A104])

"pop_40+" = SUM(Pop_40_Plus)

Pop_Xcheck[Gender] = no_CVD+ED_Dept+Pre_hospital+SUM(Living_with_CVD_HS[*,Gender])+Living_with_non_HS_CVD

Pop40Plus_%[Gender] = Aus.Pop_40_Plus/Aus.Total_Pop_MF*100

post_first_HS_CVD_deaths[Gender] = Subsequent_Attack_pre_ED_Deaths+die_with_CVD_HS+Subsequent_attack_ED_2_deaths+Susequent_Attack_Hospital_deaths+die_with_CVD_from_other_circulatory+die_other_CVD_in_subsequent_first_year+other_deaths_subsequent_first_year

"Pre-hospital_deaths_total" = SUM(Deaths_prior_to_reaching_ED[*]) {SUMMING CONVERTER}

Prevalence_HS_40P_%[Gender] = Prevalence_of_HS_MF/Pop_40_Plus*100

Prevalence_of_CVD[Gender] = Prevalence_of_HS_MF+Living_with_non_HS_CVD

Prevalence_of_CVD_40P = SUM(Prevalence_of_CVD)

Prevalence_of_HS_MF[Gender] = SUM(Living_with_CVD_HS[*,Gender])

prevrun = IF My_runcount<2 THEN NAN ELSE My_runcount-1

Primary_BP_risk_mpr = 1-change_in_primary_risk_pts_per_person

Primary_Intervention_to_use[Interv] = IF "All_Selected_Pre_Attack_Off\\On"=0 THEN "All_Off\\On_individual_selector_Primary" ELSE Intervention_Impact

Primary_LDL_risk_Mpr = 1-change_in_primary_ldl_pts_pp

Primary_Reduction_by_Intervention_a[Interv] = IF "Intervention_Off\\On"=0 THEN 1 ELSE Primary_Intervention_to_use

prior_attack_mpr = 2.5

prob_annual_hospital_other_pa = 10.5

Prob_attack_in_first_year_% = 10

Prob_subsequent_attacks[Gender] = prior_attack_mpr*age_adj_attack_prob

Relative_increase_in_non_CVD_prev = Non_CVD_prevalence/INIT(Non_CVD_prevalence)

Relative_Increase_in_Prior = "Ave_CVD_Prev_by_attack_model_40+"/ INIT("Ave_CVD_Prev_by_attack_model_40+")

Repeat_attack_% = SUM(Repeat_attacks)/Total_attacks*100

Repeat_attacks[Gender] = First_year_1_attacks+"2nd_Plus_attack"+attack_subsequent_first_year+other_HS_hospitalisations

reset = 0

reset_message_indicator = IF TIME < STOPTIME THEN reset ELSE 0

reset_message_indicator_2 = IF reset=1 AND TIME = STOPTIME THEN 1 ELSE 0

result_by_run_cumulative_cvd_attacks[comp_period, RUN] = IF reset <> 0 THEN 0 ELSE IF My_runcount = RUN THEN cumulative_CVD_attacks[comp_period] ELSE ENDVAL(SELF, 0)

result_by_run_HS_admits_cumulative[comp_period, RUN] = IF reset <> 0 THEN 0 ELSE IF My_runcount = RUN THEN cumulative_HS_admits_2019[comp_period] ELSE ENDVAL(SELF, 0)

result_by_run_HS_deaths_cumulative[comp_period, RUN] = IF reset <> 0 THEN NAN ELSE IF My_runcount = RUN THEN "cumulative_HS_deaths_2019+"[comp_period] ELSE ENDVAL(SELF, 0)

result_by_run_HS_deaths_pa[RUN] = IF reset <> 0 THEN 0 ELSE IF My_runcount = RUN THEN All_HS_Deaths_pa ELSE ENDVAL(SELF, 0)

result_by_run_other_CVD_admits_cumulative[comp_period, RUN] = IF reset <> 0 THEN 0 ELSE IF My_runcount = RUN THEN cumulative_other_cvd_admits_2019[comp_period] ELSE ENDVAL(SELF, 0)

result_by_run_other_cvd_deaths_cumulative[comp_period, RUN] = IF reset <> 0 THEN 0 ELSE IF My_runcount = RUN THEN cumulative_other_cvd_deaths_2019[comp_period] ELSE ENDVAL(SELF, 0)

Sec_Intervention_RR_to_use[Interv] = IF "All_Selected_Post_Attack_Off\\On"=0 THEN "All_Off_\\On_individual_selector_Sec" ELSE Intervention_secondary_Impact_1

Secondary_BP_risk_Mpr = 1-change_in_sec_risk_pts_per_person

Secondary_LDL_risk_Mpr = 1-change_in_sec_risk_pts_per_person_ldl

Secondary_Reduction_by_Intervention_a[Interv] = IF "Intervention_Off\\On"=0 THEN 1 ELSE Sec_Intervention_RR_to_use

Secondary_RR_Non_HS[Interv] = IF Intrervention_applies_to_Non_HS = 1 THEN Secondary_Reduction_by_Intervention_a ELSE 1

Separations_per_hospital_stay = 1.36

Stroke_75P = 2.7

Stroke_CVD[ae44] = 0

Stroke_CVD[ae49] = .2

Stroke_CVD[ae54] = .2

Stroke_CVD[ae59] = .1

Stroke_CVD[ae64] = 0.1

Stroke_CVD[ae69] = 2.2

Stroke_CVD[ae74] = 2.2

Subsequent_attack_fatality_mpr = 2

subsequent_pre_disharge_deaths[Gender] = Subsequent_Attack_pre_ED_Deaths+Subsequent_attack_ED_2_deaths+Susequent_Attack_Hospital_deaths

survive_hospital = IF TIME > 2012 THEN SUM(survive_first_attack)/SUM(into_ED) ELSE 1

Total_attacks = SUM("total_Attacks_M\\F")

"total_Attacks_M\\F"[Gender] = Repeat_attacks+First_attacks

"total_CVD_seps_2016-17" = "other_CVD_Seps_2016-_17"+"HS_Seps_2016-17"

total_CVD_seps_pa = CVD_seps+total_HS_Separations

Total_first_attacks = SUM(First_attacks[*]) {SUMMING CONVERTER}

Total_HS_Admits = SUM(Total_HS_admits_MF)+SUM(EDdead_admits)+SUM(other_HS_hospitalisations)

Total_HS_admits_MF[Gender] = admit_1+admit_3

total_HS_Separations = Total_HS_Admits*Separations_per_hospital_stay

Total_other_CVD_admits = SUM(Other_CVD_admits)

Total_Pop_m = Aus.Total_Pop_MF[M]/1e6

total_repeat_attacks = SUM(Repeat_attacks[*]) {SUMMING CONVERTER}

abs_data:

"2017_abs_5_ears"[Gender](t) = "2017_abs_5_ears"[Gender](t - dt) + (Flow_10[Gender] - Flow_11[Gender]) * dt {CONVEYOR}

INIT "2017_abs_5_ears"[Gender] = 0

TRANSIT TIME = 5

CONTINUOUS

ACCEPT MULTIPLE BATCHES

INFLOWS:

Flow_10[Gender] = "ABS_M\\F" {UNIFLOW}

OUTFLOWS:

Flow_11[Gender] = CONVEYOR OUTFLOW

CVD_deaths_last_5_years[Gender](t) = CVD_deaths_last_5_years[Gender](t - dt) + (Flow_1[Gender] - Flow_2[Gender]) * dt {CONVEYOR}

INIT CVD_deaths_last_5_years[Gender] = 0

TRANSIT TIME = 5

CONTINUOUS

ACCEPT MULTIPLE BATCHES

INFLOWS:

Flow_1[Gender] = deaths_mf {UNIFLOW}

OUTFLOWS:

Flow_2[Gender] = CONVEYOR OUTFLOW

Death_Rate_Mpr[Gender](t) = Death_Rate_Mpr[Gender](t - dt) + ( - delta_drm[Gender]) * dt {NON-NEGATIVE}

INIT Death_Rate_Mpr[Gender] = 1

OUTFLOWS:

delta_drm[Gender] = Death_Rate_Mpr*Ave_annual_Improvement_%_pa/100*"Death_rate_improvement_Off\\On" {UNIFLOW}

"Derate_to_get_2011.5"[M] = .95

"Derate_to_get_2011.5"[F] = .9

ABS_CVD_Deaths_5yr_ag[Gender, Age] = Aus.Persons_in_stock_cohort/100000*CVD_death_rates12*"Derate_to_get_2011.5"[Gender]

ABS_deaths_to_2017[Gender, Age] = Aus.Persons_in_stock_cohort*CVD_death_rates/100000

"ABS_M\\F"[Gender] = SUM(ABS_deaths_to_2017[Gender,*])

"adj_ABS_CVD_deaths_m\\f_by_age"[Gender, Age] = ABS_CVD_Deaths_5yr_ag*Death_Rate_Mpr[Gender]

Ave_annual_Improvement_%_pa[Gender] = 5

Converter_10[Gender, Age] = CVD_death_rates12*Death_Rate_Mpr[Gender]

CVD_death_rates[Gender, Age] = 1

CVD_death_rates12[Gender, Age] = 1

"Death_rate_improvement_Off\\On" = 1

deaths_40_plus[Gender] = SUM("adj_ABS_CVD_deaths_m\\f_by_age"[Gender,A44 :A104])

deaths_mf[Gender] = SUM("adj_ABS_CVD_deaths_m\\f_by_age"[Gender,*])

total_ABS_CVD_Deaths = SUM(deaths_mf)

Age_Specific_Mpr:

"Age_Specific_Hospital_death_rate_modifier_Off\\On" = 1

age_weighted_risk_mpr[Gender] = weighted_CVD_rate/INIT(weighted_CVD_rate)

Base_deaths_per_100K = 22

Converter_3[Age, Gender] = Aus.Persons_in_stock_cohort[Gender,Age]*(death_rate_from_ABS/100000)

Converter_4[Gender] = total_deaths/INIT(total_deaths)

CVD_deaths_40P = SUM(CVD_deaths_40P_MF)

CVD_deaths_40P_MF[Gender] = SUM(CVD_deaths_MF[*,Gender])

CVD_deaths_MF[Age, Gender] = weighted_CVD_rates_a*Base_deaths_per_100K/100000

death_rate_from_ABS[A4, M] = 0

death_rate_from_ABS[A4, F] = 0

death_rate_from_ABS[A9, M] = 0

death_rate_from_ABS[A9, F] = 0

death_rate_from_ABS[A14, M] = 0

death_rate_from_ABS[A14, F] = 0

death_rate_from_ABS[A19, M] = 0

death_rate_from_ABS[A19, F] = 0

death_rate_from_ABS[A24, M] = 0

death_rate_from_ABS[A24, F] = 0

death_rate_from_ABS[A29, M] = 0

death_rate_from_ABS[A29, F] = 0

death_rate_from_ABS[A34, M] = 0

death_rate_from_ABS[A34, F] = 0

death_rate_from_ABS[A39, M] = 0

death_rate_from_ABS[A39, F] = 0

death_rate_from_ABS[A44, M] = 22

death_rate_from_ABS[A44, F] = 0

death_rate_from_ABS[A49, M] = 63

death_rate_from_ABS[A49, F] = 0

death_rate_from_ABS[A54, M] = 63

death_rate_from_ABS[A54, F] = 0

death_rate_from_ABS[A59, M] = 141

death_rate_from_ABS[A59, F] = 0

death_rate_from_ABS[A64, M] = 141

death_rate_from_ABS[A64, F] = 0

death_rate_from_ABS[A69, M] = 350

death_rate_from_ABS[A69, F] = 0

death_rate_from_ABS[A74, M] = 350

death_rate_from_ABS[A74, F] = 0

death_rate_from_ABS[A79, M] = 1237

death_rate_from_ABS[A79, F] = 0

death_rate_from_ABS[A84, M] = 1237

death_rate_from_ABS[A84, F] = 0

death_rate_from_ABS[A89, M] = 5241

death_rate_from_ABS[A89, F] = 0

death_rate_from_ABS[A94, M] = 5241

death_rate_from_ABS[A94, F] = 0

death_rate_from_ABS[A99, M] = 5241

death_rate_from_ABS[A99, F] = 0

death_rate_from_ABS[A104, M] = 5241

death_rate_from_ABS[A104, F] = 0

Dummy = 1

Hospital_Death_Rate_MPR[Gender] = IF "Age_Specific_Hospital_death_rate_modifier_Off\\On"=1 THEN Total_WHDR/INIT(Total_WHDR) ELSE 1

Median_Age[Age] = Age*5-2.5

"persons_40+"[Gender] = SUM(Aus.Persons_in_stock_cohort[Gender, A44:A104])

Premature_death_%[Gender] = weighted_cvd_risk_40_to_75/Total_Weighted_Risk_a*100

Proportion_of_attacks_by_age[Gender, Age] = weighted_CVD_rates_a[Age,Gender]/Weighted_Total[Gender]

Relative_CVD_rates[A4, M] = 0

Relative_CVD_rates[A4, F] = 0

Relative_CVD_rates[A9, M] = 0

Relative_CVD_rates[A9, F] = 0

Relative_CVD_rates[A14, M] = 0

Relative_CVD_rates[A14, F] = 0

Relative_CVD_rates[A19, M] = 0

Relative_CVD_rates[A19, F] = 0

Relative_CVD_rates[A24, M] = 0

Relative_CVD_rates[A24, F] = 0

Relative_CVD_rates[A29, M] = 0

Relative_CVD_rates[A29, F] = 0

Relative_CVD_rates[A34, M] = 0

Relative_CVD_rates[A34, F] = 0

Relative_CVD_rates[A39, M] = 0

Relative_CVD_rates[A39, F] = 0

Relative_CVD_rates[A44, M] = 1

Relative_CVD_rates[A44, F] = .4

Relative_CVD_rates[A49, M] = 2.9

Relative_CVD_rates[A49, F] = 1

Relative_CVD_rates[A54, M] = 2.9

Relative_CVD_rates[A54, F] = 1

Relative_CVD_rates[A59, M] = 6.4

Relative_CVD_rates[A59, F] = 2.4

Relative_CVD_rates[A64, M] = 6.4

Relative_CVD_rates[A64, F] = 2.4

Relative_CVD_rates[A69, M] = 16

Relative_CVD_rates[A69, F] = 7.6

Relative_CVD_rates[A74, M] = 16

Relative_CVD_rates[A74, F] = 7.6

Relative_CVD_rates[A79, M] = 56.6

Relative_CVD_rates[A79, F] = 39.4

Relative_CVD_rates[A84, M] = 56.6

Relative_CVD_rates[A84, F] = 39.4

Relative_CVD_rates[A89, M] = 239.8

Relative_CVD_rates[A89, F] = 238.8

Relative_CVD_rates[A94, M] = 239.8

Relative_CVD_rates[A94, F] = 238.8

Relative_CVD_rates[A99, M] = 239.8

Relative_CVD_rates[A99, F] = 238.8

Relative_CVD_rates[A104, M] = 239.8

Relative_CVD_rates[A104, F] = 238.8

Relative_Hospital_Mortality_Rates[Age] = LOOKUP(Relative_hospital_mortality_with_age, Median_Age)

Relative_hospital_mortality_with_age = GRAPH(Dummy)

Points: (40.00, 1.000), (45.00, 1.000), (50.00, 1.096), (55.00, 1.184), (60.00, 1.272), (65.00, 1.382), (70.00, 1.579), (75.00, 1.820), (80.00, 2.522), (85.00, 3.969), (90.00, 5.000)

total_deaths[Gender] = SUM(Converter_3)/"persons_40+"

Total_Weighted_Risk_a[Gender] = SUM(weighted_CVD_rates_a[*,Gender])

Total_WHDR[Gender] = SUM(Weighted_Hospital_death_Rates[Gender,*])

weighted_CVD_rate[Gender] = SUM(weighted_CVD_rates_a)/"persons_40+"

weighted_CVD_rates_40_to_65[Gender] = SUM(weighted_CVD_rates_a[A44:A64, Gender])

weighted_CVD_rates_a[Age, Gender] = Aus.Persons_in_stock_cohort[Gender,Age]*(Relative_CVD_rates)

weighted_cvd_risk_40_to_75[Gender] = SUM(weighted_CVD_rates_a[A44:A74, Gender])

Weighted_Hospital_death_Rates[Gender, Age] = Proportion_of_attacks_by_age*Relative_Hospital_Mortality_Rates[Age]

Weighted_Total[Gender] = SUM(weighted_CVD_rates_a[*,Gender])

Worker_%_of_attacks[Gender] = weighted_CVD_rates_40_to_65/Total_Weighted_Risk_a *100

Atrial_Fibrillation:

"Cases_2014.5"(t) = "Cases_2014.5"(t - dt) + (Flow_2) * dt {NON-NEGATIVE}

INIT "Cases_2014.5" = 0

INFLOWS:

Flow_2 = IF TIME =2014.5 THEN All_Cases/DT ELSE 0 {UNIFLOW}

"Prevalence_2014.5"(t) = "Prevalence_2014.5"(t - dt) + (Flow_1) * dt {NON-NEGATIVE}

INIT "Prevalence_2014.5" = 0

INFLOWS:

Flow_1 = IF TIME =2014.5 THEN AF_Prev/DT ELSE 0 {UNIFLOW}

AF_Cases[AF_Age, Gender] = AF_Pop_55p*AF_Prev_%/100

"AF_cases_M|F"[Gender] = SUM(AF_Cases[*,Gender])

AF_Pop_55p[af59, M] = Aus.Persons_in_stock_cohort[M,A59]

AF_Pop_55p[af59, F] = Aus.Persons_in_stock_cohort[F,A59]

AF_Pop_55p[af64, M] = Aus.Persons_in_stock_cohort[M,A64]

AF_Pop_55p[af64, F] = Aus.Persons_in_stock_cohort[F,A64]

AF_Pop_55p[af69, M] = Aus.Persons_in_stock_cohort[M,A69]

AF_Pop_55p[af69, F] = Aus.Persons_in_stock_cohort[F,A69]

AF_Pop_55p[af74, M] = Aus.Persons_in_stock_cohort[M,A74]

AF_Pop_55p[af74, F] = Aus.Persons_in_stock_cohort[F,A74]

AF_Pop_55p[af79, M] = Aus.Persons_in_stock_cohort[M,A79]

AF_Pop_55p[af79, F] = Aus.Persons_in_stock_cohort[F,A79]

AF_Pop_55p[af84, M] = Aus.Persons_in_stock_cohort[M,A84]

AF_Pop_55p[af84, F] = Aus.Persons_in_stock_cohort[F,A84]

AF_Pop_55p[af85p, M] = SUM(Aus.Persons_in_stock_cohort[M,A89:A104])

AF_Pop_55p[af85p, F] = SUM(Aus.Persons_in_stock_cohort[F,A89:A104])

AF_Prev = All_Cases/All_Pop_55P*100

AF_Prev_%[af59, M] = .8

AF_Prev_%[af59, F] = .6

AF_Prev_%[af64, M] = 2.6

AF_Prev_%[af64, F] = 1

AF_Prev_%[af69, M] = 5.2

AF_Prev_%[af69, F] = 2.9

AF_Prev_%[af74, M] = 6.9

AF_Prev_%[af74, F] = 5.4

AF_Prev_%[af79, M] = 13.

AF_Prev_%[af79, F] = 6.5

AF_Prev_%[af84, M] = 15.2

AF_Prev_%[af84, F] = 12.7

AF_Prev_%[af85p, M] = 19.7

AF_Prev_%[af85p, F] = 17.5

All_Cases = SUM("AF_cases_M|F")

All_Pop_55P = SUM("Pop_55P_M\\F")

Cases_2014_dif = All_Cases-"Cases_2014.5"

"Cases_55-74" = SUM(AF_Cases[af59:af74,*])

Cases_75Plus = SUM(AF_Cases[af79:af85p, *])

Cases_by_Age[AF_Age] = SUM(AF_Cases[AF_Age,*])

MF_Prev[Gender] = "AF_cases_M|F"/"Pop_55P_M\\F"*100

"Pop_55P_M\\F"[Gender] = SUM(AF_Pop_55p[*,Gender])

Pop55P_Xcheck = SUM(AF_Pop_55p)

Prev_2014_multiple = IF "Prevalence_2014.5">0 THEN AF_Prev/"Prevalence_2014.5" ELSE 1

Aus:

Persons_in_stock_cohort[M, A4](t) = Persons_in_stock_cohort[M, A4](t - dt) + (stock_cohort_ageing_in[M, A4] + Births[M, A4] + Stock_Immigration[M, A4] - stock_cohort_ageing_out[M, A4] - stock_cohort_deaths[M, A4] - stock_Emmigration[M, A4]) * dt

INIT Persons_in_stock_cohort[M, A4] = 748527

UNITS: persons

Persons_in_stock_cohort[M, A9](t) = Persons_in_stock_cohort[M, A9](t - dt) + (stock_cohort_ageing_in[M, A9] + Births[M, A9] + Stock_Immigration[M, A9] - stock_cohort_ageing_out[M, A9] - stock_cohort_deaths[M, A9] - stock_Emmigration[M, A9]) * dt

INIT Persons_in_stock_cohort[M, A9] = 712205

UNITS: persons

Persons_in_stock_cohort[M, A14](t) = Persons_in_stock_cohort[M, A14](t - dt) + (stock_cohort_ageing_in[M, A14] + Births[M, A14] + Stock_Immigration[M, A14] - stock_cohort_ageing_out[M, A14] - stock_cohort_deaths[M, A14] - stock_Emmigration[M, A14]) * dt

INIT Persons_in_stock_cohort[M, A14] = 711543

Persons_in_stock_cohort[M, A19](t) = Persons_in_stock_cohort[M, A19](t - dt) + (stock_cohort_ageing_in[M, A19] + Births[M, A19] + Stock_Immigration[M, A19] - stock_cohort_ageing_out[M, A19] - stock_cohort_deaths[M, A19] - stock_Emmigration[M, A19]) * dt

INIT Persons_in_stock_cohort[M, A19] = 746599

UNITS: persons

Persons_in_stock_cohort[M, A24](t) = Persons_in_stock_cohort[M, A24](t - dt) + (stock_cohort_ageing_in[M, A24] + Births[M, A24] + Stock_Immigration[M, A24] - stock_cohort_ageing_out[M, A24] - stock_cohort_deaths[M, A24] - stock_Emmigration[M, A24]) * dt

INIT Persons_in_stock_cohort[M, A24] = 823470

UNITS: persons

Persons_in_stock_cohort[M, A29](t) = Persons_in_stock_cohort[M, A29](t - dt) + (stock_cohort_ageing_in[M, A29] + Births[M, A29] + Stock_Immigration[M, A29] - stock_cohort_ageing_out[M, A29] - stock_cohort_deaths[M, A29] - stock_Emmigration[M, A29]) * dt

INIT Persons_in_stock_cohort[M, A29] = 841084

UNITS: persons

Persons_in_stock_cohort[M, A34](t) = Persons_in_stock_cohort[M, A34](t - dt) + (stock_cohort_ageing_in[M, A34] + Births[M, A34] + Stock_Immigration[M, A34] - stock_cohort_ageing_out[M, A34] - stock_cohort_deaths[M, A34] - stock_Emmigration[M, A34]) * dt

INIT Persons_in_stock_cohort[M, A34] = 769211

UNITS: persons

Persons_in_stock_cohort[M, A39](t) = Persons_in_stock_cohort[M, A39](t - dt) + (stock_cohort_ageing_in[M, A39] + Births[M, A39] + Stock_Immigration[M, A39] - stock_cohort_ageing_out[M, A39] - stock_cohort_deaths[M, A39] - stock_Emmigration[M, A39]) * dt

INIT Persons_in_stock_cohort[M, A39] = 782204

UNITS: persons

Persons_in_stock_cohort[M, A44](t) = Persons_in_stock_cohort[M, A44](t - dt) + (stock_cohort_ageing_in[M, A44] + Births[M, A44] + Stock_Immigration[M, A44] - stock_cohort_ageing_out[M, A44] - stock_cohort_deaths[M, A44] - stock_Emmigration[M, A44]) * dt

INIT Persons_in_stock_cohort[M, A44] = 786748

UNITS: persons

Persons_in_stock_cohort[M, A49](t) = Persons_in_stock_cohort[M, A49](t - dt) + (stock_cohort_ageing_in[M, A49] + Births[M, A49] + Stock_Immigration[M, A49] - stock_cohort_ageing_out[M, A49] - stock_cohort_deaths[M, A49] - stock_Emmigration[M, A49]) * dt

INIT Persons_in_stock_cohort[M, A49] = 764147

UNITS: persons

Persons_in_stock_cohort[M, A54](t) = Persons_in_stock_cohort[M, A54](t - dt) + (stock_cohort_ageing_in[M, A54] + Births[M, A54] + Stock_Immigration[M, A54] - stock_cohort_ageing_out[M, A54] - stock_cohort_deaths[M, A54] - stock_Emmigration[M, A54]) * dt

INIT Persons_in_stock_cohort[M, A54] = 739627

UNITS: persons

Persons_in_stock_cohort[M, A59](t) = Persons_in_stock_cohort[M, A59](t - dt) + (stock_cohort_ageing_in[M, A59] + Births[M, A59] + Stock_Immigration[M, A59] - stock_cohort_ageing_out[M, A59] - stock_cohort_deaths[M, A59] - stock_Emmigration[M, A59]) * dt

INIT Persons_in_stock_cohort[M, A59] = 662069

UNITS: persons

Persons_in_stock_cohort[M, A64](t) = Persons_in_stock_cohort[M, A64](t - dt) + (stock_cohort_ageing_in[M, A64] + Births[M, A64] + Stock_Immigration[M, A64] - stock_cohort_ageing_out[M, A64] - stock_cohort_deaths[M, A64] - stock_Emmigration[M, A64]) * dt

INIT Persons_in_stock_cohort[M, A64] = 611198

UNITS: persons

Persons_in_stock_cohort[M, A69](t) = Persons_in_stock_cohort[M, A69](t - dt) + (stock_cohort_ageing_in[M, A69] + Births[M, A69] + Stock_Immigration[M, A69] - stock_cohort_ageing_out[M, A69] - stock_cohort_deaths[M, A69] - stock_Emmigration[M, A69]) * dt

INIT Persons_in_stock_cohort[M, A69] = 474253

UNITS: persons

Persons_in_stock_cohort[M, A74](t) = Persons_in_stock_cohort[M, A74](t - dt) + (stock_cohort_ageing_in[M, A74] + Births[M, A74] + Stock_Immigration[M, A74] - stock_cohort_ageing_out[M, A74] - stock_cohort_deaths[M, A74] - stock_Emmigration[M, A74]) * dt

INIT Persons_in_stock_cohort[M, A74] = 357296

UNITS: persons

Persons_in_stock_cohort[M, A79](t) = Persons_in_stock_cohort[M, A79](t - dt) + (stock_cohort_ageing_in[M, A79] + Births[M, A79] + Stock_Immigration[M, A79] - stock_cohort_ageing_out[M, A79] - stock_cohort_deaths[M, A79] - stock_Emmigration[M, A79]) * dt

INIT Persons_in_stock_cohort[M, A79] = 258411

UNITS: persons

Persons_in_stock_cohort[M, A84](t) = Persons_in_stock_cohort[M, A84](t - dt) + (stock_cohort_ageing_in[M, A84] + Births[M, A84] + Stock_Immigration[M, A84] - stock_cohort_ageing_out[M, A84] - stock_cohort_deaths[M, A84] - stock_Emmigration[M, A84]) * dt

INIT Persons_in_stock_cohort[M, A84] = 190572

UNITS: persons

Persons_in_stock_cohort[M, A89](t) = Persons_in_stock_cohort[M, A89](t - dt) + (stock_cohort_ageing_in[M, A89] + Births[M, A89] + Stock_Immigration[M, A89] - stock_cohort_ageing_out[M, A89] - stock_cohort_deaths[M, A89] - stock_Emmigration[M, A89]) * dt

INIT Persons_in_stock_cohort[M, A89] = 100736.359135396

UNITS: persons

Persons_in_stock_cohort[M, A94](t) = Persons_in_stock_cohort[M, A94](t - dt) + (stock_cohort_ageing_in[M, A94] + Births[M, A94] + Stock_Immigration[M, A94] - stock_cohort_ageing_out[M, A94] - stock_cohort_deaths[M, A94] - stock_Emmigration[M, A94]) * dt

INIT Persons_in_stock_cohort[M, A94] = 31772.9809861888

UNITS: persons

Persons_in_stock_cohort[M, A99](t) = Persons_in_stock_cohort[M, A99](t - dt) + (stock_cohort_ageing_in[M, A99] + Births[M, A99] + Stock_Immigration[M, A99] - stock_cohort_ageing_out[M, A99] - stock_cohort_deaths[M, A99] - stock_Emmigration[M, A99]) * dt

INIT Persons_in_stock_cohort[M, A99] = 5905.09356002357

UNITS: persons

Persons_in_stock_cohort[M, A104](t) = Persons_in_stock_cohort[M, A104](t - dt) + (stock_cohort_ageing_in[M, A104] + Births[M, A104] + Stock_Immigration[M, A104] - stock_cohort_ageing_out[M, A104] - stock_cohort_deaths[M, A104] - stock_Emmigration[M, A104]) * dt

INIT Persons_in_stock_cohort[M, A104] = 655.566318391515

UNITS: persons

Persons_in_stock_cohort[F, A4](t) = Persons_in_stock_cohort[F, A4](t - dt) + (stock_cohort_ageing_in[F, A4] + Births[F, A4] + Stock_Immigration[F, A4] - stock_cohort_ageing_out[F, A4] - stock_cohort_deaths[F, A4] - stock_Emmigration[F, A4]) * dt

INIT Persons_in_stock_cohort[F, A4] = 709587

UNITS: persons

Persons_in_stock_cohort[F, A9](t) = Persons_in_stock_cohort[F, A9](t - dt) + (stock_cohort_ageing_in[F, A9] + Births[F, A9] + Stock_Immigration[F, A9] - stock_cohort_ageing_out[F, A9] - stock_cohort_deaths[F, A9] - stock_Emmigration[F, A9]) * dt

INIT Persons_in_stock_cohort[F, A9] = 675429

UNITS: persons

Persons_in_stock_cohort[F, A14](t) = Persons_in_stock_cohort[F, A14](t - dt) + (stock_cohort_ageing_in[F, A14] + Births[F, A14] + Stock_Immigration[F, A14] - stock_cohort_ageing_out[F, A14] - stock_cohort_deaths[F, A14] - stock_Emmigration[F, A14]) * dt

INIT Persons_in_stock_cohort[F, A14] = 676322

UNITS: persons

Persons_in_stock_cohort[F, A19](t) = Persons_in_stock_cohort[F, A19](t - dt) + (stock_cohort_ageing_in[F, A19] + Births[F, A19] + Stock_Immigration[F, A19] - stock_cohort_ageing_out[F, A19] - stock_cohort_deaths[F, A19] - stock_Emmigration[F, A19]) * dt

INIT Persons_in_stock_cohort[F, A19] = 706860

UNITS: persons

Persons_in_stock_cohort[F, A24](t) = Persons_in_stock_cohort[F, A24](t - dt) + (stock_cohort_ageing_in[F, A24] + Births[F, A24] + Stock_Immigration[F, A24] - stock_cohort_ageing_out[F, A24] - stock_cohort_deaths[F, A24] - stock_Emmigration[F, A24]) * dt

INIT Persons_in_stock_cohort[F, A24] = 788193

UNITS: persons

Persons_in_stock_cohort[F, A29](t) = Persons_in_stock_cohort[F, A29](t - dt) + (stock_cohort_ageing_in[F, A29] + Births[F, A29] + Stock_Immigration[F, A29] - stock_cohort_ageing_out[F, A29] - stock_cohort_deaths[F, A29] - stock_Emmigration[F, A29]) * dt

INIT Persons_in_stock_cohort[F, A29] = 817086

UNITS: persons

Persons_in_stock_cohort[F, A34](t) = Persons_in_stock_cohort[F, A34](t - dt) + (stock_cohort_ageing_in[F, A34] + Births[F, A34] + Stock_Immigration[F, A34] - stock_cohort_ageing_out[F, A34] - stock_cohort_deaths[F, A34] - stock_Emmigration[F, A34]) * dt

INIT Persons_in_stock_cohort[F, A34] = 766950

UNITS: persons

Persons_in_stock_cohort[F, A39](t) = Persons_in_stock_cohort[F, A39](t - dt) + (stock_cohort_ageing_in[F, A39] + Births[F, A39] + Stock_Immigration[F, A39] - stock_cohort_ageing_out[F, A39] - stock_cohort_deaths[F, A39] - stock_Emmigration[F, A39]) * dt

INIT Persons_in_stock_cohort[F, A39] = 791706

UNITS: persons

Persons_in_stock_cohort[F, A44](t) = Persons_in_stock_cohort[F, A44](t - dt) + (stock_cohort_ageing_in[F, A44] + Births[F, A44] + Stock_Immigration[F, A44] - stock_cohort_ageing_out[F, A44] - stock_cohort_deaths[F, A44] - stock_Emmigration[F, A44]) * dt

INIT Persons_in_stock_cohort[F, A44] = 800496

UNITS: persons

Persons_in_stock_cohort[F, A49](t) = Persons_in_stock_cohort[F, A49](t - dt) + (stock_cohort_ageing_in[F, A49] + Births[F, A49] + Stock_Immigration[F, A49] - stock_cohort_ageing_out[F, A49] - stock_cohort_deaths[F, A49] - stock_Emmigration[F, A49]) * dt

INIT Persons_in_stock_cohort[F, A49] = 777690

UNITS: persons

Persons_in_stock_cohort[F, A54](t) = Persons_in_stock_cohort[F, A54](t - dt) + (stock_cohort_ageing_in[F, A54] + Births[F, A54] + Stock_Immigration[F, A54] - stock_cohort_ageing_out[F, A54] - stock_cohort_deaths[F, A54] - stock_Emmigration[F, A54]) * dt

INIT Persons_in_stock_cohort[F, A54] = 754436

UNITS: persons

Persons_in_stock_cohort[F, A59](t) = Persons_in_stock_cohort[F, A59](t - dt) + (stock_cohort_ageing_in[F, A59] + Births[F, A59] + Stock_Immigration[F, A59] - stock_cohort_ageing_out[F, A59] - stock_cohort_deaths[F, A59] - stock_Emmigration[F, A59]) * dt

INIT Persons_in_stock_cohort[F, A59] = 673924

UNITS: persons

Persons_in_stock_cohort[F, A64](t) = Persons_in_stock_cohort[F, A64](t - dt) + (stock_cohort_ageing_in[F, A64] + Births[F, A64] + Stock_Immigration[F, A64] - stock_cohort_ageing_out[F, A64] - stock_cohort_deaths[F, A64] - stock_Emmigration[F, A64]) * dt

INIT Persons_in_stock_cohort[F, A64] = 614802

UNITS: persons

Persons_in_stock_cohort[F, A69](t) = Persons_in_stock_cohort[F, A69](t - dt) + (stock_cohort_ageing_in[F, A69] + Births[F, A69] + Stock_Immigration[F, A69] - stock_cohort_ageing_out[F, A69] - stock_cohort_deaths[F, A69] - stock_Emmigration[F, A69]) * dt

INIT Persons_in_stock_cohort[F, A69] = 480007

UNITS: persons

Persons_in_stock_cohort[F, A74](t) = Persons_in_stock_cohort[F, A74](t - dt) + (stock_cohort_ageing_in[F, A74] + Births[F, A74] + Stock_Immigration[F, A74] - stock_cohort_ageing_out[F, A74] - stock_cohort_deaths[F, A74] - stock_Emmigration[F, A74]) * dt

INIT Persons_in_stock_cohort[F, A74] = 370375

UNITS: persons

Persons_in_stock_cohort[F, A79](t) = Persons_in_stock_cohort[F, A79](t - dt) + (stock_cohort_ageing_in[F, A79] + Births[F, A79] + Stock_Immigration[F, A79] - stock_cohort_ageing_out[F, A79] - stock_cohort_deaths[F, A79] - stock_Emmigration[F, A79]) * dt

INIT Persons_in_stock_cohort[F, A79] = 299930

Persons_in_stock_cohort[F, A84](t) = Persons_in_stock_cohort[F, A84](t - dt) + (stock_cohort_ageing_in[F, A84] + Births[F, A84] + Stock_Immigration[F, A84] - stock_cohort_ageing_out[F, A84] - stock_cohort_deaths[F, A84] - stock_Emmigration[F, A84]) * dt

INIT Persons_in_stock_cohort[F, A84] = 253460

UNITS: persons

Persons_in_stock_cohort[F, A89](t) = Persons_in_stock_cohort[F, A89](t - dt) + (stock_cohort_ageing_in[F, A89] + Births[F, A89] + Stock_Immigration[F, A89] - stock_cohort_ageing_out[F, A89] - stock_cohort_deaths[F, A89] - stock_Emmigration[F, A89]) * dt

INIT Persons_in_stock_cohort[F, A89] = 170652.937760423

UNITS: persons

Persons_in_stock_cohort[F, A94](t) = Persons_in_stock_cohort[F, A94](t - dt) + (stock_cohort_ageing_in[F, A94] + Births[F, A94] + Stock_Immigration[F, A94] - stock_cohort_ageing_out[F, A94] - stock_cohort_deaths[F, A94] - stock_Emmigration[F, A94]) * dt

INIT Persons_in_stock_cohort[F, A94] = 72260.4408001093

Persons_in_stock_cohort[F, A99](t) = Persons_in_stock_cohort[F, A99](t - dt) + (stock_cohort_ageing_in[F, A99] + Births[F, A99] + Stock_Immigration[F, A99] - stock_cohort_ageing_out[F, A99] - stock_cohort_deaths[F, A99] - stock_Emmigration[F, A99]) * dt

INIT Persons_in_stock_cohort[F, A99] = 18996.4618616089

UNITS: persons

(stock_cohort_ageing_in[F, A104] + Births[F, A104] + Stock_Immigration[F, A104] - stock_cohort_ageing_out[F, A104] - stock_cohort_deaths[F, A104] - stock_Emmigration[F, A104]) * dt

INIT Persons_in_stock_cohort[F, A104] = 2627.15957785924

UNITS: persons

Age_specific_Death_Rate[Gender, Age] = IF Death_rate_switch_2M_3H = 2 THEN death_rate_M ELSE death_rate_H

UNITS: persons/person/year

Age_specific_Fertility_rate[Age] = IF Fertility_rate_switch_1L_2M_3H = 1 THEN Age_specific_fertility_rate_L[Age] ELSE IF Fertility_rate_switch_1L_2M_3H = 2 THEN Age_specific_fertility_rate_M[Age] ELSE Age_specific_fertility_rate_H[Age]

UNITS: persons/person/year

Age_specific_fertility_rate_H[A4] = GRAPH(TIME)

Points: (2011.00, 0.00), (2012.00, 0.00), (2013.00, 0.00), (2014.00, 0.00), (2015.00, 0.00), (2016.00, 0.00), (2017.00, 0.00), (2018.00, 0.00), (2019.00, 0.00), (2020.00, 0.00), (2021.00, 0.00), (2022.00, 0.00), (2023.00, 0.00), (2024.00, 0.00), (2025.00, 0.00), (2026.00, 0.00)

UNITS: person/person/year

Age_specific_fertility_rate_H[A9] = GRAPH(TIME)

Points: (2011.00, 0.0), (2012.00, 0.0), (2013.00, 0.0), (2014.00, 0.0), (2015.00, 0.0), (2016.00, 0.0), (2017.00, 0.0), (2018.00, 0.0), (2019.00, 0.0), (2020.00, 0.0), (2021.00, 0.0), (2022.00, 0.0), (2023.00, 0.0), (2024.00, 0.0), (2025.00, 0.0), (2026.00, 0.0)

UNITS: person/person/year

Age_specific_fertility_rate_H[A14] = GRAPH(TIME)

Points: (2011.00, 0.0), (2012.00, 0.0), (2013.00, 0.0), (2014.00, 0.0), (2015.00, 0.0), (2016.00, 0.0), (2017.00, 0.0), (2018.00, 0.0), (2019.00, 0.0), (2020.00, 0.0), (2021.00, 0.0), (2022.00, 0.0), (2023.00, 0.0), (2024.00, 0.0), (2025.00, 0.0), (2026.00, 0.0)

UNITS: person/person/year

Age_specific_fertility_rate_H[A19] = GRAPH(TIME)

Points: (2011.00, 16.1), (2012.00, 16.1), (2013.00, 14.6), (2014.00, 12.8), (2015.00, 11.9), (2016.00, 10.5), (2017.00, 13.83256), (2018.00, 13.69888), (2019.00, 13.56446), (2020.00, 13.42936), (2021.00, 13.29354), (2022.00, 13.15698), (2023.00, 13.0197), (2024.00, 12.88174), (2025.00, 12.74306), (2026.00, 12.60364)

UNITS: person/person/year

Age_specific_fertility_rate_H[A24] = GRAPH(TIME)

Points: (2011.00, 53.0), (2012.00, 53.1), (2013.00, 51.1), (2014.00, 47.3), (2015.00, 47.1), (2016.00, 44.6), (2017.00, 49.63024), (2018.00, 49.30502), (2019.00, 48.97788), (2020.00, 48.64884), (2021.00, 48.31792), (2022.00, 47.9851), (2023.00, 47.6504), (2024.00, 47.31382), (2025.00, 46.97532), (2026.00, 46.63494)

UNITS: person/person/year

Age_specific_fertility_rate_H[A29] = GRAPH(TIME)

Points: (2011.00, 103.1), (2012.00, 102.5), (2013.00, 98.9), (2014.00, 94.4), (2015.00, 92.9), (2016.00, 91.9), (2017.00, 102.63114), (2018.00, 102.35428), (2019.00, 102.07526), (2020.00, 101.79402), (2021.00, 101.51058), (2022.00, 101.225), (2023.00, 100.9372), (2024.00, 100.64718), (2025.00, 100.35506), (2026.00, 100.06072)

UNITS: person/person/year

Age_specific_fertility_rate_H[A34] = GRAPH(TIME)

Points: (2011.00, 124.9), (2012.00, 126.8), (2013.00, 124.6), (2014.00, 120.2), (2015.00, 121.5), (2016.00, 123.4), (2017.00, 129.42368), (2018.00, 129.66488), (2019.00, 129.9059), (2020.00, 130.14676), (2021.00, 130.38742), (2022.00, 130.62786), (2023.00, 130.86812), (2024.00, 131.10824), (2025.00, 131.34818), (2026.00, 131.58792)

UNITS: person/person/year

Age_specific_fertility_rate_H[A39] = GRAPH(TIME)

Points: (2011.00, 70.30), (2012.00, 71.50), (2013.00, 70.80), (2014.00, 69.10), (2015.00, 69.60), (2016.00, 71.90), (2017.00, 77.4904), (2018.00, 78.31042), (2019.00, 79.13324), (2020.00, 79.95896), (2021.00, 80.7875), (2022.00, 81.61886), (2023.00, 82.45304), (2024.00, 83.29012), (2025.00, 84.13), (2026.00, 84.9727)

UNITS: person/person/year

Age_specific_fertility_rate_H[A44] = GRAPH(TIME)

Points: (2011.00, 15.2), (2012.00, 15.2), (2013.00, 15.4), (2014.00, 14.4), (2015.00, 14.6), (2016.00, 15.3), (2017.00, 18.03466), (2018.00, 18.50938), (2019.00, 18.98598), (2020.00, 19.46446), (2021.00, 19.9449), (2022.00, 20.4272), (2023.00, 20.91138), (2024.00, 21.39748), (2025.00, 21.88546), (2026.00, 22.37536)

UNITS: person/person/year

Age_specific_fertility_rate_H[A49] = GRAPH(TIME)

Points: (2011.00, 0.9), (2012.00, 1.0), (2013.00, 0.9), (2014.00, 1.0), (2015.00, 1.1), (2016.00, 1.2), (2017.00, 1.24302), (2018.00, 1.30004), (2019.00, 1.35726), (2020.00, 1.41478), (2021.00, 1.47252), (2022.00, 1.53048), (2023.00, 1.58868), (2024.00, 1.64712), (2025.00, 1.7058), (2026.00, 1.7647)

UNITS: person/person/year

Age_specific_fertility_rate_H[A54] = GRAPH(TIME)

Points: (2011.000, 0.0), (2011.33333333, 0.0), (2011.66666667, 0.0), (2012.000, 0.0), (2012.33333333, 0.0), (2012.66666667, 0.0), (2013.000, 0.0), (2013.33333333, 0.0), (2013.66666667, 0.0), (2014.000, 0.0), (2014.33333333, 0.0), (2014.66666667, 0.0), (2015.000, 0.0), (2015.33333333, 0.0), (2015.66666667, 0.0), (2016.000, 0.0)

UNITS: person/person/year

Age_specific_fertility_rate_H[A59] = GRAPH(TIME)

Points: (2011.000, 0.0), (2011.33333333, 0.0), (2011.66666667, 0.0), (2012.000, 0.0), (2012.33333333, 0.0), (2012.66666667, 0.0), (2013.000, 0.0), (2013.33333333, 0.0), (2013.66666667, 0.0), (2014.000, 0.0), (2014.33333333, 0.0), (2014.66666667, 0.0), (2015.000, 0.0), (2015.33333333, 0.0), (2015.66666667, 0.0), (2016.000, 0.0)

UNITS: person/person/year

Age_specific_fertility_rate_H[A64] = GRAPH(TIME)

Points: (2011.000, 0.0), (2011.33333333, 0.0), (2011.66666667, 0.0), (2012.000, 0.0), (2012.33333333, 0.0), (2012.66666667, 0.0), (2013.000, 0.0), (2013.33333333, 0.0), (2013.66666667, 0.0), (2014.000, 0.0), (2014.33333333, 0.0), (2014.66666667, 0.0), (2015.000, 0.0), (2015.33333333, 0.0), (2015.66666667, 0.0), (2016.000, 0.0)

UNITS: person/person/year

Age_specific_fertility_rate_H[A69] = GRAPH(TIME)

Points: (2011.000, 0.0), (2011.33333333, 0.0), (2011.66666667, 0.0), (2012.000, 0.0), (2012.33333333, 0.0), (2012.66666667, 0.0), (2013.000, 0.0), (2013.33333333, 0.0), (2013.66666667, 0.0), (2014.000, 0.0), (2014.33333333, 0.0), (2014.66666667, 0.0), (2015.000, 0.0), (2015.33333333, 0.0), (2015.66666667, 0.0), (2016.000, 0.0)

UNITS: person/person/year

Age_specific_fertility_rate_H[A74] = GRAPH(TIME)

Points: (2011.000, 0.0), (2011.33333333, 0.0), (2011.66666667, 0.0), (2012.000, 0.0), (2012.33333333, 0.0), (2012.66666667, 0.0), (2013.000, 0.0), (2013.33333333, 0.0), (2013.66666667, 0.0), (2014.000, 0.0), (2014.33333333, 0.0), (2014.66666667, 0.0), (2015.000, 0.0), (2015.33333333, 0.0), (2015.66666667, 0.0), (2016.000, 0.0)

UNITS: person/person/year

Age_specific_fertility_rate_H[A79] = GRAPH(TIME)

Points: (2011.000, 0.0), (2011.33333333, 0.0), (2011.66666667, 0.0), (2012.000, 0.0), (2012.33333333, 0.0), (2012.66666667, 0.0), (2013.000, 0.0), (2013.33333333, 0.0), (2013.66666667, 0.0), (2014.000, 0.0), (2014.33333333, 0.0), (2014.66666667, 0.0), (2015.000, 0.0), (2015.33333333, 0.0), (2015.66666667, 0.0), (2016.000, 0.0)

UNITS: person/person/year

Age_specific_fertility_rate_H[A84] = GRAPH(TIME)

Points: (2011.000, 0.0), (2011.33333333, 0.0), (2011.66666667, 0.0), (2012.000, 0.0), (2012.33333333, 0.0), (2012.66666667, 0.0), (2013.000, 0.0), (2013.33333333, 0.0), (2013.66666667, 0.0), (2014.000, 0.0), (2014.33333333, 0.0), (2014.66666667, 0.0), (2015.000, 0.0), (2015.33333333, 0.0), (2015.66666667, 0.0), (2016.000, 0.0)

UNITS: person/person/year

Age_specific_fertility_rate_H[A89] = GRAPH(TIME)

Points: (2011.000, 0.0), (2011.33333333, 0.0), (2011.66666667, 0.0), (2012.000, 0.0), (2012.33333333, 0.0), (2012.66666667, 0.0), (2013.000, 0.0), (2013.33333333, 0.0), (2013.66666667, 0.0), (2014.000, 0.0), (2014.33333333, 0.0), (2014.66666667, 0.0), (2015.000, 0.0), (2015.33333333, 0.0), (2015.66666667, 0.0), (2016.000, 0.0)

UNITS: person/person/year

Age_specific_fertility_rate_H[A94] = GRAPH(TIME)

Points: (2011.000, 0.0), (2011.33333333, 0.0), (2011.66666667, 0.0), (2012.000, 0.0), (2012.33333333, 0.0), (2012.66666667, 0.0), (2013.000, 0.0), (2013.33333333, 0.0), (2013.66666667, 0.0), (2014.000, 0.0), (2014.33333333, 0.0), (2014.66666667, 0.0), (2015.000, 0.0), (2015.33333333, 0.0), (2015.66666667, 0.0), (2016.000, 0.0)

UNITS: person/person/year

Age_specific_fertility_rate_H[A99] = GRAPH(TIME)

Points: (2011.000, 0.0), (2011.33333333, 0.0), (2011.66666667, 0.0), (2012.000, 0.0), (2012.33333333, 0.0), (2012.66666667, 0.0), (2013.000, 0.0), (2013.33333333, 0.0), (2013.66666667, 0.0), (2014.000, 0.0), (2014.33333333, 0.0), (2014.66666667, 0.0), (2015.000, 0.0), (2015.33333333, 0.0), (2015.66666667, 0.0), (2016.000, 0.0)

UNITS: person/person/year

Age_specific_fertility_rate_H[A104] = GRAPH(TIME)

Points: (2011.000, 0.0), (2011.33333333, 0.0), (2011.66666667, 0.0), (2012.000, 0.0), (2012.33333333, 0.0), (2012.66666667, 0.0), (2013.000, 0.0), (2013.33333333, 0.0), (2013.66666667, 0.0), (2014.000, 0.0), (2014.33333333, 0.0), (2014.66666667, 0.0), (2015.000, 0.0), (2015.33333333, 0.0), (2015.66666667, 0.0), (2016.000, 0.0)

UNITS: person/person/year

Age_specific_fertility_rate_L[A4] = GRAPH(TIME)

Points: (2011.00, 0.0), (2012.00, 0.0), (2013.00, 0.0), (2014.00, 0.0), (2015.00, 0.0), (2016.00, 0.0), (2017.00, 0.0), (2018.00, 0.0), (2019.00, 0.0), (2020.00, 0.0), (2021.00, 0.0), (2022.00, 0.0), (2023.00, 0.0), (2024.00, 0.0), (2025.00, 0.0), (2026.00, 0.0)

UNITS: person/person/year

Age_specific_fertility_rate_L[A9] = GRAPH(TIME)

Points: (2011.00, 0.0), (2012.00, 0.0), (2013.00, 0.0), (2014.00, 0.0), (2015.00, 0.0), (2016.00, 0.0), (2017.00, 0.0), (2018.00, 0.0), (2019.00, 0.0), (2020.00, 0.0), (2021.00, 0.0), (2022.00, 0.0), (2023.00, 0.0), (2024.00, 0.0), (2025.00, 0.0), (2026.00, 0.0)

UNITS: person/person/year

Age_specific_fertility_rate_L[A14] = GRAPH(TIME)

Points: (2011.00, 0.0), (2012.00, 0.0), (2013.00, 0.0), (2014.00, 0.0), (2015.00, 0.0), (2016.00, 0.0), (2017.00, 0.0), (2018.00, 0.0), (2019.00, 0.0), (2020.00, 0.0), (2021.00, 0.0), (2022.00, 0.0), (2023.00, 0.0), (2024.00, 0.0), (2025.00, 0.0), (2026.00, 0.0)

UNITS: person/person/year

Age_specific_fertility_rate_L[A19] = GRAPH(TIME)

Points: (2011.00, 16.1), (2012.00, 16.1), (2013.00, 14.6), (2014.00, 12.8), (2015.00, 11.9), (2016.00, 10.5), (2017.00, 12.64376), (2018.00, 12.34492), (2019.00, 12.04964), (2020.00, 11.758), (2021.00, 11.46986), (2022.00, 11.18532), (2023.00, 10.90434), (2024.00, 10.62696), (2025.00, 10.35316), (2026.00, 10.08294)

UNITS: person/person/year

Age_specific_fertility_rate_L[A24] = GRAPH(TIME)

Points: (2011.00, 53.0), (2012.00, 53.1), (2013.00, 51.1), (2014.00, 47.3), (2015.00, 47.1), (2016.00, 44.6), (2017.00, 45.36488), (2018.00, 44.43182), (2019.00, 43.50824), (2020.00, 42.59414), (2021.00, 41.68946), (2022.00, 40.79422), (2023.00, 39.9085), (2024.00, 39.03222), (2025.00, 38.16536), (2026.00, 37.30796)

UNITS: person/person/year

Age_specific_fertility_rate_L[A29] = GRAPH(TIME)

Points: (2011.00, 103.1), (2012.00, 102.5), (2013.00, 98.9), (2014.00, 94.4), (2015.00, 92.9), (2016.00, 91.9), (2017.00, 93.8107), (2018.00, 92.23788), (2019.00, 90.67598), (2020.00, 89.125), (2021.00, 87.58494), (2022.00, 86.0558), (2023.00, 84.53762), (2024.00, 83.03036), (2025.00, 81.534), (2026.00, 80.04856)

UNITS: person/person/year

Age_specific_fertility_rate_L[A34] = GRAPH(TIME)

Points: (2011.00, 124.9), (2012.00, 126.8), (2013.00, 124.6), (2014.00, 120.2), (2015.00, 121.5), (2016.00, 123.4), (2017.00, 118.30062), (2018.00, 116.84918), (2019.00, 115.39864), (2020.00, 113.94904), (2021.00, 112.50032), (2022.00, 111.0525), (2023.00, 109.6056), (2024.00, 108.15962), (2025.00, 106.71452), (2026.00, 105.27034)

UNITS: person/person/year

Age_specific_fertility_rate_L[A39] = GRAPH(TIME)

Points: (2011.00, 70.3), (2012.00, 71.5), (2013.00, 70.8), (2014.00, 69.1), (2015.00, 69.6), (2016.00, 71.9), (2017.00, 70.83062), (2018.00, 70.57044), (2019.00, 70.29604), (2020.00, 70.00748), (2021.00, 69.70472), (2022.00, 69.38778), (2023.00, 69.05666), (2024.00, 68.71136), (2025.00, 68.35184), (2026.00, 67.97816)

UNITS: person/person/year

Age_specific_fertility_rate_L[A44] = GRAPH(TIME)

Points: (2011.00, 15.2), (2012.00, 15.2), (2013.00, 15.4), (2014.00, 14.4), (2015.00, 14.6), (2016.00, 15.3), (2017.00, 16.4847), (2018.00, 16.67994), (2019.00, 16.8657), (2020.00, 17.04198), (2021.00, 17.20874), (2022.00, 17.36602), (2023.00, 17.51384), (2024.00, 17.65216), (2025.00, 17.78092), (2026.00, 17.90028)

UNITS: person/person/year

Age_specific_fertility_rate_L[A49] = GRAPH(TIME)

Points: (2011.00, 0.9), (2012.00, 1.0), (2013.00, 0.9), (2014.00, 1.0), (2015.00, 1.1), (2016.00, 1.2), (2017.00, 1.13618), (2018.00, 1.17154), (2019.00, 1.20572), (2020.00, 1.23868), (2021.00, 1.27052), (2022.00, 1.30112), (2023.00, 1.33058), (2024.00, 1.35882), (2025.00, 1.3859), (2026.00, 1.41176)

UNITS: person/person/year

Age_specific_fertility_rate_L[A54] = GRAPH(TIME)

Points: (2011.000, 0.0), (2011.33333333, 0.0), (2011.66666667, 0.0), (2012.000, 0.0), (2012.33333333, 0.0), (2012.66666667, 0.0), (2013.000, 0.0), (2013.33333333, 0.0), (2013.66666667, 0.0), (2014.000, 0.0), (2014.33333333, 0.0), (2014.66666667, 0.0), (2015.000, 0.0), (2015.33333333, 0.0), (2015.66666667, 0.0), (2016.000, 0.0)

UNITS: person/person/year

Age_specific_fertility_rate_L[A59] = GRAPH(TIME)

Points: (2011.000, 0.0), (2011.33333333, 0.0), (2011.66666667, 0.0), (2012.000, 0.0), (2012.33333333, 0.0), (2012.66666667, 0.0), (2013.000, 0.0), (2013.33333333, 0.0), (2013.66666667, 0.0), (2014.000, 0.0), (2014.33333333, 0.0), (2014.66666667, 0.0), (2015.000, 0.0), (2015.33333333, 0.0), (2015.66666667, 0.0), (2016.000, 0.0)

UNITS: person/person/year

Age_specific_fertility_rate_L[A64] = GRAPH(TIME)

Points: (2011.000, 0.0), (2011.33333333, 0.0), (2011.66666667, 0.0), (2012.000, 0.0), (2012.33333333, 0.0), (2012.66666667, 0.0), (2013.000, 0.0), (2013.33333333, 0.0), (2013.66666667, 0.0), (2014.000, 0.0), (2014.33333333, 0.0), (2014.66666667, 0.0), (2015.000, 0.0), (2015.33333333, 0.0), (2015.66666667, 0.0), (2016.000, 0.0)

UNITS: person/person/year

Age_specific_fertility_rate_L[A69] = GRAPH(TIME)

Points: (2011.000, 0.0), (2011.33333333, 0.0), (2011.66666667, 0.0), (2012.000, 0.0), (2012.33333333, 0.0), (2012.66666667, 0.0), (2013.000, 0.0), (2013.33333333, 0.0), (2013.66666667, 0.0), (2014.000, 0.0), (2014.33333333, 0.0), (2014.66666667, 0.0), (2015.000, 0.0), (2015.33333333, 0.0), (2015.66666667, 0.0), (2016.000, 0.0)

UNITS: person/person/year

Age_specific_fertility_rate_L[A74] = GRAPH(TIME)

Points: (2011.000, 0.0), (2011.33333333, 0.0), (2011.66666667, 0.0), (2012.000, 0.0), (2012.33333333, 0.0), (2012.66666667, 0.0), (2013.000, 0.0), (2013.33333333, 0.0), (2013.66666667, 0.0), (2014.000, 0.0), (2014.33333333, 0.0), (2014.66666667, 0.0), (2015.000, 0.0), (2015.33333333, 0.0), (2015.66666667, 0.0), (2016.000, 0.0)

UNITS: person/person/year

Age_specific_fertility_rate_L[A79] = GRAPH(TIME)

Points: (2011.000, 0.0), (2011.33333333, 0.0), (2011.66666667, 0.0), (2012.000, 0.0), (2012.33333333, 0.0), (2012.66666667, 0.0), (2013.000, 0.0), (2013.33333333, 0.0), (2013.66666667, 0.0), (2014.000, 0.0), (2014.33333333, 0.0), (2014.66666667, 0.0), (2015.000, 0.0), (2015.33333333, 0.0), (2015.66666667, 0.0), (2016.000, 0.0)

UNITS: person/person/year

Age_specific_fertility_rate_L[A84] = GRAPH(TIME)

Points: (2011.000, 0.0), (2011.33333333, 0.0), (2011.66666667, 0.0), (2012.000, 0.0), (2012.33333333, 0.0), (2012.66666667, 0.0), (2013.000, 0.0), (2013.33333333, 0.0), (2013.66666667, 0.0), (2014.000, 0.0), (2014.33333333, 0.0), (2014.66666667, 0.0), (2015.000, 0.0), (2015.33333333, 0.0), (2015.66666667, 0.0), (2016.000, 0.0)

UNITS: person/person/year

Age_specific_fertility_rate_L[A89] = GRAPH(TIME)

Points: (2011.000, 0.0), (2011.33333333, 0.0), (2011.66666667, 0.0), (2012.000, 0.0), (2012.33333333, 0.0), (2012.66666667, 0.0), (2013.000, 0.0), (2013.33333333, 0.0), (2013.66666667, 0.0), (2014.000, 0.0), (2014.33333333, 0.0), (2014.66666667, 0.0), (2015.000, 0.0), (2015.33333333, 0.0), (2015.66666667, 0.0), (2016.000, 0.0)

UNITS: person/person/year

Age_specific_fertility_rate_L[A94] = GRAPH(TIME)

Points: (2011.000, 0.0), (2011.33333333, 0.0), (2011.66666667, 0.0), (2012.000, 0.0), (2012.33333333, 0.0), (2012.66666667, 0.0), (2013.000, 0.0), (2013.33333333, 0.0), (2013.66666667, 0.0), (2014.000, 0.0), (2014.33333333, 0.0), (2014.66666667, 0.0), (2015.000, 0.0), (2015.33333333, 0.0), (2015.66666667, 0.0), (2016.000, 0.0)

UNITS: person/person/year

Age_specific_fertility_rate_L[A99] = GRAPH(TIME)

Points: (2011.000, 0.0), (2011.33333333, 0.0), (2011.66666667, 0.0), (2012.000, 0.0), (2012.33333333, 0.0), (2012.66666667, 0.0), (2013.000, 0.0), (2013.33333333, 0.0), (2013.66666667, 0.0), (2014.000, 0.0), (2014.33333333, 0.0), (2014.66666667, 0.0), (2015.000, 0.0), (2015.33333333, 0.0), (2015.66666667, 0.0), (2016.000, 0.0)

UNITS: person/person/year

Age_specific_fertility_rate_L[A104] = GRAPH(TIME)

Points: (2011.000, 0.0), (2011.33333333, 0.0), (2011.66666667, 0.0), (2012.000, 0.0), (2012.33333333, 0.0), (2012.66666667, 0.0), (2013.000, 0.0), (2013.33333333, 0.0), (2013.66666667, 0.0), (2014.000, 0.0), (2014.33333333, 0.0), (2014.66666667, 0.0), (2015.000, 0.0), (2015.33333333, 0.0), (2015.66666667, 0.0), (2016.000, 0.0)

UNITS: person/person/year

Age_specific_fertility_rate_M[A4] = GRAPH(TIME)

Points: (2011.00, 0.0), (2012.00, 0.0), (2013.00, 0.0), (2014.00, 0.0), (2015.00, 0.0), (2016.00, 0.0), (2017.00, 0.0), (2018.00, 0.0), (2019.00, 0.0), (2020.00, 0.0), (2021.00, 0.0), (2022.00, 0.0), (2023.00, 0.0), (2024.00, 0.0), (2025.00, 0.0), (2026.00, 0.0)

UNITS: person/person/year

Age_specific_fertility_rate_M[A9] = GRAPH(TIME)

Points: (2011.00, 0.0), (2012.00, 0.0), (2013.00, 0.0), (2014.00, 0.0), (2015.00, 0.0), (2016.00, 0.0), (2017.00, 0.0), (2018.00, 0.0), (2019.00, 0.0), (2020.00, 0.0), (2021.00, 0.0), (2022.00, 0.0), (2023.00, 0.0), (2024.00, 0.0), (2025.00, 0.0), (2026.00, 0.0)

UNITS: person/person/year

Age_specific_fertility_rate_M[A14] = GRAPH(TIME)

Points: (2011.00, 0.0), (2012.00, 0.0), (2013.00, 0.0), (2014.00, 0.0), (2015.00, 0.0), (2016.00, 0.0), (2017.00, 0.0), (2018.00, 0.0), (2019.00, 0.0), (2020.00, 0.0), (2021.00, 0.0), (2022.00, 0.0), (2023.00, 0.0), (2024.00, 0.0), (2025.00, 0.0), (2026.00, 0.0)

UNITS: person/person/year

Age_specific_fertility_rate_M[A19] = GRAPH(TIME)

Points: (2011.00, 16.1), (2012.00, 16.1), (2013.00, 14.6), (2014.00, 12.8), (2015.00, 11.9), (2016.00, 10.5), (2017.00, 13.23816), (2018.00, 13.0219), (2019.00, 12.80706), (2020.00, 12.59364), (2021.00, 12.3817), (2022.00, 12.17114), (2023.00, 11.96204), (2024.00, 11.75436), (2025.00, 11.5481), (2026.00, 11.3433)

UNITS: person/person/year

Age_specific_fertility_rate_M[A24] = GRAPH(TIME)

Points: (2011.00, 53.0), (2012.00, 53.1), (2013.00, 51.1), (2014.00, 47.3), (2015.00, 47.1), (2016.00, 44.6), (2017.00, 47.49754), (2018.00, 46.86844), (2019.00, 46.24306), (2020.00, 45.6215), (2021.00, 45.0037), (2022.00, 44.38968), (2023.00, 43.77946), (2024.00, 43.173), (2025.00, 42.57034), (2026.00, 41.97144)

Age_specific_fertility_rate_M[A29] = GRAPH(TIME)

Points: (2011.00, 103.1), (2012.00, 102.5), (2013.00, 98.9), (2014.00, 94.4), (2015.00, 92.9), (2016.00, 91.9), (2017.00, 98.22096), (2018.00, 97.2961), (2019.00, 96.37558), (2020.00, 95.4595), (2021.00, 94.54774), (2022.00, 93.6404), (2023.00, 92.7374), (2024.00, 91.8388), (2025.00, 90.94454), (2026.00, 90.05466)

UNITS: person/person/year

Age_specific_fertility_rate_M[A34] = GRAPH(TIME)

Points: (2011.00, 124.9), (2012.00, 126.8), (2013.00, 124.6), (2014.00, 120.2), (2015.00, 121.5), (2016.00, 123.4), (2017.00, 123.86218), (2018.00, 123.25704), (2019.00, 122.65226), (2020.00, 122.04788), (2021.00, 121.44386), (2022.00, 120.84016), (2023.00, 120.23686), (2024.00, 119.63392), (2025.00, 119.03136), (2026.00, 118.42914)

UNITS: person/person/year

Age_specific_fertility_rate_M[A39] = GRAPH(TIME)

Points: (2011.00, 70.30), (2012.00, 71.50), (2013.00, 70.80), (2014.00, 69.10), (2015.00, 69.60), (2016.00, 71.90), (2017.00, 74.1605), (2018.00, 74.4404), (2019.00, 74.71466), (2020.00, 74.98322), (2021.00, 75.2461), (2022.00, 75.50332), (2023.00, 75.75486), (2024.00, 76.00074), (2025.00, 76.2409), (2026.00, 76.47544)

UNITS: person/person/year

Age_specific_fertility_rate_M[A44] = GRAPH(TIME)

Points: (2011.00, 15.2), (2012.00, 15.2), (2013.00, 15.4), (2014.00, 14.4), (2015.00, 14.6), (2016.00, 15.3), (2017.00, 17.25968), (2018.00, 17.59466), (2019.00, 17.92584), (2020.00, 18.25324), (2021.00, 18.57682), (2022.00, 18.89662), (2023.00, 19.21258), (2024.00, 19.52478), (2025.00, 19.83322), (2026.00, 20.13782)

Age_specific_fertility_rate_M[A49] = GRAPH(TIME)

Points: (2011.00, 0.9), (2012.00, 1.0), (2013.00, 0.9), (2014.00, 1.0), (2015.00, 1.1), (2016.00, 1.2), (2017.00, 1.1896), (2018.00, 1.23578), (2019.00, 1.2815), (2020.00, 1.32676), (2021.00, 1.37148), (2022.00, 1.4158), (2023.00, 1.45962), (2024.00, 1.50298), (2025.00, 1.54582), (2026.00, 1.58824)

UNITS: person/person/year

DOCUMENT: Centre for Epidemiology and Evidence. Health Statistics New South Wales. Sydney:

Age_specific_fertility_rate_M[A54] = GRAPH(TIME)

Points: (2011.000, 0.0), (2011.33333333, 0.0), (2011.66666667, 0.0), (2012.000, 0.0), (2012.33333333, 0.0), (2012.66666667, 0.0), (2013.000, 0.0), (2013.33333333, 0.0), (2013.66666667, 0.0), (2014.000, 0.0), (2014.33333333, 0.0), (2014.66666667, 0.0), (2015.000, 0.0), (2015.33333333, 0.0), (2015.66666667, 0.0), (2016.000, 0.0)

UNITS: person/person/year

Age_specific_fertility_rate_M[A59] = GRAPH(TIME)

Points: (2011.000, 0.0), (2011.33333333, 0.0), (2011.66666667, 0.0), (2012.000, 0.0), (2012.33333333, 0.0), (2012.66666667, 0.0), (2013.000, 0.0), (2013.33333333, 0.0), (2013.66666667, 0.0), (2014.000, 0.0), (2014.33333333, 0.0), (2014.66666667, 0.0), (2015.000, 0.0), (2015.33333333, 0.0), (2015.66666667, 0.0), (2016.000, 0.0)

Age_specific_fertility_rate_M[A64] = GRAPH(TIME)

Points: (2011.000, 0.0), (2011.33333333, 0.0), (2011.66666667, 0.0), (2012.000, 0.0), (2012.33333333, 0.0), (2012.66666667, 0.0), (2013.000, 0.0), (2013.33333333, 0.0), (2013.66666667, 0.0), (2014.000, 0.0), (2014.33333333, 0.0), (2014.66666667, 0.0), (2015.000, 0.0), (2015.33333333, 0.0), (2015.66666667, 0.0), (2016.000, 0.0)

Age_specific_fertility_rate_M[A69] = GRAPH(TIME)

Points: (2011.000, 0.0), (2011.33333333, 0.0), (2011.66666667, 0.0), (2012.000, 0.0), (2012.33333333, 0.0), (2012.66666667, 0.0), (2013.000, 0.0), (2013.33333333, 0.0), (2013.66666667, 0.0), (2014.000, 0.0), (2014.33333333, 0.0), (2014.66666667, 0.0), (2015.000, 0.0), (2015.33333333, 0.0), (2015.66666667, 0.0), (2016.000, 0.0)

Age_specific_fertility_rate_M[A74] = GRAPH(TIME)

Points: (2011.000, 0.0), (2011.33333333, 0.0), (2011.66666667, 0.0), (2012.000, 0.0), (2012.33333333, 0.0), (2012.66666667, 0.0), (2013.000, 0.0), (2013.33333333, 0.0), (2013.66666667, 0.0), (2014.000, 0.0), (2014.33333333, 0.0), (2014.66666667, 0.0), (2015.000, 0.0), (2015.33333333, 0.0), (2015.66666667, 0.0), (2016.000, 0.0)

Age_specific_fertility_rate_M[A79] = GRAPH(TIME)

Points: (2011.000, 0.0), (2011.33333333, 0.0), (2011.66666667, 0.0), (2012.000, 0.0), (2012.33333333, 0.0), (2012.66666667, 0.0), (2013.000, 0.0), (2013.33333333, 0.0), (2013.66666667, 0.0), (2014.000, 0.0), (2014.33333333, 0.0), (2014.66666667, 0.0), (2015.000, 0.0), (2015.33333333, 0.0), (2015.66666667, 0.0), (2016.000, 0.0)

Age_specific_fertility_rate_M[A84] = GRAPH(TIME)

Points: (2011.000, 0.0), (2011.33333333, 0.0), (2011.66666667, 0.0), (2012.000, 0.0), (2012.33333333, 0.0), (2012.66666667, 0.0), (2013.000, 0.0), (2013.33333333, 0.0), (2013.66666667, 0.0), (2014.000, 0.0), (2014.33333333, 0.0), (2014.66666667, 0.0), (2015.000, 0.0), (2015.33333333, 0.0), (2015.66666667, 0.0), (2016.000, 0.0)

Age_specific_fertility_rate_M[A89] = GRAPH(TIME)

Points: (2011.000, 0.0), (2011.33333333, 0.0), (2011.66666667, 0.0), (2012.000, 0.0), (2012.33333333, 0.0), (2012.66666667, 0.0), (2013.000, 0.0), (2013.33333333, 0.0), (2013.66666667, 0.0), (2014.000, 0.0), (2014.33333333, 0.0), (2014.66666667, 0.0), (2015.000, 0.0), (2015.33333333, 0.0), (2015.66666667, 0.0), (2016.000, 0.0)

Age_specific_fertility_rate_M[A94] = GRAPH(TIME)

Points: (2011.000, 0.0), (2011.33333333, 0.0), (2011.66666667, 0.0), (2012.000, 0.0), (2012.33333333, 0.0), (2012.66666667, 0.0), (2013.000, 0.0), (2013.33333333, 0.0), (2013.66666667, 0.0), (2014.000, 0.0), (2014.33333333, 0.0), (2014.66666667, 0.0), (2015.000, 0.0), (2015.33333333, 0.0), (2015.66666667, 0.0), (2016.000, 0.0)

Age_specific_fertility_rate_M[A99] = GRAPH(TIME)

Points: (2011.000, 0.0), (2011.33333333, 0.0), (2011.66666667, 0.0), (2012.000, 0.0), (2012.33333333, 0.0), (2012.66666667, 0.0), (2013.000, 0.0), (2013.33333333, 0.0), (2013.66666667, 0.0), (2014.000, 0.0), (2014.33333333, 0.0), (2014.66666667, 0.0), (2015.000, 0.0), (2015.33333333, 0.0), (2015.66666667, 0.0), (2016.000, 0.0)

Age_specific_fertility_rate_M[A104] = GRAPH(TIME)

Points: (2011.000, 0.0), (2011.33333333, 0.0), (2011.66666667, 0.0), (2012.000, 0.0), (2012.33333333, 0.0), (2012.66666667, 0.0), (2013.000, 0.0), (2013.33333333, 0.0), (2013.66666667, 0.0), (2014.000, 0.0), (2014.33333333, 0.0), (2014.66666667, 0.0), (2015.000, 0.0), (2015.33333333, 0.0), (2015.66666667, 0.0), (2016.000, 0.0)

All_Deaths[Gender] = SUM(stock_cohort_deaths[Gender,*]) +stock_cohort_ageing_out[Gender,A104]

Births_per_stock_cohort[Age] = (Age_specific_Fertility_rate[Age])*(Persons_in_stock_cohort[F,Age]/1000)

UNITS: persons/year

cohort_length = 5 {DELAY CONVERTER}

UNITS: years

death_rate_H[M, A4] = GRAPH(TIME)

Points: (2011.00, 0.001), (2012.00, 0.0009), (2013.00, 0.0009), (2014.00, 0.0008), (2015.00, 0.0008), (2016.00, 0.0007), (2017.00, 0.001014), (2018.00, 0.000988), (2019.00, 0.000956), (2020.00, 0.000932), (2021.00, 0.000906), (2022.00, 0.00088), (2023.00, 0.000852), (2024.00, 0.000828), (2025.00, 0.000802), (2026.00, 0.000782), (2027.00, 0.000756), (2028.00, 0.000732), (2029.00, 0.000708), (2030.00, 0.000686), (2031.00, 0.000662), (2032.00, 0.000646), (2033.00, 0.000628), (2034.00, 0.000614), (2035.00, 0.000596), (2036.00, 0.000576), (2037.00, 0.00056), (2038.00, 0.000546), (2039.00, 0.000532), (2040.00, 0.000518), (2041.00, 0.000502), (2042.00, 0.00049), (2043.00, 0.000474), (2044.00, 0.000462), (2045.00, 0.00045), (2046.00, 0.000436), (2047.00, 0.000424), (2048.00, 0.000414), (2049.00, 0.000402), (2050.00, 0.000392), (2051.00, 0.000382), (2052.00, 0.00037), (2053.00, 0.00036), (2054.00, 0.00035), (2055.00, 0.00034), (2056.00, 0.000332), (2057.00, 0.000322), (2058.00, 0.000312), (2059.00, 0.000304), (2060.00, 0.000296), (2061.00, 0.00029)

UNITS: persons/person/year

DOCUMENT: Centre for Epidemiology and Evidence. Health Statistics New South Wales. Sydney: NSW Ministry of Health. Available at: www.healthstats.nsw.gov.au. Accessed 19/7/2018

death_rate_H[M, A9] = GRAPH(TIME)

Points: (2011.00, 0.0001), (2012.00, 0.0001), (2013.00, 0.0001), (2014.00, 0.0001), (2015.00, 0.0001), (2016.00, 0.0001), (2017.00, 0.000082), (2018.00, 0.000078), (2019.00, 0.000074), (2020.00, 0.00007), (2021.00, 0.000068), (2022.00, 0.000066), (2023.00, 0.00006), (2024.00, 0.000058), (2025.00, 0.000056), (2026.00, 0.000054), (2027.00, 0.000048), (2028.00, 0.000048), (2029.00, 0.000044), (2030.00, 0.000044), (2031.00, 0.000042), (2032.00, 0.000038), (2033.00, 0.000038), (2034.00, 0.000036), (2035.00, 0.000036), (2036.00, 0.000034), (2037.00, 0.000034), (2038.00, 0.000034), (2039.00, 0.000034), (2040.00, 0.000032), (2041.00, 0.000032), (2042.00, 0.000032), (2043.00, 0.00003), (2044.00, 0.000026), (2045.00, 0.000026), (2046.00, 0.000026), (2047.00, 0.000026), (2048.00, 0.000024), (2049.00, 0.000024), (2050.00, 0.000024), (2051.00, 0.000024), (2052.00, 0.000022), (2053.00, 0.000022), (2054.00, 0.000022), (2055.00, 0.00002), (2056.00, 0.00002), (2057.00, 0.00002), (2058.00, 0.00002), (2059.00, 0.00002), (2060.00, 0.00002), (2061.00, 0.00002)

UNITS: persons/person/year

death_rate_H[M, A14] = GRAPH(TIME)

Points: (2011.00, 0.0001), (2012.00, 0.0001), (2013.00, 0.0001), (2014.00, 0.0001), (2015.00, 0.0001), (2016.00, 0.0001), (2017.00, 0.000088), (2018.00, 0.000086), (2019.00, 0.00008), (2020.00, 0.000076), (2021.00, 0.000074), (2022.00, 0.00007), (2023.00, 0.000066), (2024.00, 0.000064), (2025.00, 0.00006), (2026.00, 0.000056), (2027.00, 0.000054), (2028.00, 0.00005), (2029.00, 0.000048), (2030.00, 0.000048), (2031.00, 0.00004), (2032.00, 0.00004), (2033.00, 0.00004), (2034.00, 0.000038), (2035.00, 0.000038), (2036.00, 0.000038), (2037.00, 0.000036), (2038.00, 0.000036), (2039.00, 0.000036), (2040.00, 0.000034), (2041.00, 0.000034), (2042.00, 0.000034), (2043.00, 0.00003), (2044.00, 0.000028), (2045.00, 0.000028), (2046.00, 0.000026), (2047.00, 0.000026), (2048.00, 0.000026), (2049.00, 0.000026), (2050.00, 0.000026), (2051.00, 0.000026), (2052.00, 0.000024), (2053.00, 0.000022), (2054.00, 0.000022), (2055.00, 0.000022), (2056.00, 0.000022), (2057.00, 0.000022), (2058.00, 0.000022), (2059.00, 0.000022), (2060.00, 0.000022), (2061.00, 0.000018)

death_rate_H[M, A19] = GRAPH(TIME)

Points: (2011.00, 0.0004), (2012.00, 0.0004), (2013.00, 0.0004), (2014.00, 0.0003), (2015.00, 0.0004), (2016.00, 0.0003), (2017.00, 0.000356), (2018.00, 0.00034), (2019.00, 0.000324), (2020.00, 0.000308), (2021.00, 0.000294), (2022.00, 0.000278), (2023.00, 0.000268), (2024.00, 0.000252), (2025.00, 0.000242), (2026.00, 0.000228), (2027.00, 0.00022), (2028.00, 0.000204), (2029.00, 0.000196), (2030.00, 0.000184), (2031.00, 0.000174), (2032.00, 0.00017), (2033.00, 0.000164), (2034.00, 0.000162), (2035.00, 0.000154), (2036.00, 0.000152), (2037.00, 0.000146), (2038.00, 0.000146), (2039.00, 0.000138), (2040.00, 0.000136), (2041.00, 0.000132), (2042.00, 0.000128), (2043.00, 0.000124), (2044.00, 0.000124), (2045.00, 0.000116), (2046.00, 0.000114), (2047.00, 0.000112), (2048.00, 0.000108), (2049.00, 0.000106), (2050.00, 0.000104), (2051.00, 0.000098), (2052.00, 0.000096), (2053.00, 0.000096), (2054.00, 0.000092), (2055.00, 0.000088), (2056.00, 0.000088), (2057.00, 0.000086), (2058.00, 0.000082), (2059.00, 0.000078), (2060.00, 0.000078), (2061.00, 0.000074)

UNITS: persons/person/year

death_rate_H[M, A24] = GRAPH(TIME)

Points: (2011.00, 0.0006), (2012.00, 0.0006), (2013.00, 0.0006), (2014.00, 0.0006), (2015.00, 0.0006), (2016.00, 0.0006), (2017.00, 0.000514), (2018.00, 0.000492), (2019.00, 0.000472), (2020.00, 0.000454), (2021.00, 0.000438), (2022.00, 0.000418), (2023.00, 0.0004), (2024.00, 0.00038), (2025.00, 0.000366), (2026.00, 0.00035), (2027.00, 0.000332), (2028.00, 0.00032), (2029.00, 0.000302), (2030.00, 0.00029), (2031.00, 0.000274), (2032.00, 0.00027), (2033.00, 0.00026), (2034.00, 0.00025), (2035.00, 0.000246), (2036.00, 0.00024), (2037.00, 0.000232), (2038.00, 0.000224), (2039.00, 0.00022), (2040.00, 0.000214), (2041.00, 0.00021), (2042.00, 0.000204), (2043.00, 0.000196), (2044.00, 0.000192), (2045.00, 0.000184), (2046.00, 0.000182), (2047.00, 0.000174), (2048.00, 0.000174), (2049.00, 0.000166), (2050.00, 0.000164), (2051.00, 0.000158), (2052.00, 0.000154), (2053.00, 0.000148), (2054.00, 0.000144), (2055.00, 0.000142), (2056.00, 0.000138), (2057.00, 0.000132), (2058.00, 0.00013), (2059.00, 0.000126), (2060.00, 0.000122), (2061.00, 0.00012)

death_rate_H[M, A29] = GRAPH(TIME)

Points: (2011.00, 0.0007), (2012.00, 0.0007), (2013.00, 0.0007), (2014.00, 0.0006), (2015.00, 0.0007), (2016.00, 0.0006), (2017.00, 0.00062), (2018.00, 0.000598), (2019.00, 0.00058), (2020.00, 0.000562), (2021.00, 0.000542), (2022.00, 0.000522), (2023.00, 0.000502), (2024.00, 0.000486), (2025.00, 0.00047), (2026.00, 0.000452), (2027.00, 0.000434), (2028.00, 0.00042), (2029.00, 0.000402), (2030.00, 0.000386), (2031.00, 0.000372), (2032.00, 0.000362), (2033.00, 0.000352), (2034.00, 0.000342), (2035.00, 0.000332), (2036.00, 0.000324), (2037.00, 0.000316), (2038.00, 0.000308), (2039.00, 0.000298), (2040.00, 0.00029), (2041.00, 0.000282), (2042.00, 0.000272), (2043.00, 0.000268), (2044.00, 0.00026), (2045.00, 0.00025), (2046.00, 0.000244), (2047.00, 0.00024), (2048.00, 0.000232), (2049.00, 0.000224), (2050.00, 0.00022), (2051.00, 0.000212), (2052.00, 0.000208), (2053.00, 0.000202), (2054.00, 0.000198), (2055.00, 0.000188), (2056.00, 0.000186), (2057.00, 0.000178), (2058.00, 0.000174), (2059.00, 0.00017), (2060.00, 0.000166), (2061.00, 0.000162)

UNITS: persons/person/year

death_rate_H[M, A34] = GRAPH(TIME)

Points: (2011.00, 0.0009), (2012.00, 0.0008), (2013.00, 0.0008), (2014.00, 0.0008), (2015.00, 0.0009), (2016.00, 0.0008), (2017.00, 0.000824), (2018.00, 0.000804), (2019.00, 0.000784), (2020.00, 0.000764), (2021.00, 0.000744), (2022.00, 0.000724), (2023.00, 0.000704), (2024.00, 0.000684), (2025.00, 0.000668), (2026.00, 0.00065), (2027.00, 0.00063), (2028.00, 0.00061), (2029.00, 0.00059), (2030.00, 0.000576), (2031.00, 0.00056), (2032.00, 0.000542), (2033.00, 0.00053), (2034.00, 0.000512), (2035.00, 0.0005), (2036.00, 0.000484), (2037.00, 0.000474), (2038.00, 0.000458), (2039.00, 0.000448), (2040.00, 0.000434), (2041.00, 0.000422), (2042.00, 0.000412), (2043.00, 0.0004), (2044.00, 0.00039), (2045.00, 0.00038), (2046.00, 0.000368), (2047.00, 0.000358), (2048.00, 0.000346), (2049.00, 0.000338), (2050.00, 0.000328), (2051.00, 0.00032), (2052.00, 0.00031), (2053.00, 0.000302), (2054.00, 0.000294), (2055.00, 0.000286), (2056.00, 0.000278), (2057.00, 0.00027), (2058.00, 0.000262), (2059.00, 0.000258), (2060.00, 0.00025), (2061.00, 0.00024)

UNITS: persons/person/year

death_rate_H[M, A39] = GRAPH(TIME)

Points: (2011.00, 0.0011), (2012.00, 0.0011), (2013.00, 0.0011), (2014.00, 0.0012), (2015.00, 0.0011), (2016.00, 0.001), (2017.00, 0.001092), (2018.00, 0.001072), (2019.00, 0.001052), (2020.00, 0.001034), (2021.00, 0.001014), (2022.00, 0.000994), (2023.00, 0.000976), (2024.00, 0.000956), (2025.00, 0.000936), (2026.00, 0.000918), (2027.00, 0.000898), (2028.00, 0.00088), (2029.00, 0.000862), (2030.00, 0.000842), (2031.00, 0.000822), (2032.00, 0.000802), (2033.00, 0.00078), (2034.00, 0.000758), (2035.00, 0.000736), (2036.00, 0.000716), (2037.00, 0.000696), (2038.00, 0.000682), (2039.00, 0.00066), (2040.00, 0.000642), (2041.00, 0.000624), (2042.00, 0.000606), (2043.00, 0.000588), (2044.00, 0.000574), (2045.00, 0.000558), (2046.00, 0.000542), (2047.00, 0.00053), (2048.00, 0.000512), (2049.00, 0.0005), (2050.00, 0.000484), (2051.00, 0.000474), (2052.00, 0.000458), (2053.00, 0.000446), (2054.00, 0.000434), (2055.00, 0.000422), (2056.00, 0.000412), (2057.00, 0.0004), (2058.00, 0.00039), (2059.00, 0.000378), (2060.00, 0.000368), (2061.00, 0.000356)

UNITS: persons/person/year

death_rate_H[M, A44] = GRAPH(TIME)

Points: (2011.00, 0.0015), (2012.00, 0.0015), (2013.00, 0.0015), (2014.00, 0.0016), (2015.00, 0.0017), (2016.00, 0.0014), (2017.00, 0.001492), (2018.00, 0.001472), (2019.00, 0.001452), (2020.00, 0.001434), (2021.00, 0.001414), (2022.00, 0.00139), (2023.00, 0.00137), (2024.00, 0.00135), (2025.00, 0.00133), (2026.00, 0.00131), (2027.00, 0.001288), (2028.00, 0.001268), (2029.00, 0.001248), (2030.00, 0.00123), (2031.00, 0.00121), (2032.00, 0.001176), (2033.00, 0.001144), (2034.00, 0.00111), (2035.00, 0.001082), (2036.00, 0.001052), (2037.00, 0.001024), (2038.00, 0.000996), (2039.00, 0.000966), (2040.00, 0.000942), (2041.00, 0.000914), (2042.00, 0.000892), (2043.00, 0.000868), (2044.00, 0.00084), (2045.00, 0.000818), (2046.00, 0.000796), (2047.00, 0.000774), (2048.00, 0.000754), (2049.00, 0.000732), (2050.00, 0.00071), (2051.00, 0.000692), (2052.00, 0.000672), (2053.00, 0.000654), (2054.00, 0.000636), (2055.00, 0.000622), (2056.00, 0.000602), (2057.00, 0.000586), (2058.00, 0.000568), (2059.00, 0.000552), (2060.00, 0.000542), (2061.00, 0.000524)

UNITS: persons/person/year

death_rate_H[M, A49] = GRAPH(TIME)

Points: (2011.00, 0.0023), (2012.00, 0.0022), (2013.00, 0.0023), (2014.00, 0.0022), (2015.00, 0.0023), (2016.00, 0.002), (2017.00, 0.002182), (2018.00, 0.002154), (2019.00, 0.002126), (2020.00, 0.002096), (2021.00, 0.00207), (2022.00, 0.002042), (2023.00, 0.00201), (2024.00, 0.00198), (2025.00, 0.001954), (2026.00, 0.001926), (2027.00, 0.001898), (2028.00, 0.001868), (2029.00, 0.00184), (2030.00, 0.00181), (2031.00, 0.001786), (2032.00, 0.001732), (2033.00, 0.001686), (2034.00, 0.001638), (2035.00, 0.001594), (2036.00, 0.001552), (2037.00, 0.001506), (2038.00, 0.001468), (2039.00, 0.001426), (2040.00, 0.001386), (2041.00, 0.00135), (2042.00, 0.001312), (2043.00, 0.001274), (2044.00, 0.001242), (2045.00, 0.001208), (2046.00, 0.001174), (2047.00, 0.001144), (2048.00, 0.00111), (2049.00, 0.00108), (2050.00, 0.00105), (2051.00, 0.001024), (2052.00, 0.000992), (2053.00, 0.000966), (2054.00, 0.00094), (2055.00, 0.000914), (2056.00, 0.00089), (2057.00, 0.000862), (2058.00, 0.00084), (2059.00, 0.000816), (2060.00, 0.000794), (2061.00, 0.000774)

UNITS: persons/person/year

death_rate_H[M, A54] = GRAPH(TIME)

Points: (2011.00, 0.0034), (2012.00, 0.0033), (2013.00, 0.0033), (2014.00, 0.0034), (2015.00, 0.0034), (2016.00, 0.0031), (2017.00, 0.003264), (2018.00, 0.003208), (2019.00, 0.003156), (2020.00, 0.003102), (2021.00, 0.003052), (2022.00, 0.002996), (2023.00, 0.002944), (2024.00, 0.002892), (2025.00, 0.00284), (2026.00, 0.002788), (2027.00, 0.002734), (2028.00, 0.002682), (2029.00, 0.00263), (2030.00, 0.002578), (2031.00, 0.002526), (2032.00, 0.002458), (2033.00, 0.00239), (2034.00, 0.002326), (2035.00, 0.00226), (2036.00, 0.002198), (2037.00, 0.002138), (2038.00, 0.00208), (2039.00, 0.002026), (2040.00, 0.001968), (2041.00, 0.001914), (2042.00, 0.00186), (2043.00, 0.001808), (2044.00, 0.00176), (2045.00, 0.00171), (2046.00, 0.001666), (2047.00, 0.001618), (2048.00, 0.001574), (2049.00, 0.001532), (2050.00, 0.001488), (2051.00, 0.001448), (2052.00, 0.00141), (2053.00, 0.001368), (2054.00, 0.00133), (2055.00, 0.001298), (2056.00, 0.001262), (2057.00, 0.001226), (2058.00, 0.001192), (2059.00, 0.001158), (2060.00, 0.001128), (2061.00, 0.001094)

UNITS: persons/person/year

death_rate_H[M, A59] = GRAPH(TIME)

Points: (2011.00, 0.0053), (2012.00, 0.005), (2013.00, 0.0051), (2014.00, 0.0051), (2015.00, 0.0052), (2016.00, 0.0047), (2017.00, 0.00471), (2018.00, 0.004596), (2019.00, 0.004486), (2020.00, 0.004378), (2021.00, 0.004274), (2022.00, 0.004164), (2023.00, 0.004056), (2024.00, 0.003952), (2025.00, 0.00385), (2026.00, 0.003746), (2027.00, 0.003642), (2028.00, 0.003542), (2029.00, 0.003442), (2030.00, 0.003348), (2031.00, 0.003254), (2032.00, 0.003164), (2033.00, 0.003076), (2034.00, 0.002994), (2035.00, 0.002912), (2036.00, 0.002832), (2037.00, 0.002754), (2038.00, 0.002676), (2039.00, 0.002602), (2040.00, 0.002532), (2041.00, 0.002466), (2042.00, 0.002394), (2043.00, 0.00233), (2044.00, 0.002266), (2045.00, 0.002204), (2046.00, 0.00214), (2047.00, 0.002084), (2048.00, 0.002028), (2049.00, 0.00197), (2050.00, 0.001918), (2051.00, 0.001866), (2052.00, 0.001814), (2053.00, 0.001762), (2054.00, 0.001716), (2055.00, 0.001666), (2056.00, 0.001622), (2057.00, 0.001578), (2058.00, 0.001534), (2059.00, 0.001492), (2060.00, 0.001452), (2061.00, 0.001412)

death_rate_H[M, A64] = GRAPH(TIME)

Points: (2011.00, 0.0081), (2012.00, 0.0077), (2013.00, 0.0077), (2014.00, 0.0078), (2015.00, 0.0078), (2016.00, 0.0071), (2017.00, 0.007028), (2018.00, 0.00681), (2019.00, 0.006598), (2020.00, 0.006392), (2021.00, 0.006194), (2022.00, 0.005986), (2023.00, 0.005788), (2024.00, 0.005596), (2025.00, 0.005404), (2026.00, 0.005226), (2027.00, 0.005038), (2028.00, 0.004856), (2029.00, 0.004682), (2030.00, 0.004512), (2031.00, 0.004348), (2032.00, 0.004228), (2033.00, 0.004112), (2034.00, 0.004), (2035.00, 0.00389), (2036.00, 0.003786), (2037.00, 0.00368), (2038.00, 0.003578), (2039.00, 0.003482), (2040.00, 0.003386), (2041.00, 0.003294), (2042.00, 0.003202), (2043.00, 0.003114), (2044.00, 0.00303), (2045.00, 0.002946), (2046.00, 0.002864), (2047.00, 0.002788), (2048.00, 0.00271), (2049.00, 0.002636), (2050.00, 0.00256), (2051.00, 0.002492), (2052.00, 0.002426), (2053.00, 0.002358), (2054.00, 0.002294), (2055.00, 0.002228), (2056.00, 0.002168), (2057.00, 0.002108), (2058.00, 0.002052), (2059.00, 0.001994), (2060.00, 0.00194), (2061.00, 0.001886)

death_rate_H[M, A69] = GRAPH(TIME)

Points: (2011.00, 0.0127), (2012.00, 0.0122), (2013.00, 0.0126), (2014.00, 0.0121), (2015.00, 0.0117), (2016.00, 0.0108), (2017.00, 0.011196), (2018.00, 0.010794), (2019.00, 0.010408), (2020.00, 0.010034), (2021.00, 0.009678), (2022.00, 0.009304), (2023.00, 0.008948), (2024.00, 0.008606), (2025.00, 0.008274), (2026.00, 0.007956), (2027.00, 0.007626), (2028.00, 0.007308), (2029.00, 0.007006), (2030.00, 0.006716), (2031.00, 0.006436), (2032.00, 0.006258), (2033.00, 0.00609), (2034.00, 0.005922), (2035.00, 0.005756), (2036.00, 0.0056), (2037.00, 0.005448), (2038.00, 0.0053), (2039.00, 0.005152), (2040.00, 0.00501), (2041.00, 0.004874), (2042.00, 0.00474), (2043.00, 0.00461), (2044.00, 0.004484), (2045.00, 0.00436), (2046.00, 0.00424), (2047.00, 0.004126), (2048.00, 0.00401), (2049.00, 0.003902), (2050.00, 0.003794), (2051.00, 0.00369), (2052.00, 0.003588), (2053.00, 0.00349), (2054.00, 0.003394), (2055.00, 0.003304), (2056.00, 0.003212), (2057.00, 0.003124), (2058.00, 0.003038), (2059.00, 0.002954), (2060.00, 0.002872), (2061.00, 0.002794)

death_rate_H[M, A74] = GRAPH(TIME)

Points: (2011.00, 0.0212), (2012.00, 0.0203), (2013.00, 0.0202), (2014.00, 0.0198), (2015.00, 0.0193), (2016.00, 0.0182), (2017.00, 0.01864), (2018.00, 0.018006), (2019.00, 0.017394), (2020.00, 0.016804), (2021.00, 0.016234), (2022.00, 0.01564), (2023.00, 0.015066), (2024.00, 0.01452), (2025.00, 0.01399), (2026.00, 0.013478), (2027.00, 0.012946), (2028.00, 0.012436), (2029.00, 0.01194), (2030.00, 0.011474), (2031.00, 0.011018), (2032.00, 0.010716), (2033.00, 0.010422), (2034.00, 0.010136), (2035.00, 0.009862), (2036.00, 0.00959), (2037.00, 0.009328), (2038.00, 0.009074), (2039.00, 0.008826), (2040.00, 0.008586), (2041.00, 0.00835), (2042.00, 0.008118), (2043.00, 0.007898), (2044.00, 0.007682), (2045.00, 0.00747), (2046.00, 0.007266), (2047.00, 0.007066), (2048.00, 0.006874), (2049.00, 0.006686), (2050.00, 0.006504), (2051.00, 0.006326), (2052.00, 0.00615), (2053.00, 0.005982), (2054.00, 0.005818), (2055.00, 0.005658), (2056.00, 0.005504), (2057.00, 0.005354), (2058.00, 0.005206), (2059.00, 0.005064), (2060.00, 0.004924), (2061.00, 0.004788)

death_rate_H[M, A79] = GRAPH(TIME)

Points: (2011.00, 0.0378), (2012.00, 0.036), (2013.00, 0.0346), (2014.00, 0.0343), (2015.00, 0.0336), (2016.00, 0.0314), (2017.00, 0.032622), (2018.00, 0.03167), (2019.00, 0.030742), (2020.00, 0.029842), (2021.00, 0.028964), (2022.00, 0.028056), (2023.00, 0.027174), (2024.00, 0.02632), (2025.00, 0.025494), (2026.00, 0.02469), (2027.00, 0.02385), (2028.00, 0.023038), (2029.00, 0.022252), (2030.00, 0.021494), (2031.00, 0.02076), (2032.00, 0.020196), (2033.00, 0.019644), (2034.00, 0.01911), (2035.00, 0.018592), (2036.00, 0.018086), (2037.00, 0.017594), (2038.00, 0.017114), (2039.00, 0.016644), (2040.00, 0.016192), (2041.00, 0.015752), (2042.00, 0.01532), (2043.00, 0.014906), (2044.00, 0.014498), (2045.00, 0.014102), (2046.00, 0.013716), (2047.00, 0.013344), (2048.00, 0.01298), (2049.00, 0.012624), (2050.00, 0.01228), (2051.00, 0.011942), (2052.00, 0.011618), (2053.00, 0.0113), (2054.00, 0.01099), (2055.00, 0.01069), (2056.00, 0.010398), (2057.00, 0.010114), (2058.00, 0.009838), (2059.00, 0.009566), (2060.00, 0.009306), (2061.00, 0.009052)

death_rate_H[M, A84] = GRAPH(TIME)

Points: (2011.00, 0.0677), (2012.00, 0.0662), (2013.00, 0.0633), (2014.00, 0.0632), (2015.00, 0.0618), (2016.00, 0.0587), (2017.00, 0.06068), (2018.00, 0.059348), (2019.00, 0.05804), (2020.00, 0.056764), (2021.00, 0.055514), (2022.00, 0.0542), (2023.00, 0.05292), (2024.00, 0.051666), (2025.00, 0.050446), (2026.00, 0.04925), (2027.00, 0.04799), (2028.00, 0.046762), (2029.00, 0.04556), (2030.00, 0.044392), (2031.00, 0.043252), (2032.00, 0.04209), (2033.00, 0.040958), (2034.00, 0.039856), (2035.00, 0.038782), (2036.00, 0.037736), (2037.00, 0.036718), (2038.00, 0.035728), (2039.00, 0.034764), (2040.00, 0.033826), (2041.00, 0.032912), (2042.00, 0.032022), (2043.00, 0.031158), (2044.00, 0.030312), (2045.00, 0.029494), (2046.00, 0.028694), (2047.00, 0.027916), (2048.00, 0.027158), (2049.00, 0.026422), (2050.00, 0.025708), (2051.00, 0.02501), (2052.00, 0.02433), (2053.00, 0.02367), (2054.00, 0.023026), (2055.00, 0.022402), (2056.00, 0.021792), (2057.00, 0.0212), (2058.00, 0.020624), (2059.00, 0.020062), (2060.00, 0.019514), (2061.00, 0.018986)

death_rate_H[M, A89] = GRAPH(TIME)

Points: (2011.00, 0.1222), (2012.00, 0.1204), (2013.00, 0.1151), (2014.00, 0.1141), (2015.00, 0.1155), (2016.00, 0.1079), (2017.00, 0.109464), (2018.00, 0.107906), (2019.00, 0.106372), (2020.00, 0.104856), (2021.00, 0.10336), (2022.00, 0.101778), (2023.00, 0.10022), (2024.00, 0.098686), (2025.00, 0.097172), (2026.00, 0.095682), (2027.00, 0.094094), (2028.00, 0.092532), (2029.00, 0.090992), (2030.00, 0.08948), (2031.00, 0.087992), (2032.00, 0.08568), (2033.00, 0.083426), (2034.00, 0.08123), (2035.00, 0.079088), (2036.00, 0.077), (2037.00, 0.074964), (2038.00, 0.072984), (2039.00, 0.071052), (2040.00, 0.069166), (2041.00, 0.06733), (2042.00, 0.065544), (2043.00, 0.063802), (2044.00, 0.062104), (2045.00, 0.060452), (2046.00, 0.058838), (2047.00, 0.05727), (2048.00, 0.05574), (2049.00, 0.054252), (2050.00, 0.0528), (2051.00, 0.05139), (2052.00, 0.05001), (2053.00, 0.04867), (2054.00, 0.047366), (2055.00, 0.046094), (2056.00, 0.044856), (2057.00, 0.043654), (2058.00, 0.042478), (2059.00, 0.041336), (2060.00, 0.040226), (2061.00, 0.039142)

death_rate_H[M, A94] = GRAPH(TIME)

Points: (2011.00, 0.2109), (2012.00, 0.2079), (2013.00, 0.1979), (2014.00, 0.2001), (2015.00, 0.1973), (2016.00, 0.1836), (2017.00, 0.178732), (2018.00, 0.177646), (2019.00, 0.176562), (2020.00, 0.175488), (2021.00, 0.174422), (2022.00, 0.173284), (2023.00, 0.172156), (2024.00, 0.171034), (2025.00, 0.169922), (2026.00, 0.168816), (2027.00, 0.167628), (2028.00, 0.166452), (2029.00, 0.165282), (2030.00, 0.16412), (2031.00, 0.162968), (2032.00, 0.158854), (2033.00, 0.154836), (2034.00, 0.150914), (2035.00, 0.147078), (2036.00, 0.143332), (2037.00, 0.139676), (2038.00, 0.136106), (2039.00, 0.13262), (2040.00, 0.129216), (2041.00, 0.125898), (2042.00, 0.122656), (2043.00, 0.119492), (2044.00, 0.116404), (2045.00, 0.113392), (2046.00, 0.110454), (2047.00, 0.107586), (2048.00, 0.104786), (2049.00, 0.102056), (2050.00, 0.099396), (2051.00, 0.0968), (2052.00, 0.094268), (2053.00, 0.091798), (2054.00, 0.089394), (2055.00, 0.087046), (2056.00, 0.08476), (2057.00, 0.082528), (2058.00, 0.080354), (2059.00, 0.078234), (2060.00, 0.076166), (2061.00, 0.074152)

death_rate_H[M, A99] = GRAPH(TIME)

Points: (2011.00, 0.3405), (2012.00, 0.3461), (2013.00, 0.3114), (2014.00, 0.3098), (2015.00, 0.3121), (2016.00, 0.2776), (2017.00, 0.257168), (2018.00, 0.257156), (2019.00, 0.257148), (2020.00, 0.25714), (2021.00, 0.257128), (2022.00, 0.25712), (2023.00, 0.25711), (2024.00, 0.2571), (2025.00, 0.25709), (2026.00, 0.25708), (2027.00, 0.257068), (2028.00, 0.257058), (2029.00, 0.257048), (2030.00, 0.257036), (2031.00, 0.257026), (2032.00, 0.250852), (2033.00, 0.244802), (2034.00, 0.23888), (2035.00, 0.233082), (2036.00, 0.227404), (2037.00, 0.221852), (2038.00, 0.216416), (2039.00, 0.211096), (2040.00, 0.205896), (2041.00, 0.200806), (2042.00, 0.195826), (2043.00, 0.190962), (2044.00, 0.186202), (2045.00, 0.181554), (2046.00, 0.177004), (2047.00, 0.172562), (2048.00, 0.16822), (2049.00, 0.163972), (2050.00, 0.159828), (2051.00, 0.155778), (2052.00, 0.151822), (2053.00, 0.14796), (2054.00, 0.144186), (2055.00, 0.140504), (2056.00, 0.136904), (2057.00, 0.133396), (2058.00, 0.129968), (2059.00, 0.126622), (2060.00, 0.123358), (2061.00, 0.120172)

death_rate_H[M, A104] = GRAPH(TIME)

Points: (2011.00, 0.4131), (2012.00, 0.4072), (2013.00, 0.3934), (2014.00, 0.4316), (2015.00, 0.4668), (2016.00, 0.5088), (2017.00, 0.646455), (2018.00, 0.646455), (2019.00, 0.646455), (2020.00, 0.64645), (2021.00, 0.64645), (2022.00, 0.646445), (2023.00, 0.646445), (2024.00, 0.646445), (2025.00, 0.64644), (2026.00, 0.64644), (2027.00, 0.646435), (2028.00, 0.646435), (2029.00, 0.646435), (2030.00, 0.64643), (2031.00, 0.64643), (2032.00, 0.64298), (2033.00, 0.639595), (2034.00, 0.636285), (2035.00, 0.633035), (2036.00, 0.62985), (2037.00, 0.626735), (2038.00, 0.62368), (2039.00, 0.62069), (2040.00, 0.617765), (2041.00, 0.6149), (2042.00, 0.612095), (2043.00, 0.60935), (2044.00, 0.606665), (2045.00, 0.60404), (2046.00, 0.60147), (2047.00, 0.598955), (2048.00, 0.596495), (2049.00, 0.594095), (2050.00, 0.591745), (2051.00, 0.58945), (2052.00, 0.587205), (2053.00, 0.58501), (2054.00, 0.582865), (2055.00, 0.580775), (2056.00, 0.57873), (2057.00, 0.57673), (2058.00, 0.57478), (2059.00, 0.57287), (2060.00, 0.57101), (2061.00, 0.569195)

death_rate_H[F, A4] = GRAPH(TIME)

Points: (2011.00, 0.0008), (2012.00, 0.0008), (2013.00, 0.0008), (2014.00, 0.0008), (2015.00, 0.0007), (2016.00, 0.0006), (2017.00, 0.000732), (2018.00, 0.000712), (2019.00, 0.000692), (2020.00, 0.000672), (2021.00, 0.000654), (2022.00, 0.000634), (2023.00, 0.000616), (2024.00, 0.000596), (2025.00, 0.000578), (2026.00, 0.00056), (2027.00, 0.000544), (2028.00, 0.000526), (2029.00, 0.000506), (2030.00, 0.000492), (2031.00, 0.000476), (2032.00, 0.000464), (2033.00, 0.00045), (2034.00, 0.000442), (2035.00, 0.000432), (2036.00, 0.000422), (2037.00, 0.000414), (2038.00, 0.000404), (2039.00, 0.000394), (2040.00, 0.000386), (2041.00, 0.000378), (2042.00, 0.000368), (2043.00, 0.000358), (2044.00, 0.00035), (2045.00, 0.000342), (2046.00, 0.000334), (2047.00, 0.000328), (2048.00, 0.000322), (2049.00, 0.000314), (2050.00, 0.000304), (2051.00, 0.000298), (2052.00, 0.00029), (2053.00, 0.000286), (2054.00, 0.00028), (2055.00, 0.00027), (2056.00, 0.000264), (2057.00, 0.00026), (2058.00, 0.000254), (2059.00, 0.000248), (2060.00, 0.000242), (2061.00, 0.000236)

death_rate_H[F, A9] = GRAPH(TIME)

Points: (2011.00, 0.0001), (2012.00, 0.0001), (2013.00, 0.0001), (2014.00, 0.0001), (2015.00, 0.0001), (2016.00, 0.0001), (2017.00, 0.000056), (2018.00, 0.000056), (2019.00, 0.000056), (2020.00, 0.000052), (2021.00, 0.000052), (2022.00, 0.000046), (2023.00, 0.000044), (2024.00, 0.000042), (2025.00, 0.000042), (2026.00, 0.000038), (2027.00, 0.000034), (2028.00, 0.000034), (2029.00, 0.000032), (2030.00, 0.000032), (2031.00, 0.000032), (2032.00, 0.000032), (2033.00, 0.00003), (2034.00, 0.000024), (2035.00, 0.000024), (2036.00, 0.000024), (2037.00, 0.000024), (2038.00, 0.000024), (2039.00, 0.000024), (2040.00, 0.000024), (2041.00, 0.000022), (2042.00, 0.000022), (2043.00, 0.000022), (2044.00, 0.000022), (2045.00, 0.000022), (2046.00, 0.000022), (2047.00, 0.00002), (2048.00, 0.00002), (2049.00, 0.00002), (2050.00, 0.00002), (2051.00, 0.00002), (2052.00, 0.00002), (2053.00, 0.00002), (2054.00, 0.00002), (2055.00, 0.00002), (2056.00, 0.000014), (2057.00, 0.000014), (2058.00, 0.000014), (2059.00, 0.000014), (2060.00, 0.000014), (2061.00, 0.000014)

death_rate_H[F, A14] = GRAPH(TIME)

Points: (2011.00, 0.0001), (2012.00, 0.0001), (2013.00, 0.0001), (2014.00, 0.0001), (2015.00, 0.0001), (2016.00, 0.0001), (2017.00, 0.00007), (2018.00, 0.000064), (2019.00, 0.000064), (2020.00, 0.00006), (2021.00, 0.000058), (2022.00, 0.000054), (2023.00, 0.00005), (2024.00, 0.00005), (2025.00, 0.000048), (2026.00, 0.000046), (2027.00, 0.00004), (2028.00, 0.00004), (2029.00, 0.000038), (2030.00, 0.000036), (2031.00, 0.000036), (2032.00, 0.000034), (2033.00, 0.000034), (2034.00, 0.000032), (2035.00, 0.000028), (2036.00, 0.000028), (2037.00, 0.000028), (2038.00, 0.000028), (2039.00, 0.000028), (2040.00, 0.000028), (2041.00, 0.000028), (2042.00, 0.000026), (2043.00, 0.000026), (2044.00, 0.000026), (2045.00, 0.000024), (2046.00, 0.000024), (2047.00, 0.000022), (2048.00, 0.000022), (2049.00, 0.000022), (2050.00, 0.000022), (2051.00, 0.000022), (2052.00, 0.000022), (2053.00, 0.000022), (2054.00, 0.000022), (2055.00, 0.000022), (2056.00, 0.000022), (2057.00, 0.000018), (2058.00, 0.000018), (2059.00, 0.000018), (2060.00, 0.000016), (2061.00, 0.000016)

death_rate_H[F, A19] = GRAPH(TIME)

Points: (2011.00, 0.0002), (2012.00, 0.0002), (2013.00, 0.0002), (2014.00, 0.0002), (2015.00, 0.0002), (2016.00, 0.0002), (2017.00, 0.00017), (2018.00, 0.000164), (2019.00, 0.000156), (2020.00, 0.000148), (2021.00, 0.000146), (2022.00, 0.000136), (2023.00, 0.000132), (2024.00, 0.000126), (2025.00, 0.00012), (2026.00, 0.000114), (2027.00, 0.00011), (2028.00, 0.000104), (2029.00, 0.0001), (2030.00, 0.000094), (2031.00, 0.000088), (2032.00, 0.000088), (2033.00, 0.000086), (2034.00, 0.000086), (2035.00, 0.00008), (2036.00, 0.000078), (2037.00, 0.000078), (2038.00, 0.000078), (2039.00, 0.000076), (2040.00, 0.00007), (2041.00, 0.00007), (2042.00, 0.00007), (2043.00, 0.00007), (2044.00, 0.000066), (2045.00, 0.000066), (2046.00, 0.000062), (2047.00, 0.00006), (2048.00, 0.00006), (2049.00, 0.00006), (2050.00, 0.000058), (2051.00, 0.000058), (2052.00, 0.000056), (2053.00, 0.000056), (2054.00, 0.000054), (2055.00, 0.00005), (2056.00, 0.000048), (2057.00, 0.000048), (2058.00, 0.000048), (2059.00, 0.000046), (2060.00, 0.000046), (2061.00, 0.000046)

death_rate_H[F, A24] = GRAPH(TIME)

Points: (2011.00, 0.0003), (2012.00, 0.0003), (2013.00, 0.0002), (2014.00, 0.0002), (2015.00, 0.0002), (2016.00, 0.0002), (2017.00, 0.000216), (2018.00, 0.000212), (2019.00, 0.000204), (2020.00, 0.0002), (2021.00, 0.000194), (2022.00, 0.000184), (2023.00, 0.000182), (2024.00, 0.000174), (2025.00, 0.000172), (2026.00, 0.000164), (2027.00, 0.000158), (2028.00, 0.000152), (2029.00, 0.000144), (2030.00, 0.000142), (2031.00, 0.000134), (2032.00, 0.000134), (2033.00, 0.000132), (2034.00, 0.000126), (2035.00, 0.000124), (2036.00, 0.000122), (2037.00, 0.00012), (2038.00, 0.000114), (2039.00, 0.000114), (2040.00, 0.000112), (2041.00, 0.000108), (2042.00, 0.000104), (2043.00, 0.000104), (2044.00, 0.0001), (2045.00, 0.000098), (2046.00, 0.000096), (2047.00, 0.000094), (2048.00, 0.000092), (2049.00, 0.00009), (2050.00, 0.000088), (2051.00, 0.000086), (2052.00, 0.000084), (2053.00, 0.000082), (2054.00, 0.000082), (2055.00, 0.000078), (2056.00, 0.000078), (2057.00, 0.000074), (2058.00, 0.000074), (2059.00, 0.000072), (2060.00, 0.00007), (2061.00, 0.000068)

death_rate_H[F, A29] = GRAPH(TIME)

Points: (2011.00, 0.0003), (2012.00, 0.0003), (2013.00, 0.0003), (2014.00, 0.0003), (2015.00, 0.0003), (2016.00, 0.0003), (2017.00, 0.00027), (2018.00, 0.000268), (2019.00, 0.000258), (2020.00, 0.000254), (2021.00, 0.000248), (2022.00, 0.000238), (2023.00, 0.000238), (2024.00, 0.000228), (2025.00, 0.000224), (2026.00, 0.000218), (2027.00, 0.000212), (2028.00, 0.000208), (2029.00, 0.000198), (2030.00, 0.000196), (2031.00, 0.000188), (2032.00, 0.000186), (2033.00, 0.000182), (2034.00, 0.000176), (2035.00, 0.000172), (2036.00, 0.000166), (2037.00, 0.000164), (2038.00, 0.000162), (2039.00, 0.000156), (2040.00, 0.000154), (2041.00, 0.00015), (2042.00, 0.000146), (2043.00, 0.000144), (2044.00, 0.000142), (2045.00, 0.000138), (2046.00, 0.000132), (2047.00, 0.000132), (2048.00, 0.000128), (2049.00, 0.000122), (2050.00, 0.000122), (2051.00, 0.000122), (2052.00, 0.000114), (2053.00, 0.000112), (2054.00, 0.000112), (2055.00, 0.000112), (2056.00, 0.000106), (2057.00, 0.000104), (2058.00, 0.000102), (2059.00, 0.0001), (2060.00, 0.000096), (2061.00, 0.000096)

death_rate_H[F, A34] = GRAPH(TIME)

Points: (2011.00, 0.0004), (2012.00, 0.0004), (2013.00, 0.0004), (2014.00, 0.0004), (2015.00, 0.0004), (2016.00, 0.0004), (2017.00, 0.000368), (2018.00, 0.000366), (2019.00, 0.000358), (2020.00, 0.000348), (2021.00, 0.000346), (2022.00, 0.000338), (2023.00, 0.000328), (2024.00, 0.000326), (2025.00, 0.000318), (2026.00, 0.000314), (2027.00, 0.000306), (2028.00, 0.000298), (2029.00, 0.000294), (2030.00, 0.000286), (2031.00, 0.00028), (2032.00, 0.000274), (2033.00, 0.000268), (2034.00, 0.000262), (2035.00, 0.000256), (2036.00, 0.00025), (2037.00, 0.000244), (2038.00, 0.00024), (2039.00, 0.000234), (2040.00, 0.000226), (2041.00, 0.000224), (2042.00, 0.000216), (2043.00, 0.000212), (2044.00, 0.000206), (2045.00, 0.000202), (2046.00, 0.000198), (2047.00, 0.000194), (2048.00, 0.000188), (2049.00, 0.000186), (2050.00, 0.00018), (2051.00, 0.000176), (2052.00, 0.000176), (2053.00, 0.000166), (2054.00, 0.000166), (2055.00, 0.000162), (2056.00, 0.000156), (2057.00, 0.000154), (2058.00, 0.000152), (2059.00, 0.000146), (2060.00, 0.000142), (2061.00, 0.000142)

death_rate_H[F, A39] = GRAPH(TIME)

Points: (2011.00, 0.0006), (2012.00, 0.0006), (2013.00, 0.0006), (2014.00, 0.0006), (2015.00, 0.0006), (2016.00, 0.0005), (2017.00, 0.000566), (2018.00, 0.000558), (2019.00, 0.000552), (2020.00, 0.000544), (2021.00, 0.000534), (2022.00, 0.000528), (2023.00, 0.00052), (2024.00, 0.000514), (2025.00, 0.000504), (2026.00, 0.000496), (2027.00, 0.00049), (2028.00, 0.00048), (2029.00, 0.000474), (2030.00, 0.000466), (2031.00, 0.000458), (2032.00, 0.000446), (2033.00, 0.000436), (2034.00, 0.000428), (2035.00, 0.000418), (2036.00, 0.000408), (2037.00, 0.000398), (2038.00, 0.00039), (2039.00, 0.000382), (2040.00, 0.000372), (2041.00, 0.000364), (2042.00, 0.000356), (2043.00, 0.000346), (2044.00, 0.00034), (2045.00, 0.000334), (2046.00, 0.000324), (2047.00, 0.000316), (2048.00, 0.000312), (2049.00, 0.000304), (2050.00, 0.000296), (2051.00, 0.00029), (2052.00, 0.000282), (2053.00, 0.000276), (2054.00, 0.000272), (2055.00, 0.000262), (2056.00, 0.000256), (2057.00, 0.000252), (2058.00, 0.000246), (2059.00, 0.000238), (2060.00, 0.000236), (2061.00, 0.00023)

death_rate_H[F, A44] = GRAPH(TIME)

Points: (2011.00, 0.0009), (2012.00, 0.0009), (2013.00, 0.0009), (2014.00, 0.0009), (2015.00, 0.0009), (2016.00, 0.0009), (2017.00, 0.000898), (2018.00, 0.000888), (2019.00, 0.000876), (2020.00, 0.000866), (2021.00, 0.000856), (2022.00, 0.000846), (2023.00, 0.000834), (2024.00, 0.000824), (2025.00, 0.000814), (2026.00, 0.000802), (2027.00, 0.00079), (2028.00, 0.00078), (2029.00, 0.00077), (2030.00, 0.000758), (2031.00, 0.00075), (2032.00, 0.000732), (2033.00, 0.000714), (2034.00, 0.000698), (2035.00, 0.000682), (2036.00, 0.000668), (2037.00, 0.000652), (2038.00, 0.000636), (2039.00, 0.000622), (2040.00, 0.000608), (2041.00, 0.000596), (2042.00, 0.00058), (2043.00, 0.000568), (2044.00, 0.000554), (2045.00, 0.000542), (2046.00, 0.000532), (2047.00, 0.00052), (2048.00, 0.000506), (2049.00, 0.000496), (2050.00, 0.000484), (2051.00, 0.000472), (2052.00, 0.000462), (2053.00, 0.000452), (2054.00, 0.00044), (2055.00, 0.00043), (2056.00, 0.000422), (2057.00, 0.000412), (2058.00, 0.000402), (2059.00, 0.000392), (2060.00, 0.000386), (2061.00, 0.000376)

death_rate_H[F, A49] = GRAPH(TIME)

Points: (2011.00, 0.0014), (2012.00, 0.0013), (2013.00, 0.0014), (2014.00, 0.0014), (2015.00, 0.0014), (2016.00, 0.0013), (2017.00, 0.001374), (2018.00, 0.001354), (2019.00, 0.001336), (2020.00, 0.001316), (2021.00, 0.001296), (2022.00, 0.001276), (2023.00, 0.001258), (2024.00, 0.00124), (2025.00, 0.001222), (2026.00, 0.001202), (2027.00, 0.001182), (2028.00, 0.00116), (2029.00, 0.001142), (2030.00, 0.001122), (2031.00, 0.001104), (2032.00, 0.00108), (2033.00, 0.001054), (2034.00, 0.00103), (2035.00, 0.001006), (2036.00, 0.000982), (2037.00, 0.000964), (2038.00, 0.00094), (2039.00, 0.00092), (2040.00, 0.0009), (2041.00, 0.000878), (2042.00, 0.000856), (2043.00, 0.000838), (2044.00, 0.000818), (2045.00, 0.0008), (2046.00, 0.000782), (2047.00, 0.000764), (2048.00, 0.00075), (2049.00, 0.00073), (2050.00, 0.000714), (2051.00, 0.000696), (2052.00, 0.000682), (2053.00, 0.000666), (2054.00, 0.00065), (2055.00, 0.000636), (2056.00, 0.00062), (2057.00, 0.00061), (2058.00, 0.00059), (2059.00, 0.00058), (2060.00, 0.000566), (2061.00, 0.000554)

UNITS: persons/person/year

death_rate_H[F, A54] = GRAPH(TIME)

Points: (2011.00, 0.0022), (2012.00, 0.002), (2013.00, 0.0021), (2014.00, 0.002), (2015.00, 0.0021), (2016.00, 0.0019), (2017.00, 0.001988), (2018.00, 0.00195), (2019.00, 0.001908), (2020.00, 0.001872), (2021.00, 0.001836), (2022.00, 0.001794), (2023.00, 0.001756), (2024.00, 0.001716), (2025.00, 0.001682), (2026.00, 0.001646), (2027.00, 0.001608), (2028.00, 0.00157), (2029.00, 0.001532), (2030.00, 0.001496), (2031.00, 0.00146), (2032.00, 0.00143), (2033.00, 0.001392), (2034.00, 0.001364), (2035.00, 0.001334), (2036.00, 0.001304), (2037.00, 0.001274), (2038.00, 0.001244), (2039.00, 0.001214), (2040.00, 0.00119), (2041.00, 0.00116), (2042.00, 0.001134), (2043.00, 0.001108), (2044.00, 0.001084), (2045.00, 0.001058), (2046.00, 0.001034), (2047.00, 0.001012), (2048.00, 0.00099), (2049.00, 0.000966), (2050.00, 0.000944), (2051.00, 0.000924), (2052.00, 0.000904), (2053.00, 0.00088), (2054.00, 0.00086), (2055.00, 0.000842), (2056.00, 0.000822), (2057.00, 0.000802), (2058.00, 0.000784), (2059.00, 0.000768), (2060.00, 0.00075), (2061.00, 0.000734)

UNITS: persons/person/year

death_rate_H[F, A59] = GRAPH(TIME)

Points: (2011.00, 0.0031), (2012.00, 0.003), (2013.00, 0.0031), (2014.00, 0.0031), (2015.00, 0.0031), (2016.00, 0.0028), (2017.00, 0.002798), (2018.00, 0.002726), (2019.00, 0.002658), (2020.00, 0.00259), (2021.00, 0.002524), (2022.00, 0.002454), (2023.00, 0.002388), (2024.00, 0.002318), (2025.00, 0.002256), (2026.00, 0.002196), (2027.00, 0.002128), (2028.00, 0.002064), (2029.00, 0.002006), (2030.00, 0.001944), (2031.00, 0.001884), (2032.00, 0.00184), (2033.00, 0.0018), (2034.00, 0.001758), (2035.00, 0.00172), (2036.00, 0.00168), (2037.00, 0.00164), (2038.00, 0.001604), (2039.00, 0.001566), (2040.00, 0.001532), (2041.00, 0.001496), (2042.00, 0.001462), (2043.00, 0.00143), (2044.00, 0.001398), (2045.00, 0.001364), (2046.00, 0.001332), (2047.00, 0.001304), (2048.00, 0.001276), (2049.00, 0.001246), (2050.00, 0.001218), (2051.00, 0.001188), (2052.00, 0.001162), (2053.00, 0.001132), (2054.00, 0.00111), (2055.00, 0.001086), (2056.00, 0.00106), (2057.00, 0.001034), (2058.00, 0.001012), (2059.00, 0.00099), (2060.00, 0.000966), (2061.00, 0.000942)

UNITS: persons/person/year

death_rate_H[F, A64] = GRAPH(TIME)

Points: (2011.00, 0.0047), (2012.00, 0.0048), (2013.00, 0.0047), (2014.00, 0.0047), (2015.00, 0.0045), (2016.00, 0.0042), (2017.00, 0.004132), (2018.00, 0.004002), (2019.00, 0.003878), (2020.00, 0.003756), (2021.00, 0.003636), (2022.00, 0.003516), (2023.00, 0.003396), (2024.00, 0.003284), (2025.00, 0.003172), (2026.00, 0.003066), (2027.00, 0.002954), (2028.00, 0.002846), (2029.00, 0.002742), (2030.00, 0.00264), (2031.00, 0.002548), (2032.00, 0.002488), (2033.00, 0.00243), (2034.00, 0.002378), (2035.00, 0.002322), (2036.00, 0.002268), (2037.00, 0.002216), (2038.00, 0.002164), (2039.00, 0.002118), (2040.00, 0.00207), (2041.00, 0.00202), (2042.00, 0.001974), (2043.00, 0.001932), (2044.00, 0.001888), (2045.00, 0.001844), (2046.00, 0.0018), (2047.00, 0.00176), (2048.00, 0.00172), (2049.00, 0.001682), (2050.00, 0.001644), (2051.00, 0.001606), (2052.00, 0.001568), (2053.00, 0.001534), (2054.00, 0.001498), (2055.00, 0.001462), (2056.00, 0.00143), (2057.00, 0.0014), (2058.00, 0.001368), (2059.00, 0.001334), (2060.00, 0.001306), (2061.00, 0.001276)

UNITS: persons/person/year

death_rate_H[F, A69] = GRAPH(TIME)

Points: (2011.00, 0.0074), (2012.00, 0.0075), (2013.00, 0.0072), (2014.00, 0.0073), (2015.00, 0.0071), (2016.00, 0.0066), (2017.00, 0.006584), (2018.00, 0.006374), (2019.00, 0.00617), (2020.00, 0.005972), (2021.00, 0.005786), (2022.00, 0.005586), (2023.00, 0.005394), (2024.00, 0.005208), (2025.00, 0.005032), (2026.00, 0.00486), (2027.00, 0.00468), (2028.00, 0.004506), (2029.00, 0.004338), (2030.00, 0.004176), (2031.00, 0.004022), (2032.00, 0.003928), (2033.00, 0.00384), (2034.00, 0.003752), (2035.00, 0.003668), (2036.00, 0.003582), (2037.00, 0.0035), (2038.00, 0.003424), (2039.00, 0.003344), (2040.00, 0.003268), (2041.00, 0.003194), (2042.00, 0.003122), (2043.00, 0.003048), (2044.00, 0.002984), (2045.00, 0.002914), (2046.00, 0.002848), (2047.00, 0.002782), (2048.00, 0.002718), (2049.00, 0.002656), (2050.00, 0.002596), (2051.00, 0.002536), (2052.00, 0.002482), (2053.00, 0.002424), (2054.00, 0.00237), (2055.00, 0.002314), (2056.00, 0.00226), (2057.00, 0.00221), (2058.00, 0.00216), (2059.00, 0.00211), (2060.00, 0.002064), (2061.00, 0.002016)

UNITS: persons/person/year

death_rate_H[F, A74] = GRAPH(TIME)

Points: (2011.00, 0.0133), (2012.00, 0.0127), (2013.00, 0.012), (2014.00, 0.0125), (2015.00, 0.0121), (2016.00, 0.0111), (2017.00, 0.01136), (2018.00, 0.010996), (2019.00, 0.01065), (2020.00, 0.010308), (2021.00, 0.00998), (2022.00, 0.009642), (2023.00, 0.009312), (2024.00, 0.008994), (2025.00, 0.008686), (2026.00, 0.008388), (2027.00, 0.008078), (2028.00, 0.007778), (2029.00, 0.007488), (2030.00, 0.00721), (2031.00, 0.006942), (2032.00, 0.00678), (2033.00, 0.006628), (2034.00, 0.00648), (2035.00, 0.006332), (2036.00, 0.006188), (2037.00, 0.006046), (2038.00, 0.005908), (2039.00, 0.005776), (2040.00, 0.005642), (2041.00, 0.005514), (2042.00, 0.005392), (2043.00, 0.005268), (2044.00, 0.005148), (2045.00, 0.005032), (2046.00, 0.004918), (2047.00, 0.004806), (2048.00, 0.004694), (2049.00, 0.00459), (2050.00, 0.004486), (2051.00, 0.004384), (2052.00, 0.004284), (2053.00, 0.004186), (2054.00, 0.004092), (2055.00, 0.003998), (2056.00, 0.00391), (2057.00, 0.00382), (2058.00, 0.00373), (2059.00, 0.003646), (2060.00, 0.003564), (2061.00, 0.003484)

UNITS: persons/person/year

death_rate_H[F, A79] = GRAPH(TIME)

Points: (2011.00, 0.0224), (2012.00, 0.0231), (2013.00, 0.0217), (2014.00, 0.0226), (2015.00, 0.0222), (2016.00, 0.0204), (2017.00, 0.020278), (2018.00, 0.019724), (2019.00, 0.019184), (2020.00, 0.018662), (2021.00, 0.018152), (2022.00, 0.017622), (2023.00, 0.017102), (2024.00, 0.016604), (2025.00, 0.016118), (2026.00, 0.015644), (2027.00, 0.015148), (2028.00, 0.014662), (2029.00, 0.014194), (2030.00, 0.01374), (2031.00, 0.0133), (2032.00, 0.013002), (2033.00, 0.012706), (2034.00, 0.012418), (2035.00, 0.012136), (2036.00, 0.01186), (2037.00, 0.011592), (2038.00, 0.01133), (2039.00, 0.011072), (2040.00, 0.010824), (2041.00, 0.01058), (2042.00, 0.010338), (2043.00, 0.010104), (2044.00, 0.009872), (2045.00, 0.00965), (2046.00, 0.009432), (2047.00, 0.009218), (2048.00, 0.00901), (2049.00, 0.008804), (2050.00, 0.008602), (2051.00, 0.00841), (2052.00, 0.008218), (2053.00, 0.008034), (2054.00, 0.007848), (2055.00, 0.007672), (2056.00, 0.007496), (2057.00, 0.007326), (2058.00, 0.007162), (2059.00, 0.006998), (2060.00, 0.00684), (2061.00, 0.006686)

UNITS: persons/person/year

death_rate_H[F, A84] = GRAPH(TIME)

Points: (2011.00, 0.0446), (2012.00, 0.0452), (2013.00, 0.0434), (2014.00, 0.0432), (2015.00, 0.0432), (2016.00, 0.04), (2017.00, 0.040554), (2018.00, 0.039642), (2019.00, 0.038752), (2020.00, 0.037878), (2021.00, 0.037028), (2022.00, 0.036136), (2023.00, 0.03526), (2024.00, 0.03441), (2025.00, 0.033578), (2026.00, 0.032768), (2027.00, 0.031908), (2028.00, 0.031068), (2029.00, 0.03025), (2030.00, 0.029452), (2031.00, 0.028676), (2032.00, 0.028032), (2033.00, 0.0274), (2034.00, 0.02679), (2035.00, 0.026184), (2036.00, 0.025596), (2037.00, 0.02502), (2038.00, 0.024458), (2039.00, 0.023908), (2040.00, 0.02337), (2041.00, 0.022842), (2042.00, 0.022328), (2043.00, 0.021828), (2044.00, 0.021334), (2045.00, 0.020852), (2046.00, 0.020382), (2047.00, 0.019924), (2048.00, 0.019474), (2049.00, 0.019034), (2050.00, 0.018606), (2051.00, 0.018188), (2052.00, 0.017774), (2053.00, 0.017376), (2054.00, 0.016984), (2055.00, 0.0166), (2056.00, 0.016224), (2057.00, 0.015858), (2058.00, 0.0155), (2059.00, 0.015146), (2060.00, 0.014804), (2061.00, 0.014472)

UNITS: persons/person/year

death_rate_H[F, A89] = GRAPH(TIME)

Points: (2011.00, 0.09), (2012.00, 0.0896), (2013.00, 0.0861), (2014.00, 0.0865), (2015.00, 0.0858), (2016.00, 0.0795), (2017.00, 0.082732), (2018.00, 0.081568), (2019.00, 0.08042), (2020.00, 0.079292), (2021.00, 0.078174), (2022.00, 0.076996), (2023.00, 0.075834), (2024.00, 0.074688), (2025.00, 0.073562), (2026.00, 0.072454), (2027.00, 0.071258), (2028.00, 0.070086), (2029.00, 0.068932), (2030.00, 0.067796), (2031.00, 0.066678), (2032.00, 0.06521), (2033.00, 0.063776), (2034.00, 0.06237), (2035.00, 0.060994), (2036.00, 0.059648), (2037.00, 0.05833), (2038.00, 0.05704), (2039.00, 0.055778), (2040.00, 0.054546), (2041.00, 0.053336), (2042.00, 0.052156), (2043.00, 0.050996), (2044.00, 0.049864), (2045.00, 0.048758), (2046.00, 0.047676), (2047.00, 0.046614), (2048.00, 0.04558), (2049.00, 0.044564), (2050.00, 0.043572), (2051.00, 0.042602), (2052.00, 0.041652), (2053.00, 0.040722), (2054.00, 0.039816), (2055.00, 0.038926), (2056.00, 0.038058), (2057.00, 0.037208), (2058.00, 0.036376), (2059.00, 0.035562), (2060.00, 0.034766), (2061.00, 0.033988)

UNITS: persons/person/year

death_rate_H[F, A94] = GRAPH(TIME)

Points: (2011.00, 0.1694), (2012.00, 0.1713), (2013.00, 0.1592), (2014.00, 0.165), (2015.00, 0.1662), (2016.00, 0.1531), (2017.00, 0.154208), (2018.00, 0.153204), (2019.00, 0.152208), (2020.00, 0.15122), (2021.00, 0.150242), (2022.00, 0.149198), (2023.00, 0.148158), (2024.00, 0.14713), (2025.00, 0.14611), (2026.00, 0.145094), (2027.00, 0.143996), (2028.00, 0.142906), (2029.00, 0.141824), (2030.00, 0.14075), (2031.00, 0.139684), (2032.00, 0.136664), (2033.00, 0.133708), (2034.00, 0.130808), (2035.00, 0.127968), (2036.00, 0.125184), (2037.00, 0.122456), (2038.00, 0.119784), (2039.00, 0.117168), (2040.00, 0.114604), (2041.00, 0.112094), (2042.00, 0.109636), (2043.00, 0.107226), (2044.00, 0.10487), (2045.00, 0.102556), (2046.00, 0.100298), (2047.00, 0.098084), (2048.00, 0.095914), (2049.00, 0.093792), (2050.00, 0.091716), (2051.00, 0.089682), (2052.00, 0.087688), (2053.00, 0.08574), (2054.00, 0.08383), (2055.00, 0.081964), (2056.00, 0.08014), (2057.00, 0.078352), (2058.00, 0.076602), (2059.00, 0.07489), (2060.00, 0.073212), (2061.00, 0.071574)

UNITS: persons/person/year

death_rate_H[F, A99] = GRAPH(TIME)

Points: (2011.00, 0.2964), (2012.00, 0.2945), (2013.00, 0.2778), (2014.00, 0.2895), (2015.00, 0.2889), (2016.00, 0.2569), (2017.00, 0.244998), (2018.00, 0.244776), (2019.00, 0.244554), (2020.00, 0.244334), (2021.00, 0.244112), (2022.00, 0.243872), (2023.00, 0.243638), (2024.00, 0.2434), (2025.00, 0.243162), (2026.00, 0.24293), (2027.00, 0.242672), (2028.00, 0.242414), (2029.00, 0.24216), (2030.00, 0.241904), (2031.00, 0.241648), (2032.00, 0.235926), (2033.00, 0.230322), (2034.00, 0.224832), (2035.00, 0.219456), (2036.00, 0.214194), (2037.00, 0.209042), (2038.00, 0.204), (2039.00, 0.19907), (2040.00, 0.19424), (2041.00, 0.18952), (2042.00, 0.184902), (2043.00, 0.180386), (2044.00, 0.175962), (2045.00, 0.171644), (2046.00, 0.16742), (2047.00, 0.163292), (2048.00, 0.159258), (2049.00, 0.15531), (2050.00, 0.151456), (2051.00, 0.147686), (2052.00, 0.144008), (2053.00, 0.140412), (2054.00, 0.136896), (2055.00, 0.133468), (2056.00, 0.130118), (2057.00, 0.126844), (2058.00, 0.123648), (2059.00, 0.120528), (2060.00, 0.11748), (2061.00, 0.114508)

UNITS: persons/person/year

death_rate_H[F, A104] = GRAPH(TIME)

Points: (2011.00, 0.4516), (2012.00, 0.4358), (2013.00, 0.4138), (2014.00, 0.4813), (2015.00, 0.4831), (2016.00, 0.488), (2017.00, 0.643475), (2018.00, 0.643385), (2019.00, 0.643295), (2020.00, 0.64321), (2021.00, 0.64312), (2022.00, 0.643025), (2023.00, 0.64293), (2024.00, 0.642835), (2025.00, 0.642745), (2026.00, 0.64265), (2027.00, 0.642545), (2028.00, 0.642445), (2029.00, 0.64234), (2030.00, 0.64224), (2031.00, 0.642135), (2032.00, 0.638705), (2033.00, 0.635345), (2034.00, 0.63205), (2035.00, 0.62883), (2036.00, 0.62567), (2037.00, 0.62258), (2038.00, 0.619555), (2039.00, 0.616595), (2040.00, 0.6137), (2041.00, 0.610865), (2042.00, 0.608095), (2043.00, 0.605385), (2044.00, 0.602735), (2045.00, 0.600145), (2046.00, 0.59761), (2047.00, 0.595135), (2048.00, 0.59272), (2049.00, 0.590355), (2050.00, 0.588045), (2051.00, 0.58579), (2052.00, 0.583585), (2053.00, 0.581435), (2054.00, 0.579335), (2055.00, 0.57728), (2056.00, 0.57528), (2057.00, 0.573325), (2058.00, 0.571415), (2059.00, 0.569555), (2060.00, 0.567735), (2061.00, 0.565965)

UNITS: persons/person/year

death_rate_M[M, A4] = GRAPH(TIME)

Points: (2011.00, 0.001), (2012.00, 0.0009), (2013.00, 0.0009), (2014.00, 0.0008), (2015.00, 0.0008), (2016.00, 0.0007), (2017.00, 0.001018), (2018.00, 0.000996), (2019.00, 0.000976), (2020.00, 0.000952), (2021.00, 0.000932), (2022.00, 0.000916), (2023.00, 0.0009), (2024.00, 0.000886), (2025.00, 0.000868), (2026.00, 0.000854), (2027.00, 0.000846), (2028.00, 0.000836), (2029.00, 0.000824), (2030.00, 0.000814), (2031.00, 0.000802), (2032.00, 0.000798), (2033.00, 0.000792), (2034.00, 0.000788), (2035.00, 0.000782), (2036.00, 0.000778), (2037.00, 0.00077), (2038.00, 0.000766), (2039.00, 0.000762), (2040.00, 0.000754), (2041.00, 0.00075), (2042.00, 0.000746), (2043.00, 0.000736), (2044.00, 0.000732), (2045.00, 0.000728), (2046.00, 0.000722), (2047.00, 0.000716), (2048.00, 0.000712), (2049.00, 0.000706), (2050.00, 0.0007), (2051.00, 0.000696), (2052.00, 0.000692), (2053.00, 0.000688), (2054.00, 0.000682), (2055.00, 0.000676), (2056.00, 0.000672), (2057.00, 0.000666), (2058.00, 0.000662), (2059.00, 0.000658), (2060.00, 0.000654), (2061.00, 0.00065)

UNITS: persons/person/year

death_rate_M[M, A9] = GRAPH(TIME)

Points: (2011.00, 0.0001), (2012.00, 0.0001), (2013.00, 0.0001), (2014.00, 0.0001), (2015.00, 0.0001), (2016.00, 0.0001), (2017.00, 0.000082), (2018.00, 0.00008), (2019.00, 0.000076), (2020.00, 0.000072), (2021.00, 0.00007), (2022.00, 0.000068), (2023.00, 0.000068), (2024.00, 0.000066), (2025.00, 0.000064), (2026.00, 0.00006), (2027.00, 0.00006), (2028.00, 0.000058), (2029.00, 0.000058), (2030.00, 0.000058), (2031.00, 0.000056), (2032.00, 0.000056), (2033.00, 0.000054), (2034.00, 0.000054), (2035.00, 0.000054), (2036.00, 0.000054), (2037.00, 0.000054), (2038.00, 0.000054), (2039.00, 0.000054), (2040.00, 0.000054), (2041.00, 0.000054), (2042.00, 0.000052), (2043.00, 0.00005), (2044.00, 0.000048), (2045.00, 0.000048), (2046.00, 0.000048), (2047.00, 0.000048), (2048.00, 0.000048), (2049.00, 0.000048), (2050.00, 0.000048), (2051.00, 0.000048), (2052.00, 0.000048), (2053.00, 0.000048), (2054.00, 0.000048), (2055.00, 0.000048), (2056.00, 0.000046), (2057.00, 0.000046), (2058.00, 0.000046), (2059.00, 0.000044), (2060.00, 0.000044), (2061.00, 0.000044)

UNITS: persons/person/year

death_rate_M[M, A14] = GRAPH(TIME)

Points: (2011.00, 0.0001), (2012.00, 0.0001), (2013.00, 0.0001), (2014.00, 0.0001), (2015.00, 0.0001), (2016.00, 0.0001), (2017.00, 0.000088), (2018.00, 0.000088), (2019.00, 0.000084), (2020.00, 0.000078), (2021.00, 0.000078), (2022.00, 0.000076), (2023.00, 0.000074), (2024.00, 0.00007), (2025.00, 0.000066), (2026.00, 0.000066), (2027.00, 0.000066), (2028.00, 0.000064), (2029.00, 0.000062), (2030.00, 0.00006), (2031.00, 0.00006), (2032.00, 0.00006), (2033.00, 0.00006), (2034.00, 0.00006), (2035.00, 0.00006), (2036.00, 0.00006), (2037.00, 0.00006), (2038.00, 0.000058), (2039.00, 0.000056), (2040.00, 0.000054), (2041.00, 0.000054), (2042.00, 0.000054), (2043.00, 0.000054), (2044.00, 0.000054), (2045.00, 0.000054), (2046.00, 0.000054), (2047.00, 0.000054), (2048.00, 0.000054), (2049.00, 0.000054), (2050.00, 0.000052), (2051.00, 0.000052), (2052.00, 0.000052), (2053.00, 0.00005), (2054.00, 0.00005), (2055.00, 0.000048), (2056.00, 0.000048), (2057.00, 0.000048), (2058.00, 0.000048), (2059.00, 0.000048), (2060.00, 0.000048), (2061.00, 0.000048)

UNITS: persons/person/year

death_rate_M[M, A19] = GRAPH(TIME)

Points: (2011.00, 0.0004), (2012.00, 0.0004), (2013.00, 0.0004), (2014.00, 0.0003), (2015.00, 0.0004), (2016.00, 0.0003), (2017.00, 0.000356), (2018.00, 0.000342), (2019.00, 0.000332), (2020.00, 0.000318), (2021.00, 0.000308), (2022.00, 0.000298), (2023.00, 0.00029), (2024.00, 0.00028), (2025.00, 0.000274), (2026.00, 0.000268), (2027.00, 0.00026), (2028.00, 0.000258), (2029.00, 0.00025), (2030.00, 0.000244), (2031.00, 0.000242), (2032.00, 0.000238), (2033.00, 0.000236), (2034.00, 0.000236), (2035.00, 0.000234), (2036.00, 0.000234), (2037.00, 0.00023), (2038.00, 0.000228), (2039.00, 0.000226), (2040.00, 0.000224), (2041.00, 0.000224), (2042.00, 0.00022), (2043.00, 0.00022), (2044.00, 0.00022), (2045.00, 0.00022), (2046.00, 0.000216), (2047.00, 0.000212), (2048.00, 0.000212), (2049.00, 0.000212), (2050.00, 0.00021), (2051.00, 0.000208), (2052.00, 0.000208), (2053.00, 0.000204), (2054.00, 0.000204), (2055.00, 0.000202), (2056.00, 0.0002), (2057.00, 0.0002), (2058.00, 0.000198), (2059.00, 0.000196), (2060.00, 0.000196), (2061.00, 0.000194)

UNITS: persons/person/year

death_rate_M[M, A24] = GRAPH(TIME)

Points: (2011.00, 0.0006), (2012.00, 0.0006), (2013.00, 0.0006), (2014.00, 0.0006), (2015.00, 0.0006), (2016.00, 0.0006), (2017.00, 0.00052), (2018.00, 0.000502), (2019.00, 0.000486), (2020.00, 0.00047), (2021.00, 0.000456), (2022.00, 0.000444), (2023.00, 0.00043), (2024.00, 0.00042), (2025.00, 0.00041), (2026.00, 0.0004), (2027.00, 0.000392), (2028.00, 0.000388), (2029.00, 0.00038), (2030.00, 0.000372), (2031.00, 0.000368), (2032.00, 0.000362), (2033.00, 0.00036), (2034.00, 0.00036), (2035.00, 0.000358), (2036.00, 0.000352), (2037.00, 0.00035), (2038.00, 0.00035), (2039.00, 0.000346), (2040.00, 0.00034), (2041.00, 0.00034), (2042.00, 0.00034), (2043.00, 0.000336), (2044.00, 0.00033), (2045.00, 0.00033), (2046.00, 0.00033), (2047.00, 0.000326), (2048.00, 0.000324), (2049.00, 0.00032), (2050.00, 0.00032), (2051.00, 0.000318), (2052.00, 0.000314), (2053.00, 0.00031), (2054.00, 0.00031), (2055.00, 0.00031), (2056.00, 0.000304), (2057.00, 0.000304), (2058.00, 0.0003), (2059.00, 0.0003), (2060.00, 0.000296), (2061.00, 0.000294)

UNITS: persons/person/year

death_rate_M[M, A29] = GRAPH(TIME)

Points: (2011.00, 0.0007), (2012.00, 0.0007), (2013.00, 0.0007), (2014.00, 0.0006), (2015.00, 0.0007), (2016.00, 0.0006), (2017.00, 0.000628), (2018.00, 0.000608), (2019.00, 0.000592), (2020.00, 0.000578), (2021.00, 0.000562), (2022.00, 0.00055), (2023.00, 0.000538), (2024.00, 0.000528), (2025.00, 0.000518), (2026.00, 0.000506), (2027.00, 0.0005), (2028.00, 0.000492), (2029.00, 0.000482), (2030.00, 0.000478), (2031.00, 0.000472), (2032.00, 0.000468), (2033.00, 0.000462), (2034.00, 0.000462), (2035.00, 0.000458), (2036.00, 0.000452), (2037.00, 0.00045), (2038.00, 0.000446), (2039.00, 0.000444), (2040.00, 0.00044), (2041.00, 0.000438), (2042.00, 0.000434), (2043.00, 0.00043), (2044.00, 0.000428), (2045.00, 0.000424), (2046.00, 0.000422), (2047.00, 0.00042), (2048.00, 0.000418), (2049.00, 0.000412), (2050.00, 0.000412), (2051.00, 0.000408), (2052.00, 0.000402), (2053.00, 0.000402), (2054.00, 0.0004), (2055.00, 0.000396), (2056.00, 0.000392), (2057.00, 0.00039), (2058.00, 0.000388), (2059.00, 0.000384), (2060.00, 0.000382), (2061.00, 0.000378)

UNITS: persons/person/year

death_rate_M[M, A34] = GRAPH(TIME)

Points: (2011.00, 0.0009), (2012.00, 0.0008), (2013.00, 0.0008), (2014.00, 0.0008), (2015.00, 0.0009), (2016.00, 0.0008), (2017.00, 0.00083), (2018.00, 0.000812), (2019.00, 0.000796), (2020.00, 0.00078), (2021.00, 0.000764), (2022.00, 0.000752), (2023.00, 0.00074), (2024.00, 0.00073), (2025.00, 0.000718), (2026.00, 0.000704), (2027.00, 0.000698), (2028.00, 0.00069), (2029.00, 0.000682), (2030.00, 0.000674), (2031.00, 0.000668), (2032.00, 0.00066), (2033.00, 0.00066), (2034.00, 0.00065), (2035.00, 0.00065), (2036.00, 0.000642), (2037.00, 0.00064), (2038.00, 0.000632), (2039.00, 0.00063), (2040.00, 0.000624), (2041.00, 0.000622), (2042.00, 0.000616), (2043.00, 0.000612), (2044.00, 0.00061), (2045.00, 0.000602), (2046.00, 0.0006), (2047.00, 0.000594), (2048.00, 0.00059), (2049.00, 0.000588), (2050.00, 0.00058), (2051.00, 0.000578), (2052.00, 0.000574), (2053.00, 0.000568), (2054.00, 0.000564), (2055.00, 0.000564), (2056.00, 0.000556), (2057.00, 0.000554), (2058.00, 0.000548), (2059.00, 0.000544), (2060.00, 0.000544), (2061.00, 0.000536)

UNITS: persons/person/year

death_rate_M[M, A39] = GRAPH(TIME)

Points: (2011.00, 0.0011), (2012.00, 0.0011), (2013.00, 0.0011), (2014.00, 0.0012), (2015.00, 0.0011), (2016.00, 0.001), (2017.00, 0.001098), (2018.00, 0.001082), (2019.00, 0.001066), (2020.00, 0.00105), (2021.00, 0.001034), (2022.00, 0.001022), (2023.00, 0.001012), (2024.00, 0.000998), (2025.00, 0.000988), (2026.00, 0.000976), (2027.00, 0.000968), (2028.00, 0.000962), (2029.00, 0.000952), (2030.00, 0.000946), (2031.00, 0.000936), (2032.00, 0.000932), (2033.00, 0.000922), (2034.00, 0.000916), (2035.00, 0.000912), (2036.00, 0.000902), (2037.00, 0.0009), (2038.00, 0.000892), (2039.00, 0.000884), (2040.00, 0.000878), (2041.00, 0.000872), (2042.00, 0.000866), (2043.00, 0.000858), (2044.00, 0.000854), (2045.00, 0.000848), (2046.00, 0.000842), (2047.00, 0.000834), (2048.00, 0.00083), (2049.00, 0.000824), (2050.00, 0.000818), (2051.00, 0.000812), (2052.00, 0.000806), (2053.00, 0.0008), (2054.00, 0.000796), (2055.00, 0.000788), (2056.00, 0.000782), (2057.00, 0.000778), (2058.00, 0.000772), (2059.00, 0.000764), (2060.00, 0.000762), (2061.00, 0.000752)

UNITS: persons/person/year

death_rate_M[M, A44] = GRAPH(TIME)

Points: (2011.00, 0.0015), (2012.00, 0.0015), (2013.00, 0.0015), (2014.00, 0.0016), (2015.00, 0.0017), (2016.00, 0.0014), (2017.00, 0.001498), (2018.00, 0.00148), (2019.00, 0.001464), (2020.00, 0.001446), (2021.00, 0.001434), (2022.00, 0.001418), (2023.00, 0.001406), (2024.00, 0.001396), (2025.00, 0.001382), (2026.00, 0.00137), (2027.00, 0.001362), (2028.00, 0.001354), (2029.00, 0.001348), (2030.00, 0.00134), (2031.00, 0.00133), (2032.00, 0.00132), (2033.00, 0.001312), (2034.00, 0.0013), (2035.00, 0.001292), (2036.00, 0.001284), (2037.00, 0.001274), (2038.00, 0.001266), (2039.00, 0.001256), (2040.00, 0.001246), (2041.00, 0.001236), (2042.00, 0.001228), (2043.00, 0.001222), (2044.00, 0.001212), (2045.00, 0.001204), (2046.00, 0.001194), (2047.00, 0.001188), (2048.00, 0.001178), (2049.00, 0.001168), (2050.00, 0.00116), (2051.00, 0.001152), (2052.00, 0.001144), (2053.00, 0.001134), (2054.00, 0.001128), (2055.00, 0.001118), (2056.00, 0.00111), (2057.00, 0.001104), (2058.00, 0.001096), (2059.00, 0.001086), (2060.00, 0.00108), (2061.00, 0.001074)

UNITS: persons/person/year

death_rate_M[M, A49] = GRAPH(TIME)

Points: (2011.00, 0.0023), (2012.00, 0.0022), (2013.00, 0.0023), (2014.00, 0.0022), (2015.00, 0.0023), (2016.00, 0.002), (2017.00, 0.00219), (2018.00, 0.002166), (2019.00, 0.002144), (2020.00, 0.00212), (2021.00, 0.0021), (2022.00, 0.00208), (2023.00, 0.002064), (2024.00, 0.002044), (2025.00, 0.00203), (2026.00, 0.002012), (2027.00, 0.002), (2028.00, 0.00199), (2029.00, 0.00198), (2030.00, 0.001968), (2031.00, 0.001956), (2032.00, 0.001942), (2033.00, 0.001926), (2034.00, 0.001912), (2035.00, 0.0019), (2036.00, 0.001888), (2037.00, 0.00187), (2038.00, 0.00186), (2039.00, 0.001846), (2040.00, 0.001832), (2041.00, 0.00182), (2042.00, 0.001806), (2043.00, 0.001792), (2044.00, 0.001782), (2045.00, 0.001768), (2046.00, 0.001758), (2047.00, 0.001742), (2048.00, 0.00173), (2049.00, 0.001718), (2050.00, 0.001706), (2051.00, 0.00169), (2052.00, 0.00168), (2053.00, 0.001668), (2054.00, 0.001656), (2055.00, 0.001646), (2056.00, 0.001632), (2057.00, 0.001618), (2058.00, 0.001608), (2059.00, 0.001596), (2060.00, 0.001586), (2061.00, 0.001576)

UNITS: persons/person/year

death_rate_M[M, A54] = GRAPH(TIME)

Points: (2011.00, 0.0034), (2012.00, 0.0033), (2013.00, 0.0033), (2014.00, 0.0034), (2015.00, 0.0034), (2016.00, 0.0031), (2017.00, 0.003276), (2018.00, 0.003232), (2019.00, 0.003192), (2020.00, 0.003148), (2021.00, 0.003108), (2022.00, 0.003072), (2023.00, 0.00304), (2024.00, 0.003006), (2025.00, 0.002978), (2026.00, 0.002946), (2027.00, 0.002924), (2028.00, 0.002902), (2029.00, 0.002884), (2030.00, 0.002862), (2031.00, 0.00284), (2032.00, 0.00282), (2033.00, 0.0028), (2034.00, 0.00278), (2035.00, 0.00276), (2036.00, 0.002742), (2037.00, 0.002722), (2038.00, 0.002702), (2039.00, 0.002682), (2040.00, 0.00266), (2041.00, 0.002644), (2042.00, 0.002624), (2043.00, 0.002604), (2044.00, 0.002584), (2045.00, 0.002568), (2046.00, 0.00255), (2047.00, 0.00253), (2048.00, 0.002512), (2049.00, 0.002496), (2050.00, 0.002476), (2051.00, 0.00246), (2052.00, 0.002442), (2053.00, 0.002424), (2054.00, 0.002406), (2055.00, 0.00239), (2056.00, 0.00237), (2057.00, 0.002356), (2058.00, 0.002338), (2059.00, 0.002322), (2060.00, 0.002304), (2061.00, 0.00229)

UNITS: persons/person/year

death_rate_M[M, A59] = GRAPH(TIME)

Points: (2011.00, 0.0053), (2012.00, 0.005), (2013.00, 0.0051), (2014.00, 0.0051), (2015.00, 0.0052), (2016.00, 0.0047), (2017.00, 0.004732), (2018.00, 0.004642), (2019.00, 0.004554), (2020.00, 0.004468), (2021.00, 0.00438), (2022.00, 0.004314), (2023.00, 0.00425), (2024.00, 0.004186), (2025.00, 0.004124), (2026.00, 0.00406), (2027.00, 0.004018), (2028.00, 0.003976), (2029.00, 0.003934), (2030.00, 0.003892), (2031.00, 0.00385), (2032.00, 0.003824), (2033.00, 0.003796), (2034.00, 0.003768), (2035.00, 0.003742), (2036.00, 0.003714), (2037.00, 0.00369), (2038.00, 0.003662), (2039.00, 0.003636), (2040.00, 0.003608), (2041.00, 0.003584), (2042.00, 0.003558), (2043.00, 0.003532), (2044.00, 0.00351), (2045.00, 0.003482), (2046.00, 0.003456), (2047.00, 0.003432), (2048.00, 0.00341), (2049.00, 0.003382), (2050.00, 0.00336), (2051.00, 0.003336), (2052.00, 0.00331), (2053.00, 0.003286), (2054.00, 0.003264), (2055.00, 0.00324), (2056.00, 0.003218), (2057.00, 0.003192), (2058.00, 0.00317), (2059.00, 0.003148), (2060.00, 0.003126), (2061.00, 0.003104)

UNITS: persons/person/year

death_rate_M[M, A64] = GRAPH(TIME)

Points: (2011.00, 0.0081), (2012.00, 0.0077), (2013.00, 0.0077), (2014.00, 0.0078), (2015.00, 0.0078), (2016.00, 0.0071), (2017.00, 0.007072), (2018.00, 0.0069), (2019.00, 0.006728), (2020.00, 0.006562), (2021.00, 0.0064), (2022.00, 0.006274), (2023.00, 0.006152), (2024.00, 0.006034), (2025.00, 0.005916), (2026.00, 0.0058), (2027.00, 0.00572), (2028.00, 0.005642), (2029.00, 0.005566), (2030.00, 0.005488), (2031.00, 0.005414), (2032.00, 0.005376), (2033.00, 0.005336), (2034.00, 0.005298), (2035.00, 0.005262), (2036.00, 0.005222), (2037.00, 0.005184), (2038.00, 0.005146), (2039.00, 0.005112), (2040.00, 0.005076), (2041.00, 0.005038), (2042.00, 0.005002), (2043.00, 0.004966), (2044.00, 0.004932), (2045.00, 0.004892), (2046.00, 0.00486), (2047.00, 0.004824), (2048.00, 0.004792), (2049.00, 0.00476), (2050.00, 0.004722), (2051.00, 0.004688), (2052.00, 0.004656), (2053.00, 0.00462), (2054.00, 0.004588), (2055.00, 0.004554), (2056.00, 0.004522), (2057.00, 0.00449), (2058.00, 0.004458), (2059.00, 0.004424), (2060.00, 0.004396), (2061.00, 0.004362)

UNITS: persons/person/year

death_rate_M[M, A69] = GRAPH(TIME)

Points: (2011.00, 0.0127), (2012.00, 0.0122), (2013.00, 0.0126), (2014.00, 0.0121), (2015.00, 0.0117), (2016.00, 0.0108), (2017.00, 0.01128), (2018.00, 0.010956), (2019.00, 0.010646), (2020.00, 0.010344), (2021.00, 0.010048), (2022.00, 0.00982), (2023.00, 0.0096), (2024.00, 0.009386), (2025.00, 0.009172), (2026.00, 0.008968), (2027.00, 0.008824), (2028.00, 0.008686), (2029.00, 0.008554), (2030.00, 0.008418), (2031.00, 0.008284), (2032.00, 0.008228), (2033.00, 0.00817), (2034.00, 0.008108), (2035.00, 0.00805), (2036.00, 0.007994), (2037.00, 0.007938), (2038.00, 0.007878), (2039.00, 0.007824), (2040.00, 0.007768), (2041.00, 0.007712), (2042.00, 0.007658), (2043.00, 0.007602), (2044.00, 0.007548), (2045.00, 0.007492), (2046.00, 0.007438), (2047.00, 0.007386), (2048.00, 0.007336), (2049.00, 0.00728), (2050.00, 0.007228), (2051.00, 0.007178), (2052.00, 0.007126), (2053.00, 0.007074), (2054.00, 0.007024), (2055.00, 0.006972), (2056.00, 0.006922), (2057.00, 0.006872), (2058.00, 0.006824), (2059.00, 0.006774), (2060.00, 0.006728), (2061.00, 0.00668)

death_rate_M[M, A74] = GRAPH(TIME)

Points: (2011.00, 0.0212), (2012.00, 0.0203), (2013.00, 0.0202), (2014.00, 0.0198), (2015.00, 0.0193), (2016.00, 0.0182), (2017.00, 0.018774), (2018.00, 0.018268), (2019.00, 0.017772), (2020.00, 0.017292), (2021.00, 0.016822), (2022.00, 0.016464), (2023.00, 0.01611), (2024.00, 0.01577), (2025.00, 0.01543), (2026.00, 0.015102), (2027.00, 0.014872), (2028.00, 0.014652), (2029.00, 0.014436), (2030.00, 0.01422), (2031.00, 0.014008), (2032.00, 0.013906), (2033.00, 0.013806), (2034.00, 0.013712), (2035.00, 0.013614), (2036.00, 0.013516), (2037.00, 0.013416), (2038.00, 0.013324), (2039.00, 0.013226), (2040.00, 0.013134), (2041.00, 0.01304), (2042.00, 0.012948), (2043.00, 0.012852), (2044.00, 0.01276), (2045.00, 0.01267), (2046.00, 0.01258), (2047.00, 0.012488), (2048.00, 0.012402), (2049.00, 0.012312), (2050.00, 0.012224), (2051.00, 0.012136), (2052.00, 0.01205), (2053.00, 0.011966), (2054.00, 0.01188), (2055.00, 0.011796), (2056.00, 0.01171), (2057.00, 0.011626), (2058.00, 0.011542), (2059.00, 0.011462), (2060.00, 0.01138), (2061.00, 0.0113)

death_rate_M[M, A79] = GRAPH(TIME)

Points: (2011.00, 0.0378), (2012.00, 0.036), (2013.00, 0.0346), (2014.00, 0.0343), (2015.00, 0.0336), (2016.00, 0.0314), (2017.00, 0.032826), (2018.00, 0.03206), (2019.00, 0.031312), (2020.00, 0.03058), (2021.00, 0.029868), (2022.00, 0.02932), (2023.00, 0.02878), (2024.00, 0.028252), (2025.00, 0.027732), (2026.00, 0.027222), (2027.00, 0.026872), (2028.00, 0.026528), (2029.00, 0.026188), (2030.00, 0.025854), (2031.00, 0.025522), (2032.00, 0.02534), (2033.00, 0.02516), (2034.00, 0.024984), (2035.00, 0.024804), (2036.00, 0.024628), (2037.00, 0.024454), (2038.00, 0.024278), (2039.00, 0.024106), (2040.00, 0.023936), (2041.00, 0.023768), (2042.00, 0.023596), (2043.00, 0.023432), (2044.00, 0.023264), (2045.00, 0.023098), (2046.00, 0.022932), (2047.00, 0.022772), (2048.00, 0.022612), (2049.00, 0.022448), (2050.00, 0.022288), (2051.00, 0.022134), (2052.00, 0.021974), (2053.00, 0.02182), (2054.00, 0.021662), (2055.00, 0.02151), (2056.00, 0.021358), (2057.00, 0.021204), (2058.00, 0.021054), (2059.00, 0.020902), (2060.00, 0.020756), (2061.00, 0.020606)

death_rate_M[M, A84] = GRAPH(TIME)

Points: (2011.00, 0.0677), (2012.00, 0.0662), (2013.00, 0.0633), (2014.00, 0.0632), (2015.00, 0.0618), (2016.00, 0.0587), (2017.00, 0.060962), (2018.00, 0.059892), (2019.00, 0.058844), (2020.00, 0.057814), (2021.00, 0.056804), (2022.00, 0.056018), (2023.00, 0.055244), (2024.00, 0.054484), (2025.00, 0.053732), (2026.00, 0.052988), (2027.00, 0.05248), (2028.00, 0.051974), (2029.00, 0.051474), (2030.00, 0.050982), (2031.00, 0.050488), (2032.00, 0.050138), (2033.00, 0.049784), (2034.00, 0.049436), (2035.00, 0.04909), (2036.00, 0.048746), (2037.00, 0.048404), (2038.00, 0.048066), (2039.00, 0.047728), (2040.00, 0.047392), (2041.00, 0.04706), (2042.00, 0.046734), (2043.00, 0.046404), (2044.00, 0.04608), (2045.00, 0.045756), (2046.00, 0.045434), (2047.00, 0.045116), (2048.00, 0.044798), (2049.00, 0.044484), (2050.00, 0.044168), (2051.00, 0.043858), (2052.00, 0.043552), (2053.00, 0.043244), (2054.00, 0.04294), (2055.00, 0.04264), (2056.00, 0.042338), (2057.00, 0.042042), (2058.00, 0.041746), (2059.00, 0.041452), (2060.00, 0.04116), (2061.00, 0.040872)

death_rate_M[M, A89] = GRAPH(TIME)

Points: (2011.00, 0.1222), (2012.00, 0.1204), (2013.00, 0.1151), (2014.00, 0.1141), (2015.00, 0.1155), (2016.00, 0.1079), (2017.00, 0.109788), (2018.00, 0.108546), (2019.00, 0.10732), (2020.00, 0.106106), (2021.00, 0.104902), (2022.00, 0.103966), (2023.00, 0.10304), (2024.00, 0.102118), (2025.00, 0.101208), (2026.00, 0.100304), (2027.00, 0.099684), (2028.00, 0.099062), (2029.00, 0.098446), (2030.00, 0.097834), (2031.00, 0.097228), (2032.00, 0.096564), (2033.00, 0.095902), (2034.00, 0.095248), (2035.00, 0.094596), (2036.00, 0.093948), (2037.00, 0.093304), (2038.00, 0.092668), (2039.00, 0.092032), (2040.00, 0.0914), (2041.00, 0.090776), (2042.00, 0.090152), (2043.00, 0.089536), (2044.00, 0.088922), (2045.00, 0.08831), (2046.00, 0.087704), (2047.00, 0.087102), (2048.00, 0.086504), (2049.00, 0.08591), (2050.00, 0.08532), (2051.00, 0.084732), (2052.00, 0.08415), (2053.00, 0.083568), (2054.00, 0.082996), (2055.00, 0.082426), (2056.00, 0.081856), (2057.00, 0.081294), (2058.00, 0.080734), (2059.00, 0.080176), (2060.00, 0.079624), (2061.00, 0.079078)

death_rate_M[M, A94] = GRAPH(TIME)

Points: (2011.00, 0.2109), (2012.00, 0.2079), (2013.00, 0.1979), (2014.00, 0.2001), (2015.00, 0.1973), (2016.00, 0.1836), (2017.00, 0.178958), (2018.00, 0.178092), (2019.00, 0.17723), (2020.00, 0.176374), (2021.00, 0.17552), (2022.00, 0.174854), (2023.00, 0.174192), (2024.00, 0.173528), (2025.00, 0.172872), (2026.00, 0.172218), (2027.00, 0.171762), (2028.00, 0.17131), (2029.00, 0.17086), (2030.00, 0.170408), (2031.00, 0.16996), (2032.00, 0.168846), (2033.00, 0.167736), (2034.00, 0.16663), (2035.00, 0.165534), (2036.00, 0.164446), (2037.00, 0.163362), (2038.00, 0.162288), (2039.00, 0.161216), (2040.00, 0.160152), (2041.00, 0.159094), (2042.00, 0.158042), (2043.00, 0.157), (2044.00, 0.15596), (2045.00, 0.15493), (2046.00, 0.153902), (2047.00, 0.152882), (2048.00, 0.151868), (2049.00, 0.15086), (2050.00, 0.149862), (2051.00, 0.148864), (2052.00, 0.147878), (2053.00, 0.146892), (2054.00, 0.145916), (2055.00, 0.144944), (2056.00, 0.14398), (2057.00, 0.14302), (2058.00, 0.142068), (2059.00, 0.14112), (2060.00, 0.140178), (2061.00, 0.139242)

death_rate_M[M, A99] = GRAPH(TIME)

Points: (2011.00, 0.3405), (2012.00, 0.3461), (2013.00, 0.3114), (2014.00, 0.3098), (2015.00, 0.3121), (2016.00, 0.2776), (2017.00, 0.25717), (2018.00, 0.257162), (2019.00, 0.257154), (2020.00, 0.257146), (2021.00, 0.25714), (2022.00, 0.257134), (2023.00, 0.257126), (2024.00, 0.257122), (2025.00, 0.257116), (2026.00, 0.25711), (2027.00, 0.257108), (2028.00, 0.257102), (2029.00, 0.257098), (2030.00, 0.257094), (2031.00, 0.25709), (2032.00, 0.25548), (2033.00, 0.253878), (2034.00, 0.252282), (2035.00, 0.250698), (2036.00, 0.249122), (2037.00, 0.247552), (2038.00, 0.245994), (2039.00, 0.244444), (2040.00, 0.242898), (2041.00, 0.241364), (2042.00, 0.239838), (2043.00, 0.23832), (2044.00, 0.236814), (2045.00, 0.235312), (2046.00, 0.233816), (2047.00, 0.232334), (2048.00, 0.230858), (2049.00, 0.229388), (2050.00, 0.22793), (2051.00, 0.226474), (2052.00, 0.22503), (2053.00, 0.223596), (2054.00, 0.222168), (2055.00, 0.220746), (2056.00, 0.219336), (2057.00, 0.217934), (2058.00, 0.216536), (2059.00, 0.215148), (2060.00, 0.213768), (2061.00, 0.212394)

death_rate_M[M, A104] = GRAPH(TIME)

Points: (2011.00, 0.4131), (2012.00, 0.4072), (2013.00, 0.3934), (2014.00, 0.4316), (2015.00, 0.4668), (2016.00, 0.5088), (2017.00, 0.646455), (2018.00, 0.646455), (2019.00, 0.646455), (2020.00, 0.646455), (2021.00, 0.64645), (2022.00, 0.64645), (2023.00, 0.64645), (2024.00, 0.64645), (2025.00, 0.646445), (2026.00, 0.646445), (2027.00, 0.646445), (2028.00, 0.646445), (2029.00, 0.646445), (2030.00, 0.64644), (2031.00, 0.64644), (2032.00, 0.64554), (2033.00, 0.644645), (2034.00, 0.643755), (2035.00, 0.64287), (2036.00, 0.64199), (2037.00, 0.641115), (2038.00, 0.64024), (2039.00, 0.639375), (2040.00, 0.63851), (2041.00, 0.63765), (2042.00, 0.6368), (2043.00, 0.63595), (2044.00, 0.635105), (2045.00, 0.63426), (2046.00, 0.633425), (2047.00, 0.632595), (2048.00, 0.631765), (2049.00, 0.630945), (2050.00, 0.630125), (2051.00, 0.62931), (2052.00, 0.6285), (2053.00, 0.627695), (2054.00, 0.62689), (2055.00, 0.626095), (2056.00, 0.6253), (2057.00, 0.624515), (2058.00, 0.62373), (2059.00, 0.62295), (2060.00, 0.622175), (2061.00, 0.6214)

death_rate_M[F, A4] = GRAPH(TIME)

Points: (2011.00, 0.0008), (2012.00, 0.0008), (2013.00, 0.0008), (2014.00, 0.0008), (2015.00, 0.0007), (2016.00, 0.0006), (2017.00, 0.000738), (2018.00, 0.000722), (2019.00, 0.000704), (2020.00, 0.00069), (2021.00, 0.000674), (2022.00, 0.000664), (2023.00, 0.00065), (2024.00, 0.00064), (2025.00, 0.00063), (2026.00, 0.00062), (2027.00, 0.000612), (2028.00, 0.000602), (2029.00, 0.000594), (2030.00, 0.000588), (2031.00, 0.00058), (2032.00, 0.000578), (2033.00, 0.000574), (2034.00, 0.000572), (2035.00, 0.000566), (2036.00, 0.000564), (2037.00, 0.000562), (2038.00, 0.000556), (2039.00, 0.000552), (2040.00, 0.00055), (2041.00, 0.000546), (2042.00, 0.000544), (2043.00, 0.000542), (2044.00, 0.000538), (2045.00, 0.000536), (2046.00, 0.000534), (2047.00, 0.000528), (2048.00, 0.000526), (2049.00, 0.000524), (2050.00, 0.00052), (2051.00, 0.000518), (2052.00, 0.000516), (2053.00, 0.000512), (2054.00, 0.000508), (2055.00, 0.000506), (2056.00, 0.000504), (2057.00, 0.0005), (2058.00, 0.000496), (2059.00, 0.000492), (2060.00, 0.000488), (2061.00, 0.000486)

death_rate_M[F, A9] = GRAPH(TIME)

Points: (2011.00, 0.0001), (2012.00, 0.0001), (2013.00, 0.0001), (2014.00, 0.0001), (2015.00, 0.0001), (2016.00, 0.0001), (2017.00, 0.000062), (2018.00, 0.000056), (2019.00, 0.000056), (2020.00, 0.000054), (2021.00, 0.000054), (2022.00, 0.000052), (2023.00, 0.000048), (2024.00, 0.000046), (2025.00, 0.000046), (2026.00, 0.000044), (2027.00, 0.000044), (2028.00, 0.000044), (2029.00, 0.000042), (2030.00, 0.000042), (2031.00, 0.000042), (2032.00, 0.000042), (2033.00, 0.000042), (2034.00, 0.000042), (2035.00, 0.000042), (2036.00, 0.000042), (2037.00, 0.000042), (2038.00, 0.000042), (2039.00, 0.000042), (2040.00, 0.00004), (2041.00, 0.000036), (2042.00, 0.000036), (2043.00, 0.000036), (2044.00, 0.000036), (2045.00, 0.000036), (2046.00, 0.000036), (2047.00, 0.000036), (2048.00, 0.000034), (2049.00, 0.000034), (2050.00, 0.000034), (2051.00, 0.000034), (2052.00, 0.000034), (2053.00, 0.000034), (2054.00, 0.000034), (2055.00, 0.000034), (2056.00, 0.000034), (2057.00, 0.000034), (2058.00, 0.000034), (2059.00, 0.000034), (2060.00, 0.000034), (2061.00, 0.000034)

death_rate_M[F, A14] = GRAPH(TIME)

Points: (2011.00, 0.0001), (2012.00, 0.0001), (2013.00, 0.0001), (2014.00, 0.0001), (2015.00, 0.0001), (2016.00, 0.0001), (2017.00, 0.00007), (2018.00, 0.000064), (2019.00, 0.000064), (2020.00, 0.000062), (2021.00, 0.00006), (2022.00, 0.000058), (2023.00, 0.000058), (2024.00, 0.000054), (2025.00, 0.000052), (2026.00, 0.00005), (2027.00, 0.00005), (2028.00, 0.00005), (2029.00, 0.00005), (2030.00, 0.000048), (2031.00, 0.000048), (2032.00, 0.000048), (2033.00, 0.000048), (2034.00, 0.000046), (2035.00, 0.000046), (2036.00, 0.000046), (2037.00, 0.000046), (2038.00, 0.000046), (2039.00, 0.000046), (2040.00, 0.000046), (2041.00, 0.000046), (2042.00, 0.000046), (2043.00, 0.000044), (2044.00, 0.000044), (2045.00, 0.000042), (2046.00, 0.000042), (2047.00, 0.00004), (2048.00, 0.00004), (2049.00, 0.00004), (2050.00, 0.00004), (2051.00, 0.00004), (2052.00, 0.00004), (2053.00, 0.00004), (2054.00, 0.00004), (2055.00, 0.00004), (2056.00, 0.00004), (2057.00, 0.00004), (2058.00, 0.00004), (2059.00, 0.00004), (2060.00, 0.00004), (2061.00, 0.00004)

death_rate_M[F, A19] = GRAPH(TIME)

Points: (2011.00, 0.0002), (2012.00, 0.0002), (2013.00, 0.0002), (2014.00, 0.0002), (2015.00, 0.0002), (2016.00, 0.0002), (2017.00, 0.000172), (2018.00, 0.000166), (2019.00, 0.00016), (2020.00, 0.000156), (2021.00, 0.000148), (2022.00, 0.000146), (2023.00, 0.000144), (2024.00, 0.000138), (2025.00, 0.000136), (2026.00, 0.000132), (2027.00, 0.000128), (2028.00, 0.000128), (2029.00, 0.000126), (2030.00, 0.000122), (2031.00, 0.00012), (2032.00, 0.000118), (2033.00, 0.000118), (2034.00, 0.000118), (2035.00, 0.000118), (2036.00, 0.000118), (2037.00, 0.000114), (2038.00, 0.000114), (2039.00, 0.000114), (2040.00, 0.000114), (2041.00, 0.000114), (2042.00, 0.000114), (2043.00, 0.000114), (2044.00, 0.000114), (2045.00, 0.000112), (2046.00, 0.000112), (2047.00, 0.000112), (2048.00, 0.00011), (2049.00, 0.00011), (2050.00, 0.000104), (2051.00, 0.000104), (2052.00, 0.000104), (2053.00, 0.000104), (2054.00, 0.000104), (2055.00, 0.000104), (2056.00, 0.000104), (2057.00, 0.000104), (2058.00, 0.000104), (2059.00, 0.000104), (2060.00, 0.000104), (2061.00, 0.000102)

death_rate_M[F, A24] = GRAPH(TIME)

Points: (2011.00, 0.0003), (2012.00, 0.0003), (2013.00, 0.0002), (2014.00, 0.0002), (2015.00, 0.0002), (2016.00, 0.0002), (2017.00, 0.000222), (2018.00, 0.000214), (2019.00, 0.000212), (2020.00, 0.000204), (2021.00, 0.000202), (2022.00, 0.000194), (2023.00, 0.000192), (2024.00, 0.00019), (2025.00, 0.000184), (2026.00, 0.000182), (2027.00, 0.000182), (2028.00, 0.000174), (2029.00, 0.000174), (2030.00, 0.000172), (2031.00, 0.000172), (2032.00, 0.000172), (2033.00, 0.000166), (2034.00, 0.000164), (2035.00, 0.000164), (2036.00, 0.000164), (2037.00, 0.000164), (2038.00, 0.000164), (2039.00, 0.000162), (2040.00, 0.000162), (2041.00, 0.000162), (2042.00, 0.000162), (2043.00, 0.00016), (2044.00, 0.000154), (2045.00, 0.000154), (2046.00, 0.000154), (2047.00, 0.000154), (2048.00, 0.000154), (2049.00, 0.000154), (2050.00, 0.000152), (2051.00, 0.000152), (2052.00, 0.000152), (2053.00, 0.00015), (2054.00, 0.000148), (2055.00, 0.000146), (2056.00, 0.000144), (2057.00, 0.000144), (2058.00, 0.000144), (2059.00, 0.000144), (2060.00, 0.000144), (2061.00, 0.000144)

death_rate_M[F, A29] = GRAPH(TIME)

Points: (2011.00, 0.0003), (2012.00, 0.0003), (2013.00, 0.0003), (2014.00, 0.0003), (2015.00, 0.0003), (2016.00, 0.0003), (2017.00, 0.000276), (2018.00, 0.000268), (2019.00, 0.000266), (2020.00, 0.000258), (2021.00, 0.000256), (2022.00, 0.000248), (2023.00, 0.000248), (2024.00, 0.000244), (2025.00, 0.000238), (2026.00, 0.000238), (2027.00, 0.000234), (2028.00, 0.000228), (2029.00, 0.000228), (2030.00, 0.000228), (2031.00, 0.000224), (2032.00, 0.000222), (2033.00, 0.00022), (2034.00, 0.000218), (2035.00, 0.000218), (2036.00, 0.000218), (2037.00, 0.000218), (2038.00, 0.000216), (2039.00, 0.000212), (2040.00, 0.000212), (2041.00, 0.000212), (2042.00, 0.000208), (2043.00, 0.000208), (2044.00, 0.000208), (2045.00, 0.000206), (2046.00, 0.000206), (2047.00, 0.000204), (2048.00, 0.000204), (2049.00, 0.000202), (2050.00, 0.0002), (2051.00, 0.000198), (2052.00, 0.000196), (2053.00, 0.000196), (2054.00, 0.000196), (2055.00, 0.000194), (2056.00, 0.000194), (2057.00, 0.000194), (2058.00, 0.000192), (2059.00, 0.000192), (2060.00, 0.000188), (2061.00, 0.000186)

death_rate_M[F, A34] = GRAPH(TIME)

Points: (2011.00, 0.0004), (2012.00, 0.0004), (2013.00, 0.0004), (2014.00, 0.0004), (2015.00, 0.0004), (2016.00, 0.0004), (2017.00, 0.000374), (2018.00, 0.000368), (2019.00, 0.000358), (2020.00, 0.000358), (2021.00, 0.000348), (2022.00, 0.000348), (2023.00, 0.000346), (2024.00, 0.000338), (2025.00, 0.000338), (2026.00, 0.000334), (2027.00, 0.000328), (2028.00, 0.000328), (2029.00, 0.000326), (2030.00, 0.00032), (2031.00, 0.000318), (2032.00, 0.000316), (2033.00, 0.000316), (2034.00, 0.000314), (2035.00, 0.000312), (2036.00, 0.00031), (2037.00, 0.000306), (2038.00, 0.000306), (2039.00, 0.000304), (2040.00, 0.000304), (2041.00, 0.000302), (2042.00, 0.000298), (2043.00, 0.000296), (2044.00, 0.000296), (2045.00, 0.000294), (2046.00, 0.000294), (2047.00, 0.00029), (2048.00, 0.000288), (2049.00, 0.000288), (2050.00, 0.000284), (2051.00, 0.000284), (2052.00, 0.000282), (2053.00, 0.00028), (2054.00, 0.00028), (2055.00, 0.000278), (2056.00, 0.000276), (2057.00, 0.000272), (2058.00, 0.000272), (2059.00, 0.000272), (2060.00, 0.00027), (2061.00, 0.00027)

death_rate_M[F, A39] = GRAPH(TIME)

Points: (2011.00, 0.0006), (2012.00, 0.0006), (2013.00, 0.0006), (2014.00, 0.0006), (2015.00, 0.0006), (2016.00, 0.0005), (2017.00, 0.000568), (2018.00, 0.000562), (2019.00, 0.000556), (2020.00, 0.00055), (2021.00, 0.000544), (2022.00, 0.00054), (2023.00, 0.000534), (2024.00, 0.000532), (2025.00, 0.000524), (2026.00, 0.000522), (2027.00, 0.000518), (2028.00, 0.000514), (2029.00, 0.000512), (2030.00, 0.00051), (2031.00, 0.000504), (2032.00, 0.000502), (2033.00, 0.0005), (2034.00, 0.000496), (2035.00, 0.000492), (2036.00, 0.00049), (2037.00, 0.000488), (2038.00, 0.000484), (2039.00, 0.000484), (2040.00, 0.00048), (2041.00, 0.000476), (2042.00, 0.000474), (2043.00, 0.000474), (2044.00, 0.000466), (2045.00, 0.000466), (2046.00, 0.000464), (2047.00, 0.000458), (2048.00, 0.000458), (2049.00, 0.000456), (2050.00, 0.000454), (2051.00, 0.000448), (2052.00, 0.000448), (2053.00, 0.000446), (2054.00, 0.000442), (2055.00, 0.000438), (2056.00, 0.000438), (2057.00, 0.000436), (2058.00, 0.000432), (2059.00, 0.000428), (2060.00, 0.000426), (2061.00, 0.000426)

death_rate_M[F, A44] = GRAPH(TIME)

Points: (2011.00, 0.0009), (2012.00, 0.0009), (2013.00, 0.0009), (2014.00, 0.0009), (2015.00, 0.0009), (2016.00, 0.0009), (2017.00, 0.0009), (2018.00, 0.000892), (2019.00, 0.000884), (2020.00, 0.000874), (2021.00, 0.000866), (2022.00, 0.000864), (2023.00, 0.000856), (2024.00, 0.000848), (2025.00, 0.000844), (2026.00, 0.000836), (2027.00, 0.000834), (2028.00, 0.000828), (2029.00, 0.000824), (2030.00, 0.00082), (2031.00, 0.000814), (2032.00, 0.00081), (2033.00, 0.000804), (2034.00, 0.0008), (2035.00, 0.000798), (2036.00, 0.00079), (2037.00, 0.000788), (2038.00, 0.00078), (2039.00, 0.000778), (2040.00, 0.000774), (2041.00, 0.000768), (2042.00, 0.000764), (2043.00, 0.00076), (2044.00, 0.000754), (2045.00, 0.000752), (2046.00, 0.000746), (2047.00, 0.000742), (2048.00, 0.000738), (2049.00, 0.000732), (2050.00, 0.00073), (2051.00, 0.000724), (2052.00, 0.000722), (2053.00, 0.000716), (2054.00, 0.000714), (2055.00, 0.000708), (2056.00, 0.000704), (2057.00, 0.0007), (2058.00, 0.000696), (2059.00, 0.00069), (2060.00, 0.00069), (2061.00, 0.000684)

death_rate_M[F, A49] = GRAPH(TIME)

Points: (2011.00, 0.0014), (2012.00, 0.0013), (2013.00, 0.0014), (2014.00, 0.0014), (2015.00, 0.0014), (2016.00, 0.0013), (2017.00, 0.00138), (2018.00, 0.001364), (2019.00, 0.001348), (2020.00, 0.001334), (2021.00, 0.001318), (2022.00, 0.001304), (2023.00, 0.001294), (2024.00, 0.001282), (2025.00, 0.001276), (2026.00, 0.001264), (2027.00, 0.001254), (2028.00, 0.001246), (2029.00, 0.001238), (2030.00, 0.00123), (2031.00, 0.001222), (2032.00, 0.001212), (2033.00, 0.001208), (2034.00, 0.001202), (2035.00, 0.001194), (2036.00, 0.001186), (2037.00, 0.001178), (2038.00, 0.001176), (2039.00, 0.001166), (2040.00, 0.001158), (2041.00, 0.001154), (2042.00, 0.001146), (2043.00, 0.001142), (2044.00, 0.001132), (2045.00, 0.001126), (2046.00, 0.001122), (2047.00, 0.001114), (2048.00, 0.001108), (2049.00, 0.0011), (2050.00, 0.001094), (2051.00, 0.00109), (2052.00, 0.001082), (2053.00, 0.001074), (2054.00, 0.001072), (2055.00, 0.001062), (2056.00, 0.001056), (2057.00, 0.001052), (2058.00, 0.001046), (2059.00, 0.001038), (2060.00, 0.001036), (2061.00, 0.001026)

death_rate_M[F, A54] = GRAPH(TIME)

Points: (2011.00, 0.0022), (2012.00, 0.002), (2013.00, 0.0021), (2014.00, 0.002), (2015.00, 0.0021), (2016.00, 0.0019), (2017.00, 0.001996), (2018.00, 0.001966), (2019.00, 0.001934), (2020.00, 0.001906), (2021.00, 0.001876), (2022.00, 0.001852), (2023.00, 0.001828), (2024.00, 0.001806), (2025.00, 0.001786), (2026.00, 0.001764), (2027.00, 0.001746), (2028.00, 0.001732), (2029.00, 0.001716), (2030.00, 0.001702), (2031.00, 0.001684), (2032.00, 0.001676), (2033.00, 0.001666), (2034.00, 0.001656), (2035.00, 0.001646), (2036.00, 0.001636), (2037.00, 0.001628), (2038.00, 0.001616), (2039.00, 0.001606), (2040.00, 0.001596), (2041.00, 0.00159), (2042.00, 0.00158), (2043.00, 0.001572), (2044.00, 0.001562), (2045.00, 0.001552), (2046.00, 0.001542), (2047.00, 0.001532), (2048.00, 0.001528), (2049.00, 0.001518), (2050.00, 0.00151), (2051.00, 0.0015), (2052.00, 0.001492), (2053.00, 0.001484), (2054.00, 0.001474), (2055.00, 0.001464), (2056.00, 0.001458), (2057.00, 0.00145), (2058.00, 0.001442), (2059.00, 0.001434), (2060.00, 0.001426), (2061.00, 0.001416)

death_rate_M[F, A59] = GRAPH(TIME)

Points: (2011.00, 0.0031), (2012.00, 0.003), (2013.00, 0.0031), (2014.00, 0.0031), (2015.00, 0.0031), (2016.00, 0.0028), (2017.00, 0.002814), (2018.00, 0.002758), (2019.00, 0.002704), (2020.00, 0.00265), (2021.00, 0.002596), (2022.00, 0.002556), (2023.00, 0.002516), (2024.00, 0.002478), (2025.00, 0.002436), (2026.00, 0.002398), (2027.00, 0.002372), (2028.00, 0.002344), (2029.00, 0.002318), (2030.00, 0.00229), (2031.00, 0.00226), (2032.00, 0.00225), (2033.00, 0.002234), (2034.00, 0.002224), (2035.00, 0.00221), (2036.00, 0.002198), (2037.00, 0.002188), (2038.00, 0.00217), (2039.00, 0.002158), (2040.00, 0.002146), (2041.00, 0.002136), (2042.00, 0.002124), (2043.00, 0.00211), (2044.00, 0.0021), (2045.00, 0.002084), (2046.00, 0.002074), (2047.00, 0.002064), (2048.00, 0.00205), (2049.00, 0.002038), (2050.00, 0.002028), (2051.00, 0.002014), (2052.00, 0.002004), (2053.00, 0.001992), (2054.00, 0.001978), (2055.00, 0.001966), (2056.00, 0.001956), (2057.00, 0.001946), (2058.00, 0.001932), (2059.00, 0.001922), (2060.00, 0.001912), (2061.00, 0.001902)

death_rate_M[F, A64] = GRAPH(TIME)

Points: (2011.00, 0.0047), (2012.00, 0.0048), (2013.00, 0.0047), (2014.00, 0.0047), (2015.00, 0.0045), (2016.00, 0.0042), (2017.00, 0.00416), (2018.00, 0.00406), (2019.00, 0.003958), (2020.00, 0.00386), (2021.00, 0.003768), (2022.00, 0.003694), (2023.00, 0.003624), (2024.00, 0.003558), (2025.00, 0.00349), (2026.00, 0.003422), (2027.00, 0.003372), (2028.00, 0.003324), (2029.00, 0.003278), (2030.00, 0.00323), (2031.00, 0.00318), (2032.00, 0.003164), (2033.00, 0.003146), (2034.00, 0.003126), (2035.00, 0.003112), (2036.00, 0.003092), (2037.00, 0.003076), (2038.00, 0.003056), (2039.00, 0.00304), (2040.00, 0.00302), (2041.00, 0.003002), (2042.00, 0.002984), (2043.00, 0.00297), (2044.00, 0.00295), (2045.00, 0.002934), (2046.00, 0.002918), (2047.00, 0.0029), (2048.00, 0.002884), (2049.00, 0.002868), (2050.00, 0.002852), (2051.00, 0.002836), (2052.00, 0.002818), (2053.00, 0.002804), (2054.00, 0.002786), (2055.00, 0.00277), (2056.00, 0.002756), (2057.00, 0.002738), (2058.00, 0.00272), (2059.00, 0.002708), (2060.00, 0.002692), (2061.00, 0.002676)

death_rate_M[F, A69] = GRAPH(TIME)

Points: (2011.00, 0.0074), (2012.00, 0.0075), (2013.00, 0.0072), (2014.00, 0.0073), (2015.00, 0.0071), (2016.00, 0.0066), (2017.00, 0.006632), (2018.00, 0.006464), (2019.00, 0.006302), (2020.00, 0.006144), (2021.00, 0.00599), (2022.00, 0.005876), (2023.00, 0.005762), (2024.00, 0.005652), (2025.00, 0.005542), (2026.00, 0.005436), (2027.00, 0.005354), (2028.00, 0.005276), (2029.00, 0.005198), (2030.00, 0.005124), (2031.00, 0.00505), (2032.00, 0.005016), (2033.00, 0.00499), (2034.00, 0.00496), (2035.00, 0.004932), (2036.00, 0.004904), (2037.00, 0.004876), (2038.00, 0.004848), (2039.00, 0.004818), (2040.00, 0.004794), (2041.00, 0.004766), (2042.00, 0.004736), (2043.00, 0.00471), (2044.00, 0.004684), (2045.00, 0.004656), (2046.00, 0.004628), (2047.00, 0.004602), (2048.00, 0.004574), (2049.00, 0.00455), (2050.00, 0.004524), (2051.00, 0.0045), (2052.00, 0.004472), (2053.00, 0.004444), (2054.00, 0.004418), (2055.00, 0.004392), (2056.00, 0.00437), (2057.00, 0.004342), (2058.00, 0.004318), (2059.00, 0.004294), (2060.00, 0.00427), (2061.00, 0.004244)

death_rate_M[F, A74] = GRAPH(TIME)

Points: (2011.00, 0.0133), (2012.00, 0.0127), (2013.00, 0.012), (2014.00, 0.0125), (2015.00, 0.0121), (2016.00, 0.0111), (2017.00, 0.01144), (2018.00, 0.011156), (2019.00, 0.010876), (2020.00, 0.010604), (2021.00, 0.01034), (2022.00, 0.010142), (2023.00, 0.009944), (2024.00, 0.009754), (2025.00, 0.009566), (2026.00, 0.00938), (2027.00, 0.009244), (2028.00, 0.009108), (2029.00, 0.008974), (2030.00, 0.008842), (2031.00, 0.008712), (2032.00, 0.008662), (2033.00, 0.008612), (2034.00, 0.008564), (2035.00, 0.008514), (2036.00, 0.008466), (2037.00, 0.008414), (2038.00, 0.008366), (2039.00, 0.008322), (2040.00, 0.008272), (2041.00, 0.008222), (2042.00, 0.008178), (2043.00, 0.008132), (2044.00, 0.008084), (2045.00, 0.008038), (2046.00, 0.007992), (2047.00, 0.007946), (2048.00, 0.0079), (2049.00, 0.007854), (2050.00, 0.007806), (2051.00, 0.007764), (2052.00, 0.00772), (2053.00, 0.007678), (2054.00, 0.00763), (2055.00, 0.007588), (2056.00, 0.007544), (2057.00, 0.007498), (2058.00, 0.007458), (2059.00, 0.007412), (2060.00, 0.007372), (2061.00, 0.00733)

death_rate_M[F, A79] = GRAPH(TIME)

Points: (2011.00, 0.0224), (2012.00, 0.0231), (2013.00, 0.0217), (2014.00, 0.0226), (2015.00, 0.0222), (2016.00, 0.0204), (2017.00, 0.020402), (2018.00, 0.019964), (2019.00, 0.019538), (2020.00, 0.01912), (2021.00, 0.01871), (2022.00, 0.018402), (2023.00, 0.018098), (2024.00, 0.017798), (2025.00, 0.017504), (2026.00, 0.017214), (2027.00, 0.017), (2028.00, 0.016786), (2029.00, 0.016574), (2030.00, 0.016366), (2031.00, 0.016158), (2032.00, 0.016066), (2033.00, 0.015974), (2034.00, 0.01588), (2035.00, 0.015792), (2036.00, 0.015704), (2037.00, 0.015612), (2038.00, 0.015524), (2039.00, 0.015432), (2040.00, 0.015346), (2041.00, 0.015258), (2042.00, 0.015168), (2043.00, 0.015084), (2044.00, 0.014998), (2045.00, 0.01491), (2046.00, 0.014826), (2047.00, 0.014742), (2048.00, 0.014656), (2049.00, 0.014572), (2050.00, 0.01449), (2051.00, 0.014408), (2052.00, 0.014324), (2053.00, 0.014242), (2054.00, 0.01416), (2055.00, 0.01408), (2056.00, 0.013998), (2057.00, 0.013918), (2058.00, 0.013838), (2059.00, 0.013758), (2060.00, 0.013682), (2061.00, 0.013602)

death_rate_M[F, A84] = GRAPH(TIME)

Points: (2011.00, 0.0446), (2012.00, 0.0452), (2013.00, 0.0434), (2014.00, 0.0432), (2015.00, 0.0432), (2016.00, 0.04), (2017.00, 0.040756), (2018.00, 0.040038), (2019.00, 0.039332), (2020.00, 0.038642), (2021.00, 0.03796), (2022.00, 0.037444), (2023.00, 0.036934), (2024.00, 0.036434), (2025.00, 0.035938), (2026.00, 0.035448), (2027.00, 0.035082), (2028.00, 0.03472), (2029.00, 0.03436), (2030.00, 0.034006), (2031.00, 0.033652), (2032.00, 0.03346), (2033.00, 0.033272), (2034.00, 0.03308), (2035.00, 0.032896), (2036.00, 0.032708), (2037.00, 0.032526), (2038.00, 0.03234), (2039.00, 0.032156), (2040.00, 0.031972), (2041.00, 0.03179), (2042.00, 0.03161), (2043.00, 0.03143), (2044.00, 0.031254), (2045.00, 0.031078), (2046.00, 0.0309), (2047.00, 0.030724), (2048.00, 0.03055), (2049.00, 0.030374), (2050.00, 0.030204), (2051.00, 0.030032), (2052.00, 0.029862), (2053.00, 0.02969), (2054.00, 0.029522), (2055.00, 0.029354), (2056.00, 0.029188), (2057.00, 0.029022), (2058.00, 0.02886), (2059.00, 0.028696), (2060.00, 0.02853), (2061.00, 0.028368)

death_rate_M[F, A89] = GRAPH(TIME)

Points: (2011.00, 0.09), (2012.00, 0.0896), (2013.00, 0.0861), (2014.00, 0.0865), (2015.00, 0.0858), (2016.00, 0.0795), (2017.00, 0.08299), (2018.00, 0.082076), (2019.00, 0.08117), (2020.00, 0.08028), (2021.00, 0.079396), (2022.00, 0.07872), (2023.00, 0.078054), (2024.00, 0.077392), (2025.00, 0.076734), (2026.00, 0.076084), (2027.00, 0.075594), (2028.00, 0.075106), (2029.00, 0.074622), (2030.00, 0.074138), (2031.00, 0.073658), (2032.00, 0.07325), (2033.00, 0.07284), (2034.00, 0.072442), (2035.00, 0.07204), (2036.00, 0.071638), (2037.00, 0.071238), (2038.00, 0.070842), (2039.00, 0.070452), (2040.00, 0.070056), (2041.00, 0.069668), (2042.00, 0.06928), (2043.00, 0.068898), (2044.00, 0.068514), (2045.00, 0.06813), (2046.00, 0.067752), (2047.00, 0.067376), (2048.00, 0.067002), (2049.00, 0.066626), (2050.00, 0.066258), (2051.00, 0.065886), (2052.00, 0.06552), (2053.00, 0.065154), (2054.00, 0.06479), (2055.00, 0.064432), (2056.00, 0.06407), (2057.00, 0.063714), (2058.00, 0.063358), (2059.00, 0.063004), (2060.00, 0.062656), (2061.00, 0.062302)

death_rate_M[F, A94] = GRAPH(TIME)

Points: (2011.00, 0.1694), (2012.00, 0.1713), (2013.00, 0.1592), (2014.00, 0.165), (2015.00, 0.1662), (2016.00, 0.1531), (2017.00, 0.154424), (2018.00, 0.153642), (2019.00, 0.15286), (2020.00, 0.152086), (2021.00, 0.151316), (2022.00, 0.150724), (2023.00, 0.150136), (2024.00, 0.14955), (2025.00, 0.148968), (2026.00, 0.148384), (2027.00, 0.147944), (2028.00, 0.147504), (2029.00, 0.147066), (2030.00, 0.146632), (2031.00, 0.146196), (2032.00, 0.145416), (2033.00, 0.14464), (2034.00, 0.143868), (2035.00, 0.143098), (2036.00, 0.142334), (2037.00, 0.141576), (2038.00, 0.140814), (2039.00, 0.140064), (2040.00, 0.139312), (2041.00, 0.138566), (2042.00, 0.137824), (2043.00, 0.137088), (2044.00, 0.13635), (2045.00, 0.135618), (2046.00, 0.134892), (2047.00, 0.134166), (2048.00, 0.133444), (2049.00, 0.132728), (2050.00, 0.132016), (2051.00, 0.131304), (2052.00, 0.1306), (2053.00, 0.129894), (2054.00, 0.129196), (2055.00, 0.128502), (2056.00, 0.12781), (2057.00, 0.127118), (2058.00, 0.126434), (2059.00, 0.125752), (2060.00, 0.125076), (2061.00, 0.1244)

death_rate_M[F, A99] = GRAPH(TIME)

Points: (2011.00, 0.2964), (2012.00, 0.2945), (2013.00, 0.2778), (2014.00, 0.2895), (2015.00, 0.2889), (2016.00, 0.2569), (2017.00, 0.245044), (2018.00, 0.244874), (2019.00, 0.2447), (2020.00, 0.244526), (2021.00, 0.244354), (2022.00, 0.244218), (2023.00, 0.244084), (2024.00, 0.243954), (2025.00, 0.243818), (2026.00, 0.24369), (2027.00, 0.243588), (2028.00, 0.243486), (2029.00, 0.243386), (2030.00, 0.243286), (2031.00, 0.243186), (2032.00, 0.241764), (2033.00, 0.240348), (2034.00, 0.23894), (2035.00, 0.23754), (2036.00, 0.236148), (2037.00, 0.234764), (2038.00, 0.233384), (2039.00, 0.23201), (2040.00, 0.230648), (2041.00, 0.22929), (2042.00, 0.227938), (2043.00, 0.226594), (2044.00, 0.225262), (2045.00, 0.223928), (2046.00, 0.222608), (2047.00, 0.22129), (2048.00, 0.219984), (2049.00, 0.218684), (2050.00, 0.217386), (2051.00, 0.216098), (2052.00, 0.214818), (2053.00, 0.213548), (2054.00, 0.212276), (2055.00, 0.211016), (2056.00, 0.20976), (2057.00, 0.208514), (2058.00, 0.207272), (2059.00, 0.206038), (2060.00, 0.204814), (2061.00, 0.203592)

death_rate_M[F, A104] = GRAPH(TIME)

Points: (2011.00, 0.4516), (2012.00, 0.4358), (2013.00, 0.4138), (2014.00, 0.4813), (2015.00, 0.4831), (2016.00, 0.488), (2017.00, 0.64349), (2018.00, 0.643425), (2019.00, 0.643355), (2020.00, 0.643285), (2021.00, 0.643215), (2022.00, 0.643165), (2023.00, 0.64311), (2024.00, 0.64306), (2025.00, 0.643005), (2026.00, 0.64295), (2027.00, 0.64291), (2028.00, 0.64287), (2029.00, 0.64283), (2030.00, 0.64279), (2031.00, 0.64275), (2032.00, 0.641785), (2033.00, 0.64082), (2034.00, 0.639865), (2035.00, 0.638915), (2036.00, 0.63797), (2037.00, 0.637025), (2038.00, 0.63609), (2039.00, 0.635165), (2040.00, 0.63424), (2041.00, 0.63332), (2042.00, 0.632405), (2043.00, 0.6315), (2044.00, 0.630595), (2045.00, 0.629695), (2046.00, 0.628805), (2047.00, 0.627915), (2048.00, 0.627035), (2049.00, 0.62616), (2050.00, 0.625285), (2051.00, 0.62442), (2052.00, 0.62356), (2053.00, 0.622705), (2054.00, 0.62185), (2055.00, 0.621005), (2056.00, 0.620165), (2057.00, 0.61933), (2058.00, 0.6185), (2059.00, 0.617675), (2060.00, 0.61685), (2061.00, 0.616035)

Death_rate_switch_2M_3H = 2

UNITS: Dimensionless

Deaths_40[Gender] = SUM(stock_cohort_deaths[Gender,A44:A104])+ stock_cohort_ageing_out[Gender,A104]

Fertility_rate_switch_1L_2M_3H = 2

UNITS: Dimensionless

gender_ratio[M] = GRAPH(TIME)

Points: (2011.000, 0.991), (2011.85714286, 0.991), (2012.71428571, 0.991), (2013.57142857, 0.990), (2014.42857143, 0.988), (2015.28571429, 0.987), (2016.14285714, 0.985), (2017.000, 0.984) {GF EXTRAPOLATED}

UNITS: Dimensionless

gender_ratio[F] = GRAPH(TIME)

Points: (2011.000, 1.009), (2011.85714286, 1.009), (2012.71428571, 1.009), (2013.57142857, 1.010), (2014.42857143, 1.012), (2015.28571429, 1.013), (2016.14285714, 1.015), (2017.000, 1.016)

UNITS: Dimensionless

GS_age_ratio[M, A4] = 0.0686449420303347

UNITS: Dimensionless

GS_age_ratio[M, A9] = 0.0653149658501356

UNITS: Dimensionless

GS_age_ratio[M, A14] = 0.0661376094248548

UNITS: Dimensionless

GS_age_ratio[M, A19] = 0.0679308181955819

UNITS: Dimensionless

GS_age_ratio[M, A24] = 0.069693464647861

UNITS: Dimensionless

GS_age_ratio[M, A29] = 0.0709485885380962

UNITS: Dimensionless

GS_age_ratio[M, A34] = 0.0676748470238414

UNITS: Dimensionless

GS_age_ratio[M, A39] = 0.0702572218758493

UNITS: Dimensionless

GS_age_ratio[M, A44] = 0.0709580863662908

UNITS: Dimensionless

GS_age_ratio[M, A49] = 0.0696784185833942

UNITS: Dimensionless

GS_age_ratio[M, A54] = 0.0669304429843493

UNITS: Dimensionless

GS_age_ratio[M, A59] = 0.0599811359966748

UNITS: Dimensionless

GS_age_ratio[M, A64] = 0.0562238515856201

UNITS: Dimensionless

GS_age_ratio[M, A69] = 0.0428266477086254

UNITS: Dimensionless

GS_age_ratio[M, A74] = 0.0322257549127751

UNITS: Dimensionless

GS_age_ratio[M, A79] = 0.0237849127469318

UNITS: Dimensionless

GS_age_ratio[M, A84] = 0.0177017888830272

UNITS: Dimensionless

GS_age_ratio[M, A89] = 0.00947930272775745

UNITS: Dimensionless

GS_age_ratio[M, A94] = 0.00298984108534786

UNITS: Dimensionless

GS_age_ratio[M, A99] = 0.000555669968337438

UNITS: Dimensionless

GS_age_ratio[M, A104] = 0.0000616888643136503

UNITS: Dimensionless

GS_age_ratio[F, A4] = 0.0635549603166027

UNITS: Dimensionless

GS_age_ratio[F, A9] = 0.0604544491838144

UNITS: Dimensionless

GS_age_ratio[F, A14] = 0.0614096906009192

UNITS: Dimensionless

GS_age_ratio[F, A19] = 0.0628503237769033

UNITS: Dimensionless

GS_age_ratio[F, A24] = 0.066173942224402

UNITS: Dimensionless

GS_age_ratio[F, A29] = 0.0697804452968178

UNITS: Dimensionless

GS_age_ratio[F, A34] = 0.0675135977286829

UNITS: Dimensionless

GS_age_ratio[F, A39] = 0.0710907639365511

UNITS: Dimensionless

GS_age_ratio[F, A44] = 0.0724969102028005

UNITS: Dimensionless

GS_age_ratio[F, A49] = 0.07018573546002

UNITS: Dimensionless

GS_age_ratio[F, A54] = 0.0676550400411415

UNITS: Dimensionless

GS_age_ratio[F, A59] = 0.060642333868049

UNITS: Dimensionless

GS_age_ratio[F, A64] = 0.0559360202529206

UNITS: Dimensionless

GS_age_ratio[F, A69] = 0.042662422538952

UNITS: Dimensionless

GS_age_ratio[F, A74] = 0.033603768950212

UNITS: Dimensionless

GS_age_ratio[F, A79] = 0.0268843394575034

UNITS: Dimensionless

GS_age_ratio[F, A84] = 0.0228711667454961

UNITS: Dimensionless

GS_age_ratio[F, A89] = 0.0156334219907481

UNITS: Dimensionless

GS_age_ratio[F, A94] = 0.00661973933230216

UNITS: Dimensionless

GS_age_ratio[F, A99] = 0.00174025544775918

UNITS: Dimensionless

GS_age_ratio[F, A104] = 0.00024067264740188

UNITS: Dimensionless

"Migrants_40P_M\\F"[Gender] = SUM(Net_Immigration[Gender,A44:A104])

Net_Arrivals[Gender, Age] = Overseas_arrivals+NIM_in

UNITS: persons/year

Net_Departures[Gender, Age] = NOM_out+NIM_out

UNITS: persons/year

Net_Immigration[Gender, Age] = Net_Arrivals-Net_Departures

NIM_Arrivals_H[M, A4] = GRAPH(TIME)

Points: (2011.00, 14810.0), (2012.00, 13430.0), (2013.00, 14270.0), (2014.00, 13530.0), (2015.00, 14830.0), (2016.00, 16080.0), (2017.00, 16070.0), (2018.00, 15634.0), (2019.00, 15977.0), (2020.00, 16311.0), (2021.00, 16637.0), (2022.00, 16947.0), (2023.00, 17244.0), (2024.00, 17527.0), (2025.00, 17793.0), (2026.00, 18049.0), (2027.00, 18290.0), (2028.00, 18514.0), (2029.00, 18722.0), (2030.00, 18914.0), (2031.00, 19094.0), (2032.00, 19272.0), (2033.00, 19452.0), (2034.00, 19641.0), (2035.00, 19839.0), (2036.00, 20048.0), (2037.00, 20270.0), (2038.00, 20504.0), (2039.00, 20750.0), (2040.00, 21013.0), (2041.00, 21284.0), (2042.00, 21567.0), (2043.00, 21862.0), (2044.00, 22163.0), (2045.00, 22469.0), (2046.00, 22781.0), (2047.00, 23094.0), (2048.00, 23408.0), (2049.00, 23722.0), (2050.00, 24032.0), (2051.00, 24338.0), (2052.00, 24639.0), (2053.00, 24934.0), (2054.00, 25220.0), (2055.00, 25498.0), (2056.00, 25769.0), (2057.00, 26030.0), (2058.00, 26284.0), (2059.00, 26529.0), (2060.00, 26768.0), (2061.00, 27000.0)

UNITS: persons/year

NIM_Arrivals_H[M, A9] = GRAPH(TIME)

Points: (2011.00, 10830.0), (2012.00, 9840.0), (2013.00, 10930.0), (2014.00, 10360.0), (2015.00, 11460.0), (2016.00, 12550.0), (2017.00, 12610.0), (2018.00, 11770.0), (2019.00, 12035.0), (2020.00, 12311.0), (2021.00, 12643.0), (2022.00, 12924.0), (2023.00, 13200.0), (2024.00, 13474.0), (2025.00, 13740.0), (2026.00, 13999.0), (2027.00, 14245.0), (2028.00, 14480.0), (2029.00, 14703.0), (2030.00, 14915.0), (2031.00, 15118.0), (2032.00, 15309.0), (2033.00, 15484.0), (2034.00, 15646.0), (2035.00, 15800.0), (2036.00, 15944.0), (2037.00, 16088.0), (2038.00, 16234.0), (2039.00, 16387.0), (2040.00, 16546.0), (2041.00, 16716.0), (2042.00, 16895.0), (2043.00, 17085.0), (2044.00, 17285.0), (2045.00, 17496.0), (2046.00, 17715.0), (2047.00, 17943.0), (2048.00, 18180.0), (2049.00, 18421.0), (2050.00, 18668.0), (2051.00, 18917.0), (2052.00, 19168.0), (2053.00, 19421.0), (2054.00, 19669.0), (2055.00, 19917.0), (2056.00, 20162.0), (2057.00, 20402.0), (2058.00, 20636.0), (2059.00, 20865.0), (2060.00, 21086.0), (2061.00, 21302.0)

UNITS: persons/year

NIM_Arrivals_H[M, A14] = GRAPH(TIME)

Points: (2011.00, 8420.0), (2012.00, 7360.0), (2013.00, 8220.0), (2014.00, 7640.0), (2015.00, 8350.0), (2016.00, 9020.0), (2017.00, 9010.0), (2018.00, 8387.0), (2019.00, 8590.0), (2020.00, 8781.0), (2021.00, 8898.0), (2022.00, 9083.0), (2023.00, 9276.0), (2024.00, 9474.0), (2025.00, 9681.0), (2026.00, 9931.0), (2027.00, 10141.0), (2028.00, 10347.0), (2029.00, 10553.0), (2030.00, 10752.0), (2031.00, 10945.0), (2032.00, 11129.0), (2033.00, 11306.0), (2034.00, 11472.0), (2035.00, 11630.0), (2036.00, 11784.0), (2037.00, 11926.0), (2038.00, 12057.0), (2039.00, 12178.0), (2040.00, 12292.0), (2041.00, 12400.0), (2042.00, 12507.0), (2043.00, 12616.0), (2044.00, 12729.0), (2045.00, 12849.0), (2046.00, 12976.0), (2047.00, 13108.0), (2048.00, 13251.0), (2049.00, 13401.0), (2050.00, 13558.0), (2051.00, 13723.0), (2052.00, 13893.0), (2053.00, 14069.0), (2054.00, 14250.0), (2055.00, 14434.0), (2056.00, 14621.0), (2057.00, 14808.0), (2058.00, 14997.0), (2059.00, 15183.0), (2060.00, 15370.0), (2061.00, 15552.0)

UNITS: persons/year

NIM_Arrivals_H[M, A19] = GRAPH(TIME)

Points: (2011.00, 11990.0), (2012.00, 11040.0), (2013.00, 11660.0), (2014.00, 10790.0), (2015.00, 10870.0), (2016.00, 11300.0), (2017.00, 11040.0), (2018.00, 11389.0), (2019.00, 11493.0), (2020.00, 11607.0), (2021.00, 11754.0), (2022.00, 11994.0), (2023.00, 12279.0), (2024.00, 12593.0), (2025.00, 12869.0), (2026.00, 13037.0), (2027.00, 13281.0), (2028.00, 13513.0), (2029.00, 13721.0), (2030.00, 13967.0), (2031.00, 14344.0), (2032.00, 14621.0), (2033.00, 14893.0), (2034.00, 15165.0), (2035.00, 15433.0), (2036.00, 15693.0), (2037.00, 15946.0), (2038.00, 16184.0), (2039.00, 16412.0), (2040.00, 16627.0), (2041.00, 16833.0), (2042.00, 17027.0), (2043.00, 17207.0), (2044.00, 17378.0), (2045.00, 17535.0), (2046.00, 17678.0), (2047.00, 17815.0), (2048.00, 17955.0), (2049.00, 18098.0), (2050.00, 18248.0), (2051.00, 18408.0), (2052.00, 18576.0), (2053.00, 18754.0), (2054.00, 18945.0), (2055.00, 19142.0), (2056.00, 19351.0), (2057.00, 19571.0), (2058.00, 19799.0), (2059.00, 20033.0), (2060.00, 20273.0), (2061.00, 20517.0)

UNITS: persons/year

NIM_Arrivals_H[M, A24] = GRAPH(TIME)

Points: (2011.00, 23570.0), (2012.00, 23740.0), (2013.00, 24100.0), (2014.00, 24480.0), (2015.00, 21950.0), (2016.00, 22400.0), (2017.00, 22440.0), (2018.00, 25445.0), (2019.00, 25604.0), (2020.00, 25662.0), (2021.00, 25819.0), (2022.00, 25935.0), (2023.00, 26097.0), (2024.00, 26304.0), (2025.00, 26597.0), (2026.00, 27006.0), (2027.00, 27535.0), (2028.00, 28130.0), (2029.00, 28712.0), (2030.00, 29248.0), (2031.00, 29591.0), (2032.00, 30078.0), (2033.00, 30557.0), (2034.00, 31069.0), (2035.00, 31613.0), (2036.00, 32319.0), (2037.00, 32887.0), (2038.00, 33444.0), (2039.00, 33998.0), (2040.00, 34543.0), (2041.00, 35069.0), (2042.00, 35572.0), (2043.00, 36051.0), (2044.00, 36508.0), (2045.00, 36940.0), (2046.00, 37352.0), (2047.00, 37741.0), (2048.00, 38101.0), (2049.00, 38437.0), (2050.00, 38749.0), (2051.00, 39038.0), (2052.00, 39319.0), (2053.00, 39607.0), (2054.00, 39908.0), (2055.00, 40221.0), (2056.00, 40552.0), (2057.00, 40903.0), (2058.00, 41276.0), (2059.00, 41671.0), (2060.00, 42087.0), (2061.00, 42522.0)

UNITS: persons/year

NIM_Arrivals_H[M, A29] = GRAPH(TIME)

Points: (2011.00, 27820.0), (2012.00, 28020.0), (2013.00, 28720.0), (2014.00, 28290.0), (2015.00, 27110.0), (2016.00, 27720.0), (2017.00, 27340.0), (2018.00, 29706.0), (2019.00, 29968.0), (2020.00, 30209.0), (2021.00, 30451.0), (2022.00, 30736.0), (2023.00, 30962.0), (2024.00, 31140.0), (2025.00, 31244.0), (2026.00, 31437.0), (2027.00, 31570.0), (2028.00, 31753.0), (2029.00, 32017.0), (2030.00, 32397.0), (2031.00, 32930.0), (2032.00, 33531.0), (2033.00, 34188.0), (2034.00, 34819.0), (2035.00, 35405.0), (2036.00, 35769.0), (2037.00, 36338.0), (2038.00, 36917.0), (2039.00, 37524.0), (2040.00, 38158.0), (2041.00, 38921.0), (2042.00, 39565.0), (2043.00, 40196.0), (2044.00, 40825.0), (2045.00, 41436.0), (2046.00, 42029.0), (2047.00, 42595.0), (2048.00, 43133.0), (2049.00, 43647.0), (2050.00, 44135.0), (2051.00, 44601.0), (2052.00, 45036.0), (2053.00, 45440.0), (2054.00, 45815.0), (2055.00, 46165.0), (2056.00, 46497.0), (2057.00, 46822.0), (2058.00, 47157.0), (2059.00, 47503.0), (2060.00, 47870.0), (2061.00, 48254.0)

UNITS: persons/year

NIM_Arrivals_H[M, A34] = GRAPH(TIME)

Points: (2011.00, 19850.0), (2012.00, 20200.0), (2013.00, 21510.0), (2014.00, 21440.0), (2015.00, 22020.0), (2016.00, 23380.0), (2017.00, 23290.0), (2018.00, 24211.0), (2019.00, 24629.0), (2020.00, 25080.0), (2021.00, 25402.0), (2022.00, 25658.0), (2023.00, 25909.0), (2024.00, 26126.0), (2025.00, 26324.0), (2026.00, 26524.0), (2027.00, 26750.0), (2028.00, 26928.0), (2029.00, 27074.0), (2030.00, 27163.0), (2031.00, 27316.0), (2032.00, 27423.0), (2033.00, 27570.0), (2034.00, 27787.0), (2035.00, 28096.0), (2036.00, 28528.0), (2037.00, 29012.0), (2038.00, 29535.0), (2039.00, 30036.0), (2040.00, 30502.0), (2041.00, 30794.0), (2042.00, 31247.0), (2043.00, 31717.0), (2044.00, 32204.0), (2045.00, 32713.0), (2046.00, 33321.0), (2047.00, 33836.0), (2048.00, 34340.0), (2049.00, 34842.0), (2050.00, 35332.0), (2051.00, 35806.0), (2052.00, 36258.0), (2053.00, 36691.0), (2054.00, 37100.0), (2055.00, 37490.0), (2056.00, 37862.0), (2057.00, 38211.0), (2058.00, 38535.0), (2059.00, 38835.0), (2060.00, 39113.0), (2061.00, 39379.0)

UNITS: persons/year

NIM_Arrivals_H[M, A39] = GRAPH(TIME)

Points: (2011.00, 15620.0), (2012.00, 14640.0), (2013.00, 15300.0), (2014.00, 14700.0), (2015.00, 15530.0), (2016.00, 16600.0), (2017.00, 17130.0), (2018.00, 17532.0), (2019.00, 18164.0), (2020.00, 18769.0), (2021.00, 19252.0), (2022.00, 19632.0), (2023.00, 19923.0), (2024.00, 20248.0), (2025.00, 20603.0), (2026.00, 20845.0), (2027.00, 21037.0), (2028.00, 21231.0), (2029.00, 21403.0), (2030.00, 21559.0), (2031.00, 21713.0), (2032.00, 21887.0), (2033.00, 22022.0), (2034.00, 22136.0), (2035.00, 22207.0), (2036.00, 22326.0), (2037.00, 22409.0), (2038.00, 22523.0), (2039.00, 22693.0), (2040.00, 22936.0), (2041.00, 23273.0), (2042.00, 23645.0), (2043.00, 24047.0), (2044.00, 24431.0), (2045.00, 24792.0), (2046.00, 25013.0), (2047.00, 25367.0), (2048.00, 25728.0), (2049.00, 26108.0), (2050.00, 26498.0), (2051.00, 26965.0), (2052.00, 27363.0), (2053.00, 27752.0), (2054.00, 28138.0), (2055.00, 28516.0), (2056.00, 28881.0), (2057.00, 29230.0), (2058.00, 29562.0), (2059.00, 29878.0), (2060.00, 30178.0), (2061.00, 30466.0)

UNITS: persons/year

NIM_Arrivals_H[M, A44] = GRAPH(TIME)

Points: (2011.00, 12210.0), (2012.00, 11620.0), (2013.00, 12500.0), (2014.00, 11990.0), (2015.00, 12090.0), (2016.00, 12590.0), (2017.00, 12530.0), (2018.00, 11666.0), (2019.00, 11814.0), (2020.00, 12059.0), (2021.00, 12451.0), (2022.00, 12902.0), (2023.00, 13423.0), (2024.00, 13884.0), (2025.00, 14324.0), (2026.00, 14673.0), (2027.00, 14948.0), (2028.00, 15161.0), (2029.00, 15396.0), (2030.00, 15652.0), (2031.00, 15831.0), (2032.00, 15976.0), (2033.00, 16113.0), (2034.00, 16239.0), (2035.00, 16357.0), (2036.00, 16472.0), (2037.00, 16601.0), (2038.00, 16701.0), (2039.00, 16784.0), (2040.00, 16838.0), (2041.00, 16926.0), (2042.00, 16988.0), (2043.00, 17072.0), (2044.00, 17197.0), (2045.00, 17372.0), (2046.00, 17618.0), (2047.00, 17893.0), (2048.00, 18188.0), (2049.00, 18466.0), (2050.00, 18727.0), (2051.00, 18891.0), (2052.00, 19149.0), (2053.00, 19413.0), (2054.00, 19690.0), (2055.00, 19979.0), (2056.00, 20317.0), (2057.00, 20607.0), (2058.00, 20892.0), (2059.00, 21174.0), (2060.00, 21450.0), (2061.00, 21717.0)

UNITS: persons/year

NIM_Arrivals_H[M, A49] = GRAPH(TIME)

Points: (2011.00, 9080.0), (2012.00, 8390.0), (2013.00, 9000.0), (2014.00, 8670.0), (2015.00, 8840.0), (2016.00, 9870.0), (2017.00, 10210.0), (2018.00, 9549.0), (2019.00, 9614.0), (2020.00, 9622.0), (2021.00, 9525.0), (2022.00, 9462.0), (2023.00, 9483.0), (2024.00, 9596.0), (2025.00, 9782.0), (2026.00, 10083.0), (2027.00, 10431.0), (2028.00, 10841.0), (2029.00, 11206.0), (2030.00, 11558.0), (2031.00, 11838.0), (2032.00, 12062.0), (2033.00, 12234.0), (2034.00, 12424.0), (2035.00, 12624.0), (2036.00, 12768.0), (2037.00, 12887.0), (2038.00, 13005.0), (2039.00, 13107.0), (2040.00, 13197.0), (2041.00, 13289.0), (2042.00, 13395.0), (2043.00, 13479.0), (2044.00, 13549.0), (2045.00, 13592.0), (2046.00, 13661.0), (2047.00, 13714.0), (2048.00, 13781.0), (2049.00, 13882.0), (2050.00, 14018.0), (2051.00, 14209.0), (2052.00, 14424.0), (2053.00, 14659.0), (2054.00, 14882.0), (2055.00, 15093.0), (2056.00, 15226.0), (2057.00, 15430.0), (2058.00, 15637.0), (2059.00, 15856.0), (2060.00, 16081.0), (2061.00, 16354.0)

UNITS: persons/year

NIM_Arrivals_H[M, A54] = GRAPH(TIME)

Points: (2011.00, 7390.0), (2012.00, 7260.0), (2013.00, 7660.0), (2014.00, 7140.0), (2015.00, 7250.0), (2016.00, 7820.0), (2017.00, 8150.0), (2018.00, 7665.0), (2019.00, 7754.0), (2020.00, 7920.0), (2021.00, 8192.0), (2022.00, 8432.0), (2023.00, 8577.0), (2024.00, 8639.0), (2025.00, 8654.0), (2026.00, 8569.0), (2027.00, 8514.0), (2028.00, 8528.0), (2029.00, 8627.0), (2030.00, 8790.0), (2031.00, 9055.0), (2032.00, 9361.0), (2033.00, 9722.0), (2034.00, 10046.0), (2035.00, 10361.0), (2036.00, 10614.0), (2037.00, 10817.0), (2038.00, 10971.0), (2039.00, 11143.0), (2040.00, 11322.0), (2041.00, 11451.0), (2042.00, 11561.0), (2043.00, 11669.0), (2044.00, 11763.0), (2045.00, 11843.0), (2046.00, 11926.0), (2047.00, 12024.0), (2048.00, 12102.0), (2049.00, 12167.0), (2050.00, 12205.0), (2051.00, 12270.0), (2052.00, 12317.0), (2053.00, 12381.0), (2054.00, 12470.0), (2055.00, 12592.0), (2056.00, 12761.0), (2057.00, 12951.0), (2058.00, 13162.0), (2059.00, 13364.0), (2060.00, 13555.0), (2061.00, 13675.0)

UNITS: persons/year

NIM_Arrivals_H[M, A59] = GRAPH(TIME)

Points: (2011.00, 6140.0), (2012.00, 6060.0), (2013.00, 6410.0), (2014.00, 6010.0), (2015.00, 6210.0), (2016.00, 6950.0), (2017.00, 7570.0), (2018.00, 7333.0), (2019.00, 7402.0), (2020.00, 7418.0), (2021.00, 7374.0), (2022.00, 7340.0), (2023.00, 7352.0), (2024.00, 7440.0), (2025.00, 7603.0), (2026.00, 7864.0), (2027.00, 8098.0), (2028.00, 8244.0), (2029.00, 8306.0), (2030.00, 8320.0), (2031.00, 8242.0), (2032.00, 8195.0), (2033.00, 8214.0), (2034.00, 8310.0), (2035.00, 8470.0), (2036.00, 8724.0), (2037.00, 9023.0), (2038.00, 9370.0), (2039.00, 9685.0), (2040.00, 9989.0), (2041.00, 10232.0), (2042.00, 10432.0), (2043.00, 10583.0), (2044.00, 10751.0), (2045.00, 10926.0), (2046.00, 11057.0), (2047.00, 11166.0), (2048.00, 11272.0), (2049.00, 11367.0), (2050.00, 11447.0), (2051.00, 11531.0), (2052.00, 11629.0), (2053.00, 11708.0), (2054.00, 11771.0), (2055.00, 11813.0), (2056.00, 11879.0), (2057.00, 11929.0), (2058.00, 11994.0), (2059.00, 12083.0), (2060.00, 12203.0), (2061.00, 12368.0)

UNITS: persons/year

NIM_Arrivals_H[M, A64] = GRAPH(TIME)

Points: (2011.00, 5140.0), (2012.00, 5040.0), (2013.00, 5480.0), (2014.00, 5080.0), (2015.00, 5400.0), (2016.00, 6100.0), (2017.00, 6370.0), (2018.00, 5865.0), (2019.00, 6004.0), (2020.00, 6128.0), (2021.00, 6269.0), (2022.00, 6406.0), (2023.00, 6512.0), (2024.00, 6578.0), (2025.00, 6594.0), (2026.00, 6560.0), (2027.00, 6537.0), (2028.00, 6559.0), (2029.00, 6648.0), (2030.00, 6800.0), (2031.00, 7040.0), (2032.00, 7248.0), (2033.00, 7376.0), (2034.00, 7429.0), (2035.00, 7446.0), (2036.00, 7385.0), (2037.00, 7348.0), (2038.00, 7374.0), (2039.00, 7469.0), (2040.00, 7620.0), (2041.00, 7856.0), (2042.00, 8129.0), (2043.00, 8445.0), (2044.00, 8726.0), (2045.00, 9001.0), (2046.00, 9223.0), (2047.00, 9401.0), (2048.00, 9542.0), (2049.00, 9697.0), (2050.00, 9862.0), (2051.00, 9980.0), (2052.00, 10078.0), (2053.00, 10177.0), (2054.00, 10265.0), (2055.00, 10344.0), (2056.00, 10422.0), (2057.00, 10512.0), (2058.00, 10586.0), (2059.00, 10647.0), (2060.00, 10690.0), (2061.00, 10752.0)

UNITS: persons/year

NIM_Arrivals_H[M, A69] = GRAPH(TIME)

Points: (2011.00, 3110.0), (2012.00, 3470.0), (2013.00, 4010.0), (2014.00, 3820.0), (2015.00, 4290.0), (2016.00, 4930.0), (2017.00, 4950.0), (2018.00, 4319.0), (2019.00, 4390.0), (2020.00, 4471.0), (2021.00, 4576.0), (2022.00, 4678.0), (2023.00, 4778.0), (2024.00, 4895.0), (2025.00, 5004.0), (2026.00, 5128.0), (2027.00, 5247.0), (2028.00, 5338.0), (2029.00, 5397.0), (2030.00, 5410.0), (2031.00, 5388.0), (2032.00, 5373.0), (2033.00, 5398.0), (2034.00, 5481.0), (2035.00, 5614.0), (2036.00, 5819.0), (2037.00, 5995.0), (2038.00, 6100.0), (2039.00, 6143.0), (2040.00, 6151.0), (2041.00, 6106.0), (2042.00, 6082.0), (2043.00, 6110.0), (2044.00, 6193.0), (2045.00, 6325.0), (2046.00, 6528.0), (2047.00, 6759.0), (2048.00, 7024.0), (2049.00, 7262.0), (2050.00, 7489.0), (2051.00, 7672.0), (2052.00, 7822.0), (2053.00, 7941.0), (2054.00, 8072.0), (2055.00, 8212.0), (2056.00, 8316.0), (2057.00, 8399.0), (2058.00, 8482.0), (2059.00, 8557.0), (2060.00, 8630.0), (2061.00, 8702.0)

UNITS: persons/year

NIM_Arrivals_H[M, A74] = GRAPH(TIME)

Points: (2011.00, 1710.0), (2012.00, 1850.0), (2013.00, 2160.0), (2014.00, 2030.0), (2015.00, 2400.0), (2016.00, 2800.0), (2017.00, 3210.0), (2018.00, 2859.0), (2019.00, 2957.0), (2020.00, 3054.0), (2021.00, 3128.0), (2022.00, 3136.0), (2023.00, 3185.0), (2024.00, 3245.0), (2025.00, 3313.0), (2026.00, 3398.0), (2027.00, 3478.0), (2028.00, 3561.0), (2029.00, 3659.0), (2030.00, 3746.0), (2031.00, 3846.0), (2032.00, 3943.0), (2033.00, 4017.0), (2034.00, 4068.0), (2035.00, 4085.0), (2036.00, 4073.0), (2037.00, 4067.0), (2038.00, 4088.0), (2039.00, 4155.0), (2040.00, 4257.0), (2041.00, 4416.0), (2042.00, 4554.0), (2043.00, 4637.0), (2044.00, 4677.0), (2045.00, 4689.0), (2046.00, 4657.0), (2047.00, 4643.0), (2048.00, 4665.0), (2049.00, 4730.0), (2050.00, 4831.0), (2051.00, 4986.0), (2052.00, 5166.0), (2053.00, 5370.0), (2054.00, 5556.0), (2055.00, 5734.0), (2056.00, 5876.0), (2057.00, 5993.0), (2058.00, 6089.0), (2059.00, 6192.0), (2060.00, 6301.0), (2061.00, 6382.0)

UNITS: persons/year

NIM_Arrivals_H[M, A79] = GRAPH(TIME)

Points: (2011.00, 902.0), (2012.00, 941.0), (2013.00, 1089.0), (2014.00, 1032.0), (2015.00, 1107.0), (2016.00, 1207.0), (2017.00, 1268.0), (2018.00, 1526.0), (2019.00, 1605.0), (2020.00, 1691.0), (2021.00, 1779.0), (2022.00, 1924.0), (2023.00, 2044.0), (2024.00, 2124.0), (2025.00, 2199.0), (2026.00, 2265.0), (2027.00, 2275.0), (2028.00, 2318.0), (2029.00, 2368.0), (2030.00, 2426.0), (2031.00, 2494.0), (2032.00, 2561.0), (2033.00, 2627.0), (2034.00, 2706.0), (2035.00, 2779.0), (2036.00, 2857.0), (2037.00, 2934.0), (2038.00, 2997.0), (2039.00, 3042.0), (2040.00, 3062.0), (2041.00, 3057.0), (2042.00, 3056.0), (2043.00, 3072.0), (2044.00, 3123.0), (2045.00, 3203.0), (2046.00, 3324.0), (2047.00, 3435.0), (2048.00, 3506.0), (2049.00, 3545.0), (2050.00, 3563.0), (2051.00, 3540.0), (2052.00, 3530.0), (2053.00, 3548.0), (2054.00, 3597.0), (2055.00, 3676.0), (2056.00, 3796.0), (2057.00, 3934.0), (2058.00, 4092.0), (2059.00, 4238.0), (2060.00, 4377.0), (2061.00, 4492.0)

UNITS: persons/year

NIM_Arrivals_H[M, A84] = GRAPH(TIME)

Points: (2011.00, 671.0), (2012.00, 700.0), (2013.00, 810.0), (2014.00, 768.0), (2015.00, 823.0), (2016.00, 898.0), (2017.00, 943.0), (2018.00, 935.0), (2019.00, 983.0), (2020.00, 1035.0), (2021.00, 1085.0), (2022.00, 1141.0), (2023.00, 1188.0), (2024.00, 1259.0), (2025.00, 1329.0), (2026.00, 1409.0), (2027.00, 1533.0), (2028.00, 1636.0), (2029.00, 1703.0), (2030.00, 1768.0), (2031.00, 1829.0), (2032.00, 1846.0), (2033.00, 1889.0), (2034.00, 1938.0), (2035.00, 1993.0), (2036.00, 2058.0), (2037.00, 2121.0), (2038.00, 2184.0), (2039.00, 2255.0), (2040.00, 2323.0), (2041.00, 2398.0), (2042.00, 2469.0), (2043.00, 2528.0), (2044.00, 2571.0), (2045.00, 2592.0), (2046.00, 2595.0), (2047.00, 2601.0), (2048.00, 2625.0), (2049.00, 2676.0), (2050.00, 2752.0), (2051.00, 2863.0), (2052.00, 2962.0), (2053.00, 3029.0), (2054.00, 3064.0), (2055.00, 3085.0), (2056.00, 3073.0), (2057.00, 3071.0), (2058.00, 3092.0), (2059.00, 3143.0), (2060.00, 3217.0), (2061.00, 3328.0)

UNITS: persons/year

NIM_Arrivals_H[M, A89] = GRAPH(TIME)

Points: (2011.00, 359.0), (2012.00, 375.0), (2013.00, 434.0), (2014.00, 411.0), (2015.00, 441.0), (2016.00, 481.0), (2017.00, 505.0), (2018.00, 519.0), (2019.00, 528.0), (2020.00, 537.0), (2021.00, 556.0), (2022.00, 584.0), (2023.00, 615.0), (2024.00, 653.0), (2025.00, 690.0), (2026.00, 728.0), (2027.00, 771.0), (2028.00, 810.0), (2029.00, 863.0), (2030.00, 917.0), (2031.00, 980.0), (2032.00, 1072.0), (2033.00, 1156.0), (2034.00, 1219.0), (2035.00, 1280.0), (2036.00, 1332.0), (2037.00, 1356.0), (2038.00, 1398.0), (2039.00, 1446.0), (2040.00, 1498.0), (2041.00, 1557.0), (2042.00, 1617.0), (2043.00, 1678.0), (2044.00, 1745.0), (2045.00, 1809.0), (2046.00, 1879.0), (2047.00, 1947.0), (2048.00, 2009.0), (2049.00, 2060.0), (2050.00, 2091.0), (2051.00, 2108.0), (2052.00, 2122.0), (2053.00, 2148.0), (2054.00, 2197.0), (2055.00, 2268.0), (2056.00, 2369.0), (2057.00, 2467.0), (2058.00, 2543.0), (2059.00, 2592.0), (2060.00, 2624.0), (2061.00, 2624.0)

UNITS: persons/year

NIM_Arrivals_H[M, A94] = GRAPH(TIME)

Points: (2011.00, 113.0), (2012.00, 118.0), (2013.00, 136.0), (2014.00, 129.0), (2015.00, 139.0), (2016.00, 151.0), (2017.00, 159.0), (2018.00, 224.0), (2019.00, 234.0), (2020.00, 246.0), (2021.00, 255.0), (2022.00, 261.0), (2023.00, 266.0), (2024.00, 273.0), (2025.00, 281.0), (2026.00, 295.0), (2027.00, 314.0), (2028.00, 333.0), (2029.00, 355.0), (2030.00, 380.0), (2031.00, 403.0), (2032.00, 432.0), (2033.00, 458.0), (2034.00, 497.0), (2035.00, 539.0), (2036.00, 582.0), (2037.00, 654.0), (2038.00, 708.0), (2039.00, 748.0), (2040.00, 794.0), (2041.00, 832.0), (2042.00, 858.0), (2043.00, 901.0), (2044.00, 940.0), (2045.00, 987.0), (2046.00, 1040.0), (2047.00, 1090.0), (2048.00, 1142.0), (2049.00, 1200.0), (2050.00, 1255.0), (2051.00, 1315.0), (2052.00, 1378.0), (2053.00, 1429.0), (2054.00, 1474.0), (2055.00, 1504.0), (2056.00, 1526.0), (2057.00, 1551.0), (2058.00, 1586.0), (2059.00, 1640.0), (2060.00, 1710.0), (2061.00, 1805.0)

UNITS: persons/year

NIM_Arrivals_H[M, A99] = GRAPH(TIME)

Points: (2011.00, 21.0), (2012.00, 21.0), (2013.00, 25.0), (2014.00, 24.0), (2015.00, 25.0), (2016.00, 28.0), (2017.00, 29.0), (2018.00, 51.0), (2019.00, 56.0), (2020.00, 60.0), (2021.00, 64.0), (2022.00, 67.0), (2023.00, 71.0), (2024.00, 74.0), (2025.00, 78.0), (2026.00, 81.0), (2027.00, 84.0), (2028.00, 85.0), (2029.00, 88.0), (2030.00, 92.0), (2031.00, 97.0), (2032.00, 105.0), (2033.00, 112.0), (2034.00, 122.0), (2035.00, 134.0), (2036.00, 147.0), (2037.00, 162.0), (2038.00, 177.0), (2039.00, 197.0), (2040.00, 219.0), (2041.00, 243.0), (2042.00, 280.0), (2043.00, 310.0), (2044.00, 335.0), (2045.00, 362.0), (2046.00, 387.0), (2047.00, 410.0), (2048.00, 439.0), (2049.00, 467.0), (2050.00, 499.0), (2051.00, 538.0), (2052.00, 574.0), (2053.00, 612.0), (2054.00, 654.0), (2055.00, 698.0), (2056.00, 744.0), (2057.00, 792.0), (2058.00, 834.0), (2059.00, 873.0), (2060.00, 904.0), (2061.00, 931.0)

UNITS: persons/year

NIM_Arrivals_H[M, A104] = GRAPH(TIME)

Points: (2011.00, 2.0), (2012.00, 2.0), (2013.00, 2.0), (2014.00, 2.0), (2015.00, 2.0), (2016.00, 3.0), (2017.00, 3.0), (2018.00, 7.0), (2019.00, 7.0), (2020.00, 9.0), (2021.00, 10.0), (2022.00, 11.0), (2023.00, 12.0), (2024.00, 13.0), (2025.00, 14.0), (2026.00, 15.0), (2027.00, 16.0), (2028.00, 18.0), (2029.00, 19.0), (2030.00, 20.0), (2031.00, 20.0), (2032.00, 21.0), (2033.00, 22.0), (2034.00, 24.0), (2035.00, 25.0), (2036.00, 27.0), (2037.00, 30.0), (2038.00, 34.0), (2039.00, 38.0), (2040.00, 42.0), (2041.00, 48.0), (2042.00, 54.0), (2043.00, 61.0), (2044.00, 69.0), (2045.00, 79.0), (2046.00, 90.0), (2047.00, 107.0), (2048.00, 121.0), (2049.00, 134.0), (2050.00, 149.0), (2051.00, 164.0), (2052.00, 180.0), (2053.00, 198.0), (2054.00, 217.0), (2055.00, 238.0), (2056.00, 261.0), (2057.00, 285.0), (2058.00, 311.0), (2059.00, 339.0), (2060.00, 368.0), (2061.00, 401.0)

UNITS: persons/year

NIM_Arrivals_H[F, A4] = GRAPH(TIME)

Points: (2011.00, 13960.0), (2012.00, 12760.0), (2013.00, 13070.0), (2014.00, 12660.0), (2015.00, 14180.0), (2016.00, 14990.0), (2017.00, 14890.0), (2018.00, 14843.0), (2019.00, 15166.0), (2020.00, 15483.0), (2021.00, 15791.0), (2022.00, 16084.0), (2023.00, 16364.0), (2024.00, 16630.0), (2025.00, 16885.0), (2026.00, 17128.0), (2027.00, 17357.0), (2028.00, 17569.0), (2029.00, 17762.0), (2030.00, 17946.0), (2031.00, 18116.0), (2032.00, 18284.0), (2033.00, 18456.0), (2034.00, 18634.0), (2035.00, 18821.0), (2036.00, 19018.0), (2037.00, 19228.0), (2038.00, 19449.0), (2039.00, 19683.0), (2040.00, 19929.0), (2041.00, 20187.0), (2042.00, 20455.0), (2043.00, 20733.0), (2044.00, 21017.0), (2045.00, 21308.0), (2046.00, 21602.0), (2047.00, 21900.0), (2048.00, 22197.0), (2049.00, 22493.0), (2050.00, 22787.0), (2051.00, 23076.0), (2052.00, 23361.0), (2053.00, 23639.0), (2054.00, 23911.0), (2055.00, 24174.0), (2056.00, 24430.0), (2057.00, 24677.0), (2058.00, 24916.0), (2059.00, 25149.0), (2060.00, 25376.0), (2061.00, 25596.0)

UNITS: persons/year

NIM_Arrivals_H[F, A9] = GRAPH(TIME)

Points: (2011.00, 10430.0), (2012.00, 9540.0), (2013.00, 10480.0), (2014.00, 10080.0), (2015.00, 11030.0), (2016.00, 11750.0), (2017.00, 11820.0), (2018.00, 11304.0), (2019.00, 11563.0), (2020.00, 11835.0), (2021.00, 12155.0), (2022.00, 12431.0), (2023.00, 12695.0), (2024.00, 12956.0), (2025.00, 13211.0), (2026.00, 13456.0), (2027.00, 13692.0), (2028.00, 13916.0), (2029.00, 14128.0), (2030.00, 14330.0), (2031.00, 14524.0), (2032.00, 14705.0), (2033.00, 14873.0), (2034.00, 15027.0), (2035.00, 15173.0), (2036.00, 15311.0), (2037.00, 15446.0), (2038.00, 15586.0), (2039.00, 15731.0), (2040.00, 15883.0), (2041.00, 16044.0), (2042.00, 16215.0), (2043.00, 16395.0), (2044.00, 16586.0), (2045.00, 16786.0), (2046.00, 16995.0), (2047.00, 17213.0), (2048.00, 17438.0), (2049.00, 17668.0), (2050.00, 17902.0), (2051.00, 18140.0), (2052.00, 18380.0), (2053.00, 18618.0), (2054.00, 18858.0), (2055.00, 19094.0), (2056.00, 19326.0), (2057.00, 19554.0), (2058.00, 19777.0), (2059.00, 19996.0), (2060.00, 20205.0), (2061.00, 20412.0)

UNITS: persons/year

NIM_Arrivals_H[F, A14] = GRAPH(TIME)

Points: (2011.00, 8260.0), (2012.00, 7330.0), (2013.00, 8100.0), (2014.00, 7400.0), (2015.00, 7910.0), (2016.00, 8760.0), (2017.00, 8770.0), (2018.00, 8314.0), (2019.00, 8524.0), (2020.00, 8721.0), (2021.00, 8840.0), (2022.00, 9033.0), (2023.00, 9224.0), (2024.00, 9424.0), (2025.00, 9635.0), (2026.00, 9882.0), (2027.00, 10094.0), (2028.00, 10297.0), (2029.00, 10498.0), (2030.00, 10694.0), (2031.00, 10883.0), (2032.00, 11064.0), (2033.00, 11237.0), (2034.00, 11400.0), (2035.00, 11557.0), (2036.00, 11705.0), (2037.00, 11844.0), (2038.00, 11974.0), (2039.00, 12093.0), (2040.00, 12203.0), (2041.00, 12311.0), (2042.00, 12414.0), (2043.00, 12521.0), (2044.00, 12634.0), (2045.00, 12752.0), (2046.00, 12875.0), (2047.00, 13005.0), (2048.00, 13144.0), (2049.00, 13291.0), (2050.00, 13445.0), (2051.00, 13607.0), (2052.00, 13775.0), (2053.00, 13949.0), (2054.00, 14126.0), (2055.00, 14308.0), (2056.00, 14491.0), (2057.00, 14677.0), (2058.00, 14862.0), (2059.00, 15046.0), (2060.00, 15229.0), (2061.00, 15408.0)

UNITS: persons/year

NIM_Arrivals_H[F, A19] = GRAPH(TIME)

Points: (2011.00, 12730.0), (2012.00, 11850.0), (2013.00, 12750.0), (2014.00, 11510.0), (2015.00, 11560.0), (2016.00, 11910.0), (2017.00, 11990.0), (2018.00, 12291.0), (2019.00, 12382.0), (2020.00, 12483.0), (2021.00, 12646.0), (2022.00, 12868.0), (2023.00, 13150.0), (2024.00, 13495.0), (2025.00, 13815.0), (2026.00, 14010.0), (2027.00, 14287.0), (2028.00, 14537.0), (2029.00, 14754.0), (2030.00, 15018.0), (2031.00, 15425.0), (2032.00, 15742.0), (2033.00, 16031.0), (2034.00, 16322.0), (2035.00, 16606.0), (2036.00, 16884.0), (2037.00, 17152.0), (2038.00, 17408.0), (2039.00, 17649.0), (2040.00, 17878.0), (2041.00, 18096.0), (2042.00, 18302.0), (2043.00, 18495.0), (2044.00, 18675.0), (2045.00, 18843.0), (2046.00, 18995.0), (2047.00, 19140.0), (2048.00, 19288.0), (2049.00, 19440.0), (2050.00, 19599.0), (2051.00, 19767.0), (2052.00, 19945.0), (2053.00, 20135.0), (2054.00, 20336.0), (2055.00, 20546.0), (2056.00, 20770.0), (2057.00, 21003.0), (2058.00, 21244.0), (2059.00, 21494.0), (2060.00, 21749.0), (2061.00, 22007.0)

UNITS: persons/year

NIM_Arrivals_H[F, A24] = GRAPH(TIME)

Points: (2011.00, 24200.0), (2012.00, 25110.0), (2013.00, 25430.0), (2014.00, 23760.0), (2015.00, 23010.0), (2016.00, 24220.0), (2017.00, 24350.0), (2018.00, 26713.0), (2019.00, 26944.0), (2020.00, 27088.0), (2021.00, 27289.0), (2022.00, 27437.0), (2023.00, 27636.0), (2024.00, 27828.0), (2025.00, 28099.0), (2026.00, 28537.0), (2027.00, 29051.0), (2028.00, 29667.0), (2029.00, 30299.0), (2030.00, 30891.0), (2031.00, 31271.0), (2032.00, 31798.0), (2033.00, 32309.0), (2034.00, 32860.0), (2035.00, 33458.0), (2036.00, 34196.0), (2037.00, 34820.0), (2038.00, 35408.0), (2039.00, 35993.0), (2040.00, 36567.0), (2041.00, 37123.0), (2042.00, 37655.0), (2043.00, 38161.0), (2044.00, 38644.0), (2045.00, 39101.0), (2046.00, 39536.0), (2047.00, 39946.0), (2048.00, 40327.0), (2049.00, 40680.0), (2050.00, 41008.0), (2051.00, 41315.0), (2052.00, 41613.0), (2053.00, 41918.0), (2054.00, 42235.0), (2055.00, 42569.0), (2056.00, 42919.0), (2057.00, 43292.0), (2058.00, 43687.0), (2059.00, 44102.0), (2060.00, 44543.0), (2061.00, 45003.0)

UNITS: persons/year

NIM_Arrivals_H[F, A29] = GRAPH(TIME)

Points: (2011.00, 25540.0), (2012.00, 26140.0), (2013.00, 27210.0), (2014.00, 25850.0), (2015.00, 26540.0), (2016.00, 28290.0), (2017.00, 28450.0), (2018.00, 28399.0), (2019.00, 28633.0), (2020.00, 28860.0), (2021.00, 29051.0), (2022.00, 29322.0), (2023.00, 29539.0), (2024.00, 29770.0), (2025.00, 29930.0), (2026.00, 30130.0), (2027.00, 30270.0), (2028.00, 30464.0), (2029.00, 30678.0), (2030.00, 30988.0), (2031.00, 31484.0), (2032.00, 32013.0), (2033.00, 32622.0), (2034.00, 33222.0), (2035.00, 33787.0), (2036.00, 34141.0), (2037.00, 34669.0), (2038.00, 35218.0), (2039.00, 35794.0), (2040.00, 36395.0), (2041.00, 37102.0), (2042.00, 37716.0), (2043.00, 38303.0), (2044.00, 38884.0), (2045.00, 39450.0), (2046.00, 39998.0), (2047.00, 40521.0), (2048.00, 41020.0), (2049.00, 41494.0), (2050.00, 41945.0), (2051.00, 42376.0), (2052.00, 42779.0), (2053.00, 43150.0), (2054.00, 43494.0), (2055.00, 43817.0), (2056.00, 44123.0), (2057.00, 44425.0), (2058.00, 44734.0), (2059.00, 45055.0), (2060.00, 45394.0), (2061.00, 45751.0)

UNITS: persons/year

NIM_Arrivals_H[F, A34] = GRAPH(TIME)

Points: (2011.00, 18390.0), (2012.00, 17710.0), (2013.00, 18900.0), (2014.00, 18820.0), (2015.00, 20650.0), (2016.00, 22200.0), (2017.00, 22350.0), (2018.00, 22078.0), (2019.00, 22473.0), (2020.00, 22882.0), (2021.00, 23246.0), (2022.00, 23520.0), (2023.00, 23751.0), (2024.00, 23932.0), (2025.00, 24108.0), (2026.00, 24251.0), (2027.00, 24455.0), (2028.00, 24617.0), (2029.00, 24789.0), (2030.00, 24910.0), (2031.00, 25059.0), (2032.00, 25164.0), (2033.00, 25310.0), (2034.00, 25474.0), (2035.00, 25708.0), (2036.00, 26084.0), (2037.00, 26482.0), (2038.00, 26941.0), (2039.00, 27394.0), (2040.00, 27817.0), (2041.00, 28080.0), (2042.00, 28480.0), (2043.00, 28893.0), (2044.00, 29327.0), (2045.00, 29780.0), (2046.00, 30308.0), (2047.00, 30771.0), (2048.00, 31213.0), (2049.00, 31649.0), (2050.00, 32075.0), (2051.00, 32488.0), (2052.00, 32882.0), (2053.00, 33257.0), (2054.00, 33614.0), (2055.00, 33952.0), (2056.00, 34277.0), (2057.00, 34581.0), (2058.00, 34860.0), (2059.00, 35120.0), (2060.00, 35363.0), (2061.00, 35595.0)

UNITS: persons/year

NIM_Arrivals_H[F, A39] = GRAPH(TIME)

Points: (2011.00, 14300.0), (2012.00, 12580.0), (2013.00, 13400.0), (2014.00, 12800.0), (2015.00, 13890.0), (2016.00, 15350.0), (2017.00, 15660.0), (2018.00, 15050.0), (2019.00, 15588.0), (2020.00, 16103.0), (2021.00, 16504.0), (2022.00, 16835.0), (2023.00, 17104.0), (2024.00, 17393.0), (2025.00, 17692.0), (2026.00, 17952.0), (2027.00, 18145.0), (2028.00, 18310.0), (2029.00, 18441.0), (2030.00, 18568.0), (2031.00, 18670.0), (2032.00, 18817.0), (2033.00, 18931.0), (2034.00, 19056.0), (2035.00, 19146.0), (2036.00, 19250.0), (2037.00, 19323.0), (2038.00, 19430.0), (2039.00, 19549.0), (2040.00, 19720.0), (2041.00, 19993.0), (2042.00, 20282.0), (2043.00, 20611.0), (2044.00, 20935.0), (2045.00, 21240.0), (2046.00, 21429.0), (2047.00, 21718.0), (2048.00, 22017.0), (2049.00, 22330.0), (2050.00, 22656.0), (2051.00, 23037.0), (2052.00, 23372.0), (2053.00, 23690.0), (2054.00, 24005.0), (2055.00, 24313.0), (2056.00, 24611.0), (2057.00, 24895.0), (2058.00, 25164.0), (2059.00, 25422.0), (2060.00, 25667.0), (2061.00, 25900.0)

UNITS: persons/year

NIM_Arrivals_H[F, A44] = GRAPH(TIME)

Points: (2011.00, 10350.0), (2012.00, 9440.0), (2013.00, 10600.0), (2014.00, 9630.0), (2015.00, 10240.0), (2016.00, 10980.0), (2017.00, 11120.0), (2018.00, 9876.0), (2019.00, 9980.0), (2020.00, 10172.0), (2021.00, 10475.0), (2022.00, 10822.0), (2023.00, 11228.0), (2024.00, 11610.0), (2025.00, 11979.0), (2026.00, 12265.0), (2027.00, 12501.0), (2028.00, 12693.0), (2029.00, 12897.0), (2030.00, 13108.0), (2031.00, 13293.0), (2032.00, 13433.0), (2033.00, 13553.0), (2034.00, 13646.0), (2035.00, 13735.0), (2036.00, 13810.0), (2037.00, 13916.0), (2038.00, 13999.0), (2039.00, 14085.0), (2040.00, 14150.0), (2041.00, 14225.0), (2042.00, 14278.0), (2043.00, 14353.0), (2044.00, 14438.0), (2045.00, 14558.0), (2046.00, 14751.0), (2047.00, 14954.0), (2048.00, 15189.0), (2049.00, 15420.0), (2050.00, 15636.0), (2051.00, 15772.0), (2052.00, 15974.0), (2053.00, 16187.0), (2054.00, 16408.0), (2055.00, 16641.0), (2056.00, 16912.0), (2057.00, 17150.0), (2058.00, 17377.0), (2059.00, 17600.0), (2060.00, 17819.0), (2061.00, 18032.0)

UNITS: persons/year

NIM_Arrivals_H[F, A49] = GRAPH(TIME)

Points: (2011.00, 7700.0), (2012.00, 7240.0), (2013.00, 7690.0), (2014.00, 7170.0), (2015.00, 7610.0), (2016.00, 8620.0), (2017.00, 9070.0), (2018.00, 8502.0), (2019.00, 8545.0), (2020.00, 8545.0), (2021.00, 8435.0), (2022.00, 8357.0), (2023.00, 8358.0), (2024.00, 8439.0), (2025.00, 8590.0), (2026.00, 8830.0), (2027.00, 9112.0), (2028.00, 9441.0), (2029.00, 9755.0), (2030.00, 10060.0), (2031.00, 10297.0), (2032.00, 10497.0), (2033.00, 10656.0), (2034.00, 10826.0), (2035.00, 10996.0), (2036.00, 11149.0), (2037.00, 11269.0), (2038.00, 11372.0), (2039.00, 11453.0), (2040.00, 11524.0), (2041.00, 11585.0), (2042.00, 11673.0), (2043.00, 11742.0), (2044.00, 11818.0), (2045.00, 11869.0), (2046.00, 11932.0), (2047.00, 11977.0), (2048.00, 12041.0), (2049.00, 12109.0), (2050.00, 12205.0), (2051.00, 12359.0), (2052.00, 12526.0), (2053.00, 12719.0), (2054.00, 12909.0), (2055.00, 13091.0), (2056.00, 13204.0), (2057.00, 13371.0), (2058.00, 13542.0), (2059.00, 13725.0), (2060.00, 13912.0), (2061.00, 14137.0)

UNITS: persons/year

NIM_Arrivals_H[F, A54] = GRAPH(TIME)

Points: (2011.00, 7140.0), (2012.00, 7040.0), (2013.00, 7400.0), (2014.00, 6650.0), (2015.00, 6810.0), (2016.00, 7850.0), (2017.00, 8200.0), (2018.00, 7447.0), (2019.00, 7535.0), (2020.00, 7688.0), (2021.00, 7956.0), (2022.00, 8204.0), (2023.00, 8363.0), (2024.00, 8414.0), (2025.00, 8421.0), (2026.00, 8313.0), (2027.00, 8236.0), (2028.00, 8235.0), (2029.00, 8309.0), (2030.00, 8454.0), (2031.00, 8683.0), (2032.00, 8954.0), (2033.00, 9272.0), (2034.00, 9579.0), (2035.00, 9877.0), (2036.00, 10113.0), (2037.00, 10313.0), (2038.00, 10468.0), (2039.00, 10634.0), (2040.00, 10799.0), (2041.00, 10952.0), (2042.00, 11076.0), (2043.00, 11179.0), (2044.00, 11259.0), (2045.00, 11329.0), (2046.00, 11391.0), (2047.00, 11479.0), (2048.00, 11549.0), (2049.00, 11624.0), (2050.00, 11676.0), (2051.00, 11737.0), (2052.00, 11785.0), (2053.00, 11848.0), (2054.00, 11916.0), (2055.00, 12009.0), (2056.00, 12156.0), (2057.00, 12320.0), (2058.00, 12509.0), (2059.00, 12698.0), (2060.00, 12876.0), (2061.00, 12991.0)

UNITS: persons/year

NIM_Arrivals_H[F, A59] = GRAPH(TIME)

Points: (2011.00, 5980.0), (2012.00, 6080.0), (2013.00, 6410.0), (2014.00, 5930.0), (2015.00, 6380.0), (2016.00, 7380.0), (2017.00, 8040.0), (2018.00, 7280.0), (2019.00, 7354.0), (2020.00, 7370.0), (2021.00, 7330.0), (2022.00, 7297.0), (2023.00, 7302.0), (2024.00, 7391.0), (2025.00, 7543.0), (2026.00, 7807.0), (2027.00, 8051.0), (2028.00, 8204.0), (2029.00, 8254.0), (2030.00, 8256.0), (2031.00, 8157.0), (2032.00, 8087.0), (2033.00, 8091.0), (2034.00, 8167.0), (2035.00, 8311.0), (2036.00, 8538.0), (2037.00, 8803.0), (2038.00, 9116.0), (2039.00, 9416.0), (2040.00, 9706.0), (2041.00, 9938.0), (2042.00, 10130.0), (2043.00, 10284.0), (2044.00, 10446.0), (2045.00, 10611.0), (2046.00, 10763.0), (2047.00, 10884.0), (2048.00, 10986.0), (2049.00, 11064.0), (2050.00, 11135.0), (2051.00, 11198.0), (2052.00, 11286.0), (2053.00, 11357.0), (2054.00, 11431.0), (2055.00, 11484.0), (2056.00, 11547.0), (2057.00, 11595.0), (2058.00, 11659.0), (2059.00, 11727.0), (2060.00, 11822.0), (2061.00, 11968.0)

UNITS: persons/year

NIM_Arrivals_H[F, A64] = GRAPH(TIME)

Points: (2011.00, 4730.0), (2012.00, 4720.0), (2013.00, 5310.0), (2014.00, 4960.0), (2015.00, 5210.0), (2016.00, 6270.0), (2017.00, 6430.0), (2018.00, 5651.0), (2019.00, 5793.0), (2020.00, 5922.0), (2021.00, 6072.0), (2022.00, 6204.0), (2023.00, 6307.0), (2024.00, 6374.0), (2025.00, 6390.0), (2026.00, 6356.0), (2027.00, 6332.0), (2028.00, 6340.0), (2029.00, 6421.0), (2030.00, 6554.0), (2031.00, 6788.0), (2032.00, 6996.0), (2033.00, 7127.0), (2034.00, 7169.0), (2035.00, 7173.0), (2036.00, 7090.0), (2037.00, 7033.0), (2038.00, 7038.0), (2039.00, 7106.0), (2040.00, 7233.0), (2041.00, 7430.0), (2042.00, 7660.0), (2043.00, 7933.0), (2044.00, 8191.0), (2045.00, 8442.0), (2046.00, 8643.0), (2047.00, 8810.0), (2048.00, 8944.0), (2049.00, 9086.0), (2050.00, 9231.0), (2051.00, 9361.0), (2052.00, 9467.0), (2053.00, 9555.0), (2054.00, 9625.0), (2055.00, 9689.0), (2056.00, 9745.0), (2057.00, 9823.0), (2058.00, 9886.0), (2059.00, 9951.0), (2060.00, 9997.0), (2061.00, 10054.0)

UNITS: persons/year

NIM_Arrivals_H[F, A69] = GRAPH(TIME)

Points: (2011.00, 2820.0), (2012.00, 3070.0), (2013.00, 3560.0), (2014.00, 3510.0), (2015.00, 3910.0), (2016.00, 4390.0), (2017.00, 4650.0), (2018.00, 3947.0), (2019.00, 4039.0), (2020.00, 4136.0), (2021.00, 4237.0), (2022.00, 4343.0), (2023.00, 4448.0), (2024.00, 4563.0), (2025.00, 4669.0), (2026.00, 4792.0), (2027.00, 4898.0), (2028.00, 4982.0), (2029.00, 5036.0), (2030.00, 5049.0), (2031.00, 5028.0), (2032.00, 5013.0), (2033.00, 5024.0), (2034.00, 5096.0), (2035.00, 5204.0), (2036.00, 5395.0), (2037.00, 5560.0), (2038.00, 5658.0), (2039.00, 5690.0), (2040.00, 5692.0), (2041.00, 5629.0), (2042.00, 5588.0), (2043.00, 5597.0), (2044.00, 5655.0), (2045.00, 5759.0), (2046.00, 5920.0), (2047.00, 6107.0), (2048.00, 6324.0), (2049.00, 6530.0), (2050.00, 6729.0), (2051.00, 6890.0), (2052.00, 7022.0), (2053.00, 7131.0), (2054.00, 7248.0), (2055.00, 7365.0), (2056.00, 7472.0), (2057.00, 7557.0), (2058.00, 7629.0), (2059.00, 7687.0), (2060.00, 7741.0), (2061.00, 7786.0)

UNITS: persons/year

NIM_Arrivals_H[F, A74] = GRAPH(TIME)

Points: (2011.00, 1540.0), (2012.00, 1690.0), (2013.00, 2000.0), (2014.00, 1840.0), (2015.00, 2210.0), (2016.00, 2550.0), (2017.00, 2830.0), (2018.00, 2577.0), (2019.00, 2670.0), (2020.00, 2760.0), (2021.00, 2854.0), (2022.00, 2871.0), (2023.00, 2933.0), (2024.00, 3003.0), (2025.00, 3078.0), (2026.00, 3155.0), (2027.00, 3239.0), (2028.00, 3320.0), (2029.00, 3409.0), (2030.00, 3491.0), (2031.00, 3588.0), (2032.00, 3671.0), (2033.00, 3736.0), (2034.00, 3778.0), (2035.00, 3791.0), (2036.00, 3776.0), (2037.00, 3766.0), (2038.00, 3774.0), (2039.00, 3829.0), (2040.00, 3911.0), (2041.00, 4054.0), (2042.00, 4178.0), (2043.00, 4254.0), (2044.00, 4278.0), (2045.00, 4285.0), (2046.00, 4240.0), (2047.00, 4209.0), (2048.00, 4214.0), (2049.00, 4257.0), (2050.00, 4334.0), (2051.00, 4455.0), (2052.00, 4592.0), (2053.00, 4755.0), (2054.00, 4909.0), (2055.00, 5061.0), (2056.00, 5177.0), (2057.00, 5278.0), (2058.00, 5358.0), (2059.00, 5447.0), (2060.00, 5536.0), (2061.00, 5616.0)

UNITS: persons/year

NIM_Arrivals_H[F, A79] = GRAPH(TIME)

Points: (2011.00, 1071.0), (2012.00, 1086.0), (2013.00, 1235.0), (2014.00, 1130.0), (2015.00, 1140.0), (2016.00, 1184.0), (2017.00, 1275.0), (2018.00, 1571.0), (2019.00, 1656.0), (2020.00, 1748.0), (2021.00, 1840.0), (2022.00, 1986.0), (2023.00, 2106.0), (2024.00, 2185.0), (2025.00, 2259.0), (2026.00, 2344.0), (2027.00, 2360.0), (2028.00, 2412.0), (2029.00, 2476.0), (2030.00, 2541.0), (2031.00, 2608.0), (2032.00, 2682.0), (2033.00, 2753.0), (2034.00, 2831.0), (2035.00, 2904.0), (2036.00, 2985.0), (2037.00, 3058.0), (2038.00, 3116.0), (2039.00, 3155.0), (2040.00, 3171.0), (2041.00, 3159.0), (2042.00, 3155.0), (2043.00, 3163.0), (2044.00, 3207.0), (2045.00, 3278.0), (2046.00, 3397.0), (2047.00, 3507.0), (2048.00, 3576.0), (2049.00, 3599.0), (2050.00, 3612.0), (2051.00, 3575.0), (2052.00, 3550.0), (2053.00, 3556.0), (2054.00, 3593.0), (2055.00, 3658.0), (2056.00, 3761.0), (2057.00, 3879.0), (2058.00, 4017.0), (2059.00, 4150.0), (2060.00, 4279.0), (2061.00, 4380.0)

UNITS: persons/year

NIM_Arrivals_H[F, A84] = GRAPH(TIME)

Points: (2011.00, 911.0), (2012.00, 924.0), (2013.00, 1050.0), (2014.00, 961.0), (2015.00, 970.0), (2016.00, 1007.0), (2017.00, 1084.0), (2018.00, 1144.0), (2019.00, 1186.0), (2020.00, 1229.0), (2021.00, 1275.0), (2022.00, 1334.0), (2023.00, 1391.0), (2024.00, 1474.0), (2025.00, 1557.0), (2026.00, 1645.0), (2027.00, 1783.0), (2028.00, 1890.0), (2029.00, 1975.0), (2030.00, 2052.0), (2031.00, 2129.0), (2032.00, 2149.0), (2033.00, 2204.0), (2034.00, 2266.0), (2035.00, 2331.0), (2036.00, 2398.0), (2037.00, 2469.0), (2038.00, 2541.0), (2039.00, 2616.0), (2040.00, 2688.0), (2041.00, 2769.0), (2042.00, 2840.0), (2043.00, 2902.0), (2044.00, 2945.0), (2045.00, 2964.0), (2046.00, 2962.0), (2047.00, 2958.0), (2048.00, 2971.0), (2049.00, 3014.0), (2050.00, 3081.0), (2051.00, 3200.0), (2052.00, 3306.0), (2053.00, 3378.0), (2054.00, 3413.0), (2055.00, 3427.0), (2056.00, 3395.0), (2057.00, 3373.0), (2058.00, 3381.0), (2059.00, 3419.0), (2060.00, 3486.0), (2061.00, 3586.0)

UNITS: persons/year

NIM_Arrivals_H[F, A89] = GRAPH(TIME)

Points: (2011.00, 623.0), (2012.00, 631.0), (2013.00, 718.0), (2014.00, 657.0), (2015.00, 663.0), (2016.00, 688.0), (2017.00, 741.0), (2018.00, 831.0), (2019.00, 836.0), (2020.00, 846.0), (2021.00, 863.0), (2022.00, 890.0), (2023.00, 921.0), (2024.00, 958.0), (2025.00, 999.0), (2026.00, 1041.0), (2027.00, 1094.0), (2028.00, 1147.0), (2029.00, 1222.0), (2030.00, 1298.0), (2031.00, 1378.0), (2032.00, 1505.0), (2033.00, 1604.0), (2034.00, 1678.0), (2035.00, 1751.0), (2036.00, 1816.0), (2037.00, 1845.0), (2038.00, 1902.0), (2039.00, 1963.0), (2040.00, 2028.0), (2041.00, 2097.0), (2042.00, 2166.0), (2043.00, 2239.0), (2044.00, 2313.0), (2045.00, 2384.0), (2046.00, 2465.0), (2047.00, 2536.0), (2048.00, 2598.0), (2049.00, 2641.0), (2050.00, 2663.0), (2051.00, 2667.0), (2052.00, 2672.0), (2053.00, 2692.0), (2054.00, 2747.0), (2055.00, 2819.0), (2056.00, 2940.0), (2057.00, 3046.0), (2058.00, 3115.0), (2059.00, 3152.0), (2060.00, 3163.0), (2061.00, 3142.0)

UNITS: persons/year

NIM_Arrivals_H[F, A94] = GRAPH(TIME)

Points: (2011.00, 263.0), (2012.00, 267.0), (2013.00, 304.0), (2014.00, 278.0), (2015.00, 280.0), (2016.00, 291.0), (2017.00, 314.0), (2018.00, 414.0), (2019.00, 421.0), (2020.00, 431.0), (2021.00, 438.0), (2022.00, 442.0), (2023.00, 445.0), (2024.00, 450.0), (2025.00, 458.0), (2026.00, 472.0), (2027.00, 491.0), (2028.00, 509.0), (2029.00, 534.0), (2030.00, 560.0), (2031.00, 585.0), (2032.00, 621.0), (2033.00, 657.0), (2034.00, 706.0), (2035.00, 761.0), (2036.00, 814.0), (2037.00, 901.0), (2038.00, 968.0), (2039.00, 1015.0), (2040.00, 1068.0), (2041.00, 1120.0), (2042.00, 1148.0), (2043.00, 1196.0), (2044.00, 1245.0), (2045.00, 1296.0), (2046.00, 1351.0), (2047.00, 1408.0), (2048.00, 1467.0), (2049.00, 1527.0), (2050.00, 1588.0), (2051.00, 1653.0), (2052.00, 1712.0), (2053.00, 1763.0), (2054.00, 1806.0), (2055.00, 1832.0), (2056.00, 1847.0), (2057.00, 1865.0), (2058.00, 1895.0), (2059.00, 1947.0), (2060.00, 2011.0), (2061.00, 2109.0)

UNITS: persons/year

NIM_Arrivals_H[F, A99] = GRAPH(TIME)

Points: (2011.00, 69.0), (2012.00, 70.0), (2013.00, 79.0), (2014.00, 73.0), (2015.00, 73.0), (2016.00, 76.0), (2017.00, 82.0), (2018.00, 115.0), (2019.00, 122.0), (2020.00, 127.0), (2021.00, 131.0), (2022.00, 134.0), (2023.00, 139.0), (2024.00, 141.0), (2025.00, 145.0), (2026.00, 148.0), (2027.00, 150.0), (2028.00, 151.0), (2029.00, 154.0), (2030.00, 158.0), (2031.00, 165.0), (2032.00, 173.0), (2033.00, 182.0), (2034.00, 194.0), (2035.00, 208.0), (2036.00, 223.0), (2037.00, 243.0), (2038.00, 261.0), (2039.00, 288.0), (2040.00, 316.0), (2041.00, 345.0), (2042.00, 392.0), (2043.00, 427.0), (2044.00, 456.0), (2045.00, 488.0), (2046.00, 519.0), (2047.00, 543.0), (2048.00, 574.0), (2049.00, 608.0), (2050.00, 644.0), (2051.00, 683.0), (2052.00, 723.0), (2053.00, 764.0), (2054.00, 807.0), (2055.00, 851.0), (2056.00, 899.0), (2057.00, 945.0), (2058.00, 986.0), (2059.00, 1022.0), (2060.00, 1049.0), (2061.00, 1071.0)

UNITS: persons/year

NIM_Arrivals_H[F, A104] = GRAPH(TIME)

Points: (2011.00, 9.0), (2012.00, 9.0), (2013.00, 11.0), (2014.00, 10.0), (2015.00, 10.0), (2016.00, 10.0), (2017.00, 11.0), (2018.00, 21.0), (2019.00, 22.0), (2020.00, 24.0), (2021.00, 27.0), (2022.00, 29.0), (2023.00, 31.0), (2024.00, 33.0), (2025.00, 35.0), (2026.00, 36.0), (2027.00, 38.0), (2028.00, 39.0), (2029.00, 40.0), (2030.00, 42.0), (2031.00, 43.0), (2032.00, 44.0), (2033.00, 45.0), (2034.00, 47.0), (2035.00, 49.0), (2036.00, 53.0), (2037.00, 57.0), (2038.00, 61.0), (2039.00, 67.0), (2040.00, 73.0), (2041.00, 81.0), (2042.00, 90.0), (2043.00, 99.0), (2044.00, 112.0), (2045.00, 125.0), (2046.00, 140.0), (2047.00, 162.0), (2048.00, 180.0), (2049.00, 198.0), (2050.00, 216.0), (2051.00, 236.0), (2052.00, 256.0), (2053.00, 279.0), (2054.00, 302.0), (2055.00, 328.0), (2056.00, 355.0), (2057.00, 383.0), (2058.00, 413.0), (2059.00, 445.0), (2060.00, 478.0), (2061.00, 515.0)

UNITS: persons/year

NIM_Arrivals_L[M, A4] = GRAPH(TIME)

Points: (2011.00, 14810.0), (2012.00, 13430.0), (2013.00, 14270.0), (2014.00, 13530.0), (2015.00, 14830.0), (2016.00, 16080.0), (2017.00, 16070.0), (2018.00, 14453.0), (2019.00, 14509.0), (2020.00, 14544.0), (2021.00, 14553.0), (2022.00, 14541.0), (2023.00, 14507.0), (2024.00, 14454.0), (2025.00, 14381.0), (2026.00, 14294.0), (2027.00, 14208.0), (2028.00, 14143.0), (2029.00, 14115.0), (2030.00, 14125.0), (2031.00, 14171.0), (2032.00, 14237.0), (2033.00, 14307.0), (2034.00, 14385.0), (2035.00, 14472.0), (2036.00, 14567.0), (2037.00, 14670.0), (2038.00, 14781.0), (2039.00, 14900.0), (2040.00, 15023.0), (2041.00, 15151.0), (2042.00, 15279.0), (2043.00, 15403.0), (2044.00, 15524.0), (2045.00, 15635.0), (2046.00, 15741.0), (2047.00, 15834.0), (2048.00, 15916.0), (2049.00, 15987.0), (2050.00, 16045.0), (2051.00, 16091.0), (2052.00, 16128.0), (2053.00, 16153.0), (2054.00, 16173.0), (2055.00, 16185.0), (2056.00, 16192.0), (2057.00, 16197.0), (2058.00, 16203.0), (2059.00, 16209.0), (2060.00, 16218.0), (2061.00, 16232.0)

UNITS: persons/year

NIM_Arrivals_L[M, A9] = GRAPH(TIME)

Points: (2011.00, 10830.0), (2012.00, 9840.0), (2013.00, 10930.0), (2014.00, 10360.0), (2015.00, 11460.0), (2016.00, 12550.0), (2017.00, 12610.0), (2018.00, 11574.0), (2019.00, 11705.0), (2020.00, 11808.0), (2021.00, 11925.0), (2022.00, 11961.0), (2023.00, 12008.0), (2024.00, 12045.0), (2025.00, 12066.0), (2026.00, 12072.0), (2027.00, 12060.0), (2028.00, 12029.0), (2029.00, 11985.0), (2030.00, 11926.0), (2031.00, 11856.0), (2032.00, 11794.0), (2033.00, 11756.0), (2034.00, 11742.0), (2035.00, 11755.0), (2036.00, 11793.0), (2037.00, 11849.0), (2038.00, 11907.0), (2039.00, 11971.0), (2040.00, 12041.0), (2041.00, 12121.0), (2042.00, 12205.0), (2043.00, 12296.0), (2044.00, 12393.0), (2045.00, 12492.0), (2046.00, 12593.0), (2047.00, 12695.0), (2048.00, 12797.0), (2049.00, 12890.0), (2050.00, 12981.0), (2051.00, 13064.0), (2052.00, 13136.0), (2053.00, 13201.0), (2054.00, 13257.0), (2055.00, 13301.0), (2056.00, 13339.0), (2057.00, 13366.0), (2058.00, 13386.0), (2059.00, 13401.0), (2060.00, 13411.0), (2061.00, 13418.0)

UNITS: persons/year

NIM_Arrivals_L[M, A14] = GRAPH(TIME)

Points: (2011.00, 8420.0), (2012.00, 7360.0), (2013.00, 8220.0), (2014.00, 7640.0), (2015.00, 8350.0), (2016.00, 9020.0), (2017.00, 9010.0), (2018.00, 8311.0), (2019.00, 8491.0), (2020.00, 8653.0), (2021.00, 8740.0), (2022.00, 8896.0), (2023.00, 9009.0), (2024.00, 9103.0), (2025.00, 9177.0), (2026.00, 9266.0), (2027.00, 9293.0), (2028.00, 9329.0), (2029.00, 9355.0), (2030.00, 9373.0), (2031.00, 9376.0), (2032.00, 9368.0), (2033.00, 9346.0), (2034.00, 9313.0), (2035.00, 9269.0), (2036.00, 9218.0), (2037.00, 9170.0), (2038.00, 9142.0), (2039.00, 9131.0), (2040.00, 9140.0), (2041.00, 9170.0), (2042.00, 9210.0), (2043.00, 9255.0), (2044.00, 9301.0), (2045.00, 9356.0), (2046.00, 9415.0), (2047.00, 9477.0), (2048.00, 9546.0), (2049.00, 9619.0), (2050.00, 9694.0), (2051.00, 9770.0), (2052.00, 9847.0), (2053.00, 9921.0), (2054.00, 9993.0), (2055.00, 10060.0), (2056.00, 10121.0), (2057.00, 10179.0), (2058.00, 10227.0), (2059.00, 10268.0), (2060.00, 10301.0), (2061.00, 10330.0)

UNITS: persons/year

NIM_Arrivals_L[M, A19] = GRAPH(TIME)

Points: (2011.00, 11990.0), (2012.00, 11040.0), (2013.00, 11660.0), (2014.00, 10790.0), (2015.00, 10870.0), (2016.00, 11300.0), (2017.00, 11040.0), (2018.00, 11236.0), (2019.00, 11305.0), (2020.00, 11383.0), (2021.00, 11489.0), (2022.00, 11691.0), (2023.00, 11941.0), (2024.00, 12219.0), (2025.00, 12461.0), (2026.00, 12593.0), (2027.00, 12799.0), (2028.00, 12954.0), (2029.00, 13065.0), (2030.00, 13186.0), (2031.00, 13368.0), (2032.00, 13380.0), (2033.00, 13427.0), (2034.00, 13466.0), (2035.00, 13494.0), (2036.00, 13507.0), (2037.00, 13502.0), (2038.00, 13482.0), (2039.00, 13444.0), (2040.00, 13389.0), (2041.00, 13321.0), (2042.00, 13252.0), (2043.00, 13193.0), (2044.00, 13147.0), (2045.00, 13127.0), (2046.00, 13152.0), (2047.00, 13202.0), (2048.00, 13256.0), (2049.00, 13315.0), (2050.00, 13380.0), (2051.00, 13451.0), (2052.00, 13529.0), (2053.00, 13614.0), (2054.00, 13705.0), (2055.00, 13802.0), (2056.00, 13902.0), (2057.00, 14003.0), (2058.00, 14103.0), (2059.00, 14201.0), (2060.00, 14293.0), (2061.00, 14379.0)

UNITS: persons/year

NIM_Arrivals_L[M, A24] = GRAPH(TIME)

Points: (2011.00, 23570.0), (2012.00, 23740.0), (2013.00, 24100.0), (2014.00, 24480.0), (2015.00, 21950.0), (2016.00, 22400.0), (2017.00, 22440.0), (2018.00, 24865.0), (2019.00, 24888.0), (2020.00, 24803.0), (2021.00, 24812.0), (2022.00, 24799.0), (2023.00, 24842.0), (2024.00, 24946.0), (2025.00, 25148.0), (2026.00, 25473.0), (2027.00, 25922.0), (2028.00, 26438.0), (2029.00, 26943.0), (2030.00, 27402.0), (2031.00, 27665.0), (2032.00, 28069.0), (2033.00, 28364.0), (2034.00, 28624.0), (2035.00, 28836.0), (2036.00, 29117.0), (2037.00, 29168.0), (2038.00, 29261.0), (2039.00, 29335.0), (2040.00, 29382.0), (2041.00, 29395.0), (2042.00, 29374.0), (2043.00, 29318.0), (2044.00, 29227.0), (2045.00, 29106.0), (2046.00, 28963.0), (2047.00, 28823.0), (2048.00, 28716.0), (2049.00, 28657.0), (2050.00, 28655.0), (2051.00, 28720.0), (2052.00, 28821.0), (2053.00, 28931.0), (2054.00, 29051.0), (2055.00, 29186.0), (2056.00, 29333.0), (2057.00, 29494.0), (2058.00, 29671.0), (2059.00, 29858.0), (2060.00, 30056.0), (2061.00, 30258.0)

UNITS: persons/year

NIM_Arrivals_L[M, A29] = GRAPH(TIME)

Points: (2011.00, 27820.0), (2012.00, 28020.0), (2013.00, 28720.0), (2014.00, 28290.0), (2015.00, 27110.0), (2016.00, 27720.0), (2017.00, 27340.0), (2018.00, 29141.0), (2019.00, 29227.0), (2020.00, 29271.0), (2021.00, 29303.0), (2022.00, 29379.0), (2023.00, 29403.0), (2024.00, 29393.0), (2025.00, 29322.0), (2026.00, 29354.0), (2027.00, 29341.0), (2028.00, 29393.0), (2029.00, 29543.0), (2030.00, 29819.0), (2031.00, 30254.0), (2032.00, 30766.0), (2033.00, 31334.0), (2034.00, 31875.0), (2035.00, 32368.0), (2036.00, 32636.0), (2037.00, 33109.0), (2038.00, 33442.0), (2039.00, 33725.0), (2040.00, 33958.0), (2041.00, 34237.0), (2042.00, 34315.0), (2043.00, 34424.0), (2044.00, 34509.0), (2045.00, 34563.0), (2046.00, 34579.0), (2047.00, 34554.0), (2048.00, 34491.0), (2049.00, 34392.0), (2050.00, 34260.0), (2051.00, 34104.0), (2052.00, 33962.0), (2053.00, 33872.0), (2054.00, 33836.0), (2055.00, 33861.0), (2056.00, 33945.0), (2057.00, 34066.0), (2058.00, 34198.0), (2059.00, 34342.0), (2060.00, 34500.0), (2061.00, 34674.0)

UNITS: persons/year

NIM_Arrivals_L[M, A34] = GRAPH(TIME)

Points: (2011.00, 19850.0), (2012.00, 20200.0), (2013.00, 21510.0), (2014.00, 21440.0), (2015.00, 22020.0), (2016.00, 23380.0), (2017.00, 23290.0), (2018.00, 23832.0), (2019.00, 24142.0), (2020.00, 24467.0), (2021.00, 24645.0), (2022.00, 24750.0), (2023.00, 24843.0), (2024.00, 24894.0), (2025.00, 24926.0), (2026.00, 24958.0), (2027.00, 25017.0), (2028.00, 25034.0), (2029.00, 25028.0), (2030.00, 24977.0), (2031.00, 25004.0), (2032.00, 24994.0), (2033.00, 25037.0), (2034.00, 25164.0), (2035.00, 25389.0), (2036.00, 25745.0), (2037.00, 26158.0), (2038.00, 26610.0), (2039.00, 27040.0), (2040.00, 27433.0), (2041.00, 27651.0), (2042.00, 28027.0), (2043.00, 28301.0), (2044.00, 28528.0), (2045.00, 28714.0), (2046.00, 28936.0), (2047.00, 29000.0), (2048.00, 29089.0), (2049.00, 29159.0), (2050.00, 29205.0), (2051.00, 29219.0), (2052.00, 29202.0), (2053.00, 29154.0), (2054.00, 29074.0), (2055.00, 28972.0), (2056.00, 28849.0), (2057.00, 28736.0), (2058.00, 28668.0), (2059.00, 28644.0), (2060.00, 28666.0), (2061.00, 28738.0)

UNITS: persons/year

NIM_Arrivals_L[M, A39] = GRAPH(TIME)

Points: (2011.00, 15620.0), (2012.00, 14640.0), (2013.00, 15300.0), (2014.00, 14700.0), (2015.00, 15530.0), (2016.00, 16600.0), (2017.00, 17130.0), (2018.00, 17313.0), (2019.00, 17877.0), (2020.00, 18402.0), (2021.00, 18795.0), (2022.00, 19086.0), (2023.00, 19282.0), (2024.00, 19508.0), (2025.00, 19759.0), (2026.00, 19890.0), (2027.00, 19967.0), (2028.00, 20036.0), (2029.00, 20081.0), (2030.00, 20110.0), (2031.00, 20133.0), (2032.00, 20181.0), (2033.00, 20192.0), (2034.00, 20191.0), (2035.00, 20151.0), (2036.00, 20175.0), (2037.00, 20167.0), (2038.00, 20202.0), (2039.00, 20302.0), (2040.00, 20482.0), (2041.00, 20759.0), (2042.00, 21076.0), (2043.00, 21426.0), (2044.00, 21753.0), (2045.00, 22058.0), (2046.00, 22221.0), (2047.00, 22518.0), (2048.00, 22727.0), (2049.00, 22902.0), (2050.00, 23042.0), (2051.00, 23213.0), (2052.00, 23266.0), (2053.00, 23336.0), (2054.00, 23389.0), (2055.00, 23424.0), (2056.00, 23434.0), (2057.00, 23420.0), (2058.00, 23383.0), (2059.00, 23324.0), (2060.00, 23245.0), (2061.00, 23150.0)

UNITS: persons/year

NIM_Arrivals_L[M, A44] = GRAPH(TIME)

Points: (2011.00, 12210.0), (2012.00, 11620.0), (2013.00, 12500.0), (2014.00, 11990.0), (2015.00, 12090.0), (2016.00, 12590.0), (2017.00, 12530.0), (2018.00, 11545.0), (2019.00, 11658.0), (2020.00, 11860.0), (2021.00, 12203.0), (2022.00, 12603.0), (2023.00, 13071.0), (2024.00, 13475.0), (2025.00, 13852.0), (2026.00, 14136.0), (2027.00, 14346.0), (2028.00, 14491.0), (2029.00, 14654.0), (2030.00, 14834.0), (2031.00, 14933.0), (2032.00, 14992.0), (2033.00, 15043.0), (2034.00, 15073.0), (2035.00, 15096.0), (2036.00, 15117.0), (2037.00, 15154.0), (2038.00, 15163.0), (2039.00, 15161.0), (2040.00, 15135.0), (2041.00, 15151.0), (2042.00, 15148.0), (2043.00, 15175.0), (2044.00, 15246.0), (2045.00, 15378.0), (2046.00, 15582.0), (2047.00, 15814.0), (2048.00, 16069.0), (2049.00, 16309.0), (2050.00, 16529.0), (2051.00, 16650.0), (2052.00, 16865.0), (2053.00, 17016.0), (2054.00, 17148.0), (2055.00, 17252.0), (2056.00, 17373.0), (2057.00, 17413.0), (2058.00, 17465.0), (2059.00, 17505.0), (2060.00, 17531.0), (2061.00, 17539.0)

UNITS: persons/year

NIM_Arrivals_L[M, A49] = GRAPH(TIME)

Points: (2011.00, 9080.0), (2012.00, 8390.0), (2013.00, 9000.0), (2014.00, 8670.0), (2015.00, 8840.0), (2016.00, 9870.0), (2017.00, 10210.0), (2018.00, 9486.0), (2019.00, 9532.0), (2020.00, 9516.0), (2021.00, 9391.0), (2022.00, 9298.0), (2023.00, 9286.0), (2024.00, 9366.0), (2025.00, 9516.0), (2026.00, 9780.0), (2027.00, 10088.0), (2028.00, 10452.0), (2029.00, 10770.0), (2030.00, 11074.0), (2031.00, 11303.0), (2032.00, 11474.0), (2033.00, 11591.0), (2034.00, 11723.0), (2035.00, 11861.0), (2036.00, 11941.0), (2037.00, 11992.0), (2038.00, 12037.0), (2039.00, 12065.0), (2040.00, 12079.0), (2041.00, 12095.0), (2042.00, 12129.0), (2043.00, 12139.0), (2044.00, 12140.0), (2045.00, 12119.0), (2046.00, 12132.0), (2047.00, 12131.0), (2048.00, 12152.0), (2049.00, 12209.0), (2050.00, 12310.0), (2051.00, 12464.0), (2052.00, 12644.0), (2053.00, 12847.0), (2054.00, 13039.0), (2055.00, 13215.0), (2056.00, 13313.0), (2057.00, 13483.0), (2058.00, 13603.0), (2059.00, 13708.0), (2060.00, 13791.0), (2061.00, 13895.0)

UNITS: persons/year

NIM_Arrivals_L[M, A54] = GRAPH(TIME)

Points: (2011.00, 7390.0), (2012.00, 7260.0), (2013.00, 7660.0), (2014.00, 7140.0), (2015.00, 7250.0), (2016.00, 7820.0), (2017.00, 8150.0), (2018.00, 7634.0), (2019.00, 7711.0), (2020.00, 7865.0), (2021.00, 8122.0), (2022.00, 8343.0), (2023.00, 8470.0), (2024.00, 8512.0), (2025.00, 8503.0), (2026.00, 8394.0), (2027.00, 8311.0), (2028.00, 8299.0), (2029.00, 8366.0), (2030.00, 8497.0), (2031.00, 8726.0), (2032.00, 8996.0), (2033.00, 9315.0), (2034.00, 9597.0), (2035.00, 9868.0), (2036.00, 10071.0), (2037.00, 10227.0), (2038.00, 10329.0), (2039.00, 10446.0), (2040.00, 10570.0), (2041.00, 10639.0), (2042.00, 10686.0), (2043.00, 10729.0), (2044.00, 10756.0), (2045.00, 10766.0), (2046.00, 10782.0), (2047.00, 10811.0), (2048.00, 10822.0), (2049.00, 10827.0), (2050.00, 10806.0), (2051.00, 10819.0), (2052.00, 10818.0), (2053.00, 10839.0), (2054.00, 10890.0), (2055.00, 10978.0), (2056.00, 11112.0), (2057.00, 11272.0), (2058.00, 11450.0), (2059.00, 11620.0), (2060.00, 11780.0), (2061.00, 11867.0)

UNITS: persons/year

NIM_Arrivals_L[M, A59] = GRAPH(TIME)

Points: (2011.00, 6140.0), (2012.00, 6060.0), (2013.00, 6410.0), (2014.00, 6010.0), (2015.00, 6210.0), (2016.00, 6950.0), (2017.00, 7570.0), (2018.00, 7315.0), (2019.00, 7377.0), (2020.00, 7386.0), (2021.00, 7334.0), (2022.00, 7289.0), (2023.00, 7290.0), (2024.00, 7365.0), (2025.00, 7514.0), (2026.00, 7760.0), (2027.00, 7976.0), (2028.00, 8101.0), (2029.00, 8142.0), (2030.00, 8132.0), (2031.00, 8030.0), (2032.00, 7955.0), (2033.00, 7945.0), (2034.00, 8010.0), (2035.00, 8138.0), (2036.00, 8355.0), (2037.00, 8615.0), (2038.00, 8920.0), (2039.00, 9192.0), (2040.00, 9449.0), (2041.00, 9644.0), (2042.00, 9793.0), (2043.00, 9894.0), (2044.00, 10006.0), (2045.00, 10126.0), (2046.00, 10198.0), (2047.00, 10245.0), (2048.00, 10287.0), (2049.00, 10314.0), (2050.00, 10325.0), (2051.00, 10342.0), (2052.00, 10375.0), (2053.00, 10387.0), (2054.00, 10391.0), (2055.00, 10376.0), (2056.00, 10390.0), (2057.00, 10392.0), (2058.00, 10412.0), (2059.00, 10462.0), (2060.00, 10548.0), (2061.00, 10679.0)

UNITS: persons/year

NIM_Arrivals_L[M, A64] = GRAPH(TIME)

Points: (2011.00, 5140.0), (2012.00, 5040.0), (2013.00, 5480.0), (2014.00, 5080.0), (2015.00, 5400.0), (2016.00, 6100.0), (2017.00, 6370.0), (2018.00, 5853.0), (2019.00, 5987.0), (2020.00, 6106.0), (2021.00, 6243.0), (2022.00, 6372.0), (2023.00, 6470.0), (2024.00, 6526.0), (2025.00, 6534.0), (2026.00, 6492.0), (2027.00, 6459.0), (2028.00, 6468.0), (2029.00, 6545.0), (2030.00, 6680.0), (2031.00, 6903.0), (2032.00, 7092.0), (2033.00, 7199.0), (2034.00, 7232.0), (2035.00, 7224.0), (2036.00, 7138.0), (2037.00, 7075.0), (2038.00, 7074.0), (2039.00, 7138.0), (2040.00, 7256.0), (2041.00, 7458.0), (2042.00, 7690.0), (2043.00, 7965.0), (2044.00, 8204.0), (2045.00, 8431.0), (2046.00, 8605.0), (2047.00, 8737.0), (2048.00, 8828.0), (2049.00, 8932.0), (2050.00, 9042.0), (2051.00, 9103.0), (2052.00, 9145.0), (2053.00, 9183.0), (2054.00, 9210.0), (2055.00, 9224.0), (2056.00, 9242.0), (2057.00, 9270.0), (2058.00, 9283.0), (2059.00, 9288.0), (2060.00, 9279.0), (2061.00, 9292.0)

UNITS: persons/year

NIM_Arrivals_L[M, A69] = GRAPH(TIME)

Points: (2011.00, 3110.0), (2012.00, 3470.0), (2013.00, 4010.0), (2014.00, 3820.0), (2015.00, 4290.0), (2016.00, 4930.0), (2017.00, 4950.0), (2018.00, 4311.0), (2019.00, 4379.0), (2020.00, 4457.0), (2021.00, 4558.0), (2022.00, 4653.0), (2023.00, 4749.0), (2024.00, 4862.0), (2025.00, 4963.0), (2026.00, 5080.0), (2027.00, 5187.0), (2028.00, 5271.0), (2029.00, 5317.0), (2030.00, 5321.0), (2031.00, 5288.0), (2032.00, 5261.0), (2033.00, 5275.0), (2034.00, 5345.0), (2035.00, 5461.0), (2036.00, 5646.0), (2037.00, 5803.0), (2038.00, 5886.0), (2039.00, 5909.0), (2040.00, 5895.0), (2041.00, 5827.0), (2042.00, 5782.0), (2043.00, 5782.0), (2044.00, 5839.0), (2045.00, 5940.0), (2046.00, 6110.0), (2047.00, 6306.0), (2048.00, 6532.0), (2049.00, 6728.0), (2050.00, 6912.0), (2051.00, 7052.0), (2052.00, 7159.0), (2053.00, 7235.0), (2054.00, 7318.0), (2055.00, 7410.0), (2056.00, 7466.0), (2057.00, 7497.0), (2058.00, 7528.0), (2059.00, 7551.0), (2060.00, 7568.0), (2061.00, 7587.0)

UNITS: persons/year

NIM_Arrivals_L[M, A74] = GRAPH(TIME)

Points: (2011.00, 1710.0), (2012.00, 1850.0), (2013.00, 2160.0), (2014.00, 2030.0), (2015.00, 2400.0), (2016.00, 2800.0), (2017.00, 3210.0), (2018.00, 2852.0), (2019.00, 2949.0), (2020.00, 3044.0), (2021.00, 3115.0), (2022.00, 3118.0), (2023.00, 3165.0), (2024.00, 3218.0), (2025.00, 3280.0), (2026.00, 3360.0), (2027.00, 3434.0), (2028.00, 3507.0), (2029.00, 3596.0), (2030.00, 3674.0), (2031.00, 3762.0), (2032.00, 3848.0), (2033.00, 3909.0), (2034.00, 3950.0), (2035.00, 3954.0), (2036.00, 3933.0), (2037.00, 3914.0), (2038.00, 3924.0), (2039.00, 3976.0), (2040.00, 4062.0), (2041.00, 4201.0), (2042.00, 4319.0), (2043.00, 4383.0), (2044.00, 4404.0), (2045.00, 4397.0), (2046.00, 4347.0), (2047.00, 4313.0), (2048.00, 4314.0), (2049.00, 4355.0), (2050.00, 4429.0), (2051.00, 4556.0), (2052.00, 4703.0), (2053.00, 4871.0), (2054.00, 5017.0), (2055.00, 5156.0), (2056.00, 5263.0), (2057.00, 5340.0), (2058.00, 5399.0), (2059.00, 5463.0), (2060.00, 5532.0), (2061.00, 5573.0)

UNITS: persons/year

NIM_Arrivals_L[M, A79] = GRAPH(TIME)

Points: (2011.00, 902.0), (2012.00, 941.0), (2013.00, 1089.0), (2014.00, 1032.0), (2015.00, 1107.0), (2016.00, 1207.0), (2017.00, 1268.0), (2018.00, 1524.0), (2019.00, 1601.0), (2020.00, 1685.0), (2021.00, 1770.0), (2022.00, 1913.0), (2023.00, 2030.0), (2024.00, 2105.0), (2025.00, 2175.0), (2026.00, 2237.0), (2027.00, 2239.0), (2028.00, 2274.0), (2029.00, 2317.0), (2030.00, 2366.0), (2031.00, 2425.0), (2032.00, 2482.0), (2033.00, 2538.0), (2034.00, 2605.0), (2035.00, 2664.0), (2036.00, 2730.0), (2037.00, 2792.0), (2038.00, 2842.0), (2039.00, 2873.0), (2040.00, 2880.0), (2041.00, 2865.0), (2042.00, 2852.0), (2043.00, 2858.0), (2044.00, 2894.0), (2045.00, 2957.0), (2046.00, 3057.0), (2047.00, 3147.0), (2048.00, 3199.0), (2049.00, 3220.0), (2050.00, 3220.0), (2051.00, 3183.0), (2052.00, 3158.0), (2053.00, 3158.0), (2054.00, 3188.0), (2055.00, 3242.0), (2056.00, 3333.0), (2057.00, 3439.0), (2058.00, 3564.0), (2059.00, 3673.0), (2060.00, 3777.0), (2061.00, 3859.0)

UNITS: persons/year

NIM_Arrivals_L[M, A84] = GRAPH(TIME)

Points: (2011.00, 671.0), (2012.00, 700.0), (2013.00, 810.0), (2014.00, 768.0), (2015.00, 823.0), (2016.00, 898.0), (2017.00, 943.0), (2018.00, 933.0), (2019.00, 979.0), (2020.00, 1032.0), (2021.00, 1080.0), (2022.00, 1131.0), (2023.00, 1176.0), (2024.00, 1243.0), (2025.00, 1309.0), (2026.00, 1384.0), (2027.00, 1501.0), (2028.00, 1593.0), (2029.00, 1653.0), (2030.00, 1707.0), (2031.00, 1759.0), (2032.00, 1765.0), (2033.00, 1795.0), (2034.00, 1832.0), (2035.00, 1873.0), (2036.00, 1921.0), (2037.00, 1970.0), (2038.00, 2016.0), (2039.00, 2070.0), (2040.00, 2119.0), (2041.00, 2174.0), (2042.00, 2226.0), (2043.00, 2267.0), (2044.00, 2291.0), (2045.00, 2297.0), (2046.00, 2287.0), (2047.00, 2278.0), (2048.00, 2288.0), (2049.00, 2321.0), (2050.00, 2375.0), (2051.00, 2459.0), (2052.00, 2531.0), (2053.00, 2571.0), (2054.00, 2586.0), (2055.00, 2587.0), (2056.00, 2560.0), (2057.00, 2545.0), (2058.00, 2546.0), (2059.00, 2573.0), (2060.00, 2621.0), (2061.00, 2698.0)

UNITS: persons/year

NIM_Arrivals_L[M, A89] = GRAPH(TIME)

Points: (2011.00, 359.0), (2012.00, 375.0), (2013.00, 434.0), (2014.00, 411.0), (2015.00, 441.0), (2016.00, 481.0), (2017.00, 505.0), (2018.00, 519.0), (2019.00, 525.0), (2020.00, 535.0), (2021.00, 551.0), (2022.00, 578.0), (2023.00, 607.0), (2024.00, 641.0), (2025.00, 677.0), (2026.00, 712.0), (2027.00, 749.0), (2028.00, 783.0), (2029.00, 828.0), (2030.00, 874.0), (2031.00, 927.0), (2032.00, 1006.0), (2033.00, 1072.0), (2034.00, 1122.0), (2035.00, 1164.0), (2036.00, 1199.0), (2037.00, 1207.0), (2038.00, 1231.0), (2039.00, 1257.0), (2040.00, 1290.0), (2041.00, 1326.0), (2042.00, 1362.0), (2043.00, 1398.0), (2044.00, 1440.0), (2045.00, 1477.0), (2046.00, 1518.0), (2047.00, 1559.0), (2048.00, 1592.0), (2049.00, 1616.0), (2050.00, 1626.0), (2051.00, 1624.0), (2052.00, 1621.0), (2053.00, 1628.0), (2054.00, 1652.0), (2055.00, 1691.0), (2056.00, 1754.0), (2057.00, 1811.0), (2058.00, 1852.0), (2059.00, 1871.0), (2060.00, 1876.0), (2061.00, 1860.0)

UNITS: persons/year

NIM_Arrivals_L[M, A94] = GRAPH(TIME)

Points: (2011.00, 113.0), (2012.00, 118.0), (2013.00, 136.0), (2014.00, 129.0), (2015.00, 139.0), (2016.00, 151.0), (2017.00, 159.0), (2018.00, 224.0), (2019.00, 233.0), (2020.00, 244.0), (2021.00, 254.0), (2022.00, 260.0), (2023.00, 263.0), (2024.00, 269.0), (2025.00, 275.0), (2026.00, 286.0), (2027.00, 303.0), (2028.00, 320.0), (2029.00, 339.0), (2030.00, 359.0), (2031.00, 376.0), (2032.00, 400.0), (2033.00, 419.0), (2034.00, 447.0), (2035.00, 477.0), (2036.00, 504.0), (2037.00, 556.0), (2038.00, 592.0), (2039.00, 612.0), (2040.00, 638.0), (2041.00, 655.0), (2042.00, 664.0), (2043.00, 683.0), (2044.00, 701.0), (2045.00, 721.0), (2046.00, 747.0), (2047.00, 769.0), (2048.00, 791.0), (2049.00, 819.0), (2050.00, 842.0), (2051.00, 871.0), (2052.00, 896.0), (2053.00, 915.0), (2054.00, 931.0), (2055.00, 934.0), (2056.00, 935.0), (2057.00, 937.0), (2058.00, 948.0), (2059.00, 970.0), (2060.00, 1000.0), (2061.00, 1044.0)

UNITS: persons/year

NIM_Arrivals_L[M, A99] = GRAPH(TIME)

Points: (2011.00, 21.0), (2012.00, 21.0), (2013.00, 25.0), (2014.00, 24.0), (2015.00, 25.0), (2016.00, 28.0), (2017.00, 29.0), (2018.00, 50.0), (2019.00, 56.0), (2020.00, 60.0), (2021.00, 64.0), (2022.00, 67.0), (2023.00, 71.0), (2024.00, 73.0), (2025.00, 78.0), (2026.00, 81.0), (2027.00, 82.0), (2028.00, 84.0), (2029.00, 86.0), (2030.00, 87.0), (2031.00, 92.0), (2032.00, 98.0), (2033.00, 105.0), (2034.00, 110.0), (2035.00, 118.0), (2036.00, 126.0), (2037.00, 134.0), (2038.00, 142.0), (2039.00, 151.0), (2040.00, 164.0), (2041.00, 174.0), (2042.00, 195.0), (2043.00, 208.0), (2044.00, 219.0), (2045.00, 227.0), (2046.00, 235.0), (2047.00, 240.0), (2048.00, 249.0), (2049.00, 258.0), (2050.00, 267.0), (2051.00, 278.0), (2052.00, 288.0), (2053.00, 298.0), (2054.00, 312.0), (2055.00, 322.0), (2056.00, 337.0), (2057.00, 349.0), (2058.00, 360.0), (2059.00, 366.0), (2060.00, 370.0), (2061.00, 373.0)

UNITS: persons/year

NIM_Arrivals_L[M, A104] = GRAPH(TIME)

Points: (2011.00, 2.0), (2012.00, 2.0), (2013.00, 2.0), (2014.00, 2.0), (2015.00, 2.0), (2016.00, 3.0), (2017.00, 3.0), (2018.00, 7.0), (2019.00, 7.0), (2020.00, 8.0), (2021.00, 10.0), (2022.00, 11.0), (2023.00, 12.0), (2024.00, 13.0), (2025.00, 14.0), (2026.00, 15.0), (2027.00, 16.0), (2028.00, 17.0), (2029.00, 18.0), (2030.00, 19.0), (2031.00, 20.0), (2032.00, 21.0), (2033.00, 21.0), (2034.00, 22.0), (2035.00, 23.0), (2036.00, 24.0), (2037.00, 26.0), (2038.00, 27.0), (2039.00, 29.0), (2040.00, 32.0), (2041.00, 34.0), (2042.00, 36.0), (2043.00, 39.0), (2044.00, 42.0), (2045.00, 46.0), (2046.00, 49.0), (2047.00, 55.0), (2048.00, 59.0), (2049.00, 63.0), (2050.00, 66.0), (2051.00, 70.0), (2052.00, 73.0), (2053.00, 77.0), (2054.00, 80.0), (2055.00, 84.0), (2056.00, 89.0), (2057.00, 93.0), (2058.00, 97.0), (2059.00, 102.0), (2060.00, 107.0), (2061.00, 112.0)

UNITS: persons/year

NIM_Arrivals_L[F, A4] = GRAPH(TIME)

Points: (2011.00, 13960.0), (2012.00, 12760.0), (2013.00, 13070.0), (2014.00, 12660.0), (2015.00, 14180.0), (2016.00, 14990.0), (2017.00, 14890.0), (2018.00, 13722.0), (2019.00, 13773.0), (2020.00, 13806.0), (2021.00, 13813.0), (2022.00, 13801.0), (2023.00, 13769.0), (2024.00, 13717.0), (2025.00, 13648.0), (2026.00, 13566.0), (2027.00, 13483.0), (2028.00, 13422.0), (2029.00, 13397.0), (2030.00, 13404.0), (2031.00, 13446.0), (2032.00, 13510.0), (2033.00, 13576.0), (2034.00, 13650.0), (2035.00, 13731.0), (2036.00, 13822.0), (2037.00, 13920.0), (2038.00, 14025.0), (2039.00, 14137.0), (2040.00, 14254.0), (2041.00, 14375.0), (2042.00, 14494.0), (2043.00, 14612.0), (2044.00, 14726.0), (2045.00, 14834.0), (2046.00, 14932.0), (2047.00, 15021.0), (2048.00, 15098.0), (2049.00, 15165.0), (2050.00, 15219.0), (2051.00, 15264.0), (2052.00, 15299.0), (2053.00, 15322.0), (2054.00, 15340.0), (2055.00, 15351.0), (2056.00, 15358.0), (2057.00, 15364.0), (2058.00, 15367.0), (2059.00, 15373.0), (2060.00, 15381.0), (2061.00, 15394.0)

UNITS: persons/year

NIM_Arrivals_L[F, A9] = GRAPH(TIME)

Points: (2011.00, 10430.0), (2012.00, 9540.0), (2013.00, 10480.0), (2014.00, 10080.0), (2015.00, 11030.0), (2016.00, 11750.0), (2017.00, 11820.0), (2018.00, 11117.0), (2019.00, 11245.0), (2020.00, 11348.0), (2021.00, 11466.0), (2022.00, 11505.0), (2023.00, 11548.0), (2024.00, 11582.0), (2025.00, 11601.0), (2026.00, 11605.0), (2027.00, 11591.0), (2028.00, 11562.0), (2029.00, 11518.0), (2030.00, 11460.0), (2031.00, 11392.0), (2032.00, 11331.0), (2033.00, 11294.0), (2034.00, 11281.0), (2035.00, 11292.0), (2036.00, 11328.0), (2037.00, 11380.0), (2038.00, 11435.0), (2039.00, 11496.0), (2040.00, 11565.0), (2041.00, 11637.0), (2042.00, 11717.0), (2043.00, 11805.0), (2044.00, 11896.0), (2045.00, 11991.0), (2046.00, 12086.0), (2047.00, 12185.0), (2048.00, 12280.0), (2049.00, 12368.0), (2050.00, 12454.0), (2051.00, 12532.0), (2052.00, 12602.0), (2053.00, 12663.0), (2054.00, 12715.0), (2055.00, 12758.0), (2056.00, 12791.0), (2057.00, 12817.0), (2058.00, 12836.0), (2059.00, 12849.0), (2060.00, 12859.0), (2061.00, 12863.0)

UNITS: persons/year

NIM_Arrivals_L[F, A14] = GRAPH(TIME)

Points: (2011.00, 8260.0), (2012.00, 7330.0), (2013.00, 8100.0), (2014.00, 7400.0), (2015.00, 7910.0), (2016.00, 8760.0), (2017.00, 8770.0), (2018.00, 8239.0), (2019.00, 8428.0), (2020.00, 8599.0), (2021.00, 8688.0), (2022.00, 8853.0), (2023.00, 8962.0), (2024.00, 9057.0), (2025.00, 9136.0), (2026.00, 9228.0), (2027.00, 9257.0), (2028.00, 9293.0), (2029.00, 9318.0), (2030.00, 9333.0), (2031.00, 9336.0), (2032.00, 9327.0), (2033.00, 9304.0), (2034.00, 9271.0), (2035.00, 9227.0), (2036.00, 9174.0), (2037.00, 9127.0), (2038.00, 9099.0), (2039.00, 9089.0), (2040.00, 9097.0), (2041.00, 9126.0), (2042.00, 9166.0), (2043.00, 9210.0), (2044.00, 9258.0), (2045.00, 9310.0), (2046.00, 9368.0), (2047.00, 9431.0), (2048.00, 9498.0), (2049.00, 9569.0), (2050.00, 9644.0), (2051.00, 9721.0), (2052.00, 9795.0), (2053.00, 9870.0), (2054.00, 9942.0), (2055.00, 10008.0), (2056.00, 10070.0), (2057.00, 10123.0), (2058.00, 10172.0), (2059.00, 10214.0), (2060.00, 10247.0), (2061.00, 10273.0)

UNITS: persons/year

NIM_Arrivals_L[F, A19] = GRAPH(TIME)

Points: (2011.00, 12730.0), (2012.00, 11850.0), (2013.00, 12750.0), (2014.00, 11510.0), (2015.00, 11560.0), (2016.00, 11910.0), (2017.00, 11990.0), (2018.00, 12125.0), (2019.00, 12180.0), (2020.00, 12237.0), (2021.00, 12355.0), (2022.00, 12539.0), (2023.00, 12782.0), (2024.00, 13091.0), (2025.00, 13372.0), (2026.00, 13527.0), (2027.00, 13765.0), (2028.00, 13931.0), (2029.00, 14042.0), (2030.00, 14177.0), (2031.00, 14374.0), (2032.00, 14404.0), (2033.00, 14453.0), (2034.00, 14494.0), (2035.00, 14524.0), (2036.00, 14535.0), (2037.00, 14530.0), (2038.00, 14508.0), (2039.00, 14463.0), (2040.00, 14403.0), (2041.00, 14330.0), (2042.00, 14255.0), (2043.00, 14192.0), (2044.00, 14141.0), (2045.00, 14118.0), (2046.00, 14143.0), (2047.00, 14196.0), (2048.00, 14253.0), (2049.00, 14316.0), (2050.00, 14382.0), (2051.00, 14458.0), (2052.00, 14541.0), (2053.00, 14632.0), (2054.00, 14729.0), (2055.00, 14830.0), (2056.00, 14936.0), (2057.00, 15044.0), (2058.00, 15151.0), (2059.00, 15255.0), (2060.00, 15353.0), (2061.00, 15444.0)

UNITS: persons/year

NIM_Arrivals_L[F, A24] = GRAPH(TIME)

Points: (2011.00, 24200.0), (2012.00, 25110.0), (2013.00, 25430.0), (2014.00, 23760.0), (2015.00, 23010.0), (2016.00, 24220.0), (2017.00, 24350.0), (2018.00, 26032.0), (2019.00, 26109.0), (2020.00, 26092.0), (2021.00, 26131.0), (2022.00, 26133.0), (2023.00, 26205.0), (2024.00, 26282.0), (2025.00, 26453.0), (2026.00, 26798.0), (2027.00, 27227.0), (2028.00, 27759.0), (2029.00, 28308.0), (2030.00, 28811.0), (2031.00, 29102.0), (2032.00, 29539.0), (2033.00, 29849.0), (2034.00, 30126.0), (2035.00, 30366.0), (2036.00, 30653.0), (2037.00, 30735.0), (2038.00, 30833.0), (2039.00, 30911.0), (2040.00, 30959.0), (2041.00, 30976.0), (2042.00, 30953.0), (2043.00, 30893.0), (2044.00, 30800.0), (2045.00, 30675.0), (2046.00, 30526.0), (2047.00, 30382.0), (2048.00, 30277.0), (2049.00, 30218.0), (2050.00, 30222.0), (2051.00, 30291.0), (2052.00, 30399.0), (2053.00, 30515.0), (2054.00, 30645.0), (2055.00, 30786.0), (2056.00, 30943.0), (2057.00, 31115.0), (2058.00, 31300.0), (2059.00, 31497.0), (2060.00, 31704.0), (2061.00, 31916.0)

UNITS: persons/year

NIM_Arrivals_L[F, A29] = GRAPH(TIME)

Points: (2011.00, 25540.0), (2012.00, 26140.0), (2013.00, 27210.0), (2014.00, 25850.0), (2015.00, 26540.0), (2016.00, 28290.0), (2017.00, 28450.0), (2018.00, 27759.0), (2019.00, 27804.0), (2020.00, 27825.0), (2021.00, 27792.0), (2022.00, 27845.0), (2023.00, 27859.0), (2024.00, 27899.0), (2025.00, 27887.0), (2026.00, 27932.0), (2027.00, 27930.0), (2028.00, 28002.0), (2029.00, 28107.0), (2030.00, 28321.0), (2031.00, 28726.0), (2032.00, 29170.0), (2033.00, 29695.0), (2034.00, 30212.0), (2035.00, 30688.0), (2036.00, 30954.0), (2037.00, 31393.0), (2038.00, 31708.0), (2039.00, 31979.0), (2040.00, 32203.0), (2041.00, 32459.0), (2042.00, 32551.0), (2043.00, 32651.0), (2044.00, 32729.0), (2045.00, 32776.0), (2046.00, 32790.0), (2047.00, 32766.0), (2048.00, 32705.0), (2049.00, 32612.0), (2050.00, 32489.0), (2051.00, 32345.0), (2052.00, 32212.0), (2053.00, 32131.0), (2054.00, 32101.0), (2055.00, 32126.0), (2056.00, 32206.0), (2057.00, 32320.0), (2058.00, 32442.0), (2059.00, 32576.0), (2060.00, 32724.0), (2061.00, 32887.0)

UNITS: persons/year

NIM_Arrivals_L[F, A34] = GRAPH(TIME)

Points: (2011.00, 18390.0), (2012.00, 17710.0), (2013.00, 18900.0), (2014.00, 18820.0), (2015.00, 20650.0), (2016.00, 22200.0), (2017.00, 22350.0), (2018.00, 21683.0), (2019.00, 21961.0), (2020.00, 22238.0), (2021.00, 22450.0), (2022.00, 22568.0), (2023.00, 22636.0), (2024.00, 22647.0), (2025.00, 22655.0), (2026.00, 22631.0), (2027.00, 22674.0), (2028.00, 22683.0), (2029.00, 22711.0), (2030.00, 22704.0), (2031.00, 22737.0), (2032.00, 22737.0), (2033.00, 22791.0), (2034.00, 22874.0), (2035.00, 23037.0), (2036.00, 23347.0), (2037.00, 23684.0), (2038.00, 24081.0), (2039.00, 24471.0), (2040.00, 24831.0), (2041.00, 25031.0), (2042.00, 25363.0), (2043.00, 25601.0), (2044.00, 25806.0), (2045.00, 25975.0), (2046.00, 26166.0), (2047.00, 26238.0), (2048.00, 26315.0), (2049.00, 26374.0), (2050.00, 26412.0), (2051.00, 26422.0), (2052.00, 26407.0), (2053.00, 26362.0), (2054.00, 26293.0), (2055.00, 26201.0), (2056.00, 26093.0), (2057.00, 25994.0), (2058.00, 25934.0), (2059.00, 25914.0), (2060.00, 25935.0), (2061.00, 25997.0)

UNITS: persons/year

NIM_Arrivals_L[F, A39] = GRAPH(TIME)

Points: (2011.00, 14300.0), (2012.00, 12580.0), (2013.00, 13400.0), (2014.00, 12800.0), (2015.00, 13890.0), (2016.00, 15350.0), (2017.00, 15660.0), (2018.00, 14861.0), (2019.00, 15338.0), (2020.00, 15781.0), (2021.00, 16100.0), (2022.00, 16345.0), (2023.00, 16521.0), (2024.00, 16715.0), (2025.00, 16912.0), (2026.00, 17064.0), (2027.00, 17145.0), (2028.00, 17192.0), (2029.00, 17202.0), (2030.00, 17211.0), (2031.00, 17194.0), (2032.00, 17225.0), (2033.00, 17230.0), (2034.00, 17251.0), (2035.00, 17248.0), (2036.00, 17270.0), (2037.00, 17272.0), (2038.00, 17312.0), (2039.00, 17373.0), (2040.00, 17491.0), (2041.00, 17721.0), (2042.00, 17966.0), (2043.00, 18252.0), (2044.00, 18533.0), (2045.00, 18794.0), (2046.00, 18937.0), (2047.00, 19181.0), (2048.00, 19353.0), (2049.00, 19500.0), (2050.00, 19620.0), (2051.00, 19761.0), (2052.00, 19813.0), (2053.00, 19871.0), (2054.00, 19913.0), (2055.00, 19941.0), (2056.00, 19949.0), (2057.00, 19938.0), (2058.00, 19905.0), (2059.00, 19856.0), (2060.00, 19790.0), (2061.00, 19711.0)

UNITS: persons/year

NIM_Arrivals_L[F, A44] = GRAPH(TIME)

Points: (2011.00, 10350.0), (2012.00, 9440.0), (2013.00, 10600.0), (2014.00, 9630.0), (2015.00, 10240.0), (2016.00, 10980.0), (2017.00, 11120.0), (2018.00, 9778.0), (2019.00, 9850.0), (2020.00, 10006.0), (2021.00, 10269.0), (2022.00, 10573.0), (2023.00, 10932.0), (2024.00, 11263.0), (2025.00, 11577.0), (2026.00, 11805.0), (2027.00, 11980.0), (2028.00, 12105.0), (2029.00, 12242.0), (2030.00, 12378.0), (2031.00, 12487.0), (2032.00, 12547.0), (2033.00, 12583.0), (2034.00, 12591.0), (2035.00, 12594.0), (2036.00, 12583.0), (2037.00, 12606.0), (2038.00, 12610.0), (2039.00, 12626.0), (2040.00, 12625.0), (2041.00, 12639.0), (2042.00, 12640.0), (2043.00, 12668.0), (2044.00, 12711.0), (2045.00, 12795.0), (2046.00, 12954.0), (2047.00, 13127.0), (2048.00, 13332.0), (2049.00, 13530.0), (2050.00, 13716.0), (2051.00, 13818.0), (2052.00, 13988.0), (2053.00, 14111.0), (2054.00, 14216.0), (2055.00, 14304.0), (2056.00, 14405.0), (2057.00, 14442.0), (2058.00, 14482.0), (2059.00, 14514.0), (2060.00, 14532.0), (2061.00, 14538.0)

UNITS: persons/year

NIM_Arrivals_L[F, A49] = GRAPH(TIME)

Points: (2011.00, 7700.0), (2012.00, 7240.0), (2013.00, 7690.0), (2014.00, 7170.0), (2015.00, 7610.0), (2016.00, 8620.0), (2017.00, 9070.0), (2018.00, 8453.0), (2019.00, 8479.0), (2020.00, 8459.0), (2021.00, 8329.0), (2022.00, 8226.0), (2023.00, 8200.0), (2024.00, 8252.0), (2025.00, 8373.0), (2026.00, 8582.0), (2027.00, 8827.0), (2028.00, 9119.0), (2029.00, 9393.0), (2030.00, 9654.0), (2031.00, 9845.0), (2032.00, 9995.0), (2033.00, 10100.0), (2034.00, 10215.0), (2035.00, 10325.0), (2036.00, 10416.0), (2037.00, 10471.0), (2038.00, 10505.0), (2039.00, 10516.0), (2040.00, 10516.0), (2041.00, 10507.0), (2042.00, 10527.0), (2043.00, 10534.0), (2044.00, 10548.0), (2045.00, 10546.0), (2046.00, 10559.0), (2047.00, 10561.0), (2048.00, 10586.0), (2049.00, 10620.0), (2050.00, 10687.0), (2051.00, 10814.0), (2052.00, 10956.0), (2053.00, 11122.0), (2054.00, 11288.0), (2055.00, 11444.0), (2056.00, 11530.0), (2057.00, 11670.0), (2058.00, 11771.0), (2059.00, 11860.0), (2060.00, 11933.0), (2061.00, 12019.0)

UNITS: persons/year

NIM_Arrivals_L[F, A54] = GRAPH(TIME)

Points: (2011.00, 7140.0), (2012.00, 7040.0), (2013.00, 7400.0), (2014.00, 6650.0), (2015.00, 6810.0), (2016.00, 7850.0), (2017.00, 8200.0), (2018.00, 7416.0), (2019.00, 7495.0), (2020.00, 7636.0), (2021.00, 7892.0), (2022.00, 8124.0), (2023.00, 8265.0), (2024.00, 8298.0), (2025.00, 8283.0), (2026.00, 8154.0), (2027.00, 8053.0), (2028.00, 8026.0), (2029.00, 8072.0), (2030.00, 8186.0), (2031.00, 8384.0), (2032.00, 8619.0), (2033.00, 8899.0), (2034.00, 9165.0), (2035.00, 9420.0), (2036.00, 9610.0), (2037.00, 9758.0), (2038.00, 9860.0), (2039.00, 9970.0), (2040.00, 10076.0), (2041.00, 10166.0), (2042.00, 10226.0), (2043.00, 10261.0), (2044.00, 10272.0), (2045.00, 10273.0), (2046.00, 10265.0), (2047.00, 10285.0), (2048.00, 10295.0), (2049.00, 10308.0), (2050.00, 10305.0), (2051.00, 10320.0), (2052.00, 10323.0), (2053.00, 10348.0), (2054.00, 10381.0), (2055.00, 10445.0), (2056.00, 10565.0), (2057.00, 10703.0), (2058.00, 10865.0), (2059.00, 11028.0), (2060.00, 11179.0), (2061.00, 11266.0)

UNITS: persons/year

NIM_Arrivals_L[F, A59] = GRAPH(TIME)

Points: (2011.00, 5980.0), (2012.00, 6080.0), (2013.00, 6410.0), (2014.00, 5930.0), (2015.00, 6380.0), (2016.00, 7380.0), (2017.00, 8040.0), (2018.00, 7260.0), (2019.00, 7327.0), (2020.00, 7336.0), (2021.00, 7285.0), (2022.00, 7244.0), (2023.00, 7238.0), (2024.00, 7319.0), (2025.00, 7456.0), (2026.00, 7708.0), (2027.00, 7936.0), (2028.00, 8071.0), (2029.00, 8102.0), (2030.00, 8084.0), (2031.00, 7960.0), (2032.00, 7867.0), (2033.00, 7844.0), (2034.00, 7892.0), (2035.00, 8005.0), (2036.00, 8200.0), (2037.00, 8431.0), (2038.00, 8704.0), (2039.00, 8962.0), (2040.00, 9209.0), (2041.00, 9391.0), (2042.00, 9535.0), (2043.00, 9635.0), (2044.00, 9742.0), (2045.00, 9849.0), (2046.00, 9937.0), (2047.00, 9995.0), (2048.00, 10029.0), (2049.00, 10040.0), (2050.00, 10042.0), (2051.00, 10038.0), (2052.00, 10059.0), (2053.00, 10069.0), (2054.00, 10082.0), (2055.00, 10083.0), (2056.00, 10099.0), (2057.00, 10104.0), (2058.00, 10129.0), (2059.00, 10163.0), (2060.00, 10228.0), (2061.00, 10347.0)

UNITS: persons/year

NIM_Arrivals_L[F, A64] = GRAPH(TIME)

Points: (2011.00, 4730.0), (2012.00, 4720.0), (2013.00, 5310.0), (2014.00, 4960.0), (2015.00, 5210.0), (2016.00, 6270.0), (2017.00, 6430.0), (2018.00, 5637.0), (2019.00, 5774.0), (2020.00, 5899.0), (2021.00, 6043.0), (2022.00, 6170.0), (2023.00, 6267.0), (2024.00, 6324.0), (2025.00, 6333.0), (2026.00, 6291.0), (2027.00, 6258.0), (2028.00, 6255.0), (2029.00, 6327.0), (2030.00, 6448.0), (2031.00, 6667.0), (2032.00, 6859.0), (2033.00, 6975.0), (2034.00, 6998.0), (2035.00, 6985.0), (2036.00, 6881.0), (2037.00, 6803.0), (2038.00, 6783.0), (2039.00, 6827.0), (2040.00, 6926.0), (2041.00, 7094.0), (2042.00, 7291.0), (2043.00, 7529.0), (2044.00, 7749.0), (2045.00, 7962.0), (2046.00, 8118.0), (2047.00, 8242.0), (2048.00, 8327.0), (2049.00, 8421.0), (2050.00, 8513.0), (2051.00, 8589.0), (2052.00, 8638.0), (2053.00, 8668.0), (2054.00, 8678.0), (2055.00, 8683.0), (2056.00, 8678.0), (2057.00, 8698.0), (2058.00, 8707.0), (2059.00, 8720.0), (2060.00, 8721.0), (2061.00, 8736.0)

UNITS: persons/year

NIM_Arrivals_L[F, A69] = GRAPH(TIME)

Points: (2011.00, 2820.0), (2012.00, 3070.0), (2013.00, 3560.0), (2014.00, 3510.0), (2015.00, 3910.0), (2016.00, 4390.0), (2017.00, 4650.0), (2018.00, 3940.0), (2019.00, 4028.0), (2020.00, 4121.0), (2021.00, 4219.0), (2022.00, 4321.0), (2023.00, 4422.0), (2024.00, 4530.0), (2025.00, 4631.0), (2026.00, 4746.0), (2027.00, 4845.0), (2028.00, 4922.0), (2029.00, 4970.0), (2030.00, 4973.0), (2031.00, 4945.0), (2032.00, 4920.0), (2033.00, 4922.0), (2034.00, 4984.0), (2035.00, 5080.0), (2036.00, 5257.0), (2037.00, 5407.0), (2038.00, 5490.0), (2039.00, 5507.0), (2040.00, 5492.0), (2041.00, 5412.0), (2042.00, 5353.0), (2043.00, 5341.0), (2044.00, 5378.0), (2045.00, 5459.0), (2046.00, 5594.0), (2047.00, 5751.0), (2048.00, 5938.0), (2049.00, 6112.0), (2050.00, 6276.0), (2051.00, 6399.0), (2052.00, 6494.0), (2053.00, 6563.0), (2054.00, 6638.0), (2055.00, 6712.0), (2056.00, 6772.0), (2057.00, 6811.0), (2058.00, 6831.0), (2059.00, 6840.0), (2060.00, 6845.0), (2061.00, 6844.0)

UNITS: persons/year

NIM_Arrivals_L[F, A74] = GRAPH(TIME)

Points: (2011.00, 1540.0), (2012.00, 1690.0), (2013.00, 2000.0), (2014.00, 1840.0), (2015.00, 2210.0), (2016.00, 2550.0), (2017.00, 2830.0), (2018.00, 2574.0), (2019.00, 2664.0), (2020.00, 2752.0), (2021.00, 2844.0), (2022.00, 2859.0), (2023.00, 2917.0), (2024.00, 2984.0), (2025.00, 3055.0), (2026.00, 3127.0), (2027.00, 3205.0), (2028.00, 3282.0), (2029.00, 3363.0), (2030.00, 3440.0), (2031.00, 3529.0), (2032.00, 3603.0), (2033.00, 3661.0), (2034.00, 3694.0), (2035.00, 3700.0), (2036.00, 3679.0), (2037.00, 3660.0), (2038.00, 3663.0), (2039.00, 3707.0), (2040.00, 3776.0), (2041.00, 3908.0), (2042.00, 4019.0), (2043.00, 4080.0), (2044.00, 4092.0), (2045.00, 4085.0), (2046.00, 4028.0), (2047.00, 3984.0), (2048.00, 3973.0), (2049.00, 3999.0), (2050.00, 4057.0), (2051.00, 4157.0), (2052.00, 4271.0), (2053.00, 4409.0), (2054.00, 4536.0), (2055.00, 4658.0), (2056.00, 4746.0), (2057.00, 4816.0), (2058.00, 4866.0), (2059.00, 4922.0), (2060.00, 4977.0), (2061.00, 5019.0)

UNITS: persons/year

NIM_Arrivals_L[F, A79] = GRAPH(TIME)

Points: (2011.00, 1071.0), (2012.00, 1086.0), (2013.00, 1235.0), (2014.00, 1130.0), (2015.00, 1140.0), (2016.00, 1184.0), (2017.00, 1275.0), (2018.00, 1569.0), (2019.00, 1653.0), (2020.00, 1741.0), (2021.00, 1833.0), (2022.00, 1976.0), (2023.00, 2092.0), (2024.00, 2170.0), (2025.00, 2239.0), (2026.00, 2320.0), (2027.00, 2332.0), (2028.00, 2379.0), (2029.00, 2436.0), (2030.00, 2494.0), (2031.00, 2556.0), (2032.00, 2620.0), (2033.00, 2684.0), (2034.00, 2752.0), (2035.00, 2818.0), (2036.00, 2890.0), (2037.00, 2953.0), (2038.00, 3002.0), (2039.00, 3033.0), (2040.00, 3037.0), (2041.00, 3020.0), (2042.00, 3005.0), (2043.00, 3006.0), (2044.00, 3041.0), (2045.00, 3100.0), (2046.00, 3205.0), (2047.00, 3299.0), (2048.00, 3353.0), (2049.00, 3363.0), (2050.00, 3362.0), (2051.00, 3316.0), (2052.00, 3276.0), (2053.00, 3270.0), (2054.00, 3289.0), (2055.00, 3337.0), (2056.00, 3418.0), (2057.00, 3511.0), (2058.00, 3626.0), (2059.00, 3729.0), (2060.00, 3831.0), (2061.00, 3904.0)

UNITS: persons/year

NIM_Arrivals_L[F, A84] = GRAPH(TIME)

Points: (2011.00, 911.0), (2012.00, 924.0), (2013.00, 1050.0), (2014.00, 961.0), (2015.00, 970.0), (2016.00, 1007.0), (2017.00, 1084.0), (2018.00, 1143.0), (2019.00, 1184.0), (2020.00, 1225.0), (2021.00, 1269.0), (2022.00, 1326.0), (2023.00, 1382.0), (2024.00, 1460.0), (2025.00, 1541.0), (2026.00, 1622.0), (2027.00, 1757.0), (2028.00, 1857.0), (2029.00, 1933.0), (2030.00, 2002.0), (2031.00, 2071.0), (2032.00, 2085.0), (2033.00, 2131.0), (2034.00, 2182.0), (2035.00, 2237.0), (2036.00, 2293.0), (2037.00, 2353.0), (2038.00, 2411.0), (2039.00, 2474.0), (2040.00, 2533.0), (2041.00, 2600.0), (2042.00, 2658.0), (2043.00, 2703.0), (2044.00, 2734.0), (2045.00, 2740.0), (2046.00, 2727.0), (2047.00, 2715.0), (2048.00, 2716.0), (2049.00, 2747.0), (2050.00, 2800.0), (2051.00, 2898.0), (2052.00, 2983.0), (2053.00, 3035.0), (2054.00, 3053.0), (2055.00, 3051.0), (2056.00, 3008.0), (2057.00, 2976.0), (2058.00, 2969.0), (2059.00, 2989.0), (2060.00, 3032.0), (2061.00, 3107.0)

UNITS: persons/year

NIM_Arrivals_L[F, A89] = GRAPH(TIME)

Points: (2011.00, 623.0), (2012.00, 631.0), (2013.00, 718.0), (2014.00, 657.0), (2015.00, 663.0), (2016.00, 688.0), (2017.00, 741.0), (2018.00, 829.0), (2019.00, 833.0), (2020.00, 842.0), (2021.00, 861.0), (2022.00, 885.0), (2023.00, 912.0), (2024.00, 946.0), (2025.00, 985.0), (2026.00, 1023.0), (2027.00, 1070.0), (2028.00, 1117.0), (2029.00, 1185.0), (2030.00, 1254.0), (2031.00, 1325.0), (2032.00, 1439.0), (2033.00, 1524.0), (2034.00, 1584.0), (2035.00, 1643.0), (2036.00, 1692.0), (2037.00, 1709.0), (2038.00, 1751.0), (2039.00, 1794.0), (2040.00, 1842.0), (2041.00, 1893.0), (2042.00, 1943.0), (2043.00, 1994.0), (2044.00, 2048.0), (2045.00, 2099.0), (2046.00, 2157.0), (2047.00, 2206.0), (2048.00, 2246.0), (2049.00, 2270.0), (2050.00, 2275.0), (2051.00, 2263.0), (2052.00, 2256.0), (2053.00, 2260.0), (2054.00, 2295.0), (2055.00, 2346.0), (2056.00, 2433.0), (2057.00, 2510.0), (2058.00, 2549.0), (2059.00, 2562.0), (2060.00, 2554.0), (2061.00, 2522.0)

UNITS: persons/year

NIM_Arrivals_L[F, A94] = GRAPH(TIME)

Points: (2011.00, 263.0), (2012.00, 267.0), (2013.00, 304.0), (2014.00, 278.0), (2015.00, 280.0), (2016.00, 291.0), (2017.00, 314.0), (2018.00, 413.0), (2019.00, 421.0), (2020.00, 429.0), (2021.00, 436.0), (2022.00, 438.0), (2023.00, 440.0), (2024.00, 444.0), (2025.00, 451.0), (2026.00, 463.0), (2027.00, 478.0), (2028.00, 493.0), (2029.00, 514.0), (2030.00, 535.0), (2031.00, 556.0), (2032.00, 586.0), (2033.00, 613.0), (2034.00, 651.0), (2035.00, 693.0), (2036.00, 732.0), (2037.00, 800.0), (2038.00, 847.0), (2039.00, 878.0), (2040.00, 911.0), (2041.00, 942.0), (2042.00, 955.0), (2043.00, 980.0), (2044.00, 1008.0), (2045.00, 1037.0), (2046.00, 1069.0), (2047.00, 1100.0), (2048.00, 1131.0), (2049.00, 1167.0), (2050.00, 1198.0), (2051.00, 1235.0), (2052.00, 1265.0), (2053.00, 1289.0), (2054.00, 1305.0), (2055.00, 1310.0), (2056.00, 1306.0), (2057.00, 1305.0), (2058.00, 1314.0), (2059.00, 1342.0), (2060.00, 1373.0), (2061.00, 1431.0)

UNITS: persons/year

NIM_Arrivals_L[F, A99] = GRAPH(TIME)

Points: (2011.00, 69.0), (2012.00, 70.0), (2013.00, 79.0), (2014.00, 73.0), (2015.00, 73.0), (2016.00, 76.0), (2017.00, 82.0), (2018.00, 115.0), (2019.00, 122.0), (2020.00, 126.0), (2021.00, 131.0), (2022.00, 134.0), (2023.00, 137.0), (2024.00, 140.0), (2025.00, 144.0), (2026.00, 145.0), (2027.00, 146.0), (2028.00, 147.0), (2029.00, 149.0), (2030.00, 153.0), (2031.00, 157.0), (2032.00, 163.0), (2033.00, 170.0), (2034.00, 177.0), (2035.00, 186.0), (2036.00, 194.0), (2037.00, 205.0), (2038.00, 216.0), (2039.00, 232.0), (2040.00, 249.0), (2041.00, 263.0), (2042.00, 290.0), (2043.00, 310.0), (2044.00, 322.0), (2045.00, 335.0), (2046.00, 346.0), (2047.00, 353.0), (2048.00, 367.0), (2049.00, 377.0), (2050.00, 392.0), (2051.00, 406.0), (2052.00, 419.0), (2053.00, 435.0), (2054.00, 450.0), (2055.00, 465.0), (2056.00, 481.0), (2057.00, 496.0), (2058.00, 507.0), (2059.00, 514.0), (2060.00, 518.0), (2061.00, 519.0)

UNITS: persons/year

NIM_Arrivals_L[F, A104] = GRAPH(TIME)

Points: (2011.00, 9.0), (2012.00, 9.0), (2013.00, 11.0), (2014.00, 10.0), (2015.00, 10.0), (2016.00, 10.0), (2017.00, 11.0), (2018.00, 21.0), (2019.00, 22.0), (2020.00, 24.0), (2021.00, 27.0), (2022.00, 29.0), (2023.00, 31.0), (2024.00, 33.0), (2025.00, 34.0), (2026.00, 36.0), (2027.00, 37.0), (2028.00, 39.0), (2029.00, 40.0), (2030.00, 41.0), (2031.00, 42.0), (2032.00, 42.0), (2033.00, 43.0), (2034.00, 44.0), (2035.00, 45.0), (2036.00, 47.0), (2037.00, 49.0), (2038.00, 51.0), (2039.00, 53.0), (2040.00, 56.0), (2041.00, 59.0), (2042.00, 63.0), (2043.00, 67.0), (2044.00, 72.0), (2045.00, 77.0), (2046.00, 82.0), (2047.00, 91.0), (2048.00, 97.0), (2049.00, 102.0), (2050.00, 108.0), (2051.00, 113.0), (2052.00, 118.0), (2053.00, 124.0), (2054.00, 129.0), (2055.00, 135.0), (2056.00, 141.0), (2057.00, 148.0), (2058.00, 154.0), (2059.00, 161.0), (2060.00, 168.0), (2061.00, 175.0)

UNITS: persons/year

NIM_Arrivals_M[M, A4] = GRAPH(TIME)

Points: (2011.00, 14810.0), (2012.00, 13430.0), (2013.00, 14270.0), (2014.00, 13530.0), (2015.00, 14830.0), (2016.00, 16080.0), (2017.00, 16070.0), (2018.00, 15044.0), (2019.00, 15241.0), (2020.00, 15423.0), (2021.00, 15588.0), (2022.00, 15734.0), (2023.00, 15858.0), (2024.00, 15967.0), (2025.00, 16059.0), (2026.00, 16138.0), (2027.00, 16206.0), (2028.00, 16280.0), (2029.00, 16361.0), (2030.00, 16455.0), (2031.00, 16563.0), (2032.00, 16679.0), (2033.00, 16801.0), (2034.00, 16928.0), (2035.00, 17063.0), (2036.00, 17211.0), (2037.00, 17368.0), (2038.00, 17536.0), (2039.00, 17711.0), (2040.00, 17898.0), (2041.00, 18091.0), (2042.00, 18289.0), (2043.00, 18490.0), (2044.00, 18691.0), (2045.00, 18891.0), (2046.00, 19089.0), (2047.00, 19280.0), (2048.00, 19466.0), (2049.00, 19642.0), (2050.00, 19813.0), (2051.00, 19972.0), (2052.00, 20124.0), (2053.00, 20267.0), (2054.00, 20402.0), (2055.00, 20529.0), (2056.00, 20652.0), (2057.00, 20767.0), (2058.00, 20877.0), (2059.00, 20985.0), (2060.00, 21093.0), (2061.00, 21199.0)

UNITS: persons/year

NIM_Arrivals_M[M, A9] = GRAPH(TIME)

Points: (2011.00, 10830.0), (2012.00, 9840.0), (2013.00, 10930.0), (2014.00, 10360.0), (2015.00, 11460.0), (2016.00, 12550.0), (2017.00, 12610.0), (2018.00, 11673.0), (2019.00, 11870.0), (2020.00, 12061.0), (2021.00, 12286.0), (2022.00, 12443.0), (2023.00, 12603.0), (2024.00, 12757.0), (2025.00, 12899.0), (2026.00, 13029.0), (2027.00, 13143.0), (2028.00, 13240.0), (2029.00, 13324.0), (2030.00, 13396.0), (2031.00, 13456.0), (2032.00, 13513.0), (2033.00, 13577.0), (2034.00, 13647.0), (2035.00, 13724.0), (2036.00, 13810.0), (2037.00, 13905.0), (2038.00, 14002.0), (2039.00, 14108.0), (2040.00, 14219.0), (2041.00, 14338.0), (2042.00, 14465.0), (2043.00, 14601.0), (2044.00, 14743.0), (2045.00, 14895.0), (2046.00, 15051.0), (2047.00, 15210.0), (2048.00, 15371.0), (2049.00, 15532.0), (2050.00, 15692.0), (2051.00, 15849.0), (2052.00, 16000.0), (2053.00, 16148.0), (2054.00, 16289.0), (2055.00, 16423.0), (2056.00, 16552.0), (2057.00, 16671.0), (2058.00, 16786.0), (2059.00, 16894.0), (2060.00, 16995.0), (2061.00, 17092.0)

UNITS: persons/year

NIM_Arrivals_M[M, A14] = GRAPH(TIME)

Points: (2011.00, 8420.0), (2012.00, 7360.0), (2013.00, 8220.0), (2014.00, 7640.0), (2015.00, 8350.0), (2016.00, 9020.0), (2017.00, 9010.0), (2018.00, 8348.0), (2019.00, 8541.0), (2020.00, 8718.0), (2021.00, 8820.0), (2022.00, 8990.0), (2023.00, 9143.0), (2024.00, 9288.0), (2025.00, 9430.0), (2026.00, 9597.0), (2027.00, 9717.0), (2028.00, 9837.0), (2029.00, 9952.0), (2030.00, 10060.0), (2031.00, 10155.0), (2032.00, 10241.0), (2033.00, 10314.0), (2034.00, 10376.0), (2035.00, 10431.0), (2036.00, 10476.0), (2037.00, 10521.0), (2038.00, 10566.0), (2039.00, 10619.0), (2040.00, 10677.0), (2041.00, 10741.0), (2042.00, 10811.0), (2043.00, 10885.0), (2044.00, 10962.0), (2045.00, 11046.0), (2046.00, 11135.0), (2047.00, 11230.0), (2048.00, 11333.0), (2049.00, 11439.0), (2050.00, 11551.0), (2051.00, 11668.0), (2052.00, 11786.0), (2053.00, 11906.0), (2054.00, 12028.0), (2055.00, 12148.0), (2056.00, 12264.0), (2057.00, 12379.0), (2058.00, 12490.0), (2059.00, 12595.0), (2060.00, 12697.0), (2061.00, 12793.0)

UNITS: persons/year

NIM_Arrivals_M[M, A19] = GRAPH(TIME)

Points: (2011.00, 11990.0), (2012.00, 11040.0), (2013.00, 11660.0), (2014.00, 10790.0), (2015.00, 10870.0), (2016.00, 11300.0), (2017.00, 11040.0), (2018.00, 11313.0), (2019.00, 11399.0), (2020.00, 11496.0), (2021.00, 11622.0), (2022.00, 11842.0), (2023.00, 12110.0), (2024.00, 12406.0), (2025.00, 12664.0), (2026.00, 12814.0), (2027.00, 13039.0), (2028.00, 13232.0), (2029.00, 13391.0), (2030.00, 13575.0), (2031.00, 13855.0), (2032.00, 14000.0), (2033.00, 14157.0), (2034.00, 14313.0), (2035.00, 14459.0), (2036.00, 14593.0), (2037.00, 14714.0), (2038.00, 14820.0), (2039.00, 14910.0), (2040.00, 14985.0), (2041.00, 15049.0), (2042.00, 15106.0), (2043.00, 15162.0), (2044.00, 15219.0), (2045.00, 15280.0), (2046.00, 15359.0), (2047.00, 15450.0), (2048.00, 15542.0), (2049.00, 15640.0), (2050.00, 15741.0), (2051.00, 15851.0), (2052.00, 15971.0), (2053.00, 16099.0), (2054.00, 16234.0), (2055.00, 16378.0), (2056.00, 16526.0), (2057.00, 16681.0), (2058.00, 16840.0), (2059.00, 16997.0), (2060.00, 17155.0), (2061.00, 17312.0)

UNITS: persons/year

NIM_Arrivals_M[M, A24] = GRAPH(TIME)

Points: (2011.00, 23570.0), (2012.00, 23740.0), (2013.00, 24100.0), (2014.00, 24480.0), (2015.00, 21950.0), (2016.00, 22400.0), (2017.00, 22440.0), (2018.00, 25156.0), (2019.00, 25246.0), (2020.00, 25233.0), (2021.00, 25314.0), (2022.00, 25366.0), (2023.00, 25467.0), (2024.00, 25625.0), (2025.00, 25872.0), (2026.00, 26240.0), (2027.00, 26727.0), (2028.00, 27283.0), (2029.00, 27825.0), (2030.00, 28322.0), (2031.00, 28624.0), (2032.00, 29070.0), (2033.00, 29455.0), (2034.00, 29840.0), (2035.00, 30216.0), (2036.00, 30709.0), (2037.00, 31017.0), (2038.00, 31339.0), (2039.00, 31648.0), (2040.00, 31940.0), (2041.00, 32204.0), (2042.00, 32439.0), (2043.00, 32642.0), (2044.00, 32813.0), (2045.00, 32960.0), (2046.00, 33083.0), (2047.00, 33192.0), (2048.00, 33307.0), (2049.00, 33433.0), (2050.00, 33576.0), (2051.00, 33742.0), (2052.00, 33925.0), (2053.00, 34115.0), (2054.00, 34316.0), (2055.00, 34530.0), (2056.00, 34761.0), (2057.00, 35006.0), (2058.00, 35272.0), (2059.00, 35554.0), (2060.00, 35849.0), (2061.00, 36156.0)

UNITS: persons/year

NIM_Arrivals_M[M, A29] = GRAPH(TIME)

Points: (2011.00, 27820.0), (2012.00, 28020.0), (2013.00, 28720.0), (2014.00, 28290.0), (2015.00, 27110.0), (2016.00, 27720.0), (2017.00, 27340.0), (2018.00, 29425.0), (2019.00, 29597.0), (2020.00, 29743.0), (2021.00, 29877.0), (2022.00, 30058.0), (2023.00, 30182.0), (2024.00, 30266.0), (2025.00, 30280.0), (2026.00, 30392.0), (2027.00, 30450.0), (2028.00, 30567.0), (2029.00, 30775.0), (2030.00, 31101.0), (2031.00, 31585.0), (2032.00, 32138.0), (2033.00, 32750.0), (2034.00, 33334.0), (2035.00, 33872.0), (2036.00, 34189.0), (2037.00, 34707.0), (2038.00, 35163.0), (2039.00, 35606.0), (2040.00, 36037.0), (2041.00, 36555.0), (2042.00, 36913.0), (2043.00, 37279.0), (2044.00, 37631.0), (2045.00, 37958.0), (2046.00, 38253.0), (2047.00, 38514.0), (2048.00, 38743.0), (2049.00, 38936.0), (2050.00, 39101.0), (2051.00, 39241.0), (2052.00, 39373.0), (2053.00, 39515.0), (2054.00, 39671.0), (2055.00, 39844.0), (2056.00, 40041.0), (2057.00, 40255.0), (2058.00, 40476.0), (2059.00, 40711.0), (2060.00, 40961.0), (2061.00, 41230.0)

UNITS: persons/year

NIM_Arrivals_M[M, A34] = GRAPH(TIME)

Points: (2011.00, 19850.0), (2012.00, 20200.0), (2013.00, 21510.0), (2014.00, 21440.0), (2015.00, 22020.0), (2016.00, 23380.0), (2017.00, 23290.0), (2018.00, 24022.0), (2019.00, 24386.0), (2020.00, 24774.0), (2021.00, 25023.0), (2022.00, 25204.0), (2023.00, 25374.0), (2024.00, 25508.0), (2025.00, 25622.0), (2026.00, 25738.0), (2027.00, 25879.0), (2028.00, 25979.0), (2029.00, 26045.0), (2030.00, 26063.0), (2031.00, 26151.0), (2032.00, 26198.0), (2033.00, 26293.0), (2034.00, 26463.0), (2035.00, 26728.0), (2036.00, 27120.0), (2037.00, 27568.0), (2038.00, 28054.0), (2039.00, 28518.0), (2040.00, 28944.0), (2041.00, 29198.0), (2042.00, 29611.0), (2043.00, 29982.0), (2044.00, 30337.0), (2045.00, 30682.0), (2046.00, 31093.0), (2047.00, 31380.0), (2048.00, 31675.0), (2049.00, 31955.0), (2050.00, 32218.0), (2051.00, 32454.0), (2052.00, 32664.0), (2053.00, 32845.0), (2054.00, 33001.0), (2055.00, 33135.0), (2056.00, 33247.0), (2057.00, 33354.0), (2058.00, 33467.0), (2059.00, 33594.0), (2060.00, 33735.0), (2061.00, 33893.0)

UNITS: persons/year

NIM_Arrivals_M[M, A39] = GRAPH(TIME)

Points: (2011.00, 15620.0), (2012.00, 14640.0), (2013.00, 15300.0), (2014.00, 14700.0), (2015.00, 15530.0), (2016.00, 16600.0), (2017.00, 17130.0), (2018.00, 17422.0), (2019.00, 18020.0), (2020.00, 18586.0), (2021.00, 19022.0), (2022.00, 19358.0), (2023.00, 19600.0), (2024.00, 19877.0), (2025.00, 20177.0), (2026.00, 20364.0), (2027.00, 20499.0), (2028.00, 20630.0), (2029.00, 20738.0), (2030.00, 20829.0), (2031.00, 20916.0), (2032.00, 21025.0), (2033.00, 21098.0), (2034.00, 21152.0), (2035.00, 21166.0), (2036.00, 21236.0), (2037.00, 21271.0), (2038.00, 21344.0), (2039.00, 21478.0), (2040.00, 21687.0), (2041.00, 21992.0), (2042.00, 22334.0), (2043.00, 22708.0), (2044.00, 23062.0), (2045.00, 23392.0), (2046.00, 23582.0), (2047.00, 23906.0), (2048.00, 24189.0), (2049.00, 24464.0), (2050.00, 24728.0), (2051.00, 25043.0), (2052.00, 25266.0), (2053.00, 25491.0), (2054.00, 25708.0), (2055.00, 25909.0), (2056.00, 26090.0), (2057.00, 26252.0), (2058.00, 26391.0), (2059.00, 26511.0), (2060.00, 26612.0), (2061.00, 26697.0)

UNITS: persons/year

NIM_Arrivals_M[M, A44] = GRAPH(TIME)

Points: (2011.00, 12210.0), (2012.00, 11620.0), (2013.00, 12500.0), (2014.00, 11990.0), (2015.00, 12090.0), (2016.00, 12590.0), (2017.00, 12530.0), (2018.00, 11605.0), (2019.00, 11736.0), (2020.00, 11958.0), (2021.00, 12326.0), (2022.00, 12752.0), (2023.00, 13247.0), (2024.00, 13677.0), (2025.00, 14086.0), (2026.00, 14403.0), (2027.00, 14644.0), (2028.00, 14822.0), (2029.00, 15021.0), (2030.00, 15237.0), (2031.00, 15378.0), (2032.00, 15477.0), (2033.00, 15571.0), (2034.00, 15646.0), (2035.00, 15716.0), (2036.00, 15783.0), (2037.00, 15865.0), (2038.00, 15917.0), (2039.00, 15954.0), (2040.00, 15967.0), (2041.00, 16017.0), (2042.00, 16044.0), (2043.00, 16099.0), (2044.00, 16195.0), (2045.00, 16346.0), (2046.00, 16569.0), (2047.00, 16821.0), (2048.00, 17093.0), (2049.00, 17348.0), (2050.00, 17588.0), (2051.00, 17727.0), (2052.00, 17962.0), (2053.00, 18167.0), (2054.00, 18369.0), (2055.00, 18563.0), (2056.00, 18791.0), (2057.00, 18953.0), (2058.00, 19118.0), (2059.00, 19274.0), (2060.00, 19422.0), (2061.00, 19554.0)

UNITS: persons/year

NIM_Arrivals_M[M, A49] = GRAPH(TIME)

Points: (2011.00, 9080.0), (2012.00, 8390.0), (2013.00, 9000.0), (2014.00, 8670.0), (2015.00, 8840.0), (2016.00, 9870.0), (2017.00, 10210.0), (2018.00, 9518.0), (2019.00, 9574.0), (2020.00, 9568.0), (2021.00, 9457.0), (2022.00, 9380.0), (2023.00, 9383.0), (2024.00, 9480.0), (2025.00, 9648.0), (2026.00, 9929.0), (2027.00, 10257.0), (2028.00, 10643.0), (2029.00, 10984.0), (2030.00, 11311.0), (2031.00, 11565.0), (2032.00, 11761.0), (2033.00, 11904.0), (2034.00, 12064.0), (2035.00, 12233.0), (2036.00, 12343.0), (2037.00, 12426.0), (2038.00, 12506.0), (2039.00, 12570.0), (2040.00, 12619.0), (2041.00, 12671.0), (2042.00, 12739.0), (2043.00, 12784.0), (2044.00, 12818.0), (2045.00, 12826.0), (2046.00, 12866.0), (2047.00, 12889.0), (2048.00, 12931.0), (2049.00, 13008.0), (2050.00, 13123.0), (2051.00, 13293.0), (2052.00, 13489.0), (2053.00, 13705.0), (2054.00, 13908.0), (2055.00, 14100.0), (2056.00, 14214.0), (2057.00, 14398.0), (2058.00, 14559.0), (2059.00, 14717.0), (2060.00, 14869.0), (2061.00, 15054.0)

UNITS: persons/year

NIM_Arrivals_M[M, A54] = GRAPH(TIME)

Points: (2011.00, 7390.0), (2012.00, 7260.0), (2013.00, 7660.0), (2014.00, 7140.0), (2015.00, 7250.0), (2016.00, 7820.0), (2017.00, 8150.0), (2018.00, 7649.0), (2019.00, 7732.0), (2020.00, 7892.0), (2021.00, 8157.0), (2022.00, 8385.0), (2023.00, 8521.0), (2024.00, 8574.0), (2025.00, 8576.0), (2026.00, 8478.0), (2027.00, 8409.0), (2028.00, 8410.0), (2029.00, 8494.0), (2030.00, 8639.0), (2031.00, 8884.0), (2032.00, 9171.0), (2033.00, 9512.0), (2034.00, 9811.0), (2035.00, 10101.0), (2036.00, 10327.0), (2037.00, 10505.0), (2038.00, 10631.0), (2039.00, 10773.0), (2040.00, 10922.0), (2041.00, 11020.0), (2042.00, 11096.0), (2043.00, 11169.0), (2044.00, 11227.0), (2045.00, 11270.0), (2046.00, 11315.0), (2047.00, 11375.0), (2048.00, 11419.0), (2049.00, 11449.0), (2050.00, 11456.0), (2051.00, 11493.0), (2052.00, 11513.0), (2053.00, 11552.0), (2054.00, 11620.0), (2055.00, 11722.0), (2056.00, 11869.0), (2057.00, 12042.0), (2058.00, 12234.0), (2059.00, 12416.0), (2060.00, 12587.0), (2061.00, 12688.0)

UNITS: persons/year

NIM_Arrivals_M[M, A59] = GRAPH(TIME)

Points: (2011.00, 6140.0), (2012.00, 6060.0), (2013.00, 6410.0), (2014.00, 6010.0), (2015.00, 6210.0), (2016.00, 6950.0), (2017.00, 7570.0), (2018.00, 7323.0), (2019.00, 7389.0), (2020.00, 7401.0), (2021.00, 7353.0), (2022.00, 7313.0), (2023.00, 7318.0), (2024.00, 7400.0), (2025.00, 7555.0), (2026.00, 7807.0), (2027.00, 8032.0), (2028.00, 8165.0), (2029.00, 8216.0), (2030.00, 8217.0), (2031.00, 8126.0), (2032.00, 8062.0), (2033.00, 8066.0), (2034.00, 8145.0), (2035.00, 8286.0), (2036.00, 8521.0), (2037.00, 8798.0), (2038.00, 9121.0), (2039.00, 9411.0), (2040.00, 9687.0), (2041.00, 9903.0), (2042.00, 10073.0), (2043.00, 10195.0), (2044.00, 10332.0), (2045.00, 10476.0), (2046.00, 10575.0), (2047.00, 10649.0), (2048.00, 10719.0), (2049.00, 10775.0), (2050.00, 10819.0), (2051.00, 10867.0), (2052.00, 10926.0), (2053.00, 10970.0), (2054.00, 10999.0), (2055.00, 11009.0), (2056.00, 11046.0), (2057.00, 11069.0), (2058.00, 11108.0), (2059.00, 11172.0), (2060.00, 11273.0), (2061.00, 11415.0)

UNITS: persons/year

NIM_Arrivals_M[M, A64] = GRAPH(TIME)

Points: (2011.00, 5140.0), (2012.00, 5040.0), (2013.00, 5480.0), (2014.00, 5080.0), (2015.00, 5400.0), (2016.00, 6100.0), (2017.00, 6370.0), (2018.00, 5858.0), (2019.00, 5994.0), (2020.00, 6117.0), (2021.00, 6255.0), (2022.00, 6388.0), (2023.00, 6490.0), (2024.00, 6548.0), (2025.00, 6559.0), (2026.00, 6521.0), (2027.00, 6491.0), (2028.00, 6505.0), (2029.00, 6585.0), (2030.00, 6726.0), (2031.00, 6956.0), (2032.00, 7153.0), (2033.00, 7268.0), (2034.00, 7308.0), (2035.00, 7309.0), (2036.00, 7234.0), (2037.00, 7183.0), (2038.00, 7191.0), (2039.00, 7268.0), (2040.00, 7400.0), (2041.00, 7616.0), (2042.00, 7863.0), (2043.00, 8153.0), (2044.00, 8408.0), (2045.00, 8655.0), (2046.00, 8848.0), (2047.00, 8997.0), (2048.00, 9109.0), (2049.00, 9234.0), (2050.00, 9367.0), (2051.00, 9453.0), (2052.00, 9518.0), (2053.00, 9581.0), (2054.00, 9633.0), (2055.00, 9676.0), (2056.00, 9720.0), (2057.00, 9775.0), (2058.00, 9813.0), (2059.00, 9843.0), (2060.00, 9853.0), (2061.00, 9889.0)

UNITS: persons/year

NIM_Arrivals_M[M, A69] = GRAPH(TIME)

Points: (2011.00, 3110.0), (2012.00, 3470.0), (2013.00, 4010.0), (2014.00, 3820.0), (2015.00, 4290.0), (2016.00, 4930.0), (2017.00, 4950.0), (2018.00, 4315.0), (2019.00, 4383.0), (2020.00, 4462.0), (2021.00, 4565.0), (2022.00, 4663.0), (2023.00, 4761.0), (2024.00, 4873.0), (2025.00, 4976.0), (2026.00, 5095.0), (2027.00, 5206.0), (2028.00, 5291.0), (2029.00, 5342.0), (2030.00, 5348.0), (2031.00, 5317.0), (2032.00, 5293.0), (2033.00, 5309.0), (2034.00, 5383.0), (2035.00, 5504.0), (2036.00, 5696.0), (2037.00, 5858.0), (2038.00, 5949.0), (2039.00, 5977.0), (2040.00, 5972.0), (2041.00, 5913.0), (2042.00, 5876.0), (2043.00, 5886.0), (2044.00, 5954.0), (2045.00, 6065.0), (2046.00, 6246.0), (2047.00, 6454.0), (2048.00, 6693.0), (2049.00, 6902.0), (2050.00, 7102.0), (2051.00, 7257.0), (2052.00, 7379.0), (2053.00, 7470.0), (2054.00, 7574.0), (2055.00, 7684.0), (2056.00, 7758.0), (2057.00, 7810.0), (2058.00, 7861.0), (2059.00, 7904.0), (2060.00, 7946.0), (2061.00, 7986.0)

UNITS: persons/year

NIM_Arrivals_M[M, A74] = GRAPH(TIME)

Points: (2011.00, 1710.0), (2012.00, 1850.0), (2013.00, 2160.0), (2014.00, 2030.0), (2015.00, 2400.0), (2016.00, 2800.0), (2017.00, 3210.0), (2018.00, 2854.0), (2019.00, 2953.0), (2020.00, 3048.0), (2021.00, 3120.0), (2022.00, 3122.0), (2023.00, 3171.0), (2024.00, 3225.0), (2025.00, 3289.0), (2026.00, 3368.0), (2027.00, 3444.0), (2028.00, 3519.0), (2029.00, 3609.0), (2030.00, 3688.0), (2031.00, 3778.0), (2032.00, 3865.0), (2033.00, 3930.0), (2034.00, 3971.0), (2035.00, 3978.0), (2036.00, 3958.0), (2037.00, 3945.0), (2038.00, 3954.0), (2039.00, 4009.0), (2040.00, 4100.0), (2041.00, 4244.0), (2042.00, 4367.0), (2043.00, 4434.0), (2044.00, 4461.0), (2045.00, 4459.0), (2046.00, 4413.0), (2047.00, 4388.0), (2048.00, 4396.0), (2049.00, 4445.0), (2050.00, 4528.0), (2051.00, 4662.0), (2052.00, 4818.0), (2053.00, 4995.0), (2054.00, 5155.0), (2055.00, 5303.0), (2056.00, 5423.0), (2057.00, 5512.0), (2058.00, 5583.0), (2059.00, 5661.0), (2060.00, 5744.0), (2061.00, 5800.0)

UNITS: persons/year

NIM_Arrivals_M[M, A79] = GRAPH(TIME)

Points: (2011.00, 902.0), (2012.00, 941.0), (2013.00, 1089.0), (2014.00, 1032.0), (2015.00, 1107.0), (2016.00, 1207.0), (2017.00, 1268.0), (2018.00, 1524.0), (2019.00, 1603.0), (2020.00, 1686.0), (2021.00, 1773.0), (2022.00, 1914.0), (2023.00, 2031.0), (2024.00, 2109.0), (2025.00, 2179.0), (2026.00, 2240.0), (2027.00, 2244.0), (2028.00, 2280.0), (2029.00, 2323.0), (2030.00, 2373.0), (2031.00, 2433.0), (2032.00, 2490.0), (2033.00, 2546.0), (2034.00, 2614.0), (2035.00, 2675.0), (2036.00, 2743.0), (2037.00, 2806.0), (2038.00, 2856.0), (2039.00, 2889.0), (2040.00, 2897.0), (2041.00, 2884.0), (2042.00, 2872.0), (2043.00, 2880.0), (2044.00, 2917.0), (2045.00, 2984.0), (2046.00, 3088.0), (2047.00, 3179.0), (2048.00, 3235.0), (2049.00, 3258.0), (2050.00, 3262.0), (2051.00, 3232.0), (2052.00, 3210.0), (2053.00, 3215.0), (2054.00, 3251.0), (2055.00, 3310.0), (2056.00, 3407.0), (2057.00, 3521.0), (2058.00, 3652.0), (2059.00, 3770.0), (2060.00, 3882.0), (2061.00, 3971.0)

UNITS: persons/year

NIM_Arrivals_M[M, A84] = GRAPH(TIME)

Points: (2011.00, 671.0), (2012.00, 700.0), (2013.00, 810.0), (2014.00, 768.0), (2015.00, 823.0), (2016.00, 898.0), (2017.00, 943.0), (2018.00, 933.0), (2019.00, 980.0), (2020.00, 1033.0), (2021.00, 1080.0), (2022.00, 1132.0), (2023.00, 1177.0), (2024.00, 1245.0), (2025.00, 1312.0), (2026.00, 1386.0), (2027.00, 1503.0), (2028.00, 1597.0), (2029.00, 1657.0), (2030.00, 1710.0), (2031.00, 1762.0), (2032.00, 1770.0), (2033.00, 1803.0), (2034.00, 1839.0), (2035.00, 1880.0), (2036.00, 1928.0), (2037.00, 1976.0), (2038.00, 2023.0), (2039.00, 2080.0), (2040.00, 2129.0), (2041.00, 2186.0), (2042.00, 2238.0), (2043.00, 2278.0), (2044.00, 2305.0), (2045.00, 2311.0), (2046.00, 2303.0), (2047.00, 2296.0), (2048.00, 2307.0), (2049.00, 2343.0), (2050.00, 2396.0), (2051.00, 2484.0), (2052.00, 2558.0), (2053.00, 2601.0), (2054.00, 2619.0), (2055.00, 2624.0), (2056.00, 2601.0), (2057.00, 2589.0), (2058.00, 2594.0), (2059.00, 2626.0), (2060.00, 2678.0), (2061.00, 2761.0)

UNITS: persons/year

NIM_Arrivals_M[M, A89] = GRAPH(TIME)

Points: (2011.00, 359.0), (2012.00, 375.0), (2013.00, 434.0), (2014.00, 411.0), (2015.00, 441.0), (2016.00, 481.0), (2017.00, 505.0), (2018.00, 519.0), (2019.00, 526.0), (2020.00, 536.0), (2021.00, 552.0), (2022.00, 579.0), (2023.00, 608.0), (2024.00, 643.0), (2025.00, 679.0), (2026.00, 712.0), (2027.00, 751.0), (2028.00, 783.0), (2029.00, 829.0), (2030.00, 877.0), (2031.00, 929.0), (2032.00, 1009.0), (2033.00, 1076.0), (2034.00, 1125.0), (2035.00, 1167.0), (2036.00, 1202.0), (2037.00, 1209.0), (2038.00, 1235.0), (2039.00, 1263.0), (2040.00, 1295.0), (2041.00, 1332.0), (2042.00, 1369.0), (2043.00, 1407.0), (2044.00, 1449.0), (2045.00, 1485.0), (2046.00, 1528.0), (2047.00, 1570.0), (2048.00, 1604.0), (2049.00, 1629.0), (2050.00, 1639.0), (2051.00, 1638.0), (2052.00, 1635.0), (2053.00, 1644.0), (2054.00, 1669.0), (2055.00, 1710.0), (2056.00, 1775.0), (2057.00, 1837.0), (2058.00, 1877.0), (2059.00, 1899.0), (2060.00, 1906.0), (2061.00, 1894.0)

UNITS: persons/year

NIM_Arrivals_M[M, A94] = GRAPH(TIME)

Points: (2011.00, 113.0), (2012.00, 118.0), (2013.00, 136.0), (2014.00, 129.0), (2015.00, 139.0), (2016.00, 151.0), (2017.00, 159.0), (2018.00, 224.0), (2019.00, 233.0), (2020.00, 245.0), (2021.00, 254.0), (2022.00, 259.0), (2023.00, 264.0), (2024.00, 269.0), (2025.00, 275.0), (2026.00, 287.0), (2027.00, 304.0), (2028.00, 321.0), (2029.00, 340.0), (2030.00, 360.0), (2031.00, 377.0), (2032.00, 400.0), (2033.00, 420.0), (2034.00, 447.0), (2035.00, 477.0), (2036.00, 506.0), (2037.00, 558.0), (2038.00, 594.0), (2039.00, 614.0), (2040.00, 639.0), (2041.00, 657.0), (2042.00, 666.0), (2043.00, 685.0), (2044.00, 702.0), (2045.00, 723.0), (2046.00, 748.0), (2047.00, 771.0), (2048.00, 794.0), (2049.00, 820.0), (2050.00, 846.0), (2051.00, 875.0), (2052.00, 901.0), (2053.00, 920.0), (2054.00, 935.0), (2055.00, 941.0), (2056.00, 941.0), (2057.00, 944.0), (2058.00, 954.0), (2059.00, 978.0), (2060.00, 1008.0), (2061.00, 1053.0)

UNITS: persons/year

NIM_Arrivals_M[M, A99] = GRAPH(TIME)

Points: (2011.00, 21.0), (2012.00, 21.0), (2013.00, 25.0), (2014.00, 24.0), (2015.00, 25.0), (2016.00, 28.0), (2017.00, 29.0), (2018.00, 51.0), (2019.00, 56.0), (2020.00, 60.0), (2021.00, 64.0), (2022.00, 67.0), (2023.00, 71.0), (2024.00, 73.0), (2025.00, 78.0), (2026.00, 80.0), (2027.00, 82.0), (2028.00, 84.0), (2029.00, 86.0), (2030.00, 87.0), (2031.00, 92.0), (2032.00, 98.0), (2033.00, 105.0), (2034.00, 110.0), (2035.00, 118.0), (2036.00, 126.0), (2037.00, 135.0), (2038.00, 142.0), (2039.00, 152.0), (2040.00, 164.0), (2041.00, 175.0), (2042.00, 195.0), (2043.00, 209.0), (2044.00, 219.0), (2045.00, 227.0), (2046.00, 235.0), (2047.00, 241.0), (2048.00, 250.0), (2049.00, 259.0), (2050.00, 268.0), (2051.00, 279.0), (2052.00, 290.0), (2053.00, 302.0), (2054.00, 313.0), (2055.00, 325.0), (2056.00, 337.0), (2057.00, 350.0), (2058.00, 362.0), (2059.00, 369.0), (2060.00, 373.0), (2061.00, 376.0)

UNITS: persons/year

NIM_Arrivals_M[M, A104] = GRAPH(TIME)

Points: (2011.00, 2.0), (2012.00, 2.0), (2013.00, 2.0), (2014.00, 2.0), (2015.00, 2.0), (2016.00, 3.0), (2017.00, 3.0), (2018.00, 7.0), (2019.00, 7.0), (2020.00, 8.0), (2021.00, 10.0), (2022.00, 11.0), (2023.00, 12.0), (2024.00, 13.0), (2025.00, 14.0), (2026.00, 15.0), (2027.00, 16.0), (2028.00, 17.0), (2029.00, 18.0), (2030.00, 19.0), (2031.00, 20.0), (2032.00, 21.0), (2033.00, 21.0), (2034.00, 22.0), (2035.00, 23.0), (2036.00, 24.0), (2037.00, 26.0), (2038.00, 28.0), (2039.00, 29.0), (2040.00, 32.0), (2041.00, 34.0), (2042.00, 36.0), (2043.00, 39.0), (2044.00, 42.0), (2045.00, 46.0), (2046.00, 49.0), (2047.00, 55.0), (2048.00, 59.0), (2049.00, 63.0), (2050.00, 66.0), (2051.00, 70.0), (2052.00, 73.0), (2053.00, 77.0), (2054.00, 80.0), (2055.00, 84.0), (2056.00, 89.0), (2057.00, 93.0), (2058.00, 98.0), (2059.00, 102.0), (2060.00, 107.0), (2061.00, 113.0)

UNITS: persons/year

NIM_Arrivals_M[F, A4] = GRAPH(TIME)

Points: (2011.00, 13960.0), (2012.00, 12760.0), (2013.00, 13070.0), (2014.00, 12660.0), (2015.00, 14180.0), (2016.00, 14990.0), (2017.00, 14890.0), (2018.00, 14283.0), (2019.00, 14469.0), (2020.00, 14641.0), (2021.00, 14797.0), (2022.00, 14934.0), (2023.00, 15051.0), (2024.00, 15154.0), (2025.00, 15242.0), (2026.00, 15314.0), (2027.00, 15380.0), (2028.00, 15449.0), (2029.00, 15526.0), (2030.00, 15615.0), (2031.00, 15717.0), (2032.00, 15828.0), (2033.00, 15942.0), (2034.00, 16062.0), (2035.00, 16192.0), (2036.00, 16331.0), (2037.00, 16479.0), (2038.00, 16638.0), (2039.00, 16806.0), (2040.00, 16982.0), (2041.00, 17164.0), (2042.00, 17352.0), (2043.00, 17542.0), (2044.00, 17734.0), (2045.00, 17922.0), (2046.00, 18108.0), (2047.00, 18289.0), (2048.00, 18465.0), (2049.00, 18634.0), (2050.00, 18794.0), (2051.00, 18947.0), (2052.00, 19090.0), (2053.00, 19226.0), (2054.00, 19353.0), (2055.00, 19474.0), (2056.00, 19588.0), (2057.00, 19697.0), (2058.00, 19803.0), (2059.00, 19906.0), (2060.00, 20006.0), (2061.00, 20108.0)

UNITS: persons/year

NIM_Arrivals_M[F, A9] = GRAPH(TIME)

Points: (2011.00, 10430.0), (2012.00, 9540.0), (2013.00, 10480.0), (2014.00, 10080.0), (2015.00, 11030.0), (2016.00, 11750.0), (2017.00, 11820.0), (2018.00, 11212.0), (2019.00, 11404.0), (2020.00, 11593.0), (2021.00, 11810.0), (2022.00, 11967.0), (2023.00, 12121.0), (2024.00, 12266.0), (2025.00, 12402.0), (2026.00, 12523.0), (2027.00, 12633.0), (2028.00, 12724.0), (2029.00, 12804.0), (2030.00, 12872.0), (2031.00, 12929.0), (2032.00, 12984.0), (2033.00, 13042.0), (2034.00, 13108.0), (2035.00, 13182.0), (2036.00, 13265.0), (2037.00, 13355.0), (2038.00, 13448.0), (2039.00, 13547.0), (2040.00, 13652.0), (2041.00, 13766.0), (2042.00, 13888.0), (2043.00, 14017.0), (2044.00, 14154.0), (2045.00, 14297.0), (2046.00, 14445.0), (2047.00, 14596.0), (2048.00, 14749.0), (2049.00, 14903.0), (2050.00, 15055.0), (2051.00, 15204.0), (2052.00, 15350.0), (2053.00, 15490.0), (2054.00, 15625.0), (2055.00, 15752.0), (2056.00, 15873.0), (2057.00, 15988.0), (2058.00, 16096.0), (2059.00, 16198.0), (2060.00, 16294.0), (2061.00, 16386.0)

UNITS: persons/year

NIM_Arrivals_M[F, A14] = GRAPH(TIME)

Points: (2011.00, 8260.0), (2012.00, 7330.0), (2013.00, 8100.0), (2014.00, 7400.0), (2015.00, 7910.0), (2016.00, 8760.0), (2017.00, 8770.0), (2018.00, 8275.0), (2019.00, 8474.0), (2020.00, 8659.0), (2021.00, 8764.0), (2022.00, 8944.0), (2023.00, 9093.0), (2024.00, 9241.0), (2025.00, 9386.0), (2026.00, 9556.0), (2027.00, 9675.0), (2028.00, 9794.0), (2029.00, 9907.0), (2030.00, 10010.0), (2031.00, 10105.0), (2032.00, 10189.0), (2033.00, 10260.0), (2034.00, 10324.0), (2035.00, 10375.0), (2036.00, 10420.0), (2037.00, 10460.0), (2038.00, 10507.0), (2039.00, 10557.0), (2040.00, 10614.0), (2041.00, 10677.0), (2042.00, 10746.0), (2043.00, 10819.0), (2044.00, 10895.0), (2045.00, 10978.0), (2046.00, 11066.0), (2047.00, 11160.0), (2048.00, 11260.0), (2049.00, 11366.0), (2050.00, 11477.0), (2051.00, 11591.0), (2052.00, 11709.0), (2053.00, 11828.0), (2054.00, 11948.0), (2055.00, 12065.0), (2056.00, 12181.0), (2057.00, 12294.0), (2058.00, 12403.0), (2059.00, 12507.0), (2060.00, 12607.0), (2061.00, 12700.0)

UNITS: persons/year

NIM_Arrivals_M[F, A19] = GRAPH(TIME)

Points: (2011.00, 12730.0), (2012.00, 11850.0), (2013.00, 12750.0), (2014.00, 11510.0), (2015.00, 11560.0), (2016.00, 11910.0), (2017.00, 11990.0), (2018.00, 12209.0), (2019.00, 12281.0), (2020.00, 12360.0), (2021.00, 12501.0), (2022.00, 12705.0), (2023.00, 12968.0), (2024.00, 13294.0), (2025.00, 13593.0), (2026.00, 13768.0), (2027.00, 14027.0), (2028.00, 14235.0), (2029.00, 14398.0), (2030.00, 14599.0), (2031.00, 14901.0), (2032.00, 15075.0), (2033.00, 15242.0), (2034.00, 15405.0), (2035.00, 15562.0), (2036.00, 15704.0), (2037.00, 15833.0), (2038.00, 15945.0), (2039.00, 16041.0), (2040.00, 16121.0), (2041.00, 16187.0), (2042.00, 16246.0), (2043.00, 16305.0), (2044.00, 16364.0), (2045.00, 16429.0), (2046.00, 16514.0), (2047.00, 16610.0), (2048.00, 16706.0), (2049.00, 16810.0), (2050.00, 16919.0), (2051.00, 17036.0), (2052.00, 17163.0), (2053.00, 17299.0), (2054.00, 17441.0), (2055.00, 17594.0), (2056.00, 17752.0), (2057.00, 17915.0), (2058.00, 18083.0), (2059.00, 18252.0), (2060.00, 18422.0), (2061.00, 18588.0)

UNITS: persons/year

NIM_Arrivals_M[F, A24] = GRAPH(TIME)

Points: (2011.00, 24200.0), (2012.00, 25110.0), (2013.00, 25430.0), (2014.00, 23760.0), (2015.00, 23010.0), (2016.00, 24220.0), (2017.00, 24350.0), (2018.00, 26373.0), (2019.00, 26527.0), (2020.00, 26591.0), (2021.00, 26710.0), (2022.00, 26786.0), (2023.00, 26922.0), (2024.00, 27057.0), (2025.00, 27276.0), (2026.00, 27666.0), (2027.00, 28140.0), (2028.00, 28714.0), (2029.00, 29305.0), (2030.00, 29853.0), (2031.00, 30185.0), (2032.00, 30668.0), (2033.00, 31078.0), (2034.00, 31491.0), (2035.00, 31910.0), (2036.00, 32422.0), (2037.00, 32772.0), (2038.00, 33113.0), (2039.00, 33441.0), (2040.00, 33749.0), (2041.00, 34028.0), (2042.00, 34276.0), (2043.00, 34489.0), (2044.00, 34674.0), (2045.00, 34829.0), (2046.00, 34959.0), (2047.00, 35079.0), (2048.00, 35202.0), (2049.00, 35337.0), (2050.00, 35491.0), (2051.00, 35669.0), (2052.00, 35863.0), (2053.00, 36064.0), (2054.00, 36278.0), (2055.00, 36506.0), (2056.00, 36753.0), (2057.00, 37015.0), (2058.00, 37296.0), (2059.00, 37593.0), (2060.00, 37904.0), (2061.00, 38228.0)

UNITS: persons/year

NIM_Arrivals_M[F, A29] = GRAPH(TIME)

Points: (2011.00, 25540.0), (2012.00, 26140.0), (2013.00, 27210.0), (2014.00, 25850.0), (2015.00, 26540.0), (2016.00, 28290.0), (2017.00, 28450.0), (2018.00, 28080.0), (2019.00, 28217.0), (2020.00, 28344.0), (2021.00, 28422.0), (2022.00, 28585.0), (2023.00, 28699.0), (2024.00, 28834.0), (2025.00, 28910.0), (2026.00, 29031.0), (2027.00, 29100.0), (2028.00, 29232.0), (2029.00, 29391.0), (2030.00, 29653.0), (2031.00, 30104.0), (2032.00, 30590.0), (2033.00, 31156.0), (2034.00, 31715.0), (2035.00, 32233.0), (2036.00, 32543.0), (2037.00, 33028.0), (2038.00, 33457.0), (2039.00, 33882.0), (2040.00, 34292.0), (2041.00, 34771.0), (2042.00, 35123.0), (2043.00, 35464.0), (2044.00, 35789.0), (2045.00, 36091.0), (2046.00, 36365.0), (2047.00, 36606.0), (2048.00, 36816.0), (2049.00, 36996.0), (2050.00, 37149.0), (2051.00, 37278.0), (2052.00, 37401.0), (2053.00, 37533.0), (2054.00, 37678.0), (2055.00, 37838.0), (2056.00, 38022.0), (2057.00, 38220.0), (2058.00, 38426.0), (2059.00, 38645.0), (2060.00, 38879.0), (2061.00, 39131.0)

UNITS: persons/year

NIM_Arrivals_M[F, A34] = GRAPH(TIME)

Points: (2011.00, 18390.0), (2012.00, 17710.0), (2013.00, 18900.0), (2014.00, 18820.0), (2015.00, 20650.0), (2016.00, 22200.0), (2017.00, 22350.0), (2018.00, 21881.0), (2019.00, 22215.0), (2020.00, 22560.0), (2021.00, 22846.0), (2022.00, 23044.0), (2023.00, 23193.0), (2024.00, 23290.0), (2025.00, 23381.0), (2026.00, 23442.0), (2027.00, 23565.0), (2028.00, 23648.0), (2029.00, 23748.0), (2030.00, 23805.0), (2031.00, 23896.0), (2032.00, 23947.0), (2033.00, 24048.0), (2034.00, 24170.0), (2035.00, 24369.0), (2036.00, 24711.0), (2037.00, 25078.0), (2038.00, 25506.0), (2039.00, 25925.0), (2040.00, 26317.0), (2041.00, 26548.0), (2042.00, 26913.0), (2043.00, 27238.0), (2044.00, 27557.0), (2045.00, 27866.0), (2046.00, 28224.0), (2047.00, 28492.0), (2048.00, 28748.0), (2049.00, 28992.0), (2050.00, 29221.0), (2051.00, 29427.0), (2052.00, 29609.0), (2053.00, 29769.0), (2054.00, 29903.0), (2055.00, 30019.0), (2056.00, 30115.0), (2057.00, 30208.0), (2058.00, 30309.0), (2059.00, 30420.0), (2060.00, 30541.0), (2061.00, 30681.0)

UNITS: persons/year

NIM_Arrivals_M[F, A39] = GRAPH(TIME)

Points: (2011.00, 14300.0), (2012.00, 12580.0), (2013.00, 13400.0), (2014.00, 12800.0), (2015.00, 13890.0), (2016.00, 15350.0), (2017.00, 15660.0), (2018.00, 14957.0), (2019.00, 15463.0), (2020.00, 15942.0), (2021.00, 16303.0), (2022.00, 16591.0), (2023.00, 16813.0), (2024.00, 17053.0), (2025.00, 17302.0), (2026.00, 17507.0), (2027.00, 17645.0), (2028.00, 17751.0), (2029.00, 17821.0), (2030.00, 17887.0), (2031.00, 17931.0), (2032.00, 18019.0), (2033.00, 18077.0), (2034.00, 18151.0), (2035.00, 18193.0), (2036.00, 18257.0), (2037.00, 18293.0), (2038.00, 18366.0), (2039.00, 18455.0), (2040.00, 18600.0), (2041.00, 18850.0), (2042.00, 19116.0), (2043.00, 19423.0), (2044.00, 19726.0), (2045.00, 20007.0), (2046.00, 20172.0), (2047.00, 20438.0), (2048.00, 20672.0), (2049.00, 20903.0), (2050.00, 21124.0), (2051.00, 21385.0), (2052.00, 21576.0), (2053.00, 21761.0), (2054.00, 21938.0), (2055.00, 22104.0), (2056.00, 22253.0), (2057.00, 22385.0), (2058.00, 22497.0), (2059.00, 22595.0), (2060.00, 22678.0), (2061.00, 22748.0)

UNITS: persons/year

NIM_Arrivals_M[F, A44] = GRAPH(TIME)

Points: (2011.00, 10350.0), (2012.00, 9440.0), (2013.00, 10600.0), (2014.00, 9630.0), (2015.00, 10240.0), (2016.00, 10980.0), (2017.00, 11120.0), (2018.00, 9827.0), (2019.00, 9915.0), (2020.00, 10089.0), (2021.00, 10372.0), (2022.00, 10697.0), (2023.00, 11081.0), (2024.00, 11437.0), (2025.00, 11777.0), (2026.00, 12035.0), (2027.00, 12241.0), (2028.00, 12397.0), (2029.00, 12569.0), (2030.00, 12742.0), (2031.00, 12888.0), (2032.00, 12990.0), (2033.00, 13066.0), (2034.00, 13116.0), (2035.00, 13161.0), (2036.00, 13193.0), (2037.00, 13257.0), (2038.00, 13299.0), (2039.00, 13350.0), (2040.00, 13380.0), (2041.00, 13423.0), (2042.00, 13451.0), (2043.00, 13504.0), (2044.00, 13566.0), (2045.00, 13667.0), (2046.00, 13841.0), (2047.00, 14029.0), (2048.00, 14247.0), (2049.00, 14462.0), (2050.00, 14661.0), (2051.00, 14780.0), (2052.00, 14966.0), (2053.00, 15133.0), (2054.00, 15295.0), (2055.00, 15455.0), (2056.00, 15638.0), (2057.00, 15775.0), (2058.00, 15906.0), (2059.00, 16031.0), (2060.00, 16148.0), (2061.00, 16254.0)

UNITS: persons/year

NIM_Arrivals_M[F, A49] = GRAPH(TIME)

Points: (2011.00, 7700.0), (2012.00, 7240.0), (2013.00, 7690.0), (2014.00, 7170.0), (2015.00, 7610.0), (2016.00, 8620.0), (2017.00, 9070.0), (2018.00, 8477.0), (2019.00, 8513.0), (2020.00, 8502.0), (2021.00, 8382.0), (2022.00, 8291.0), (2023.00, 8278.0), (2024.00, 8345.0), (2025.00, 8482.0), (2026.00, 8705.0), (2027.00, 8968.0), (2028.00, 9280.0), (2029.00, 9573.0), (2030.00, 9856.0), (2031.00, 10070.0), (2032.00, 10244.0), (2033.00, 10374.0), (2034.00, 10517.0), (2035.00, 10657.0), (2036.00, 10778.0), (2037.00, 10864.0), (2038.00, 10933.0), (2039.00, 10977.0), (2040.00, 11013.0), (2041.00, 11037.0), (2042.00, 11091.0), (2043.00, 11128.0), (2044.00, 11171.0), (2045.00, 11196.0), (2046.00, 11234.0), (2047.00, 11254.0), (2048.00, 11297.0), (2049.00, 11350.0), (2050.00, 11430.0), (2051.00, 11568.0), (2052.00, 11721.0), (2053.00, 11900.0), (2054.00, 12076.0), (2055.00, 12244.0), (2056.00, 12342.0), (2057.00, 12494.0), (2058.00, 12629.0), (2059.00, 12764.0), (2060.00, 12894.0), (2061.00, 13049.0)

UNITS: persons/year

NIM_Arrivals_M[F, A54] = GRAPH(TIME)

Points: (2011.00, 7140.0), (2012.00, 7040.0), (2013.00, 7400.0), (2014.00, 6650.0), (2015.00, 6810.0), (2016.00, 7850.0), (2017.00, 8200.0), (2018.00, 7432.0), (2019.00, 7514.0), (2020.00, 7662.0), (2021.00, 7924.0), (2022.00, 8163.0), (2023.00, 8315.0), (2024.00, 8354.0), (2025.00, 8351.0), (2026.00, 8232.0), (2027.00, 8143.0), (2028.00, 8127.0), (2029.00, 8188.0), (2030.00, 8317.0), (2031.00, 8530.0), (2032.00, 8781.0), (2033.00, 9081.0), (2034.00, 9367.0), (2035.00, 9642.0), (2036.00, 9854.0), (2037.00, 10026.0), (2038.00, 10153.0), (2039.00, 10290.0), (2040.00, 10424.0), (2041.00, 10545.0), (2042.00, 10637.0), (2043.00, 10705.0), (2044.00, 10749.0), (2045.00, 10783.0), (2046.00, 10809.0), (2047.00, 10861.0), (2048.00, 10900.0), (2049.00, 10941.0), (2050.00, 10965.0), (2051.00, 11002.0), (2052.00, 11026.0), (2053.00, 11070.0), (2054.00, 11119.0), (2055.00, 11195.0), (2056.00, 11328.0), (2057.00, 11476.0), (2058.00, 11651.0), (2059.00, 11824.0), (2060.00, 11988.0), (2061.00, 12087.0)

UNITS: persons/year

NIM_Arrivals_M[F, A59] = GRAPH(TIME)

Points: (2011.00, 5980.0), (2012.00, 6080.0), (2013.00, 6410.0), (2014.00, 5930.0), (2015.00, 6380.0), (2016.00, 7380.0), (2017.00, 8040.0), (2018.00, 7269.0), (2019.00, 7340.0), (2020.00, 7351.0), (2021.00, 7307.0), (2022.00, 7270.0), (2023.00, 7269.0), (2024.00, 7353.0), (2025.00, 7498.0), (2026.00, 7757.0), (2027.00, 7990.0), (2028.00, 8134.0), (2029.00, 8173.0), (2030.00, 8165.0), (2031.00, 8052.0), (2032.00, 7971.0), (2033.00, 7959.0), (2034.00, 8020.0), (2035.00, 8149.0), (2036.00, 8357.0), (2037.00, 8604.0), (2038.00, 8896.0), (2039.00, 9173.0), (2040.00, 9440.0), (2041.00, 9645.0), (2042.00, 9812.0), (2043.00, 9936.0), (2044.00, 10069.0), (2045.00, 10204.0), (2046.00, 10323.0), (2047.00, 10409.0), (2048.00, 10475.0), (2049.00, 10517.0), (2050.00, 10552.0), (2051.00, 10581.0), (2052.00, 10631.0), (2053.00, 10672.0), (2054.00, 10714.0), (2055.00, 10739.0), (2056.00, 10777.0), (2057.00, 10801.0), (2058.00, 10842.0), (2059.00, 10894.0), (2060.00, 10970.0), (2061.00, 11101.0)

UNITS: persons/year

NIM_Arrivals_M[F, A64] = GRAPH(TIME)

Points: (2011.00, 4730.0), (2012.00, 4720.0), (2013.00, 5310.0), (2014.00, 4960.0), (2015.00, 5210.0), (2016.00, 6270.0), (2017.00, 6430.0), (2018.00, 5644.0), (2019.00, 5782.0), (2020.00, 5910.0), (2021.00, 6057.0), (2022.00, 6185.0), (2023.00, 6286.0), (2024.00, 6347.0), (2025.00, 6360.0), (2026.00, 6320.0), (2027.00, 6290.0), (2028.00, 6292.0), (2029.00, 6367.0), (2030.00, 6492.0), (2031.00, 6718.0), (2032.00, 6916.0), (2033.00, 7038.0), (2034.00, 7070.0), (2035.00, 7065.0), (2036.00, 6970.0), (2037.00, 6901.0), (2038.00, 6893.0), (2039.00, 6947.0), (2040.00, 7058.0), (2041.00, 7239.0), (2042.00, 7449.0), (2043.00, 7703.0), (2044.00, 7939.0), (2045.00, 8169.0), (2046.00, 8345.0), (2047.00, 8489.0), (2048.00, 8596.0), (2049.00, 8711.0), (2050.00, 8827.0), (2051.00, 8928.0), (2052.00, 9002.0), (2053.00, 9059.0), (2054.00, 9098.0), (2055.00, 9130.0), (2056.00, 9152.0), (2057.00, 9198.0), (2058.00, 9232.0), (2059.00, 9271.0), (2060.00, 9292.0), (2061.00, 9325.0)

UNITS: persons/year

NIM_Arrivals_M[F, A69] = GRAPH(TIME)

Points: (2011.00, 2820.0), (2012.00, 3070.0), (2013.00, 3560.0), (2014.00, 3510.0), (2015.00, 3910.0), (2016.00, 4390.0), (2017.00, 4650.0), (2018.00, 3943.0), (2019.00, 4033.0), (2020.00, 4128.0), (2021.00, 4227.0), (2022.00, 4331.0), (2023.00, 4433.0), (2024.00, 4544.0), (2025.00, 4646.0), (2026.00, 4764.0), (2027.00, 4865.0), (2028.00, 4946.0), (2029.00, 4994.0), (2030.00, 5001.0), (2031.00, 4975.0), (2032.00, 4954.0), (2033.00, 4958.0), (2034.00, 5023.0), (2035.00, 5125.0), (2036.00, 5306.0), (2037.00, 5461.0), (2038.00, 5550.0), (2039.00, 5574.0), (2040.00, 5565.0), (2041.00, 5491.0), (2042.00, 5440.0), (2043.00, 5437.0), (2044.00, 5483.0), (2045.00, 5573.0), (2046.00, 5719.0), (2047.00, 5888.0), (2048.00, 6087.0), (2049.00, 6274.0), (2050.00, 6453.0), (2051.00, 6591.0), (2052.00, 6702.0), (2053.00, 6787.0), (2054.00, 6880.0), (2055.00, 6975.0), (2056.00, 7054.0), (2057.00, 7112.0), (2058.00, 7157.0), (2059.00, 7188.0), (2060.00, 7214.0), (2061.00, 7234.0)

UNITS: persons/year

NIM_Arrivals_M[F, A74] = GRAPH(TIME)

Points: (2011.00, 1540.0), (2012.00, 1690.0), (2013.00, 2000.0), (2014.00, 1840.0), (2015.00, 2210.0), (2016.00, 2550.0), (2017.00, 2830.0), (2018.00, 2576.0), (2019.00, 2666.0), (2020.00, 2755.0), (2021.00, 2848.0), (2022.00, 2863.0), (2023.00, 2923.0), (2024.00, 2990.0), (2025.00, 3061.0), (2026.00, 3137.0), (2027.00, 3216.0), (2028.00, 3292.0), (2029.00, 3376.0), (2030.00, 3454.0), (2031.00, 3544.0), (2032.00, 3621.0), (2033.00, 3681.0), (2034.00, 3717.0), (2035.00, 3724.0), (2036.00, 3703.0), (2037.00, 3687.0), (2038.00, 3691.0), (2039.00, 3739.0), (2040.00, 3812.0), (2041.00, 3946.0), (2042.00, 4060.0), (2043.00, 4126.0), (2044.00, 4143.0), (2045.00, 4141.0), (2046.00, 4088.0), (2047.00, 4050.0), (2048.00, 4045.0), (2049.00, 4077.0), (2050.00, 4143.0), (2051.00, 4249.0), (2052.00, 4372.0), (2053.00, 4519.0), (2054.00, 4655.0), (2055.00, 4787.0), (2056.00, 4887.0), (2057.00, 4969.0), (2058.00, 5032.0), (2059.00, 5100.0), (2060.00, 5170.0), (2061.00, 5227.0)

UNITS: persons/year

NIM_Arrivals_M[F, A79] = GRAPH(TIME)

Points: (2011.00, 1071.0), (2012.00, 1086.0), (2013.00, 1235.0), (2014.00, 1130.0), (2015.00, 1140.0), (2016.00, 1184.0), (2017.00, 1275.0), (2018.00, 1571.0), (2019.00, 1656.0), (2020.00, 1744.0), (2021.00, 1835.0), (2022.00, 1980.0), (2023.00, 2096.0), (2024.00, 2175.0), (2025.00, 2243.0), (2026.00, 2325.0), (2027.00, 2337.0), (2028.00, 2386.0), (2029.00, 2444.0), (2030.00, 2502.0), (2031.00, 2565.0), (2032.00, 2631.0), (2033.00, 2695.0), (2034.00, 2767.0), (2035.00, 2832.0), (2036.00, 2906.0), (2037.00, 2971.0), (2038.00, 3020.0), (2039.00, 3052.0), (2040.00, 3059.0), (2041.00, 3044.0), (2042.00, 3032.0), (2043.00, 3033.0), (2044.00, 3071.0), (2045.00, 3132.0), (2046.00, 3240.0), (2047.00, 3336.0), (2048.00, 3395.0), (2049.00, 3408.0), (2050.00, 3411.0), (2051.00, 3368.0), (2052.00, 3336.0), (2053.00, 3333.0), (2054.00, 3358.0), (2055.00, 3411.0), (2056.00, 3499.0), (2057.00, 3599.0), (2058.00, 3720.0), (2059.00, 3833.0), (2060.00, 3943.0), (2061.00, 4025.0)

UNITS: persons/year

NIM_Arrivals_M[F, A84] = GRAPH(TIME)

Points: (2011.00, 911.0), (2012.00, 924.0), (2013.00, 1050.0), (2014.00, 961.0), (2015.00, 970.0), (2016.00, 1007.0), (2017.00, 1084.0), (2018.00, 1144.0), (2019.00, 1185.0), (2020.00, 1225.0), (2021.00, 1272.0), (2022.00, 1328.0), (2023.00, 1383.0), (2024.00, 1463.0), (2025.00, 1542.0), (2026.00, 1627.0), (2027.00, 1759.0), (2028.00, 1860.0), (2029.00, 1937.0), (2030.00, 2008.0), (2031.00, 2077.0), (2032.00, 2091.0), (2033.00, 2137.0), (2034.00, 2188.0), (2035.00, 2244.0), (2036.00, 2301.0), (2037.00, 2362.0), (2038.00, 2423.0), (2039.00, 2486.0), (2040.00, 2546.0), (2041.00, 2614.0), (2042.00, 2671.0), (2043.00, 2720.0), (2044.00, 2750.0), (2045.00, 2760.0), (2046.00, 2748.0), (2047.00, 2737.0), (2048.00, 2739.0), (2049.00, 2773.0), (2050.00, 2827.0), (2051.00, 2928.0), (2052.00, 3016.0), (2053.00, 3072.0), (2054.00, 3093.0), (2055.00, 3093.0), (2056.00, 3055.0), (2057.00, 3025.0), (2058.00, 3024.0), (2059.00, 3048.0), (2060.00, 3097.0), (2061.00, 3178.0)

UNITS: persons/year

NIM_Arrivals_M[F, A89] = GRAPH(TIME)

Points: (2011.00, 623.0), (2012.00, 631.0), (2013.00, 718.0), (2014.00, 657.0), (2015.00, 663.0), (2016.00, 688.0), (2017.00, 741.0), (2018.00, 829.0), (2019.00, 833.0), (2020.00, 842.0), (2021.00, 861.0), (2022.00, 885.0), (2023.00, 914.0), (2024.00, 946.0), (2025.00, 985.0), (2026.00, 1024.0), (2027.00, 1072.0), (2028.00, 1120.0), (2029.00, 1188.0), (2030.00, 1257.0), (2031.00, 1326.0), (2032.00, 1443.0), (2033.00, 1527.0), (2034.00, 1588.0), (2035.00, 1648.0), (2036.00, 1696.0), (2037.00, 1713.0), (2038.00, 1755.0), (2039.00, 1800.0), (2040.00, 1847.0), (2041.00, 1900.0), (2042.00, 1951.0), (2043.00, 2002.0), (2044.00, 2057.0), (2045.00, 2109.0), (2046.00, 2168.0), (2047.00, 2218.0), (2048.00, 2257.0), (2049.00, 2285.0), (2050.00, 2289.0), (2051.00, 2279.0), (2052.00, 2274.0), (2053.00, 2279.0), (2054.00, 2316.0), (2055.00, 2366.0), (2056.00, 2458.0), (2057.00, 2534.0), (2058.00, 2578.0), (2059.00, 2594.0), (2060.00, 2589.0), (2061.00, 2559.0)

UNITS: persons/year

NIM_Arrivals_M[F, A94] = GRAPH(TIME)

Points: (2011.00, 263.0), (2012.00, 267.0), (2013.00, 304.0), (2014.00, 278.0), (2015.00, 280.0), (2016.00, 291.0), (2017.00, 314.0), (2018.00, 414.0), (2019.00, 421.0), (2020.00, 429.0), (2021.00, 435.0), (2022.00, 438.0), (2023.00, 441.0), (2024.00, 445.0), (2025.00, 451.0), (2026.00, 465.0), (2027.00, 478.0), (2028.00, 493.0), (2029.00, 514.0), (2030.00, 536.0), (2031.00, 557.0), (2032.00, 586.0), (2033.00, 613.0), (2034.00, 653.0), (2035.00, 696.0), (2036.00, 734.0), (2037.00, 802.0), (2038.00, 851.0), (2039.00, 880.0), (2040.00, 913.0), (2041.00, 945.0), (2042.00, 958.0), (2043.00, 984.0), (2044.00, 1012.0), (2045.00, 1041.0), (2046.00, 1072.0), (2047.00, 1106.0), (2048.00, 1138.0), (2049.00, 1171.0), (2050.00, 1204.0), (2051.00, 1242.0), (2052.00, 1273.0), (2053.00, 1296.0), (2054.00, 1314.0), (2055.00, 1320.0), (2056.00, 1316.0), (2057.00, 1318.0), (2058.00, 1327.0), (2059.00, 1354.0), (2060.00, 1389.0), (2061.00, 1446.0)

UNITS: persons/year

NIM_Arrivals_M[F, A99] = GRAPH(TIME)

Points: (2011.00, 69.0), (2012.00, 70.0), (2013.00, 79.0), (2014.00, 73.0), (2015.00, 73.0), (2016.00, 76.0), (2017.00, 82.0), (2018.00, 115.0), (2019.00, 122.0), (2020.00, 126.0), (2021.00, 131.0), (2022.00, 134.0), (2023.00, 137.0), (2024.00, 140.0), (2025.00, 144.0), (2026.00, 146.0), (2027.00, 146.0), (2028.00, 147.0), (2029.00, 149.0), (2030.00, 153.0), (2031.00, 157.0), (2032.00, 163.0), (2033.00, 170.0), (2034.00, 178.0), (2035.00, 187.0), (2036.00, 194.0), (2037.00, 206.0), (2038.00, 216.0), (2039.00, 233.0), (2040.00, 249.0), (2041.00, 266.0), (2042.00, 294.0), (2043.00, 311.0), (2044.00, 322.0), (2045.00, 335.0), (2046.00, 347.0), (2047.00, 354.0), (2048.00, 367.0), (2049.00, 381.0), (2050.00, 393.0), (2051.00, 407.0), (2052.00, 421.0), (2053.00, 437.0), (2054.00, 452.0), (2055.00, 466.0), (2056.00, 484.0), (2057.00, 499.0), (2058.00, 510.0), (2059.00, 519.0), (2060.00, 522.0), (2061.00, 525.0)

UNITS: persons/year

NIM_Arrivals_M[F, A104] = GRAPH(TIME)

Points: (2011.00, 9.0), (2012.00, 9.0), (2013.00, 11.0), (2014.00, 10.0), (2015.00, 10.0), (2016.00, 10.0), (2017.00, 11.0), (2018.00, 21.0), (2019.00, 22.0), (2020.00, 24.0), (2021.00, 27.0), (2022.00, 29.0), (2023.00, 31.0), (2024.00, 33.0), (2025.00, 34.0), (2026.00, 36.0), (2027.00, 37.0), (2028.00, 39.0), (2029.00, 40.0), (2030.00, 41.0), (2031.00, 42.0), (2032.00, 42.0), (2033.00, 43.0), (2034.00, 44.0), (2035.00, 45.0), (2036.00, 47.0), (2037.00, 49.0), (2038.00, 51.0), (2039.00, 53.0), (2040.00, 56.0), (2041.00, 59.0), (2042.00, 63.0), (2043.00, 67.0), (2044.00, 72.0), (2045.00, 77.0), (2046.00, 83.0), (2047.00, 91.0), (2048.00, 98.0), (2049.00, 103.0), (2050.00, 108.0), (2051.00, 114.0), (2052.00, 119.0), (2053.00, 124.0), (2054.00, 130.0), (2055.00, 136.0), (2056.00, 142.0), (2057.00, 148.0), (2058.00, 155.0), (2059.00, 162.0), (2060.00, 169.0), (2061.00, 176.0)

UNITS: persons/year

NIM_Departures_H[M, A4] = GRAPH(TIME)

Points: (2011.00, 14810), (2012.00, 13430), (2013.00, 14270), (2014.00, 13530), (2015.00, 14830), (2016.00, 16080), (2017.00, 16070), (2018.00, 15634), (2019.00, 15977), (2020.00, 16311), (2021.00, 16637), (2022.00, 16947), (2023.00, 17244), (2024.00, 17527), (2025.00, 17793), (2026.00, 18049), (2027.00, 18290), (2028.00, 18514), (2029.00, 18722), (2030.00, 18914), (2031.00, 19094), (2032.00, 19272), (2033.00, 19452), (2034.00, 19641), (2035.00, 19839), (2036.00, 20048), (2037.00, 20270), (2038.00, 20504), (2039.00, 20750), (2040.00, 21013), (2041.00, 21284), (2042.00, 21567), (2043.00, 21862), (2044.00, 22163), (2045.00, 22469), (2046.00, 22781), (2047.00, 23094), (2048.00, 23408), (2049.00, 23722), (2050.00, 24032), (2051.00, 24338), (2052.00, 24639), (2053.00, 24934), (2054.00, 25220), (2055.00, 25498), (2056.00, 25769), (2057.00, 26030), (2058.00, 26284), (2059.00, 26529), (2060.00, 26768), (2061.00, 27000)

UNITS: persons/year

NIM_Departures_H[M, A9] = GRAPH(TIME)

Points: (2011.00, 10830.0), (2012.00, 9840.0), (2013.00, 10930.0), (2014.00, 10360.0), (2015.00, 11460.0), (2016.00, 12550.0), (2017.00, 12610.0), (2018.00, 11770.0), (2019.00, 12035.0), (2020.00, 12311.0), (2021.00, 12643.0), (2022.00, 12924.0), (2023.00, 13200.0), (2024.00, 13474.0), (2025.00, 13740.0), (2026.00, 13999.0), (2027.00, 14245.0), (2028.00, 14480.0), (2029.00, 14703.0), (2030.00, 14915.0), (2031.00, 15118.0), (2032.00, 15309.0), (2033.00, 15484.0), (2034.00, 15646.0), (2035.00, 15800.0), (2036.00, 15944.0), (2037.00, 16088.0), (2038.00, 16234.0), (2039.00, 16387.0), (2040.00, 16546.0), (2041.00, 16716.0), (2042.00, 16895.0), (2043.00, 17085.0), (2044.00, 17285.0), (2045.00, 17496.0), (2046.00, 17715.0), (2047.00, 17943.0), (2048.00, 18180.0), (2049.00, 18421.0), (2050.00, 18668.0), (2051.00, 18917.0), (2052.00, 19168.0), (2053.00, 19421.0), (2054.00, 19669.0), (2055.00, 19917.0), (2056.00, 20162.0), (2057.00, 20402.0), (2058.00, 20636.0), (2059.00, 20865.0), (2060.00, 21086.0), (2061.00, 21302.0)

UNITS: persons/year

NIM_Departures_H[M, A14] = GRAPH(TIME)

Points: (2011.00, 8420.0), (2012.00, 7360.0), (2013.00, 8220.0), (2014.00, 7640.0), (2015.00, 8350.0), (2016.00, 9020.0), (2017.00, 9010.0), (2018.00, 8387.0), (2019.00, 8590.0), (2020.00, 8781.0), (2021.00, 8898.0), (2022.00, 9083.0), (2023.00, 9276.0), (2024.00, 9474.0), (2025.00, 9681.0), (2026.00, 9931.0), (2027.00, 10141.0), (2028.00, 10347.0), (2029.00, 10553.0), (2030.00, 10752.0), (2031.00, 10945.0), (2032.00, 11129.0), (2033.00, 11306.0), (2034.00, 11472.0), (2035.00, 11630.0), (2036.00, 11784.0), (2037.00, 11926.0), (2038.00, 12057.0), (2039.00, 12178.0), (2040.00, 12292.0), (2041.00, 12400.0), (2042.00, 12507.0), (2043.00, 12616.0), (2044.00, 12729.0), (2045.00, 12849.0), (2046.00, 12976.0), (2047.00, 13108.0), (2048.00, 13251.0), (2049.00, 13401.0), (2050.00, 13558.0), (2051.00, 13723.0), (2052.00, 13893.0), (2053.00, 14069.0), (2054.00, 14250.0), (2055.00, 14434.0), (2056.00, 14621.0), (2057.00, 14808.0), (2058.00, 14997.0), (2059.00, 15183.0), (2060.00, 15370.0), (2061.00, 15552.0)

UNITS: persons/year

NIM_Departures_H[M, A19] = GRAPH(TIME)

Points: (2011.00, 11990.0), (2012.00, 11040.0), (2013.00, 11660.0), (2014.00, 10790.0), (2015.00, 10870.0), (2016.00, 11300.0), (2017.00, 11040.0), (2018.00, 11389.0), (2019.00, 11493.0), (2020.00, 11607.0), (2021.00, 11754.0), (2022.00, 11994.0), (2023.00, 12279.0), (2024.00, 12593.0), (2025.00, 12869.0), (2026.00, 13037.0), (2027.00, 13281.0), (2028.00, 13513.0), (2029.00, 13721.0), (2030.00, 13967.0), (2031.00, 14344.0), (2032.00, 14621.0), (2033.00, 14893.0), (2034.00, 15165.0), (2035.00, 15433.0), (2036.00, 15693.0), (2037.00, 15946.0), (2038.00, 16184.0), (2039.00, 16412.0), (2040.00, 16627.0), (2041.00, 16833.0), (2042.00, 17027.0), (2043.00, 17207.0), (2044.00, 17378.0), (2045.00, 17535.0), (2046.00, 17678.0), (2047.00, 17815.0), (2048.00, 17955.0), (2049.00, 18098.0), (2050.00, 18248.0), (2051.00, 18408.0), (2052.00, 18576.0), (2053.00, 18754.0), (2054.00, 18945.0), (2055.00, 19142.0), (2056.00, 19351.0), (2057.00, 19571.0), (2058.00, 19799.0), (2059.00, 20033.0), (2060.00, 20273.0), (2061.00, 20517.0)

UNITS: persons/year

NIM_Departures_H[M, A24] = GRAPH(TIME)

Points: (2011.00, 23570.0), (2012.00, 23740.0), (2013.00, 24100.0), (2014.00, 24480.0), (2015.00, 21950.0), (2016.00, 22400.0), (2017.00, 22440.0), (2018.00, 25445.0), (2019.00, 25604.0), (2020.00, 25662.0), (2021.00, 25819.0), (2022.00, 25935.0), (2023.00, 26097.0), (2024.00, 26304.0), (2025.00, 26597.0), (2026.00, 27006.0), (2027.00, 27535.0), (2028.00, 28130.0), (2029.00, 28712.0), (2030.00, 29248.0), (2031.00, 29591.0), (2032.00, 30078.0), (2033.00, 30557.0), (2034.00, 31069.0), (2035.00, 31613.0), (2036.00, 32319.0), (2037.00, 32887.0), (2038.00, 33444.0), (2039.00, 33998.0), (2040.00, 34543.0), (2041.00, 35069.0), (2042.00, 35572.0), (2043.00, 36051.0), (2044.00, 36508.0), (2045.00, 36940.0), (2046.00, 37352.0), (2047.00, 37741.0), (2048.00, 38101.0), (2049.00, 38437.0), (2050.00, 38749.0), (2051.00, 39038.0), (2052.00, 39319.0), (2053.00, 39607.0), (2054.00, 39908.0), (2055.00, 40221.0), (2056.00, 40552.0), (2057.00, 40903.0), (2058.00, 41276.0), (2059.00, 41671.0), (2060.00, 42087.0), (2061.00, 42522.0)

UNITS: persons/year

NIM_Departures_H[M, A29] = GRAPH(TIME)

Points: (2011.00, 27820.0), (2012.00, 28020.0), (2013.00, 28720.0), (2014.00, 28290.0), (2015.00, 27110.0), (2016.00, 27720.0), (2017.00, 27340.0), (2018.00, 29706.0), (2019.00, 29968.0), (2020.00, 30209.0), (2021.00, 30451.0), (2022.00, 30736.0), (2023.00, 30962.0), (2024.00, 31140.0), (2025.00, 31244.0), (2026.00, 31437.0), (2027.00, 31570.0), (2028.00, 31753.0), (2029.00, 32017.0), (2030.00, 32397.0), (2031.00, 32930.0), (2032.00, 33531.0), (2033.00, 34188.0), (2034.00, 34819.0), (2035.00, 35405.0), (2036.00, 35769.0), (2037.00, 36338.0), (2038.00, 36917.0), (2039.00, 37524.0), (2040.00, 38158.0), (2041.00, 38921.0), (2042.00, 39565.0), (2043.00, 40196.0), (2044.00, 40825.0), (2045.00, 41436.0), (2046.00, 42029.0), (2047.00, 42595.0), (2048.00, 43133.0), (2049.00, 43647.0), (2050.00, 44135.0), (2051.00, 44601.0), (2052.00, 45036.0), (2053.00, 45440.0), (2054.00, 45815.0), (2055.00, 46165.0), (2056.00, 46497.0), (2057.00, 46822.0), (2058.00, 47157.0), (2059.00, 47503.0), (2060.00, 47870.0), (2061.00, 48254.0)

UNITS: persons/year

NIM_Departures_H[M, A34] = GRAPH(TIME)

Points: (2011.00, 19850.0), (2012.00, 20200.0), (2013.00, 21510.0), (2014.00, 21440.0), (2015.00, 22020.0), (2016.00, 23380.0), (2017.00, 23290.0), (2018.00, 24211.0), (2019.00, 24629.0), (2020.00, 25080.0), (2021.00, 25402.0), (2022.00, 25658.0), (2023.00, 25909.0), (2024.00, 26126.0), (2025.00, 26324.0), (2026.00, 26524.0), (2027.00, 26750.0), (2028.00, 26928.0), (2029.00, 27074.0), (2030.00, 27163.0), (2031.00, 27316.0), (2032.00, 27423.0), (2033.00, 27570.0), (2034.00, 27787.0), (2035.00, 28096.0), (2036.00, 28528.0), (2037.00, 29012.0), (2038.00, 29535.0), (2039.00, 30036.0), (2040.00, 30502.0), (2041.00, 30794.0), (2042.00, 31247.0), (2043.00, 31717.0), (2044.00, 32204.0), (2045.00, 32713.0), (2046.00, 33321.0), (2047.00, 33836.0), (2048.00, 34340.0), (2049.00, 34842.0), (2050.00, 35332.0), (2051.00, 35806.0), (2052.00, 36258.0), (2053.00, 36691.0), (2054.00, 37100.0), (2055.00, 37490.0), (2056.00, 37862.0), (2057.00, 38211.0), (2058.00, 38535.0), (2059.00, 38835.0), (2060.00, 39113.0), (2061.00, 39379.0)

UNITS: persons/year

NIM_Departures_H[M, A39] = GRAPH(TIME)

Points: (2011.00, 15620.0), (2012.00, 14640.0), (2013.00, 15300.0), (2014.00, 14700.0), (2015.00, 15530.0), (2016.00, 16600.0), (2017.00, 17130.0), (2018.00, 17532.0), (2019.00, 18164.0), (2020.00, 18769.0), (2021.00, 19252.0), (2022.00, 19632.0), (2023.00, 19923.0), (2024.00, 20248.0), (2025.00, 20603.0), (2026.00, 20845.0), (2027.00, 21037.0), (2028.00, 21231.0), (2029.00, 21403.0), (2030.00, 21559.0), (2031.00, 21713.0), (2032.00, 21887.0), (2033.00, 22022.0), (2034.00, 22136.0), (2035.00, 22207.0), (2036.00, 22326.0), (2037.00, 22409.0), (2038.00, 22523.0), (2039.00, 22693.0), (2040.00, 22936.0), (2041.00, 23273.0), (2042.00, 23645.0), (2043.00, 24047.0), (2044.00, 24431.0), (2045.00, 24792.0), (2046.00, 25013.0), (2047.00, 25367.0), (2048.00, 25728.0), (2049.00, 26108.0), (2050.00, 26498.0), (2051.00, 26965.0), (2052.00, 27363.0), (2053.00, 27752.0), (2054.00, 28138.0), (2055.00, 28516.0), (2056.00, 28881.0), (2057.00, 29230.0), (2058.00, 29562.0), (2059.00, 29878.0), (2060.00, 30178.0), (2061.00, 30466.0)

UNITS: persons/year

NIM_Departures_H[M, A44] = GRAPH(TIME)

Points: (2011.00, 12210.0), (2012.00, 11620.0), (2013.00, 12500.0), (2014.00, 11990.0), (2015.00, 12090.0), (2016.00, 12590.0), (2017.00, 12530.0), (2018.00, 11666.0), (2019.00, 11814.0), (2020.00, 12059.0), (2021.00, 12451.0), (2022.00, 12902.0), (2023.00, 13423.0), (2024.00, 13884.0), (2025.00, 14324.0), (2026.00, 14673.0), (2027.00, 14948.0), (2028.00, 15161.0), (2029.00, 15396.0), (2030.00, 15652.0), (2031.00, 15831.0), (2032.00, 15976.0), (2033.00, 16113.0), (2034.00, 16239.0), (2035.00, 16357.0), (2036.00, 16472.0), (2037.00, 16601.0), (2038.00, 16701.0), (2039.00, 16784.0), (2040.00, 16838.0), (2041.00, 16926.0), (2042.00, 16988.0), (2043.00, 17072.0), (2044.00, 17197.0), (2045.00, 17372.0), (2046.00, 17618.0), (2047.00, 17893.0), (2048.00, 18188.0), (2049.00, 18466.0), (2050.00, 18727.0), (2051.00, 18891.0), (2052.00, 19149.0), (2053.00, 19413.0), (2054.00, 19690.0), (2055.00, 19979.0), (2056.00, 20317.0), (2057.00, 20607.0), (2058.00, 20892.0), (2059.00, 21174.0), (2060.00, 21450.0), (2061.00, 21717.0)

UNITS: persons/year

NIM_Departures_H[M, A49] = GRAPH(TIME)

Points: (2011.00, 9080.0), (2012.00, 8390.0), (2013.00, 9000.0), (2014.00, 8670.0), (2015.00, 8840.0), (2016.00, 9870.0), (2017.00, 10210.0), (2018.00, 9549.0), (2019.00, 9614.0), (2020.00, 9622.0), (2021.00, 9525.0), (2022.00, 9462.0), (2023.00, 9483.0), (2024.00, 9596.0), (2025.00, 9782.0), (2026.00, 10083.0), (2027.00, 10431.0), (2028.00, 10841.0), (2029.00, 11206.0), (2030.00, 11558.0), (2031.00, 11838.0), (2032.00, 12062.0), (2033.00, 12234.0), (2034.00, 12424.0), (2035.00, 12624.0), (2036.00, 12768.0), (2037.00, 12887.0), (2038.00, 13005.0), (2039.00, 13107.0), (2040.00, 13197.0), (2041.00, 13289.0), (2042.00, 13395.0), (2043.00, 13479.0), (2044.00, 13549.0), (2045.00, 13592.0), (2046.00, 13661.0), (2047.00, 13714.0), (2048.00, 13781.0), (2049.00, 13882.0), (2050.00, 14018.0), (2051.00, 14209.0), (2052.00, 14424.0), (2053.00, 14659.0), (2054.00, 14882.0), (2055.00, 15093.0), (2056.00, 15226.0), (2057.00, 15430.0), (2058.00, 15637.0), (2059.00, 15856.0), (2060.00, 16081.0), (2061.00, 16354.0)

UNITS: persons/year

NIM_Departures_H[M, A54] = GRAPH(TIME)

Points: (2011.00, 7390.0), (2012.00, 7260.0), (2013.00, 7660.0), (2014.00, 7140.0), (2015.00, 7250.0), (2016.00, 7820.0), (2017.00, 8150.0), (2018.00, 7665.0), (2019.00, 7754.0), (2020.00, 7920.0), (2021.00, 8192.0), (2022.00, 8432.0), (2023.00, 8577.0), (2024.00, 8639.0), (2025.00, 8654.0), (2026.00, 8569.0), (2027.00, 8514.0), (2028.00, 8528.0), (2029.00, 8627.0), (2030.00, 8790.0), (2031.00, 9055.0), (2032.00, 9361.0), (2033.00, 9722.0), (2034.00, 10046.0), (2035.00, 10361.0), (2036.00, 10614.0), (2037.00, 10817.0), (2038.00, 10971.0), (2039.00, 11143.0), (2040.00, 11322.0), (2041.00, 11451.0), (2042.00, 11561.0), (2043.00, 11669.0), (2044.00, 11763.0), (2045.00, 11843.0), (2046.00, 11926.0), (2047.00, 12024.0), (2048.00, 12102.0), (2049.00, 12167.0), (2050.00, 12205.0), (2051.00, 12270.0), (2052.00, 12317.0), (2053.00, 12381.0), (2054.00, 12470.0), (2055.00, 12592.0), (2056.00, 12761.0), (2057.00, 12951.0), (2058.00, 13162.0), (2059.00, 13364.0), (2060.00, 13555.0), (2061.00, 13675.0)

UNITS: persons/year

NIM_Departures_H[M, A59] = GRAPH(TIME)

Points: (2011.00, 6140.0), (2012.00, 6060.0), (2013.00, 6410.0), (2014.00, 6010.0), (2015.00, 6210.0), (2016.00, 6950.0), (2017.00, 7570.0), (2018.00, 7333.0), (2019.00, 7402.0), (2020.00, 7418.0), (2021.00, 7374.0), (2022.00, 7340.0), (2023.00, 7352.0), (2024.00, 7440.0), (2025.00, 7603.0), (2026.00, 7864.0), (2027.00, 8098.0), (2028.00, 8244.0), (2029.00, 8306.0), (2030.00, 8320.0), (2031.00, 8242.0), (2032.00, 8195.0), (2033.00, 8214.0), (2034.00, 8310.0), (2035.00, 8470.0), (2036.00, 8724.0), (2037.00, 9023.0), (2038.00, 9370.0), (2039.00, 9685.0), (2040.00, 9989.0), (2041.00, 10232.0), (2042.00, 10432.0), (2043.00, 10583.0), (2044.00, 10751.0), (2045.00, 10926.0), (2046.00, 11057.0), (2047.00, 11166.0), (2048.00, 11272.0), (2049.00, 11367.0), (2050.00, 11447.0), (2051.00, 11531.0), (2052.00, 11629.0), (2053.00, 11708.0), (2054.00, 11771.0), (2055.00, 11813.0), (2056.00, 11879.0), (2057.00, 11929.0), (2058.00, 11994.0), (2059.00, 12083.0), (2060.00, 12203.0), (2061.00, 12368.0)

UNITS: persons/year

NIM_Departures_H[M, A64] = GRAPH(TIME)

Points: (2011.00, 5140.0), (2012.00, 5040.0), (2013.00, 5480.0), (2014.00, 5080.0), (2015.00, 5400.0), (2016.00, 6100.0), (2017.00, 6370.0), (2018.00, 5865.0), (2019.00, 6004.0), (2020.00, 6128.0), (2021.00, 6269.0), (2022.00, 6406.0), (2023.00, 6512.0), (2024.00, 6578.0), (2025.00, 6594.0), (2026.00, 6560.0), (2027.00, 6537.0), (2028.00, 6559.0), (2029.00, 6648.0), (2030.00, 6800.0), (2031.00, 7040.0), (2032.00, 7248.0), (2033.00, 7376.0), (2034.00, 7429.0), (2035.00, 7446.0), (2036.00, 7385.0), (2037.00, 7348.0), (2038.00, 7374.0), (2039.00, 7469.0), (2040.00, 7620.0), (2041.00, 7856.0), (2042.00, 8129.0), (2043.00, 8445.0), (2044.00, 8726.0), (2045.00, 9001.0), (2046.00, 9223.0), (2047.00, 9401.0), (2048.00, 9542.0), (2049.00, 9697.0), (2050.00, 9862.0), (2051.00, 9980.0), (2052.00, 10078.0), (2053.00, 10177.0), (2054.00, 10265.0), (2055.00, 10344.0), (2056.00, 10422.0), (2057.00, 10512.0), (2058.00, 10586.0), (2059.00, 10647.0), (2060.00, 10690.0), (2061.00, 10752.0)

UNITS: persons/year

NIM_Departures_H[M, A69] = GRAPH(TIME)

Points: (2011.00, 3110.0), (2012.00, 3470.0), (2013.00, 4010.0), (2014.00, 3820.0), (2015.00, 4290.0), (2016.00, 4930.0), (2017.00, 4950.0), (2018.00, 4319.0), (2019.00, 4390.0), (2020.00, 4471.0), (2021.00, 4576.0), (2022.00, 4678.0), (2023.00, 4778.0), (2024.00, 4895.0), (2025.00, 5004.0), (2026.00, 5128.0), (2027.00, 5247.0), (2028.00, 5338.0), (2029.00, 5397.0), (2030.00, 5410.0), (2031.00, 5388.0), (2032.00, 5373.0), (2033.00, 5398.0), (2034.00, 5481.0), (2035.00, 5614.0), (2036.00, 5819.0), (2037.00, 5995.0), (2038.00, 6100.0), (2039.00, 6143.0), (2040.00, 6151.0), (2041.00, 6106.0), (2042.00, 6082.0), (2043.00, 6110.0), (2044.00, 6193.0), (2045.00, 6325.0), (2046.00, 6528.0), (2047.00, 6759.0), (2048.00, 7024.0), (2049.00, 7262.0), (2050.00, 7489.0), (2051.00, 7672.0), (2052.00, 7822.0), (2053.00, 7941.0), (2054.00, 8072.0), (2055.00, 8212.0), (2056.00, 8316.0), (2057.00, 8399.0), (2058.00, 8482.0), (2059.00, 8557.0), (2060.00, 8630.0), (2061.00, 8702.0)

UNITS: persons/year

NIM_Departures_H[M, A74] = GRAPH(TIME)

Points: (2011.00, 1710.0), (2012.00, 1850.0), (2013.00, 2160.0), (2014.00, 2030.0), (2015.00, 2400.0), (2016.00, 2800.0), (2017.00, 3210.0), (2018.00, 2859.0), (2019.00, 2957.0), (2020.00, 3054.0), (2021.00, 3128.0), (2022.00, 3136.0), (2023.00, 3185.0), (2024.00, 3245.0), (2025.00, 3313.0), (2026.00, 3398.0), (2027.00, 3478.0), (2028.00, 3561.0), (2029.00, 3659.0), (2030.00, 3746.0), (2031.00, 3846.0), (2032.00, 3943.0), (2033.00, 4017.0), (2034.00, 4068.0), (2035.00, 4085.0), (2036.00, 4073.0), (2037.00, 4067.0), (2038.00, 4088.0), (2039.00, 4155.0), (2040.00, 4257.0), (2041.00, 4416.0), (2042.00, 4554.0), (2043.00, 4637.0), (2044.00, 4677.0), (2045.00, 4689.0), (2046.00, 4657.0), (2047.00, 4643.0), (2048.00, 4665.0), (2049.00, 4730.0), (2050.00, 4831.0), (2051.00, 4986.0), (2052.00, 5166.0), (2053.00, 5370.0), (2054.00, 5556.0), (2055.00, 5734.0), (2056.00, 5876.0), (2057.00, 5993.0), (2058.00, 6089.0), (2059.00, 6192.0), (2060.00, 6301.0), (2061.00, 6382.0)

UNITS: persons/year

NIM_Departures_H[M, A79] = GRAPH(TIME)

Points: (2011.00, 902.0), (2012.00, 941.0), (2013.00, 1089.0), (2014.00, 1032.0), (2015.00, 1107.0), (2016.00, 1207.0), (2017.00, 1268.0), (2018.00, 1526.0), (2019.00, 1605.0), (2020.00, 1691.0), (2021.00, 1779.0), (2022.00, 1924.0), (2023.00, 2044.0), (2024.00, 2124.0), (2025.00, 2199.0), (2026.00, 2265.0), (2027.00, 2275.0), (2028.00, 2318.0), (2029.00, 2368.0), (2030.00, 2426.0), (2031.00, 2494.0), (2032.00, 2561.0), (2033.00, 2627.0), (2034.00, 2706.0), (2035.00, 2779.0), (2036.00, 2857.0), (2037.00, 2934.0), (2038.00, 2997.0), (2039.00, 3042.0), (2040.00, 3062.0), (2041.00, 3057.0), (2042.00, 3056.0), (2043.00, 3072.0), (2044.00, 3123.0), (2045.00, 3203.0), (2046.00, 3324.0), (2047.00, 3435.0), (2048.00, 3506.0), (2049.00, 3545.0), (2050.00, 3563.0), (2051.00, 3540.0), (2052.00, 3530.0), (2053.00, 3548.0), (2054.00, 3597.0), (2055.00, 3676.0), (2056.00, 3796.0), (2057.00, 3934.0), (2058.00, 4092.0), (2059.00, 4238.0), (2060.00, 4377.0), (2061.00, 4492.0)

UNITS: persons/year

NIM_Departures_H[M, A84] = GRAPH(TIME)

Points: (2011.00, 671.0), (2012.00, 700.0), (2013.00, 810.0), (2014.00, 768.0), (2015.00, 823.0), (2016.00, 898.0), (2017.00, 943.0), (2018.00, 935.0), (2019.00, 983.0), (2020.00, 1035.0), (2021.00, 1085.0), (2022.00, 1141.0), (2023.00, 1188.0), (2024.00, 1259.0), (2025.00, 1329.0), (2026.00, 1409.0), (2027.00, 1533.0), (2028.00, 1636.0), (2029.00, 1703.0), (2030.00, 1768.0), (2031.00, 1829.0), (2032.00, 1846.0), (2033.00, 1889.0), (2034.00, 1938.0), (2035.00, 1993.0), (2036.00, 2058.0), (2037.00, 2121.0), (2038.00, 2184.0), (2039.00, 2255.0), (2040.00, 2323.0), (2041.00, 2398.0), (2042.00, 2469.0), (2043.00, 2528.0), (2044.00, 2571.0), (2045.00, 2592.0), (2046.00, 2595.0), (2047.00, 2601.0), (2048.00, 2625.0), (2049.00, 2676.0), (2050.00, 2752.0), (2051.00, 2863.0), (2052.00, 2962.0), (2053.00, 3029.0), (2054.00, 3064.0), (2055.00, 3085.0), (2056.00, 3073.0), (2057.00, 3071.0), (2058.00, 3092.0), (2059.00, 3143.0), (2060.00, 3217.0), (2061.00, 3328.0)

UNITS: persons/year

NIM_Departures_H[M, A89] = GRAPH(TIME)

Points: (2011.00, 359.0), (2012.00, 375.0), (2013.00, 434.0), (2014.00, 411.0), (2015.00, 441.0), (2016.00, 481.0), (2017.00, 505.0), (2018.00, 519.0), (2019.00, 528.0), (2020.00, 537.0), (2021.00, 556.0), (2022.00, 584.0), (2023.00, 615.0), (2024.00, 653.0), (2025.00, 690.0), (2026.00, 728.0), (2027.00, 771.0), (2028.00, 810.0), (2029.00, 863.0), (2030.00, 917.0), (2031.00, 980.0), (2032.00, 1072.0), (2033.00, 1156.0), (2034.00, 1219.0), (2035.00, 1280.0), (2036.00, 1332.0), (2037.00, 1356.0), (2038.00, 1398.0), (2039.00, 1446.0), (2040.00, 1498.0), (2041.00, 1557.0), (2042.00, 1617.0), (2043.00, 1678.0), (2044.00, 1745.0), (2045.00, 1809.0), (2046.00, 1879.0), (2047.00, 1947.0), (2048.00, 2009.0), (2049.00, 2060.0), (2050.00, 2091.0), (2051.00, 2108.0), (2052.00, 2122.0), (2053.00, 2148.0), (2054.00, 2197.0), (2055.00, 2268.0), (2056.00, 2369.0), (2057.00, 2467.0), (2058.00, 2543.0), (2059.00, 2592.0), (2060.00, 2624.0), (2061.00, 2624.0)

UNITS: persons/year

NIM_Departures_H[M, A94] = GRAPH(TIME)

Points: (2011.00, 113.0), (2012.00, 118.0), (2013.00, 136.0), (2014.00, 129.0), (2015.00, 139.0), (2016.00, 151.0), (2017.00, 159.0), (2018.00, 224.0), (2019.00, 234.0), (2020.00, 246.0), (2021.00, 255.0), (2022.00, 261.0), (2023.00, 266.0), (2024.00, 273.0), (2025.00, 281.0), (2026.00, 295.0), (2027.00, 314.0), (2028.00, 333.0), (2029.00, 355.0), (2030.00, 380.0), (2031.00, 403.0), (2032.00, 432.0), (2033.00, 458.0), (2034.00, 497.0), (2035.00, 539.0), (2036.00, 582.0), (2037.00, 654.0), (2038.00, 708.0), (2039.00, 748.0), (2040.00, 794.0), (2041.00, 832.0), (2042.00, 858.0), (2043.00, 901.0), (2044.00, 940.0), (2045.00, 987.0), (2046.00, 1040.0), (2047.00, 1090.0), (2048.00, 1142.0), (2049.00, 1200.0), (2050.00, 1255.0), (2051.00, 1315.0), (2052.00, 1378.0), (2053.00, 1429.0), (2054.00, 1474.0), (2055.00, 1504.0), (2056.00, 1526.0), (2057.00, 1551.0), (2058.00, 1586.0), (2059.00, 1640.0), (2060.00, 1710.0), (2061.00, 1805.0)

UNITS: persons/year

NIM_Departures_H[M, A99] = GRAPH(TIME)

Points: (2011.00, 21.0), (2012.00, 21.0), (2013.00, 25.0), (2014.00, 24.0), (2015.00, 25.0), (2016.00, 28.0), (2017.00, 29.0), (2018.00, 51.0), (2019.00, 56.0), (2020.00, 60.0), (2021.00, 64.0), (2022.00, 67.0), (2023.00, 71.0), (2024.00, 74.0), (2025.00, 78.0), (2026.00, 81.0), (2027.00, 84.0), (2028.00, 85.0), (2029.00, 88.0), (2030.00, 92.0), (2031.00, 97.0), (2032.00, 105.0), (2033.00, 112.0), (2034.00, 122.0), (2035.00, 134.0), (2036.00, 147.0), (2037.00, 162.0), (2038.00, 177.0), (2039.00, 197.0), (2040.00, 219.0), (2041.00, 243.0), (2042.00, 280.0), (2043.00, 310.0), (2044.00, 335.0), (2045.00, 362.0), (2046.00, 387.0), (2047.00, 410.0), (2048.00, 439.0), (2049.00, 467.0), (2050.00, 499.0), (2051.00, 538.0), (2052.00, 574.0), (2053.00, 612.0), (2054.00, 654.0), (2055.00, 698.0), (2056.00, 744.0), (2057.00, 792.0), (2058.00, 834.0), (2059.00, 873.0), (2060.00, 904.0), (2061.00, 931.0)

UNITS: persons/year

NIM_Departures_H[M, A104] = GRAPH(TIME)

Points: (2011.00, 2.0), (2012.00, 2.0), (2013.00, 2.0), (2014.00, 2.0), (2015.00, 2.0), (2016.00, 3.0), (2017.00, 3.0), (2018.00, 7.0), (2019.00, 7.0), (2020.00, 9.0), (2021.00, 10.0), (2022.00, 11.0), (2023.00, 12.0), (2024.00, 13.0), (2025.00, 14.0), (2026.00, 15.0), (2027.00, 16.0), (2028.00, 18.0), (2029.00, 19.0), (2030.00, 20.0), (2031.00, 20.0), (2032.00, 21.0), (2033.00, 22.0), (2034.00, 24.0), (2035.00, 25.0), (2036.00, 27.0), (2037.00, 30.0), (2038.00, 34.0), (2039.00, 38.0), (2040.00, 42.0), (2041.00, 48.0), (2042.00, 54.0), (2043.00, 61.0), (2044.00, 69.0), (2045.00, 79.0), (2046.00, 90.0), (2047.00, 107.0), (2048.00, 121.0), (2049.00, 134.0), (2050.00, 149.0), (2051.00, 164.0), (2052.00, 180.0), (2053.00, 198.0), (2054.00, 217.0), (2055.00, 238.0), (2056.00, 261.0), (2057.00, 285.0), (2058.00, 311.0), (2059.00, 339.0), (2060.00, 368.0), (2061.00, 401.0)

UNITS: persons/year

NIM_Departures_H[F, A4] = GRAPH(TIME)

Points: (2011.00, 13960.0), (2012.00, 12760.0), (2013.00, 13070.0), (2014.00, 12660.0), (2015.00, 14180.0), (2016.00, 14990.0), (2017.00, 14890.0), (2018.00, 14843.0), (2019.00, 15166.0), (2020.00, 15483.0), (2021.00, 15791.0), (2022.00, 16084.0), (2023.00, 16364.0), (2024.00, 16630.0), (2025.00, 16885.0), (2026.00, 17128.0), (2027.00, 17357.0), (2028.00, 17569.0), (2029.00, 17762.0), (2030.00, 17946.0), (2031.00, 18116.0), (2032.00, 18284.0), (2033.00, 18456.0), (2034.00, 18634.0), (2035.00, 18821.0), (2036.00, 19018.0), (2037.00, 19228.0), (2038.00, 19449.0), (2039.00, 19683.0), (2040.00, 19929.0), (2041.00, 20187.0), (2042.00, 20455.0), (2043.00, 20733.0), (2044.00, 21017.0), (2045.00, 21308.0), (2046.00, 21602.0), (2047.00, 21900.0), (2048.00, 22197.0), (2049.00, 22493.0), (2050.00, 22787.0), (2051.00, 23076.0), (2052.00, 23361.0), (2053.00, 23639.0), (2054.00, 23911.0), (2055.00, 24174.0), (2056.00, 24430.0), (2057.00, 24677.0), (2058.00, 24916.0), (2059.00, 25149.0), (2060.00, 25376.0), (2061.00, 25596.0)

UNITS: persons/year

NIM_Departures_H[F, A9] = GRAPH(TIME)

Points: (2011.00, 10430.0), (2012.00, 9540.0), (2013.00, 10480.0), (2014.00, 10080.0), (2015.00, 11030.0), (2016.00, 11750.0), (2017.00, 11820.0), (2018.00, 11304.0), (2019.00, 11563.0), (2020.00, 11835.0), (2021.00, 12155.0), (2022.00, 12431.0), (2023.00, 12695.0), (2024.00, 12956.0), (2025.00, 13211.0), (2026.00, 13456.0), (2027.00, 13692.0), (2028.00, 13916.0), (2029.00, 14128.0), (2030.00, 14330.0), (2031.00, 14524.0), (2032.00, 14705.0), (2033.00, 14873.0), (2034.00, 15027.0), (2035.00, 15173.0), (2036.00, 15311.0), (2037.00, 15446.0), (2038.00, 15586.0), (2039.00, 15731.0), (2040.00, 15883.0), (2041.00, 16044.0), (2042.00, 16215.0), (2043.00, 16395.0), (2044.00, 16586.0), (2045.00, 16786.0), (2046.00, 16995.0), (2047.00, 17213.0), (2048.00, 17438.0), (2049.00, 17668.0), (2050.00, 17902.0), (2051.00, 18140.0), (2052.00, 18380.0), (2053.00, 18618.0), (2054.00, 18858.0), (2055.00, 19094.0), (2056.00, 19326.0), (2057.00, 19554.0), (2058.00, 19777.0), (2059.00, 19996.0), (2060.00, 20205.0), (2061.00, 20412.0)

UNITS: persons/year

NIM_Departures_H[F, A14] = GRAPH(TIME)

Points: (2011.00, 8260.0), (2012.00, 7330.0), (2013.00, 8100.0), (2014.00, 7400.0), (2015.00, 7910.0), (2016.00, 8760.0), (2017.00, 8770.0), (2018.00, 8314.0), (2019.00, 8524.0), (2020.00, 8721.0), (2021.00, 8840.0), (2022.00, 9033.0), (2023.00, 9224.0), (2024.00, 9424.0), (2025.00, 9635.0), (2026.00, 9882.0), (2027.00, 10094.0), (2028.00, 10297.0), (2029.00, 10498.0), (2030.00, 10694.0), (2031.00, 10883.0), (2032.00, 11064.0), (2033.00, 11237.0), (2034.00, 11400.0), (2035.00, 11557.0), (2036.00, 11705.0), (2037.00, 11844.0), (2038.00, 11974.0), (2039.00, 12093.0), (2040.00, 12203.0), (2041.00, 12311.0), (2042.00, 12414.0), (2043.00, 12521.0), (2044.00, 12634.0), (2045.00, 12752.0), (2046.00, 12875.0), (2047.00, 13005.0), (2048.00, 13144.0), (2049.00, 13291.0), (2050.00, 13445.0), (2051.00, 13607.0), (2052.00, 13775.0), (2053.00, 13949.0), (2054.00, 14126.0), (2055.00, 14308.0), (2056.00, 14491.0), (2057.00, 14677.0), (2058.00, 14862.0), (2059.00, 15046.0), (2060.00, 15229.0), (2061.00, 15408.0)

UNITS: persons/year

NIM_Departures_H[F, A19] = GRAPH(TIME)

Points: (2011.00, 12730.0), (2012.00, 11850.0), (2013.00, 12750.0), (2014.00, 11510.0), (2015.00, 11560.0), (2016.00, 11910.0), (2017.00, 11990.0), (2018.00, 12291.0), (2019.00, 12382.0), (2020.00, 12483.0), (2021.00, 12646.0), (2022.00, 12868.0), (2023.00, 13150.0), (2024.00, 13495.0), (2025.00, 13815.0), (2026.00, 14010.0), (2027.00, 14287.0), (2028.00, 14537.0), (2029.00, 14754.0), (2030.00, 15018.0), (2031.00, 15425.0), (2032.00, 15742.0), (2033.00, 16031.0), (2034.00, 16322.0), (2035.00, 16606.0), (2036.00, 16884.0), (2037.00, 17152.0), (2038.00, 17408.0), (2039.00, 17649.0), (2040.00, 17878.0), (2041.00, 18096.0), (2042.00, 18302.0), (2043.00, 18495.0), (2044.00, 18675.0), (2045.00, 18843.0), (2046.00, 18995.0), (2047.00, 19140.0), (2048.00, 19288.0), (2049.00, 19440.0), (2050.00, 19599.0), (2051.00, 19767.0), (2052.00, 19945.0), (2053.00, 20135.0), (2054.00, 20336.0), (2055.00, 20546.0), (2056.00, 20770.0), (2057.00, 21003.0), (2058.00, 21244.0), (2059.00, 21494.0), (2060.00, 21749.0), (2061.00, 22007.0)

UNITS: persons/year

NIM_Departures_H[F, A24] = GRAPH(TIME)

Points: (2011.00, 24200.0), (2012.00, 25110.0), (2013.00, 25430.0), (2014.00, 23760.0), (2015.00, 23010.0), (2016.00, 24220.0), (2017.00, 24350.0), (2018.00, 26713.0), (2019.00, 26944.0), (2020.00, 27088.0), (2021.00, 27289.0), (2022.00, 27437.0), (2023.00, 27636.0), (2024.00, 27828.0), (2025.00, 28099.0), (2026.00, 28537.0), (2027.00, 29051.0), (2028.00, 29667.0), (2029.00, 30299.0), (2030.00, 30891.0), (2031.00, 31271.0), (2032.00, 31798.0), (2033.00, 32309.0), (2034.00, 32860.0), (2035.00, 33458.0), (2036.00, 34196.0), (2037.00, 34820.0), (2038.00, 35408.0), (2039.00, 35993.0), (2040.00, 36567.0), (2041.00, 37123.0), (2042.00, 37655.0), (2043.00, 38161.0), (2044.00, 38644.0), (2045.00, 39101.0), (2046.00, 39536.0), (2047.00, 39946.0), (2048.00, 40327.0), (2049.00, 40680.0), (2050.00, 41008.0), (2051.00, 41315.0), (2052.00, 41613.0), (2053.00, 41918.0), (2054.00, 42235.0), (2055.00, 42569.0), (2056.00, 42919.0), (2057.00, 43292.0), (2058.00, 43687.0), (2059.00, 44102.0), (2060.00, 44543.0), (2061.00, 45003.0)

UNITS: persons/year

NIM_Departures_H[F, A29] = GRAPH(TIME)

Points: (2011.00, 25540.0), (2012.00, 26140.0), (2013.00, 27210.0), (2014.00, 25850.0), (2015.00, 26540.0), (2016.00, 28290.0), (2017.00, 28450.0), (2018.00, 28399.0), (2019.00, 28633.0), (2020.00, 28860.0), (2021.00, 29051.0), (2022.00, 29322.0), (2023.00, 29539.0), (2024.00, 29770.0), (2025.00, 29930.0), (2026.00, 30130.0), (2027.00, 30270.0), (2028.00, 30464.0), (2029.00, 30678.0), (2030.00, 30988.0), (2031.00, 31484.0), (2032.00, 32013.0), (2033.00, 32622.0), (2034.00, 33222.0), (2035.00, 33787.0), (2036.00, 34141.0), (2037.00, 34669.0), (2038.00, 35218.0), (2039.00, 35794.0), (2040.00, 36395.0), (2041.00, 37102.0), (2042.00, 37716.0), (2043.00, 38303.0), (2044.00, 38884.0), (2045.00, 39450.0), (2046.00, 39998.0), (2047.00, 40521.0), (2048.00, 41020.0), (2049.00, 41494.0), (2050.00, 41945.0), (2051.00, 42376.0), (2052.00, 42779.0), (2053.00, 43150.0), (2054.00, 43494.0), (2055.00, 43817.0), (2056.00, 44123.0), (2057.00, 44425.0), (2058.00, 44734.0), (2059.00, 45055.0), (2060.00, 45394.0), (2061.00, 45751.0)

UNITS: persons/year

NIM_Departures_H[F, A34] = GRAPH(TIME)

Points: (2011.00, 18390.0), (2012.00, 17710.0), (2013.00, 18900.0), (2014.00, 18820.0), (2015.00, 20650.0), (2016.00, 22200.0), (2017.00, 22350.0), (2018.00, 22078.0), (2019.00, 22473.0), (2020.00, 22882.0), (2021.00, 23246.0), (2022.00, 23520.0), (2023.00, 23751.0), (2024.00, 23932.0), (2025.00, 24108.0), (2026.00, 24251.0), (2027.00, 24455.0), (2028.00, 24617.0), (2029.00, 24789.0), (2030.00, 24910.0), (2031.00, 25059.0), (2032.00, 25164.0), (2033.00, 25310.0), (2034.00, 25474.0), (2035.00, 25708.0), (2036.00, 26084.0), (2037.00, 26482.0), (2038.00, 26941.0), (2039.00, 27394.0), (2040.00, 27817.0), (2041.00, 28080.0), (2042.00, 28480.0), (2043.00, 28893.0), (2044.00, 29327.0), (2045.00, 29780.0), (2046.00, 30308.0), (2047.00, 30771.0), (2048.00, 31213.0), (2049.00, 31649.0), (2050.00, 32075.0), (2051.00, 32488.0), (2052.00, 32882.0), (2053.00, 33257.0), (2054.00, 33614.0), (2055.00, 33952.0), (2056.00, 34277.0), (2057.00, 34581.0), (2058.00, 34860.0), (2059.00, 35120.0), (2060.00, 35363.0), (2061.00, 35595.0)

UNITS: persons/year

NIM_Departures_H[F, A39] = GRAPH(TIME)

Points: (2011.00, 14300.0), (2012.00, 12580.0), (2013.00, 13400.0), (2014.00, 12800.0), (2015.00, 13890.0), (2016.00, 15350.0), (2017.00, 15660.0), (2018.00, 15050.0), (2019.00, 15588.0), (2020.00, 16103.0), (2021.00, 16504.0), (2022.00, 16835.0), (2023.00, 17104.0), (2024.00, 17393.0), (2025.00, 17692.0), (2026.00, 17952.0), (2027.00, 18145.0), (2028.00, 18310.0), (2029.00, 18441.0), (2030.00, 18568.0), (2031.00, 18670.0), (2032.00, 18817.0), (2033.00, 18931.0), (2034.00, 19056.0), (2035.00, 19146.0), (2036.00, 19250.0), (2037.00, 19323.0), (2038.00, 19430.0), (2039.00, 19549.0), (2040.00, 19720.0), (2041.00, 19993.0), (2042.00, 20282.0), (2043.00, 20611.0), (2044.00, 20935.0), (2045.00, 21240.0), (2046.00, 21429.0), (2047.00, 21718.0), (2048.00, 22017.0), (2049.00, 22330.0), (2050.00, 22656.0), (2051.00, 23037.0), (2052.00, 23372.0), (2053.00, 23690.0), (2054.00, 24005.0), (2055.00, 24313.0), (2056.00, 24611.0), (2057.00, 24895.0), (2058.00, 25164.0), (2059.00, 25422.0), (2060.00, 25667.0), (2061.00, 25900.0)

UNITS: persons/year

NIM_Departures_H[F, A44] = GRAPH(TIME)

Points: (2011.00, 10350.0), (2012.00, 9440.0), (2013.00, 10600.0), (2014.00, 9630.0), (2015.00, 10240.0), (2016.00, 10980.0), (2017.00, 11120.0), (2018.00, 9876.0), (2019.00, 9980.0), (2020.00, 10172.0), (2021.00, 10475.0), (2022.00, 10822.0), (2023.00, 11228.0), (2024.00, 11610.0), (2025.00, 11979.0), (2026.00, 12265.0), (2027.00, 12501.0), (2028.00, 12693.0), (2029.00, 12897.0), (2030.00, 13108.0), (2031.00, 13293.0), (2032.00, 13433.0), (2033.00, 13553.0), (2034.00, 13646.0), (2035.00, 13735.0), (2036.00, 13810.0), (2037.00, 13916.0), (2038.00, 13999.0), (2039.00, 14085.0), (2040.00, 14150.0), (2041.00, 14225.0), (2042.00, 14278.0), (2043.00, 14353.0), (2044.00, 14438.0), (2045.00, 14558.0), (2046.00, 14751.0), (2047.00, 14954.0), (2048.00, 15189.0), (2049.00, 15420.0), (2050.00, 15636.0), (2051.00, 15772.0), (2052.00, 15974.0), (2053.00, 16187.0), (2054.00, 16408.0), (2055.00, 16641.0), (2056.00, 16912.0), (2057.00, 17150.0), (2058.00, 17377.0), (2059.00, 17600.0), (2060.00, 17819.0), (2061.00, 18032.0)

UNITS: persons/year

NIM_Departures_H[F, A49] = GRAPH(TIME)

Points: (2011.00, 7700.0), (2012.00, 7240.0), (2013.00, 7690.0), (2014.00, 7170.0), (2015.00, 7610.0), (2016.00, 8620.0), (2017.00, 9070.0), (2018.00, 8502.0), (2019.00, 8545.0), (2020.00, 8545.0), (2021.00, 8435.0), (2022.00, 8357.0), (2023.00, 8358.0), (2024.00, 8439.0), (2025.00, 8590.0), (2026.00, 8830.0), (2027.00, 9112.0), (2028.00, 9441.0), (2029.00, 9755.0), (2030.00, 10060.0), (2031.00, 10297.0), (2032.00, 10497.0), (2033.00, 10656.0), (2034.00, 10826.0), (2035.00, 10996.0), (2036.00, 11149.0), (2037.00, 11269.0), (2038.00, 11372.0), (2039.00, 11453.0), (2040.00, 11524.0), (2041.00, 11585.0), (2042.00, 11673.0), (2043.00, 11742.0), (2044.00, 11818.0), (2045.00, 11869.0), (2046.00, 11932.0), (2047.00, 11977.0), (2048.00, 12041.0), (2049.00, 12109.0), (2050.00, 12205.0), (2051.00, 12359.0), (2052.00, 12526.0), (2053.00, 12719.0), (2054.00, 12909.0), (2055.00, 13091.0), (2056.00, 13204.0), (2057.00, 13371.0), (2058.00, 13542.0), (2059.00, 13725.0), (2060.00, 13912.0), (2061.00, 14137.0)

UNITS: persons/year

NIM_Departures_H[F, A54] = GRAPH(TIME)

Points: (2011.00, 7140.0), (2012.00, 7040.0), (2013.00, 7400.0), (2014.00, 6650.0), (2015.00, 6810.0), (2016.00, 7850.0), (2017.00, 8200.0), (2018.00, 7447.0), (2019.00, 7535.0), (2020.00, 7688.0), (2021.00, 7956.0), (2022.00, 8204.0), (2023.00, 8363.0), (2024.00, 8414.0), (2025.00, 8421.0), (2026.00, 8313.0), (2027.00, 8236.0), (2028.00, 8235.0), (2029.00, 8309.0), (2030.00, 8454.0), (2031.00, 8683.0), (2032.00, 8954.0), (2033.00, 9272.0), (2034.00, 9579.0), (2035.00, 9877.0), (2036.00, 10113.0), (2037.00, 10313.0), (2038.00, 10468.0), (2039.00, 10634.0), (2040.00, 10799.0), (2041.00, 10952.0), (2042.00, 11076.0), (2043.00, 11179.0), (2044.00, 11259.0), (2045.00, 11329.0), (2046.00, 11391.0), (2047.00, 11479.0), (2048.00, 11549.0), (2049.00, 11624.0), (2050.00, 11676.0), (2051.00, 11737.0), (2052.00, 11785.0), (2053.00, 11848.0), (2054.00, 11916.0), (2055.00, 12009.0), (2056.00, 12156.0), (2057.00, 12320.0), (2058.00, 12509.0), (2059.00, 12698.0), (2060.00, 12876.0), (2061.00, 12991.0)

UNITS: persons/year

NIM_Departures_H[F, A59] = GRAPH(TIME)

Points: (2011.00, 5980.0), (2012.00, 6080.0), (2013.00, 6410.0), (2014.00, 5930.0), (2015.00, 6380.0), (2016.00, 7380.0), (2017.00, 8040.0), (2018.00, 7280.0), (2019.00, 7354.0), (2020.00, 7370.0), (2021.00, 7330.0), (2022.00, 7297.0), (2023.00, 7302.0), (2024.00, 7391.0), (2025.00, 7543.0), (2026.00, 7807.0), (2027.00, 8051.0), (2028.00, 8204.0), (2029.00, 8254.0), (2030.00, 8256.0), (2031.00, 8157.0), (2032.00, 8087.0), (2033.00, 8091.0), (2034.00, 8167.0), (2035.00, 8311.0), (2036.00, 8538.0), (2037.00, 8803.0), (2038.00, 9116.0), (2039.00, 9416.0), (2040.00, 9706.0), (2041.00, 9938.0), (2042.00, 10130.0), (2043.00, 10284.0), (2044.00, 10446.0), (2045.00, 10611.0), (2046.00, 10763.0), (2047.00, 10884.0), (2048.00, 10986.0), (2049.00, 11064.0), (2050.00, 11135.0), (2051.00, 11198.0), (2052.00, 11286.0), (2053.00, 11357.0), (2054.00, 11431.0), (2055.00, 11484.0), (2056.00, 11547.0), (2057.00, 11595.0), (2058.00, 11659.0), (2059.00, 11727.0), (2060.00, 11822.0), (2061.00, 11968.0)

UNITS: persons/year

NIM_Departures_H[F, A64] = GRAPH(TIME)

Points: (2011.00, 4730.0), (2012.00, 4720.0), (2013.00, 5310.0), (2014.00, 4960.0), (2015.00, 5210.0), (2016.00, 6270.0), (2017.00, 6430.0), (2018.00, 5651.0), (2019.00, 5793.0), (2020.00, 5922.0), (2021.00, 6072.0), (2022.00, 6204.0), (2023.00, 6307.0), (2024.00, 6374.0), (2025.00, 6390.0), (2026.00, 6356.0), (2027.00, 6332.0), (2028.00, 6340.0), (2029.00, 6421.0), (2030.00, 6554.0), (2031.00, 6788.0), (2032.00, 6996.0), (2033.00, 7127.0), (2034.00, 7169.0), (2035.00, 7173.0), (2036.00, 7090.0), (2037.00, 7033.0), (2038.00, 7038.0), (2039.00, 7106.0), (2040.00, 7233.0), (2041.00, 7430.0), (2042.00, 7660.0), (2043.00, 7933.0), (2044.00, 8191.0), (2045.00, 8442.0), (2046.00, 8643.0), (2047.00, 8810.0), (2048.00, 8944.0), (2049.00, 9086.0), (2050.00, 9231.0), (2051.00, 9361.0), (2052.00, 9467.0), (2053.00, 9555.0), (2054.00, 9625.0), (2055.00, 9689.0), (2056.00, 9745.0), (2057.00, 9823.0), (2058.00, 9886.0), (2059.00, 9951.0), (2060.00, 9997.0), (2061.00, 10054.0)

UNITS: persons/year

NIM_Departures_H[F, A69] = GRAPH(TIME)

Points: (2011.00, 2820.0), (2012.00, 3070.0), (2013.00, 3560.0), (2014.00, 3510.0), (2015.00, 3910.0), (2016.00, 4390.0), (2017.00, 4650.0), (2018.00, 3947.0), (2019.00, 4039.0), (2020.00, 4136.0), (2021.00, 4237.0), (2022.00, 4343.0), (2023.00, 4448.0), (2024.00, 4563.0), (2025.00, 4669.0), (2026.00, 4792.0), (2027.00, 4898.0), (2028.00, 4982.0), (2029.00, 5036.0), (2030.00, 5049.0), (2031.00, 5028.0), (2032.00, 5013.0), (2033.00, 5024.0), (2034.00, 5096.0), (2035.00, 5204.0), (2036.00, 5395.0), (2037.00, 5560.0), (2038.00, 5658.0), (2039.00, 5690.0), (2040.00, 5692.0), (2041.00, 5629.0), (2042.00, 5588.0), (2043.00, 5597.0), (2044.00, 5655.0), (2045.00, 5759.0), (2046.00, 5920.0), (2047.00, 6107.0), (2048.00, 6324.0), (2049.00, 6530.0), (2050.00, 6729.0), (2051.00, 6890.0), (2052.00, 7022.0), (2053.00, 7131.0), (2054.00, 7248.0), (2055.00, 7365.0), (2056.00, 7472.0), (2057.00, 7557.0), (2058.00, 7629.0), (2059.00, 7687.0), (2060.00, 7741.0), (2061.00, 7786.0)

UNITS: persons/year

NIM_Departures_H[F, A74] = GRAPH(TIME)

Points: (2011.00, 1540.0), (2012.00, 1690.0), (2013.00, 2000.0), (2014.00, 1840.0), (2015.00, 2210.0), (2016.00, 2550.0), (2017.00, 2830.0), (2018.00, 2577.0), (2019.00, 2670.0), (2020.00, 2760.0), (2021.00, 2854.0), (2022.00, 2871.0), (2023.00, 2933.0), (2024.00, 3003.0), (2025.00, 3078.0), (2026.00, 3155.0), (2027.00, 3239.0), (2028.00, 3320.0), (2029.00, 3409.0), (2030.00, 3491.0), (2031.00, 3588.0), (2032.00, 3671.0), (2033.00, 3736.0), (2034.00, 3778.0), (2035.00, 3791.0), (2036.00, 3776.0), (2037.00, 3766.0), (2038.00, 3774.0), (2039.00, 3829.0), (2040.00, 3911.0), (2041.00, 4054.0), (2042.00, 4178.0), (2043.00, 4254.0), (2044.00, 4278.0), (2045.00, 4285.0), (2046.00, 4240.0), (2047.00, 4209.0), (2048.00, 4214.0), (2049.00, 4257.0), (2050.00, 4334.0), (2051.00, 4455.0), (2052.00, 4592.0), (2053.00, 4755.0), (2054.00, 4909.0), (2055.00, 5061.0), (2056.00, 5177.0), (2057.00, 5278.0), (2058.00, 5358.0), (2059.00, 5447.0), (2060.00, 5536.0), (2061.00, 5616.0)

UNITS: persons/year

NIM_Departures_H[F, A79] = GRAPH(TIME)

Points: (2011.00, 1071.0), (2012.00, 1086.0), (2013.00, 1235.0), (2014.00, 1130.0), (2015.00, 1140.0), (2016.00, 1184.0), (2017.00, 1275.0), (2018.00, 1571.0), (2019.00, 1656.0), (2020.00, 1748.0), (2021.00, 1840.0), (2022.00, 1986.0), (2023.00, 2106.0), (2024.00, 2185.0), (2025.00, 2259.0), (2026.00, 2344.0), (2027.00, 2360.0), (2028.00, 2412.0), (2029.00, 2476.0), (2030.00, 2541.0), (2031.00, 2608.0), (2032.00, 2682.0), (2033.00, 2753.0), (2034.00, 2831.0), (2035.00, 2904.0), (2036.00, 2985.0), (2037.00, 3058.0), (2038.00, 3116.0), (2039.00, 3155.0), (2040.00, 3171.0), (2041.00, 3159.0), (2042.00, 3155.0), (2043.00, 3163.0), (2044.00, 3207.0), (2045.00, 3278.0), (2046.00, 3397.0), (2047.00, 3507.0), (2048.00, 3576.0), (2049.00, 3599.0), (2050.00, 3612.0), (2051.00, 3575.0), (2052.00, 3550.0), (2053.00, 3556.0), (2054.00, 3593.0), (2055.00, 3658.0), (2056.00, 3761.0), (2057.00, 3879.0), (2058.00, 4017.0), (2059.00, 4150.0), (2060.00, 4279.0), (2061.00, 4380.0)

UNITS: persons/year

NIM_Departures_H[F, A84] = GRAPH(TIME)

Points: (2011.00, 911.0), (2012.00, 924.0), (2013.00, 1050.0), (2014.00, 961.0), (2015.00, 970.0), (2016.00, 1007.0), (2017.00, 1084.0), (2018.00, 1144.0), (2019.00, 1186.0), (2020.00, 1229.0), (2021.00, 1275.0), (2022.00, 1334.0), (2023.00, 1391.0), (2024.00, 1474.0), (2025.00, 1557.0), (2026.00, 1645.0), (2027.00, 1783.0), (2028.00, 1890.0), (2029.00, 1975.0), (2030.00, 2052.0), (2031.00, 2129.0), (2032.00, 2149.0), (2033.00, 2204.0), (2034.00, 2266.0), (2035.00, 2331.0), (2036.00, 2398.0), (2037.00, 2469.0), (2038.00, 2541.0), (2039.00, 2616.0), (2040.00, 2688.0), (2041.00, 2769.0), (2042.00, 2840.0), (2043.00, 2902.0), (2044.00, 2945.0), (2045.00, 2964.0), (2046.00, 2962.0), (2047.00, 2958.0), (2048.00, 2971.0), (2049.00, 3014.0), (2050.00, 3081.0), (2051.00, 3200.0), (2052.00, 3306.0), (2053.00, 3378.0), (2054.00, 3413.0), (2055.00, 3427.0), (2056.00, 3395.0), (2057.00, 3373.0), (2058.00, 3381.0), (2059.00, 3419.0), (2060.00, 3486.0), (2061.00, 3586.0)

UNITS: persons/year

NIM_Departures_H[F, A89] = GRAPH(TIME)

Points: (2011.00, 623.0), (2012.00, 631.0), (2013.00, 718.0), (2014.00, 657.0), (2015.00, 663.0), (2016.00, 688.0), (2017.00, 741.0), (2018.00, 831.0), (2019.00, 836.0), (2020.00, 846.0), (2021.00, 863.0), (2022.00, 890.0), (2023.00, 921.0), (2024.00, 958.0), (2025.00, 999.0), (2026.00, 1041.0), (2027.00, 1094.0), (2028.00, 1147.0), (2029.00, 1222.0), (2030.00, 1298.0), (2031.00, 1378.0), (2032.00, 1505.0), (2033.00, 1604.0), (2034.00, 1678.0), (2035.00, 1751.0), (2036.00, 1816.0), (2037.00, 1845.0), (2038.00, 1902.0), (2039.00, 1963.0), (2040.00, 2028.0), (2041.00, 2097.0), (2042.00, 2166.0), (2043.00, 2239.0), (2044.00, 2313.0), (2045.00, 2384.0), (2046.00, 2465.0), (2047.00, 2536.0), (2048.00, 2598.0), (2049.00, 2641.0), (2050.00, 2663.0), (2051.00, 2667.0), (2052.00, 2672.0), (2053.00, 2692.0), (2054.00, 2747.0), (2055.00, 2819.0), (2056.00, 2940.0), (2057.00, 3046.0), (2058.00, 3115.0), (2059.00, 3152.0), (2060.00, 3163.0), (2061.00, 3142.0)

UNITS: persons/year

NIM_Departures_H[F, A94] = GRAPH(TIME)

Points: (2011.00, 263.0), (2012.00, 267.0), (2013.00, 304.0), (2014.00, 278.0), (2015.00, 280.0), (2016.00, 291.0), (2017.00, 314.0), (2018.00, 414.0), (2019.00, 421.0), (2020.00, 431.0), (2021.00, 438.0), (2022.00, 442.0), (2023.00, 445.0), (2024.00, 450.0), (2025.00, 458.0), (2026.00, 472.0), (2027.00, 491.0), (2028.00, 509.0), (2029.00, 534.0), (2030.00, 560.0), (2031.00, 585.0), (2032.00, 621.0), (2033.00, 657.0), (2034.00, 706.0), (2035.00, 761.0), (2036.00, 814.0), (2037.00, 901.0), (2038.00, 968.0), (2039.00, 1015.0), (2040.00, 1068.0), (2041.00, 1120.0), (2042.00, 1148.0), (2043.00, 1196.0), (2044.00, 1245.0), (2045.00, 1296.0), (2046.00, 1351.0), (2047.00, 1408.0), (2048.00, 1467.0), (2049.00, 1527.0), (2050.00, 1588.0), (2051.00, 1653.0), (2052.00, 1712.0), (2053.00, 1763.0), (2054.00, 1806.0), (2055.00, 1832.0), (2056.00, 1847.0), (2057.00, 1865.0), (2058.00, 1895.0), (2059.00, 1947.0), (2060.00, 2011.0), (2061.00, 2109.0)

UNITS: persons/year

NIM_Departures_H[F, A99] = GRAPH(TIME)

Points: (2011.00, 69.0), (2012.00, 70.0), (2013.00, 79.0), (2014.00, 73.0), (2015.00, 73.0), (2016.00, 76.0), (2017.00, 82.0), (2018.00, 115.0), (2019.00, 122.0), (2020.00, 127.0), (2021.00, 131.0), (2022.00, 134.0), (2023.00, 139.0), (2024.00, 141.0), (2025.00, 145.0), (2026.00, 148.0), (2027.00, 150.0), (2028.00, 151.0), (2029.00, 154.0), (2030.00, 158.0), (2031.00, 165.0), (2032.00, 173.0), (2033.00, 182.0), (2034.00, 194.0), (2035.00, 208.0), (2036.00, 223.0), (2037.00, 243.0), (2038.00, 261.0), (2039.00, 288.0), (2040.00, 316.0), (2041.00, 345.0), (2042.00, 392.0), (2043.00, 427.0), (2044.00, 456.0), (2045.00, 488.0), (2046.00, 519.0), (2047.00, 543.0), (2048.00, 574.0), (2049.00, 608.0), (2050.00, 644.0), (2051.00, 683.0), (2052.00, 723.0), (2053.00, 764.0), (2054.00, 807.0), (2055.00, 851.0), (2056.00, 899.0), (2057.00, 945.0), (2058.00, 986.0), (2059.00, 1022.0), (2060.00, 1049.0), (2061.00, 1071.0)

UNITS: persons/year

NIM_Departures_H[F, A104] = GRAPH(TIME)

Points: (2011.00, 9.0), (2012.00, 9.0), (2013.00, 11.0), (2014.00, 10.0), (2015.00, 10.0), (2016.00, 10.0), (2017.00, 11.0), (2018.00, 21.0), (2019.00, 22.0), (2020.00, 24.0), (2021.00, 27.0), (2022.00, 29.0), (2023.00, 31.0), (2024.00, 33.0), (2025.00, 35.0), (2026.00, 36.0), (2027.00, 38.0), (2028.00, 39.0), (2029.00, 40.0), (2030.00, 42.0), (2031.00, 43.0), (2032.00, 44.0), (2033.00, 45.0), (2034.00, 47.0), (2035.00, 49.0), (2036.00, 53.0), (2037.00, 57.0), (2038.00, 61.0), (2039.00, 67.0), (2040.00, 73.0), (2041.00, 81.0), (2042.00, 90.0), (2043.00, 99.0), (2044.00, 112.0), (2045.00, 125.0), (2046.00, 140.0), (2047.00, 162.0), (2048.00, 180.0), (2049.00, 198.0), (2050.00, 216.0), (2051.00, 236.0), (2052.00, 256.0), (2053.00, 279.0), (2054.00, 302.0), (2055.00, 328.0), (2056.00, 355.0), (2057.00, 383.0), (2058.00, 413.0), (2059.00, 445.0), (2060.00, 478.0), (2061.00, 515.0)

UNITS: persons/year

NIM_Departures_L[M, A4] = GRAPH(TIME)
[truncated: 429,358 more chars]
