# Supplementary material for: Gazing through time and beyond the health sector: Insights from a system dynamics model of cardiovascular disease in Australia
Source: PLoS One. 2021 Sep 30;16(9):e0257760. doi: 10.1371/journal.pone.0257760 (PMC8483334; doi:10.1371/journal.pone.0257760)
Supplement: Attachment — Submitted filename: Response to reviewers.docx [file pone.0257760.s002.docx]

# Response to reviewers

Reviewer #1: This is a very good article. There are only three omissions that need to be taken care of to make it an even better article.

1) You mention a group model building activity that provided the foundation of the model. It would be useful for the reader to know more about the insights that came out of that activity in the form of one or more causal diagrams that show some of the relationships that link interventions and outcomes.

Author response: Thanks. We have added a paragraph in page 19 discussing the learnings and our use of group model building. Since the goal was to build a simulation model and we anticipated the structure to be largely mechanistic based on similar CVD models, we used the participatory process to discuss mechanisms and focused on stock-and-flow structures rather than causal diagrams. The mechanisms and assumptions of interventions are described in details in the Appendix.

2) The results shown in Table 2 require better explanation. For example, why do the results for the Medication and Treatment strategy look so much worse than the Supportive Environment strategy. To what extent is this because you have estimates for the interventions that make up the Medication and Treatment strategy and don't estimate costs for the Supportive environment? Why does the Pre-Hospital strategy produce a significant health system benefit? I would have expected better Pre-Hospital interventions would have saved lives of people who then require expensive hospital and follow up care including recurrent acute-CVD. If I've misunderstood, you need to explain better. In general, for results in both the graphs and tables, you need to relate the results better to the model's structure rather than simply reporting the results.

Author response: We appreciate Reviewer 1 pointing these out. We have added explanations in pages 15 and 17 to clarify. In general, the findings were described at a high-level rather than as explicit reference to model structure. For example, the reason for an increase in hospitalisations as a result of improvement in response to acute events was alluded to as survivors experiencing recurrent hospitalisations.

3) It would be interesting to see the impact of combined strategies and whether they can produce better results than single ones by complementing each others' strengths. This is often a lesson derived from SD health care and other models and a valuable lesson the those who might advocate for a particular class of interventions to the exclusion of others.

Author response: Thank you, a valid point. We tested different combinations of strategies, which led to no significant findings. The model included a diverse range of interventions targeting different mechanisms, and it is largely compartmental in nature, with limited feedback loops. We agree that future modelling with a narrower scope and more depth should explore the interactions between different drivers and policies, and that synergistic effects would be important to note.

Reviewer #2: The article reports on outcomes of a participatory modeling projects to explore health policies that address the problem of cardiovascular diseases in Australia. Overall, the contribution of this work comes as a result of its participatory method (group model building) which brings many experts together and builds a simulation model that is consistent with the shared mental model of the participants. There is a rich history of group model building in system dynamics, and the method has proved to be effective to overcome modeling and implementation challenges.
Overall, I think this is a good work, and my suggestions are about improving the writings.

1- Please provide a better reference to the method and new developments in Group Model Building (GMB). GMB was introduced by George Richardson and David Andersen in their seminal works in 90s.
• Richardson, G. P., & Andersen, D. F. (1995). Teamwork in group model building. System Dynamics Review, 11(2), 113-137.
• Andersen, D. F., & Richardson, G. P. (1997). Scripts for group model building. System Dynamics Review: The Journal of the System Dynamics Society, 13(2), 107-129.
New advancements in the method including parameter estimation in group settings, model as a boundary object, and stakeholder analysis in GMBs are reported in these works:
• Luna‐Reyes, L. F., Black, L. J., Ran, W., Andersen, D. L., Jarman, H., Richardson, G. P., & Andersen, D. F. (2019). Modeling and simulation as boundary objects to facilitate interdisciplinary research. Systems Research and Behavioral Science, 36(4), 494-513.
• Hosseinichimeh, N., MacDonald, R., Hyder, A., Ebrahimvandi, A., Porter, L., Reno, R., ... & Andersen, D. F. (2017). Group model building techniques for rapid elicitation of parameter values, effect sizes, and data sources. System Dynamics Review, 33(1), 71-84.

Author response: Thank you. We have referenced the publication from Anderson and Richardson as the most relevant one to the process we have used (page 19).

2. Related to the first item, I strongly suggest the author to add a short reflection at the end to describes its methodological contributions, by reflecting on how the process worked, what they learned, and how they can further improve it, by potentially comparing and contrasting their works with the ones mentioned above. A paragraph in discussion would be enough.

Author response: We have added a paragraph on page 19 briefly discussing our process and how it compares to the group model building used in SD. Methodological differences with other non-SD modelling studies of CVD are discussed on pages 18 – 20.

3. Their model documentation is not meeting standards of a modeling work, and the need for reproducibility of scientific studies. To that end, first, the simulation model should be provided as a supplement. Second, the model equations (in mathemtaical format) should be all documented, so in case someone does not use Stella can replicate their findings in other softwares. Please refer to Rahmandad and Sterman’s article on minimum requirements for model documentation.
• Rahmandad, H., & Sterman, J. D. (2012). Reporting guidelines for simulation-based research in social sciences. System Dynamics Review 28(4): 396 – 411.
But I admit that the authors do a very good job of documenting parameter values and experimental designs in the Appendix.

Author response: We have added the full list of equations in the Appendix. We have also added that the simulation model can be provided on request.

4. I could not read the figures. The quality of the graphs, especially Figure 2, were very low. So my judgments are mainly based on the text. Please fix the figures.

Author response: We have re-produced the figures for clarity.

5. Figure 1, the model: please add the inflow(s). (Looks to me the only inflow is birth rate and goes to No CVD). Further, guide the readers to go through the model by providing a descriptive text.

Author response: We have added an inflow of ‘turning 40 years of age’. A descriptive text of the model structure follows the reference to Figure 1 on page 9.

6. It appears that there are not many (any?) feedback loops in your model. This is fine as Homer and colleagues’ works of modeling cardiovascular disease also does not have many (any?) feedback loops. In limitations of the paper, please mention that the modelers focused on the compartmental modeling aspect of the project, and the project will benefit from exploring feedback mechanisms that can further affect the health statuses and demand for healthcare.

Author response: This is now covered by the new paragraph on page 19.

7. There is a history of health models in System Dynamics and specifically CVDs. Please check this source, as a very good literature review, and make sure major CVD modelings are not missed:
• Darabi, N., & Hosseinichimeh, N. (2020). System dynamics modeling in health and medicine: a systematic literature review. System Dynamics Review, 36(1), 29-73.

Author response: We have checked the CVD models cited in this systematic review and found no significant discrepancy. This systematic review has now been referenced on page 19.

8. Minor: It might be better to spell out cardiovascular disease in the title, and maybe the first time it appears in the abstract, as CVD might not be a familiar term for many (I note that you mention it in the first page, which is good, but the title and abstract are unclear).

Author response: This has been amended.

9. One of the findings of this work is that better screening, while helpful, leads to more demand for hospitalizations. That’s interesting (and you may talk more about it).

Author response: Screening leads to greater disease detection, and in some cases, higher demand for hospitalisations. However, we did not look at screening specifically. There is a finding that improvement in response to acute cardiovascular events leads to higher demand for hospitalisations, since the survivors can experience further hospitalisations. This is discussed on page 17.
